# The Mitochondrial Genome of the Lycophyte *Huperzia squarrosa:* The Most Archaic Form in Vascular Plants

**DOI:** 10.1371/journal.pone.0035168

**Published:** 2012-04-12

**Authors:** Yang Liu, Bin Wang, Peng Cui, Libo Li, Jia-Yu Xue, Jun Yu, Yin-Long Qiu

**Affiliations:** 1 Department of Ecology and Evolutionary Biology, University of Michigan, Ann Arbor, Michigan, United States of America; 2 CAS Key Laboratory of Genome Science and Information, Beijing Institute of Genomics, Chinese Academy of Sciences, Beijing, People's Republic of China; 3 School of Life Sciences, Nanjing University, Nanjing, Jiangsu, People's Republic of China; University of Melbourne, Australia

## Abstract

Mitochondrial genomes have maintained some bacterial features despite their residence within eukaryotic cells for approximately two billion years. One of these features is the frequent presence of polycistronic operons. In land plants, however, it has been shown that all sequenced vascular plant chondromes lack large polycistronic operons while bryophyte chondromes have many of them. In this study, we provide the completely sequenced mitochondrial genome of a lycophyte, from *Huperzia squarrosa*, which is a member of the sister group to all other vascular plants. The genome, at a size of 413,530 base pairs, contains 66 genes and 32 group II introns. In addition, it has 69 pseudogene fragments for 24 of the 40 protein- and rRNA-coding genes. It represents the most archaic form of mitochondrial genomes of all vascular plants. In particular, it has one large conserved gene cluster containing up to 10 ribosomal protein genes, which likely represents a polycistronic operon but has been disrupted and greatly reduced in the chondromes of other vascular plants. It also has the least rearranged gene order in comparison to the chondromes of other vascular plants. The genome is ancestral in vascular plants in several other aspects: the gene content resembling those of charophytes and most bryophytes, all introns being *cis*-spliced, a low level of RNA editing, and lack of foreign DNA of chloroplast or nuclear origin.

## Introduction

Mitochondria are the cellular power houses of nearly all eukaryotes [1]. Extensive sequencing of their genomes over the last three decades reveals that this organellar genome has maintained one of its ancestral bacterial features in most protists, fungi, animals, and early land plants: genes being organized into large syntenic blocks, many of which represent polycistronic operons [1], [2], [3], [4], [5], [6], [7]. A major exception, however, is found in flowering plants, whose chondromes contain mostly free-standing genes with their own transcriptional regulatory elements [7], [8], [9], [10], [11], [12]. Recent sequencing of a chondrome from the gymnosperm *Cycas taitungensis* [13] shows that this type of derived mitochondrial genome is likely shared by all seed plants. When this type of mitochondrial genome with a unique gene organization and transcription system arose in plant evolution has been a long-standing question in mitochondrial research. Sequencing of chondromes from representatives of major lineages of charophytic algae [14], [15], [16], [17] and land plants [13], [18], [19], [20], [21], [22], [23], [24], [25], [26], [27] suggests that early vascular plants are likely the groups where the genome experienced a major change. In this study we report the completely sequenced mitochondrial genome of a lycophyte, from *Huperzia squarrosa* of Lycopodiaceae, which bridges the gap between the ancestral type of mitochondrial genomes found in bryophytes and the derived type in seed plants.

Lycophytes are sister to all other vascular plants [28], [29], and hence are an appropriate group for investigating the ancestral condition of mitochondrial genome in vascular plants. There are three lineages within lycophytes: Lycopodiaceae, Isoetaceae, and Selaginellaceae [30], [31], [32]. Lycopodiaceae represent the basalmost clade of lycophytes [29]; a species in the family becomes a natural choice to look for the most archaic chondrome of all vascular plants. Recent reports of nearly complete chondromes from *Isoetes* and *Selaginella* show that mitochondrial genomes in these two lineages have independently acquired some features found in angiosperm chondromes, e.g., rapid rearrangement of gene order, loss of many ribosomal protein and tRNA genes, *trans*-splicing of introns, heavy RNA editing, and invasion of foreign DNAs of chloroplast and nuclear origins [24], [25]. These studies make it more urgent to sequence a chondrome from Lycopodiaceae so that the accurate state of mitochondrial genome in the basalmost vascular plants can be determined.

## Results and Discussion

### General Features of the *Huperzia* Mitochondrial Genome

**Figure 1:**
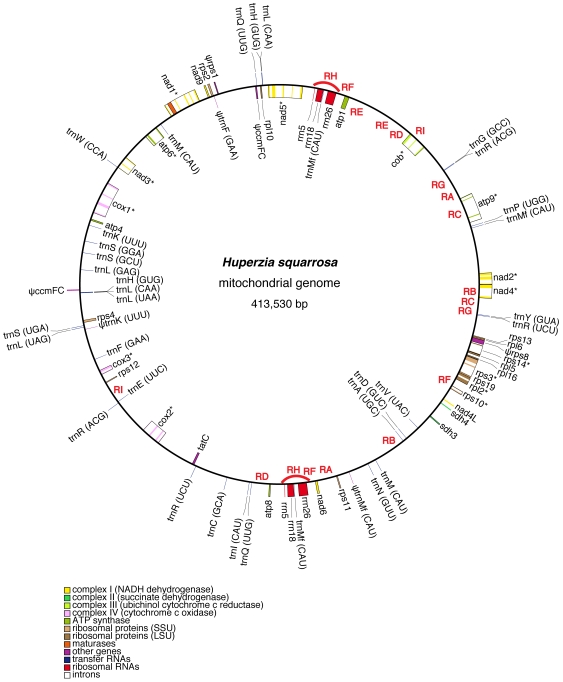
The gene map of *Huperzia squarrosa* mitochondrial genome. Genes (exons indicated as closed boxes) shown on the outside of the circle are transcribed counter-clockwise, whereas those on the inside are transcribed clockwise. Genes with group II introns (open boxes) are labeled with asterisks. Pseudogenes are indicated with the prefix “ψ”. Repeats are marked with bold-face upper case letters (RA – RI) in regions where they are located. The two red arcs indicate the duplicated rRNA gene clusters.

The mitochondrial genome of *Huperzia squarrosa* is assembled as a single circular molecule (Fig. 1, deposited in GenBank under the accession JQ002659). Its size is 413,530 base pairs (bp), with AT content of 55.8%. The genes account for 27% of the genome, 10% and 17% of which are exons and introns respectively (Table 1).

**Table 1:** Genome sizes and proportions of the various types of sequence in the mitochondrial genomes of *Chara vulgaris* and seven land plants^1^.

Species	Genome size (bp)	AT (%)	Genes (%)	Exons (%)	Introns (%)	Intergenic spacers (%)
*Chara vulgaris*	67,737	59.1	91	52	39	9
*Marchantia polymorpha*	186,609	57.6	51	23	28	49
*Physcomitrella patens*	105,340	59.4	65	37	28	35
*Megaceros aenigmaticus*	184,908	54.0	50	16	34	50
*Huperzia squarrosa*	413,530	55.8	27	10	17	73
*Cycas taitungensis*	414,903	53.1	20	9	11	80
*Oryza sativa*	490,520	56.1	14	8	6	86
*Brassica napus*	221,853	54.8	29	16	13	71

1 Intron-encoded genes such as *mat*R in vascular plants were excluded from calculation of genes and exons.

From our fosmid library screening experiments, we believe that the *Huperzia* chondrome sequence reported here represents a completely sequenced mitochondrial genome of an early vascular plant. With seven bryophyte chondromes and over two dozens of seed plant chondromes sequenced (http://www.ncbi.nlm.nih.gov/genomes/GenomesGroup.cgi?taxid=33090&opt=organelle), this genome provides an important piece of data for comparison to identify the phylogenetic point at which the organellar genome experienced dramatic changes, particularly in genome size. The bryophyte chondromes are 100–200 kb in size (Table 1) [18], whereas the seed plant chondromes show a much broader size range, from slightly over 200 kb in *Brassica* [33], [34] to 11.3 mb (million base pairs) in *Silene* [35]. The over 400 kb mitochondrial genome in the lycophyte *Huperzia* is approximately twice the size of the largest bryophyte chondrome, that of the hornwort *Phaeoceros laevis* [23]. This size increase is mostly caused by expansion of intergenic spacers, whose percentage in the whole genome jumps from 35–50% in bryophytes to 73% in the lycophyte (Table 1). This expansion does not seem to be caused by transposons, as the percentages of transposon fragment sequences in the genomes have remained largely unchanged from bryophytes to the lycophyte based on a preliminary analysis (data not shown). Instead, insertion of a large number of pseudogene pieces has partially resulted in expansion of the spacers. Sixty-nine pseudogene pieces longer than 50 bp were detected. They added to 18,026 bp and account for 4.4% of the genome (Table S1). Previously, a moderate number of pseudogene pieces were found in intergenic spacers in the chondromes of the three liverworts, but only a few in the two hornworts and none in the two mosses, likely due to presence/absence of reverse transcriptase and different constraints on genome sizes in different species [18]. In the *Marchantia* chondrome, which has the most pseudogene pieces among bryophyte chondromes, their total length was only 5,402 bp, accounting for 2.9% of the genome [18]. During the same evolutionary transition, contribution to the genome size by exons and introns decreases significantly, from 50–65% to 27%, and the proportions of decrease for exons and introns are similar (Table 1).

RNA editing likely occurs in the *Huperzia* mitochondrial genome, as annotation of all protein-coding genes using the standard genetic code requires introduction of 19 editing events to reconstitute start or stop codons and to remove internal stop codons (Table 2). The *in silico* analyses with the software PREPACT [36] using sequences of genes from *Marchantia* or cDNAs from *Isoetes* and *Selaginella* as reference templates suggest that there are 334, 576, and 364 edited sites respectively (Table 3). Despite some uncertainty associated with these analyses, it is reasonable to say that the level of editing in the *Huperzia* chondrome is less than what have been reported in the chondromes of two other lycophytes, *Isoetes* and *Selaginella*, where 1,782 and 2,152 editing events are required to make entire transcript populations functional [25], [37].

**Table 2:** A list of start and stop codons created or removed through putative RNA editing events in coding sequences within *Huperzia squarrosa* mitochondrial genome^1^.

Gene name	Start codon created	Stop codon removed	Stop codon created
*sdh4*	ACG→AUG		CAA→UAA
*atp9*			CAA→UAA
*rps13*	ACG→AUG		CGA→UGA
*nad9*	ACG→AUG		CAA→UAA
*nad1*	ACG→AUG	UAA→CAA	CGA→UGA
*cox1*	ACG→AUG		
*nad5*	ACG→AUG	UAA→CAA	CAA→UAA
*rps12*		UAA→CAA	
*rps2*			CAA→UAA
*rpl6*			CAA→UAA
*cob*		UAG→CAG	
*cox3*			CAA→UAA

1 GTG is the start codon for *rpl16*.

**Table 3:** Predicted RNA editing sites in the mitochondrial genome of *Huperzia squarrosa* using sequences of genes from *Marchantia* or cDNAs from *Isoetes* and *Selaginella* with the software PREPACT.

gene	*Marchantia polymorpha*			*Isoetes engelmannii*			*Selaginella moellendorffii*		
	C>U	U>C	total	C>U	U>C	total	C>U	U>C	total
atp1	3	0	3	8	26	34	6	5	11
atp4	7	1	8	6	5	11			
atp6	9	0	9	14	19	33			
atp8	5	2	7	4	6	10	4	7	11
atp9	7	0	7	8	0	8			
cob	8	3	11	10	6	16			
cox1	19	0	19	30	38	68			
cox2	4	0	4	2	12	14	12	12	24
cox3	6	0	6	13	19	32	13	12	25
nad1	16	4	20	19	5	24	14	15	39
nad2	16	0	16	23	29	52	18	26	44
nad3	12	0	12	10	4	14	7	5	12
nad4	21	2	23	23	3	26	17	25	42
nad4L	3	0	3	3	2	5	4	5	9
nad5	15	7	22	41	42	83	26	40	66
nad6	10	1	11	13	7	20	5	14	19
nad9	4	1	5	8	14	22	6	10	16
rpl2	15	24	39						
rpl5	6	0	6	4	8	12			
rpl6	4	0	4						
rpl10	4	14	18						
rpl16	2	0	2						
rps2	3	1	4	5	9	14			
rps3	5	7	12	16	12	28			
rps4	5	2	7	5	4	9			
rps10	1	2	3						
rps11	4	2	6						
rps12	1	0	1						
rps13	8	0	8						
rps14	0	0	0						
rps19	1	1	2						
sdh3	6	3	9	2	2	4			
sdh4	7	2	9						
tatC	13	5	18	12	25	37	14	32	46
**sum**	**250**	**84**	**334**	**279**	**297**	**576**	**146**	**208**	**364**

No foreign DNA of chloroplast or nuclear origin was detected in the *Huperzia* mitochondrial genome. This result is the same as what was found in the *Selaginella* chondrome [25]. In the *Isoetes* chondrome, however, three short pieces of chloroplast and nuclear DNAs were detected despite the fact that the genome seemed to be relatively compact [24].

### Gene Content

The *Huperzia* mitochondrial genome contains 66 genes, with 37 coding for proteins, 3 for ribosomal RNAs, and 26 for transfer RNAs (Fig. 1, Table S2). The 37 protein-coding genes include 8 genes for NADH:ubiquinone oxidoreductase (complex I of the respiratory chain, as designated in [1]; *nad1-6, 4L, 9*), 2 genes for succinate:ubiquinone oxidoreductase (complex II; *sdh3, 4*), 1 gene for ubiquinol:cytochrome *c* oxidoreductase (complex III; *cob*), 3 genes for cytochrome *c* oxidase (complex IV; *cox1-3*), 5 genes for adenosine triphosphate synthase (complex V; *atp1, 4, 6, 8, 9*), 1 gene for cytochrome *c* biogenesis (*ccmFC*), 16 genes for ribosomal proteins, and 1 gene for other functions (*tatC*).

There is a duplicated set of rRNA genes. Several tRNA genes also have duplicated copies, some up to 3 copies (Table S2). There is no chloroplast-originated tRNA gene in the *Huperzia* chondrome.

Among the total of 80 genes (66 unique ones plus 14 duplicated copies), six are pseudogenes. One of them is *ccmFC*, which is the only remaining member of the gene complex coding for cytochrome *c* biogenesis function. The pseudogene argument is supported by two lines of evidence: about 500 nucleotides are missing in the first exon and there are several indels that disrupt the reading frame (Fig. S1). One interesting aspect about this gene is that it is split into two pieces located on two different strands far apart in the genome, with 80 nucleotides of well conserved sequence of the 3′-end of the intron attached to the second exon (Figs. 1 and S1). The gene *nad7*, present in other two lycophytes (*Isoetes* [24] and *Selaginella* [25]) and other vascular plants, but absent or present as a pseudogene in some bryophytes, is absent in the *Huperzia* chondrome (Table S2). The repeated efforts to find this gene in the fosmid library screening experiments did not yield any positive clone. For ribosomal protein genes, many of which have been lost from the completely sequenced mitochondrial genomes of two hornworts [22], [23] and apparently also from the chondromes of *Isoetes* and *Selaginella* [24], [25], there are still 16 genes in the *Huperzia* mitochondrial genome and 14 of them are functional. In land plants, only liverworts have more ribosomal protein genes in their chondromes [18]. Likewise, the *Huperzia* mitochondrial genome is among the most tRNA gene-rich land plant chondromes, and this condition is in stark contrast to the other two lycophytes, which seem to have lost most or all tRNA genes from their chondromes [24], [25].

Three recent studies reported pseudogene pieces in intergenic spacers [18], [38], [39]. One of them performed a systematic survey of pseudogene pieces in all seven sequenced bryophyte mitochondrial genomes and found that the three liverworts had a few dozens of such fragments in the spacers, whereas the two hornworts had only a few pieces and the two mosses had none [18]. In the *Huperzia* chondrome, 69 pseudogene pieces were found in 32 spacers (Table S1), and they were derived from 24 of the 40 protein- and rRNA-coding genes. For all genes encoding functions involved in respiration (excluding the dysfunctional *ccmFC*), only *nad2* and *sdh4* had no pseudogene piece in the spacers. In addition, *tatC* and *rrn18* lacked any piece in the spacers. In contrast, only six of the 14 functional ribosomal protein genes had pseudogenes, *rpl2*, *rps2*, *rps3*, *rps4*, *rps10*, and *rps12*. Pseudogenes were also detected for seven tRNA genes: *trnFgaa*, *trnKuuu*, *trnLuaa*, *trnMfcau*, *trnPugg*, *trnWcaa*, and *trnYgua*. Because tRNA genes in general are very short and show extreme sequence conservation, they could not be subject to the same kind of analyses as were done to the protein- and rRNA-coding genes for investigation of the sources and mechanisms of origins of the pseudogenes. Hence, they will not be discussed any further.

One question to ask is where these pseudogene pieces came from. We examined alignment of the functional copy and pseudogene pieces of the gene as well as its functional ortholog from other sequenced plant mitochondrial genomes (Fig. S2). In addition, we performed phylogenetic analysis using the alignment (phylogenetic trees not shown). Among the 24 genes of this kind, 17 genes have their pseudogene pieces grouped with the functional copy from the *Huperzia* chondrome. For the other seven genes (*atp8*, *cob*, *nad4L*, *nad5*, *rps2*, *rps3*, and *rps12*), one or a few pseudogene pieces were either short or somewhat divergent, and thus grouped with the functional ortholog from other species. Finally, the same kind of analyses were performed for five fragments of three group II introns in such pseudogene pieces: *cox2i691*, *cox3i171*, and *rps10i235* (Fig. S3), and the results showed that all intron fragments in the pseudogene pieces were more closely related to the introns in the functional genes of the *Huperzia* chondrome than to those from other plant chondromes. Therefore, these data suggest that the most pseudogene pieces came from their corresponding functional genes in the *Huperzia* chondrome. For those that did not group with the functional copy from the *Huperzia* chondrome, one explanation may be that they have accumulated aberrant mutations after pseudogenization, and our examination of the alignment seems to support such an interpretation. The intron *cox3i171* provides extra information to support that the pseudogene pieces in the *Huperzia* chondrome originated within the genome, not from outside, because this intron has only been found in liverworts and Lycopodiaceae so far [18], [19], [20], [40], and three copies of this intron from the *Huperzia* pseudogenes are all much more similar to the intron in the functional copy of the *Huperzia* chondrome than those from the three liverworts (Fig. S3).

A further question to ask is how these pseudogene pieces arose. One possible mechanism is retroposition: reverse transcription of the gene transcript and insertion of the cDNA back into the genome. A piece of evidence supporting this scenario is that several intron-containing genes have intron-less fragments in the spacers (Table S1, Fig. S4). However, some pseudogene pieces contain intron fragments. This situation can be explained by the use of intron-containing pre-mRNAs as templates for reverse transcription or by other mechanisms of sequence duplication that do not involve RNA intermediates. Our examination of alignment between the functional gene and pseudogene piece(s) in all cases (Fig. S4) showed that a majority of the pseudogene pieces that lack introns were resulted from cDNAs with precise splicing removal of introns and connection of exons. In cases where introns were still present, regions of the exon/intron juncture were well aligned; the introns also aligned well between the functional gene and the pseudogene piece(s) (Figs. S3 & S4). Ideally, RNA-edited sites can also be compared between the pseudogene pieces and the functional gene to test whether reverse transcription was involved, but lack of cDNA sequences data prevents this analysis from being done. The results of *in silico* analyses of RNA editing are just not accurate enough to permit such secondary analysis. Finally, we emphasize that despite the relatively strong evidence uncovered in this study that supports a retroposition mechanism for the origin of the pseudogene pieces in intergenic spacers, other mechanisms cannot be completely excluded, particularly for those pieces that did not group with the functional copy of the same species.

Regardless of mechanisms responsible for origins of these pseudogene pieces, their presence in such abundance from so many genes in the *Huperzia* mitochondrial genome poses an interesting question on why they exist. Recently, it has been reported that thousands of or even more pseudogenes are present in sequenced nuclear genomes of plants and animals and that retroposition seems to be the mechanism of their origin [41], [42], [43], [44]. Some of these pseudogenes produce antisense small RNAs with features similar to small interfering RNAs [43]. It will be desirable to investigate whether pseudogenes in plant mitochondrial genomes have similar functions.

### Gene Order and Repeat Sequences

**Figure 2:**
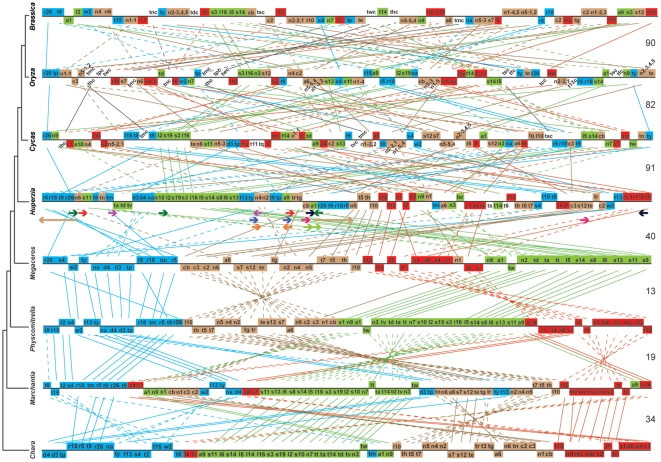
Gene order comparison among mitochondrial genomes of *Chara vulgaris*, *Marchantia polymorpha*, *Physcomitrella patens*, *Megaceros aenigmaticus*, *Huperzia squarrosa*, *Cycas taitungensis*, *Oryza sativa*, and *Brassica napus*. Species are arranged according to the organismal phylogeny of land plants and the outgroup [29]. Solid lines connect orthologous genes between species with the same orientation, and dashed lines connect those with the reversed orientation. Repeat sequences (shown in colored arrows) in *Huperzia* are color-coded: RepA – red, RepB – purple, RepC – blue, RepD – black, RepE – light green, RepF – green, RepG – orange, RepH – brown, and RepI – pink. The inferred number of events of deletion, duplication, inversion, and translocation required to bring the two adjacent chondromes into complete synteny is shown on the right between the two genomes.

The gene order in the *Huperzia* mitochondrial genome can be described as half bryophyte-like and half seed plant-like. This genome exhibits the most dramatic rearrangement since the origin of land plants; 40 events of deletion, duplication, inversion, and translocation are required to bring the chondromes of *Huperzia* and *Megaceros* into complete synteny (Fig. 2). The level of rearrangement during the origin of vascular plants surpasses what the mitochondrial genome experienced when plants colonized land (34 events). Ten gene clusters conserved in the *Chara* and bryophyte chondromes are present in this early vascular plant chondrome: s10-l2-s19-s3-l16-l5-s14-s8-l6-s13, r5-r18-t9-r26, tv-td-ta, d3-d4, t13-ty, n2-n4, tr-tg, t7-t5-th, a4-c1, and te-s12 (see Table S2 for abbreviated and full gene names). The ribosomal protein gene cluster, a putative polycistronic operon that can be traced back to the mitochondrial genome of *Reclinomonas americana*, an early eukaryote [5], is still intact and comprises 10 genes in *Huperzia*. It is also interesting to note that the gene cluster of n5-t5-th-l10-t10-my-tf-s1-s2, formed through juxtaposition by parts of two gene clusters likely in the common ancestor of hornworts and vascular plants, survived genome shuffling during the bryophyte-vascular plant transition (Fig. 2). On the other hand, several blocks of genes in the chondromes of charophytes and bryophytes no longer stay together in the *Huperzia* chondrome, e.g., (n6)-c2-c3-(n1)-cb, n2-n4-n5, c1-a4-(a8-s1). In Figure 2, most genes shown in blue, brown, and red color and even some genes in green, which largely stayed together in the *Chara* and bryophyte chondromes, are dispersed all over the genome in *Huperzia*.

Nine classes of repeat sequences longer than 100 bp were detected in the *Huperzia* chondrome (Table 4). All of them have two copies except one, RepF, which has three copies. Some of them are direct repeats whereas others are inversely oriented. Six of the repeat classes (RepB, C, D, E, F and I) show homology to genes or introns in the genome, and three of them (RepB, C, and I) in fact involve introns as the repeats *per se*. These sequence homologies suggest that the repeats arose from duplication of pre-existing sequences within the genome, perhaps mediated initially by transposons. A preliminary examination of transposon fragment distribution shows that most of the nine repeat classes have such fragments located within 2 kb on at least one side (data not shown).

**Table 4:** Repeat sequences in the mitochondrial genome of *Huperzia squarrosa* 1.

Name	Location	Length (bp)	Orientation	Origin of the repeat
RepA_Hs	*atp9-trnRacg*	124	direct	*–*
	*nad6-rps11*			
RepB_Hs	*trnMcau-trnAugc*	1,241	inverted	*nad4i461g2*
	part *nad4i461g2*			
RepC_Hs	part *atp9i87g2*	681	direct	*atp9i87g2*
	*trnYgua-nad4*			
RepD_Hs	*cob-atp1*	362	inverted	*rrn26*
	*trnQuug-atp8*			
RepE_Hs	*cob-atp1*	143	direct	*atp8*
	*cob-atp1*			
RepF_Hs	*rrn26-nad6*	168	direct	*cox3i171g2*
	*rps10-rpl2*			
	*atp1-rrn26*		(inverted)	
RepG_Hs	*atp9-trnRacg*	100	inverted	*–*
	*trnYgua-nad4*			
RepH_Hs^2^	*atp1-nad5*	14,637	inverted	*rrn26, trnMfcau,*
	*atp8-nad6*			*rrn18, rrn5*
RepI_Hs	part *cobi693g2*	764	direct	*cobi693g2*
	*rps12-trnEuuc*			

1 Only identical repeats are listed, except for RepH_Hs, which has one indel and one substitution, and RepI_Hs, which has two indels and one substitution between the two copies.

2 RepH_Hs includes the complete genes of *rrn5*, *rrn18*, *rrn26*, and *trnMfcau* and part of *nad5* gene.

Fifteen microsatellite sequences of di-, tri-, and tetra-nucleotides were found in the *Huperzia* chondrome, with the tri-nucleotide type being the most abundant (9 sequences), the di-nucleotide type less so (4 sequences), and the tetra-nucleotide type the least so (2 sequences). None of them was located in any of the repeat sequences identified above, in a stark contrast to what was found in the *Selaginella* chondrome, where a much larger number of microsatellites were detected and 82 out of the total of 98 microsatellites occurred in five repeats [25].

A model was proposed more than twenty years ago on how repeat sequences were responsible for plant mitochondrial genome rearrangement and large repeats were generated via short direct repeats-mediated recombination [45], [46]. This model has recently been substantiated by data from the completely sequenced cucumber chondrome [47]. It also seems to explain the distribution of repeats and genes that have changed locations in the *Huperzia* chondrome (relative to the bryophyte chondromes). First, three classes of repeats were involved in disruption of some gene clusters in bryophytes and resulting in the current gene distribution pattern in the *Huperzia*: RepA for *rps11-atp9* (which was linked in *Chara*, *Physcomitrella*, and *Megaceros*); RepD for *cob-trnQuug*(t10) (linked in *Chara* and *Chaetosphaeridium* (NC_004118)); RepG for *nad4-nad2-trnGgcc*(tg)-*trnRacg*(tr) (linked in *Physcomitrella* and *Megaceros* (tr is lost in the latter)); RepG for *trnGgcc*(tg)-*trnRacg*(tr)-trnRucu(t13)-trnYgua(ty) (linked in *Marchantia*) (Figs. 1 and 2). Second, two of the three copies of RepF are located near the two copies of RepH, which is the sole long (14 kb) repeat class in the *Huperzia* chondrome. Third, the RepB, C, and I are all duplicated intron portions and rearrangement involving them would disrupt genes. Given that there is lack of *trans*-splicing capability in the genome (no *trans*-splicing intron (see below)), it is understandable that these three repeat classes were not involved in genome rearrangement. Finally, for RepE, which has both copies located in the same long spacer between *atp1* and *cob*, any rearrangement facilitated by them would not be detected.

One unexplained observation is that all repeats except one copy of RepI are located in half of the genome (Fig. 1). Nevertheless, in both halves of the genome that contain or lack repeats, there are regions that show many rearrangements or gene order conservation (Fig. 2). Thus, there are probably many repeats under 100 bp that escaped detection because of the search criterion of 100 bp.

Finally, we want to add that in the process of isolating mitochondrial DNA fragments for sequencing and assembling the genome, we did not detect existence of multipartite subgenomic circles as found in some angiosperms [47], [48].

### Intron Content

The *Huperzia* mitochondrial genome contains 32 group II introns and no group I intron, according to the definitions of these mobile genetic elements [49]. They are located in 15 genes: *atp6*, *atp9*, *cob*, *cox1*, *cox2*, *cox3*, *nad1*, *nad2*, *nad3*, *nad4*, *nad5*, *rpl2*, *rps3*, *rps10*, and *rps14*. All of them are *cis*-spliced (Table S3). The intron complement in the *Huperzia* chondrome is a mixed result of intron gains and losses at different stages of land plant evolution.

While intron content has been shown to be highly stable in the mitochondrial genomes of each of the three bryophyte lineages [18], it is not in the chondromes of lycophytes. Among a total of 48 intron positions in the chondromes of three lycophytes (*Huperzia*, *Isoetes*, and *Selaginella*), 32 positions show variability in intron content: *atp6i80g2*, *atp9i95g2*, *cox1i227g2*, *cox1i266g2*, *cox1i323g2*, *cox1i395g1*, *cox1i511g2*, *cox1i876g1*, *cox1i1149g2*, *cox1i1305g1*, *cox2i373g2*, *cox2i691g2*, *cox3i171g2*, *nad1i477g2*, *nad1i669g288*, *nad1i728g2*, *nad2i542g2*, *nad2i709g2*, *nad4i976g2*, *nad4i1399g2*, *nad5i392g2*, *nad7i140g2*, *nad7i209g2*, *nad7i676g2*, *nad7i917g2*, *nad7i1113g2*, *rpl2i917g2*, *rps3i74g2*, *rps3i257g2*, *rps10i235g2*, *rps14i114g2*, and *rrn18i839g1* (Table S3). This level of intron distribution variation within a major lineage is unprecedented in land plants. It may be partly due to the fact that two of the three lycophytes, *Isoetes* [24] and *Selaginella* [25], have extremely unusual mitochondrial genomes while *Huperzia* has a rather conventional plant chondrome.

### The Most Archaic Mitochondrial Genome of Vascular Plants in *Huperzia*

Lycophytes are the sister lineage to all other vascular plants [28], [29], and hence are likely to capture many ancestral features of vascular plants. The *Huperzia* chondrome represents the most archaic form of vascular plant mitochondrial genomes when compared with those of other vascular plants and the outgroup bryophytes. Its ancestral nature is primarily reflected in the gene order. Among more than two dozens of vascular plant chondromes sequenced to date (http://www.ncbi.nlm.nih.gov/genomes/GenomesGroup.cgi?taxid=33090&opt=organelle), the *Huperzia* mitochondrial genome has the least rearranged gene order relative to the seven bryophyte chondromes [18], [19], [20], [21], [22], [23] (Fig. 2). First, it has a large conserved gene cluster (containing 10 genes) that has been well conserved since the origin of mitochondria – the ribosomal protein gene cluster [5]. In contrast, this gene cluster is broken into much smaller ones containing no more than four genes in the chondromes of seed plants and two other lycophytes, *Isoetes* and *Selaginella* [24], [25] (Fig. 2). Second, the low level of genome rearrangement in the *Huperzia* mitochondrial genome is reflected by the fact that all of its 32 introns are *cis*-spliced. In the highly rearranged chondromes of seed plants, several group II introns in *nad1*, *nad2*, and *nad5* are *trans*-spliced [50] (Table S3), and one of them, *nad1i728g2*, has undergone *cis*- to *trans*-splicing transition many times independently [51], [52]. Not surprisingly, the highly rearranged chondromes of *Isoetes* and *Selaginella* contain their own sets of *trans*-splicing introns, and in fact a first ever *trans*-splicing group I intron has been discovered in *Isoetes* [24], [25] (Table S3). A third indicator of the archaic gene order in the *Huperzia* chondrome is that only 40 events of deletion, duplication, inversion, and translocation are required to bring this genome and that of *Megaceros* into complete synteny, whereas more than twice as many events are required to bring the chondromes of *Huperzia*, *Cycas*, *Oryza*, and *Brassica* into complete synteny (Fig. 2). It should be added that this indicator does not reflect accurately the level of genomic rearrangement that happened during evolution because of the following two facts. One is that evolutionary gaps between *Huperzia* and *Cycas*, between *Cycas* and *Oryza*, and between *Oryza* and *Brassica* are smaller than that between *Megaceros* and *Huperzia* [53]. The other is that in seed plants the number of events inferred to bring two chondromes into complete synteny is almost certainly underestimated because these genomes are so recombinogenic that the rate is likely saturated. For example, two cytotypes of one maize species differ by as many as 16 rearrangement events [54].

Presence of the large conserved ribosomal protein gene cluster and several small gene clusters in the *Huperzia* chondrome suggests that this genome still uses an ancestral type of gene expression system, presumably with a relatively small number of promoter sequences in the genome. In contrast, the mitochondrial genomes of seed plants probably have a derived type of gene expression system, with one or multiple promoters for each of their most genes, because of the high frequency of genome rearrangement among species and the genome structure of having mostly free-standing genes (or gene pieces in cases of *trans*-splicing intron-connected exons) [8], [9], [10], [11], [12].

Several other aspects of the *Huperzia* chondrome reinforces its archaic status among all vascular plant mitochondrial genomes. One is its gene content, with nearly the full set of genes found in the chondromes of *Chara*, *Marchantia*, and *Physcomitrella* still present in this genome. The only major categories of genes that are missing or have become pseudogenes are *ccm* genes and *nad7*. Ribosomal protein genes and tRNA genes, which have been lost in *Isoetes*, *Selaginella* and some angiosperms [24], [25], [55], are almost all present in *Huperzia*. Second, the level of RNA editing is quite low in the *Huperzia* mitochondrial genome when compared with that in the *Isoetes* and *Selaginella* chondromes [25], [37], but is comparable with the editing levels in several angiosperm mitochondrial genomes [34], [56], [57], [58]. Third, there is lack of foreign DNAs of chloroplast or nuclear origin in the *Huperzia* chondrome, unlike what was observed in *Isoetes*, *Cycas*, and some angiosperms, where chloroplast tRNA genes and other fragments, or nuclear DNAs have invaded the mitochondrial genome, sometimes on a massive scale [13], [24], [47], [56], [59]. Finally, even though the *Huperzia* chondrome is 2–4 times the sizes of bryophyte chondromes, it is in no position to compete with some monstrous angiosperm mitochondrial genomes [35], [47], [56], [59]. The genome size increase in the *Huperzia* mitochondria seems to be related to the overall tolerance of large genomes in cells of vascular plants when the diploid phase becomes dominant in the life cycle of a plant [60], [61]. It is perhaps caused mostly by retroposition of pseudogenes into intergenic spacers, not as a result of massive invasion of foreign DNAs from the chloroplast and nucleus as seen in some angiosperm chondromes [47], [56], [59].

## Materials and Methods

Approximately 10 g of fresh tissue of *Huperzia squarrosa* (G. Forster) Trevis was collected in Matthaei Botanical Gardens at the University of Michigan. The material was brought to the lab for cleaning under a dissecting scope. A voucher specimen numbered Qiu 05001 was deposited at the University Herbarium.

Total cellular DNA was extracted with the CTAB method [62], and purified with phenol extraction to remove proteins. A fosmid library was constructed using the CopyControl™ kit (EPICENTRE Biotechnologies, Madison, Wisconsin, USA) from the total cellular DNA fragments of 35–45 kb size-selected by agarose gel electrophoresis. No restriction enzyme digestion or mechanical shearing was used before electrophoresis. Clones containing mitochondrial DNA fragments were identified through Southern hybridizations using the HRP chemiluminescent blotting kit (KPL, Inc., Gaithersburg, Maryland, USA), with major mitochondrial genes as probes. The probes were made by amplification from total cellular DNAs of *Marchantia polymorpha* and *Arabidopsis thaliana*.

The inserts were sequenced with two methods. First, fosmid DNA was sheared into 2–3 kb segments and then the DNA segments were purified by agarose gel and cloned in pUC-18 vector for shotgun-sequencing library construction. Thermo-cycling sequencing reaction was performed in a final volume of 24 µL containing 16-µL DYEnamic ET Terminator sequencing kit premix, 10 pM universal sequencing primers, and 500 ng plasmid DNA. The reaction conditions were 95°C for 2 min, followed by 35 cycles of 95°C denaturation for 15 s, 50°C annealing for 15 s, and 60°C extension for 90 s. The amplified DNA fragments were sequenced on an ABI-3730 DNA sequencer (Applied Biosystems, Foster City, California, USA). DNA sequences were assembled by using the software package phred/phrap/consed/ [63], [64] on a PC/UNIX platform. Approximately 270 kb was obtained with this method. Second, more inserts, which connected the entire genome circle, were sequenced using primer-walking on an ABI 3100 genetic analyzer (Applied Biosystems, Foster City, California, USA). Sequences were assembled using Sequencher (Gene Codes Corp., Ann Arbor, Michigan, USA).

The mitochondrial genomes were annotated in seven steps. First, genes for known mitochondrial proteins and rRNAs were identified by Basic Local Alignment Search Tool (BLAST) searches [65] (http://www.ncbi.nlm.nih.gov/blast/Blast.cgi) of the non-redundant database at the National Center for Biotechnology Information (NCBI). The exact gene and exon/intron boundaries were predicted by alignment of orthologous genes from annotated plant mitochondrial genomes available at the organelle genomic biology website at NCBI (http://www.ncbi.nlm.nih.gov/genomes/ORGANELLES/organelles.html). Occurrence of RNA editing was inferred through creation of proper start and stop codons as well as removal of internal stop codons. Further, RNA editing sites were predicted by *in silico* analyses using the recently developed software PREPACT (www.prepact.de) and following the default settings [36]. Sequences of mitochondrial genes from *Marchantia* [19] or cDNAs from *Isoetes* (GenBank accessions HQ616410–HQ616434) [37] and *Selaginella* (GenBank accessions JF276233–JF276250) [25] were used as reference templates in three separate analyses to minimize the effect of sequence divergence among species. The *Marchantia* gene sequences could be used for such analyses because no RNA editing has been detected in this chondrome. Second, genes for hypothetical proteins were identified using the web-based tool - Open Reading Frames Finder (ORF-finder; http://www.ncbi.nlm.nih.gov/gorf/gorf.html) with the standard genetic code. Third, genes for tRNAs were found using tRNAscan-SE [66] (http://lowelab.ucsc.edu/tRNAscan-SE/). Fourth, repeated sequences were searched using REPuter [67] (http://bibiserv.techfak.uni-bielefeld.de/reputer/) or BLAST. Fifth, microsatellite sequences were screened using msatcommander 0.8.2 with the following settings: accepting di-nucleotide (di-) repeats of six or more, and tri-, tetra-, penta- and hexa-nucleotide repeats of four or more [68] (five was used for all five categories in the *Selaginella* study [25]). Finally, pseudogene pieces in intergenic spacers were identified by BLAST genes against spacers, and those longer than 50 bp were recorded in this study.

To detect DNAs of chloroplast and nuclear origin in the *Huperzia* mitochondrial genome, we compared the *Huperzia* chondrome with the chloroplast genome of *Huperzia lucidula* [69] and the nuclear genome of *Selaginella moellendorffii* [70] using the program blastn at NCBI. In both analyses, default settings were used.

The annotated GenBank file of the *Huperzia* mitochondrial genome was used to draw a gene map by using OrganellarGenomeDRAW tool (OGDRAW) [71]. The map was then examined for further comparison of gene order and content. When sequence homology in some parts of certain genes or intergenic spacers was uncertain, the sequences were aligned using CLUSTAL_X [72], with visual examination followed.

## Supporting Information

Figure S1 **Alignment of** ***ccmFC*** **Sequences from** ***Physcomitrella*** **,** ***Cycas*** **, and** ***Huperzia*** **.**
(DOCX)

Figure S2 **Alignment of 24 genes and their pseudogene piece(s) from the** ***Huperzia*** **mitochondrial genome and the functional ortholog from other plants.** Most of these plants have their mitochondrial genomes sequenced, which are available at NCBI Organelle Genome Resources (http://www.ncbi.nlm.nih.gov/genomes/GenomesHome.cgi?taxid=2759&hopt=html). A small number of sequences are from GenBank and have their accession numbers listed after the taxon names. Coordinate numbers indicating location of a pseudogene piece within the *Huperzia* mitochondrial genome are listed in the sequence name. If desired, each matrix can be copied in “word” to make a “.txt” file and opened in PAUP to run a phylogenetic analysis to determine evolutionary relationships of the pseudogene pieces.
(DOCX)

Figure S3 **Alignment of introns that are attached to pseudogene piece(s) or located in the functional gene in the** ***Huperzia*** **mitochondrial genome and the ortholog intron from other plants.** Most of these plants have their mitochondrial genomes sequenced, which are available at NCBI Organelle Genome Resources (http://www.ncbi.nlm.nih.gov/genomes/GenomesHome.cgi?taxid=2759&hopt=html). Coordinate numbers indicating location of a pseudogene intron piece within the *Huperzia* mitochondrial genome are listed in the sequence name. If desired, each matrix can be copied in “word” to make a “.txt” file and opened in PAUP to run a phylogenetic analysis to determine evolutionary relationships of the introns attached to the pseudogene pieces.
(DOCX)

Figure S4 **Alignment of functional genes and their pseudogene pieces in the** ***Huperzia*** **mitochondrial genome.** All queries are functional genes whereas subjects are pseudogene pieces.
(DOCX)

Table S1 **Pseudogene pieces in intergenic spacers of** ***Huperzia squarrosa*** **mitochondrial genome.**
(DOC)

Table S2 **Gene contents in mitochondrial genomes of selected charophyte and land plants.**
(DOC)

Table S3 **Intron contents in mitochondrial genomes of selected charophyte and land plants.**
(DOC)
