# Supplementary material for: The Mitochondrial Genome of the Lycophyte Huperzia squarrosa: The Most Archaic Form in Vascular Plants
Source: PLoS One. 2012 Apr 12;7(4):e35168. doi: 10.1371/journal.pone.0035168 (PMC3325193; doi:10.1371/journal.pone.0035168)
Supplement: Figure S1 — Alignment of ccmFC Sequences from Physcomitrella , Cycas , and Huperzia . (DOCX) [file pone.0035168.s001.docx]

**Alignment of *ccmFC* exon 1 of *Physcomitrella, Cycas*, and *Huperzia* (*Huperzia* has only the first ~325 bp.)**

[ 10 20 30 40 50 60 70 80 90 100]

[ . . . . . . . . . .]

Cycas_ccmFC_E1 ATGGTCCAAC CACAGAACTT CTTCTTTTTC ATTACTTTCA TGGTCGTGCC TCGTGGCAC- --GGCAGCAC CCGTACTATT GAAATGGTTC GTCAGTAGAG [97]

Physcomitrella .......... T......... T..T.....T C...TC..T. ....T....T .T....T..- --........ ..A....... TC.......G ..A....... [97]

Huperzia .......... T......... .C.A...A.T ....T..CT. ....T....T ........TC AT.......A G.AC-.CG.A CT.T.....G A.A.A..T.. [99]

[ 110 120 130 140 150 160 170 180 190 200]

[ . . . . . . . . . .]

Cycas_ccmFC_E1 ATGTTCCCAC AGGTGCTCCT TCTCCCAGTG GTACTCTAAT TCCTATTCCT ATATCTTCAT TACCTTTTCT AGTCCATCT- --GCATTCCA G---GGAATT [191]

Physcomitrella .......... .......... .T.T.TCA.. .....A.... A......TT. .CC....T.. C.TTGC..T. ...TT..G.- --A....... .---..G... [191]

Huperzia .....T...T T.....C.T. ...T.-.A.. .....A.... A.T....TTC .CC....... ....GG.... ...T.G.A.T ATA....... .CTG..G... [198]

[ 210 220 230 240 250 260 270 280 290 300]

[ . . . . . . . . . .]

Cycas_ccmFC_E1 CATACGCTCT ATGGACGAAG CGAAAAGTAT AGTGCTGGTT GGAGCAAGCC GCCCTATCCT ATTACCAGAC ATAATT---G GGAGAAGCTC ACCCG----- [283]

Physcomitrella .......... ......A..A .AG....G.. ...TT....A A.......-- -A..C..TT. ......CA.. ......---. AA.A...... C..AA----- [280]

Huperzia .......... .........A .AG......C ....T....A A.......-- -G..C..T.C ......CA.. .A....ATT. AT.A...... ....AACCCT [295]

[ 310 320 330 340 350 360 370 380 390 400]

[ . . . . . . . . . .]

Cycas_ccmFC_E1 -GAACTGGAG CTAGAAACGC ATCATT---C TGTTTCGTTC TCGTTCTTCA T--CTTATTC TTCTCGGATT CGTGGGA--- GACTTGTCAT ATTCAGAATC [374]

Physcomitrella -A....A... ...A...T.. ..TT..TTT. .T...TA..T ..A..T.CA. .--T..T..A ..T..AA... TA.....--- .......... ...T...... [374]

Huperzia A.....CA.A ...A...... ..TT..TTTT C..CAAA.A. GAAAAAAAA. AAG.CA.ACA .GG..AC.A. T.CTACCTTC .G.C.CCT.C .AC..A.... [395]

[ 410 420 430 440 450 460 470 480 490 500]

[ . . . . . . . . . .]

Cycas_ccmFC_E1 TTTCCGCGGT GTGCTCCGTT TACTATTATT TCGTACCCTT TTCCTACCAT ATAATCATGG GCGC-GATAG GTTAGCGA-A CAAAGGGC-- ---------- [460]

Physcomitrella ....T.T... .....TT... .TT....... .T....GT.. ...T..T... C...AT..A. ....-....C ..GG..A.-. ...G.A..CT ---AGGCTTG [469]

Huperzia .AGTTTTAT. A.A.GGGCAA C..C.A...A CTT..GTAAA .CA.ATGG.. ...GAAGAA. CT..TA..TC AGA.A.A.C. TG.GTATTTC CACAATTTTT [495]

[ 510 520 530 540 550 560 570 580 590 600]

[ . . . . . . . . . .]

Cycas_ccmFC_E1 ---------- ---------- ----GTGAGC GGGCGCGGAG AAGAAAAC-G CCAAACGCTT CG---GCCTA ACGGG--AAT GAGCAACGAC GAAATGACAA [530]

Physcomitrella GAATAGAAAA AAAAAGAAAA GCGC..A... ....A.A... ......G.-. ....G..... T.TTG..... ...AA--..A A..A...A.A .....A.A.. [566]

Huperzia TTTCTGGCTT ACCCGTCTAT CAAA..TTTT .TTTCTTCT. TTC...T.A. .....TAA.. .AATG.GT.. CTTA.TC.CC AT.GG.TA.. CC.G.A..TG [595]

[ 610 620 630 640 650 660 670 680 690 700]

[ . . . . . . . . . .]

Cycas_ccmFC_E1 GATA--AGGT GCCCCGGGAG CTCGTGCC-- -----CCCCC CCACCCCCCC ATTTA----G AAAGAAGGGG CGAAGGTTTT GGGC-CT--- -GTAGCTTCC [612]

Physcomitrella ..A.GA.AA. ..ATTTTT.T T..T.T.AAA TAA-AT.AAA AATATTTTTG .....TT-T. CTGC..TTTT .A..AACC.. C...-T.CAA C.A.AAAG.. [663]

Huperzia .GG.GC.AAA AAAGA..CGA AG.ACTTGAT GGGCAG.G.T GGGGTA.GTA G....TGGC. ..GCTCA... AAT..AA.G. .TT.T..CCA TCA...CG.G [695]

[ 710 720 730 740 750 760 770 780 790 800]

[ . . . . . . . . . .]

Cycas_ccmFC_E1 CCCGTCCC-- CCCTTCGTCG GGTGGT-GCT TGTGGGGGGG GTGTGCCACC CGAGGCAGAG ATTGGGTACT ATCTTGAAGC GCGTTCTCTC GCCTTACCAA [709]

Physcomitrella AAAA.TTTGG .TT..TA.TC TC..C.T... .T.TC.CAA. C.TATT.TT. ..TCC.T..A .A.A.T.GGA ..AGATTTTT T.T.GT..G. .......... [763]

Huperzia AA.C.G.TGG .TGC.....A ....CGTAGC AC.TC.TT.. CGAGCGA..G AAG.C.GA.T .A.AATA.TA .GTAG.TGA. A..AA.GT.. T..GA..G.. [795]

[ 810 820 830 840 850 860 870 880 890 900]

[ . . . . . . . . . .]

Cycas_ccmFC_E1 CGAGTCGACT GCTGATGGCT GTTGGTCACG ACTGCTACCG AAAAGCTCCA ATGAATATGA ATATTTCACA TGGAGGAGTG TGCATCTTTA TTATGGGTGT [809]

Physcomitrella AA..------ A.......A. ........T. ...TT---.. .....T...C ....C..... .A........ .........T .......... .......... [854]

Huperzia G.GAGT.GAG AAC..A.CT. .C.TC..CGA ..GAAGTTT. CTTC....AC T.CGTCCA.. GA.C..GG.T G.C.AA.--- ---------- ---------- [872]

[ ]

[ ]

Cycas_ccmFC_E1 TATTTTGTC [818]

Physcomitrella ..------- [856]

Huperzia --------- [872]

**Alignment of *ccmFC* intron (well conserved portion highlighted in purple) & exon 2 of *Physcomitrella, Cycas*, and *Huperzia***

[ 10 20 30 40 50 60 70 80 90 100]

[ . . . . . . . . . .]

Cycas_ccmFC_In&E2 ---------- --GTGCGACC CGGCGGCTTG TGTGC--ATC TTTATAGCTT TCAGGCCCAC GCCTCCTATT TCTTCAGGGC GG-GCGGCGC GAACTCTGGT [85]

Physcomitrella ---------- --........ ....A....A ..C..--GA. CA.C.C.TG. .T.A..T... ...CA.---- ---------- ---AT....T .......... [69]

Huperzia TTGCTAGTAA TG.CTG..T. TAC.AT.... .CCATTAGAT A.A..GAAGC AG.A.GTAGT ATAG..ATAA A.A....AAT AATCT...AA ...---.AA. [97]

[ 110 120 130 140 150 160 170 180 190 200]

[ . . . . . . . . . .]

Cycas_ccmFC_In&E2 TCAATTCGGG TATTCAATCC CGCCGCTGAG ATGCTCGGTT GACTCCTGAA CCTTGATAGG AAGATG---- ---GCTTATT CCCAAATTCG TGC-ATGAGG [177]

Physcomitrella .G........ .TC.A..... .......... ......A..C .....T.... .......G.. ....C.---- ---....C.. ........A. ...-.AA... [161]

Huperzia C.G.A.AATC .C.AT.G.AG TT..ATGA.C .AT.CTTTC. .GAAT.G..T TTA.ATAGT. ..A...CCAT AAA..GCGAA ..A.C..C.. .AAG.C..A. [197]

[ 210 220 230 240 250 260 270 280 290 300]

[ . . . . . . . . . .]

Cycas_ccmFC_In&E2 GTCAGGA--- -ACTTTGGAT G----AACC- AATGTGAATG GGTGTAAGCT CCGCT---GC TCGGAAACAC CCAGTGCTGA CCGCACTGAG AGGCACTTG- [264]

Physcomitrella T..CA.G--- -....A.... .----...TT .......... A..A...... T....---.. ..AA...... .....AT... ..A....... ..--.....- [247]

Huperzia A...AT.CAA T.A..A.A.A .GAAA..TAT C..CAT.... CA.CA..AAA ..ATGAAG.. .GAC..T.T. TTTAGATCAG ..CT...AGA ..CAG..G.A [297]

[ 310 320 330 340 350 360 370 380 390 400]

[ . . . . . . . . . .]

Cycas_ccmFC_In&E2 CCGAAGG-AG TAATCACCGC AGGTAACGCC AGTTGGCGAA GTGGCGTTAA GCATCCCTAG TGGTACGAAA AGAGAGGTCG TGA----TGA TACCATCTAC [359]

Physcomitrella ..A.T..C.A G..AGG.... G........T .......... A......... ....T..... C.A....G.. .......... ...TGGA... ..T....... [347]

Huperzia T.A..AAA.. A.GC..AGAA .AACG.AA.. -.C.ATTA.G AA.ATTAG.. CTG.ATT.TT .A.C.TAG.T .CC.GAT..- C.TTTCT..C AG....A.CT [395]

[ 410 420 430 440 450 460 470 480 490 500]

[ . . . . . . . . . .]

Cycas_ccmFC_In&E2 GTCCGTACCG CTCCTCGTGG AGTAGATCCC ACATCCAACC ATTTGACCAG GGAACGGGAT AATTCCCA-- -CTAAGCTCC -AGTTCAACC GGCG----GG [451]

Physcomitrella ..T.....T. T......... ....A...T. ........AA ..C.A..... .......... ........-- -T....T... -...AA...T ...A----.. [439]

Huperzia AATAA.CTTT ..T..TCACA .AC.A.G..T ..GA..GG-- .C....GG.. .AGG.CTTTC CC..A..TGC T...CCA..T G..C.AC..A A..CCAATAA [493]

[ 510 520 530 540 550 560 570 580 590 600]

[ . . . . . . . . . .]

Cycas_ccmFC_In&E2 CCAGCCGGGC CGTGAGCGCG GTGGGAACAG GA-TCTTCCC AAAAAGCCAA ---AACCTTC GGCCCGGGCT CGCGAATGAA GGGGACGGCC CTAATGTTGG [547]

Physcomitrella .......... ...A...-TA .......... ..-.TC.... G.....A... ---....... .A-------- ---------- ---------- --------.. [498]

Huperzia T..CT.TT.. TA.T.CT.AA AAAAT...C. ..A..CGAG. C..G....GG CTC....AA. AT.TAC.ATG ..T.TG.TTC TT..TTC.G. TGCT.C..T. [593]

[ 610 620 630 640 650 660 670 680 690 700]

[ . . . . . . . . . .]

Cycas_ccmFC_In&E2 CAAAGCCAAC CACGCCCACC CCTCCAGGAT GGGTGGGGCC GGGT-TGCGG GGAAAAGCGG ACGTGGGGAC TCGGGGCGCA GCGTA--ACA ATGGCGAGGA [644]

Physcomitrella ....------ --...T...T TTG.TC.CG. TA...AA.TA AATC-.CGAA .A...G..C. ...C...... ..A....... ..A..--..- --------.. [578]

Huperzia .CGG..A..A A.AAAT.G.T .TG.A.A..A .CAGA.C..A ..TAA.AAAA C.CG...... .ACAAAAAG. .GTT...TTT A..A.CG.GG GCC.A..AA. [693]

[ 710 720 730 740 750 760 770 780 790 800]

[ . . . . . . . . . .]

Cycas_ccmFC_In&E2 ATGAGCGGAA TGGGCAGTTC ATTC-GAGCG GATCTCTCTC TGCTCTGATA GGTGTCCAGA AAAAGCCGA- ----TTGGTC AGA--CGACG ACTACTTCTT [736]

Physcomitrella ...G------ ..AAG..... G.AT-....A ..-------- ---------- -A..AA.... ....AAA..- ----..T-.T ..G--...AT G.C.TG.A-- [642]

Huperzia .CA.ATATCT .TTC..CGC. G..TA..TTT ...ACGC.G. G..G.--G.T CAA.....A. TG.....TTC GGCC...C.. C.CTT..CGA ....AG.AGG [791]

[ 810 820 830 840 850 860 870 880 890 900]

[ . . . . . . . . . .]

Cycas_ccmFC_In&E2 AGCCTT--AG CCGCCG---- -CTTACTG-C ATTATACAAA TAACGTCTAT CACATCAATA ACGCGAAGCG GTTGGGCGGA GAATAAGCTT GCTTCT-TCA [827]

Physcomitrella --------.A T.T...---- -...-----T .....TT.-- ---------- ---------- ---------- ---------- ---------- ---------- [662]

Huperzia ..AGAAGA.A A...GAAGAA C...GTG.TT T..GA.G... CCCTTCGGT. TT.T..TC.T GG..TTC.AA .C..AC.CCT A..CG.AA.C A..CGGAC.. [891]

[ 910 920 930 940 950 960 970 980 990 1000]

[ . . . . . . . . . .]

Cycas_ccmFC_In&E2 CAAGCTTATT CTATCCCCGT TCCCCTGACA GCTGCTGGGT TTTTGGCCAT CTCTCCTTCA CCTTCCCTGG TCTTCGGCCC CATGGGAAGA GCCGTATGAG [927]

Physcomitrella -----..... .--------- ---------- -------... ...CA.---- ---------- ---------. .T-----... .C..A..... .......... [703]

Huperzia ..G.TAA.GA T..AGGT.AG A..A.AATGC ATGCAG.A.G .C.CAAG.T. ..TGT.AAG. T...TGGC.. GGAGACATTA A..TAA.... ...-...... [990]

[ 1010 1020 1030 1040 1050 1060 1070 1080 1090 1100]

[ . . . . . . . . . .]

Cycas_ccmFC_In&E2 GCAAAAACTC GTCCCACGTA CGGTTCGGAG GCCGAGCCCC A--GCAGTTT TATCAATATG AGGAAGATTA ATCAAATAGG ATCTGATCAC TATTTATCTA [1025]

Physcomitrella ..CGT.---- .G.T...... .........A ...A...... T--.....GA .GC---.G.. GCTT..G... .CT------- ---------- --...G..C. [775]

Huperzia ..C.T.---- .G.T...... T........A .......... CCC.....AA .GCTG-CC.. CT.CG.C.C. GGT------- ---------- ----..A... [1064]

[ 1110 1120 1130 1140 1150 1160 1170 1180 1190 1200]

[ . . . . . . . . . .]

Cycas_ccmFC_In&E2 TTAATCCTTA TGATATTCTA GGTGCGGCTT AGGTCAACTG ACACGAAAAA GATACAGTTT ACTCA---AC GATTGCCTTT GGGTCCCGAA CTCC---ATA [1119]

Physcomitrella ---------- ---------- ---------- ---------- ....A..... ..G....... .....---.T T......... A...T.T... ..A.---... [829]

Huperzia ---------- ---------- ---------- ---------- ...-A.T.T. A......... ....CTCC.. C......... ....T..... ....TCCG.. [1123]

[ 1210 1220 1230 1240 1250 1260 1270 1280 1290 1300]

[ . . . . . . . . . .]

Cycas_ccmFC_In&E2 TGGGGAAGGA GCGTTGTTGT TTGCGAGGTT CTGATCATTC ACATGGACCC ACTTTTCATT CCATTTGTGG TAATTCGATG ATCTATAAAC CGTCCCTAAC [1219]

Physcomitrella .A..A.GA.. ..A....... .........A T......A.T .......... ..C....... .......... .....T...T ..T....... .....T...A [929]

Huperzia .A......T. A......... ..T.TCAT.C T........T ...AA..... .......... .A...C...C .....AA..T ..T.C..... .A.......A [1223]

[ 1310 1320 1330 1340 1350 1360 1370 1380 1390 1400]

[ . . . . . . . . . .]

Cycas_ccmFC_In&E2 GAACCCATTC ATGCTTGAGC ATGATGAATC ACTTCGTGCC ATAATCGACC TGTTGCCAAT C----CACTT T----TCGGC CTCATATGG- -GAATGGAAA [1309]

Physcomitrella A..T...... ..TT.G..T. .......... .......... .........T .......... TGCAG.G.C. .CGTAC.A.A ..G.A.AA.T T...AAA... [1029]

Huperzia ACC....... ......A... ....G.G... .......A-- .......... .......... .--------- -------A.. ........A- -A....A.G. [1303]

[ 1410 1420 1430 1440 1450 1460 1470 1480 1490 1500]

[ . . . . . . . . . .]

Cycas_ccmFC_In&E2 ACTGGATCAT TTTCTGCA-- --TCGTGAAA GATCGTGGTG GATAGAGAA- TCGCGAACAT AATAATTCC- -----TGGTT GACCATGTCC CCAGAAAAAA [1398]

Physcomitrella TA.AT..AT. ...T.T..AC TT.TT.CC.T .G.GACA.AT C..G.....A .......... C...G..T.C CACTT..... ...TG...T. .......... [1129]

Huperzia .G.AA..... .CAT.C..-- --.T...... .....----- --.G..T..A .T........ C...G..T.C CACTT..... ...T....T. ...T...... [1392]

[ 1510 1520 1530 1540 1550 1560 1570 1580 1590 1600]

[ . . . . . . . . . .]

Cycas_ccmFC_In&E2 GATACTTTTT CTCCATTCAG GAAACGACGA GCGCGACTGA AGTTGCTATA CATACAAATC TATTTACGGA TCCATATGCT CCGATTGGAA CTGGAAGTTT [1498]

Physcomitrella ...TT.C... T..T.A...A .....A.--- ..A.T..CA. ...G...... ....GT.... .T........ ..T....... TTA....... .......... [1226]

Huperzia ...TG..... T..A...G.A ...-...--- ..A....CA. ...GA..... .....C.... .C........ .......... TT........ .......... [1488]

[ 1610 1620 1630 1640 1650 1660 1670 1680 1690 1700]

[ . . . . . . . . . .]

Cycas_ccmFC_In&E2 CGGAACAGGC GGCTGGTATA CCACCATAAT GGAACTGCCT CTTATTTTTC ----GCATTC --GGATAGGA TTTCTGTTGG CTTCGCCGGG GGGCTTGCGT [1592]

Physcomitrella T.A....... ---....... TT........ .A....A... T.C.....CT ----.T...T --.......G ...A.TC... ....ATT... A......... [1317]

Huperzia AAA...T... ---G...... .T........ ......A..C ..CTC..... CGCC.T...T AT.......T ...G.TC... .....TT... A..------. [1579]

[ 1710 1720 1730 1740 ]

[ . . . . ]

Cycas_ccmFC_In&E2 AGTTTGTTAC GTCAGCTCCA AAAGGATAAG TTGCATTGGA ATCGA [1637]

Physcomitrella .....TC... .......GGC TTTAT...GA ...G...... ..T.. [1362]

Huperzia .....T.... .C......GC TTTT..C.GA ...G...... .GAA. [1624]
