# Supplementary material for: The Mitochondrial Genome of the Lycophyte Huperzia squarrosa: The Most Archaic Form in Vascular Plants
Source: PLoS One. 2012 Apr 12;7(4):e35168. doi: 10.1371/journal.pone.0035168 (PMC3325193; doi:10.1371/journal.pone.0035168)
Supplement: Figure S2 — Alignment of 24 genes and their pseudogene piece(s) from the Huperzia mitochondrial genome and the functional ortholog from other plants. Most of these plants have their mitochondrial genomes sequenced, which are available at NCBI Organelle Genome Resources (http://www.ncbi.nlm.nih.gov/genomes/GenomesHome.cgi?taxid=2759&hopt=html). A small number of sequences are from GenBank and have their accession numbers listed after the taxon names. Coordinate numbers indicating location of a pseudogene piece within the Huperzia mitochondrial genome are listed in the sequence name. If desired, each matrix can be copied in “word” to make a “.txt” file and opened in PAUP to run a phylogenetic analysis to determine evolutionary relationships of the pseudogene pieces. (DOCX) [file pone.0035168.s002.docx]

**atp1**

#NEXUS

[MacClade 4.05 registered to Yin-Long Qiu Lab, EEB, University of Michigan]

BEGIN DATA;

DIMENSIONS NTAX=18 NCHAR=1663;

FORMAT DATATYPE=DNA MISSING=? GAP=- MATCHCHAR=. INTERLEAVE ;

MATRIX

[ 10 20 30 40 50]

[ . . . . .]

Huperzia_atp1 ATGAACAGATTGGCAGAAATC------------GCAGAACTATCTACTTT [38]

Huperzia_atp1_238360_237880 -------------------------------------------------- [0]

Huperzia_atp1_4707_5191 --------.............------------................. [30]

Huperzia_atp1_240083_239893 ....................T---------GCG..G..G........... [41]

Huperzia_atp1_235705_235625 -------------------------------------------------- [0]

Huperzia_atp1_292893_293673 -------------------------------------------------- [0]

Huperzia_atp1_129376_129434 -------------------------------------------------- [0]

Huperzia_atp1_330104_330468 -------------------------------------------------- [0]

Isoetes_HQ616419 .....------------CGGA------------..G...T...TG..... [26]

Zea_perennis ...G.---...CT..CC..GA---------GCT..G.....CA.G...C. [38]

Arabidopsis ...G.---...AT.TCCT.GA---------GCT..G......A.G.A.C. [38]

Cycas .C...---...ATATCCTGGA---------GCT..G....CCA.....A. [38]

Phaeoceros .C.....A...T..T.G..C---------------G....C......G.. [35]

Megaceros .C.....A...T..T.G..C---------------G..G.C......G.. [35]

Physcomitrella .......A...AA.T.G..ATAAGTTGGCTGGA..............A.. [50]

Anomodon .C.....A...AA.T.G..ATAAGTTGGCTGGA..............A.. [50]

Treubia .......A......T.G.GCT---------------.....C..C..... [35]

Marchantia .......A......T.G.GCT---------------.....C..C..... [35]

[ 60 70 80 90 100]

[ . . . . .]

Huperzia_atp1 ATTAGAACGAAGAATTACCAACTTTCACACCAAATTGCCAGTGGATGAGA [88]

Huperzia_atp1_238360_237880 -------------------------------------------------- [0]

Huperzia_atp1_4707_5191 .................................................. [80]

Huperzia_atp1_240083_239893 ..............................................A... [91]

Huperzia_atp1_235705_235625 -------------------------------------------------- [0]

Huperzia_atp1_292893_293673 -------------------------------------------------- [0]

Huperzia_atp1_129376_129434 -------------------------------------------------- [0]

Huperzia_atp1_330104_330468 -------------------------------------------------- [0]

Isoetes_HQ616419 .C.G...G....G..........A.............GA........... [76]

Zea_perennis .......A.T.....G.T.......T....G..T...AA........... [88]

Arabidopsis ...C...A.TC......GG......T..G.G..T..T.A........... [88]

Cycas ..CG.G.A.......C.......A.TG......T..A.A...T....G.. [88]

Phaeoceros ........A.........T....A.T............A........... [85]

Megaceros ........A..............A.T............A........... [85]

Physcomitrella ........A............T.ACT............A........... [100]

Anomodon ........A............T.ACT............A........... [100]

Treubia ........A..............A.T............A........... [85]

Marchantia ........A..............A.T............A........... [85]

[ 110 120 130 140 150]

[ . . . . .]

Huperzia_atp1 TCGGTCGAGTGGTCTCAGTGGGAGATGGAATTGCACGTGTTTAT----GG [134]

Huperzia_atp1_238360_237880 -------------------------------------------------- [0]

Huperzia_atp1_4707_5191 .....................A.........G..........C.----.. [126]

Huperzia_atp1_240083_239893 ......C.......................AG............TTAT.. [141]

Huperzia_atp1_235705_235625 -----................A.........G..........C.----.A [41]

Huperzia_atp1_292893_293673 -------------------------------------------------- [0]

Huperzia_atp1_129376_129434 -------------------------------------------------- [0]

Huperzia_atp1_330104_330468 -------------------------------------------------- [0]

Isoetes_HQ616419 ............................................----.. [122]

Zea_perennis ...................T........G........A.....C----.. [134]

Arabidopsis ...................T........G.......AA......----.. [134]

Cycas ..............C....T..G.....G...............----.. [134]

Phaeoceros .T...........A.....T........................----.. [131]

Megaceros .............A.....T........................----.. [131]

Physcomitrella .............A.....T........................----.. [146]

Anomodon .............A.....T........................----.. [146]

Treubia .............TC....T........................----.. [131]

Marchantia .............T.....T........................----.. [131]

[ 160 170 180 190 200]

[ . . . . .]

Huperzia_atp1 ATTGAACA---AAATTCAAGCTGGGGAACTGGTTGA---ATTTGCCAGTG [178]

Huperzia_atp1_238360_237880 -------------------------------------------------- [0]

Huperzia_atp1_4707_5191 ..G....T--C.....T.-.................ATA.........CA [173]

Huperzia_atp1_240083_239893 .......TACT.........................---.........C. [188]

Huperzia_atp1_235705_235625 ...T...T---.............A.......C.-----..C......-- [81]

Huperzia_atp1_292893_293673 -------------------------------------------------- [0]

Huperzia_atp1_129376_129434 -------------------------------------------------- [0]

Huperzia_atp1_330104_330468 -------------------------------------------------- [0]

Isoetes_HQ616419 ........---GG...............T.A.....---......TG.C. [166]

Zea_perennis .......G---.G........A..A...A....G..---.........C. [178]

Arabidopsis .......G---.G...............A.....CT---T.......AC. [178]

Cycas ..CA...G---.G...............A.......---...C.....C. [178]

Phaeoceros ........---.G...T.G..A......A....C..---.........C. [175]

Megaceros ..C.....---.G...T.G..A......A....C..---.........C. [175]

Physcomitrella ...A....---.G...........A...A.......---.........C. [190]

Anomodon ...A....---.G...........A...A.......---.........CA [190]

Treubia ........---.G........A......A.......---.........C. [175]

Marchantia ........---.G........A......A.......---.........C. [175]

[ 210 220 230 240 250]

[ . . . . .]

Huperzia_atp1 GTG-----------------------------------TTAAAGGAATGG [193]

Huperzia_atp1_238360_237880 -------------------------------------------------- [0]

Huperzia_atp1_4707_5191 .C.GTGTGAAAGGTTCGAAGATCTTTGTTGGCGAAAAA.G.GCC...... [223]

Huperzia_atp1_240083_239893 ...----------------------------------------------- [191]

Huperzia_atp1_235705_235625 -------------------------------------------------- [81]

Huperzia_atp1_292893_293673 -------------------------------------------------- [0]

Huperzia_atp1_129376_129434 -------------------------------------------------- [0]

Huperzia_atp1_330104_330468 -------------------------------------------------- [0]

Isoetes_HQ616419 ...-----------------------------------CG.......... [181]

Zea_perennis ...-----------------------------------.G........A. [193]

Arabidopsis ...-----------------------------------.G.......... [193]

Cycas ...-----------------------------------.G........A. [193]

Phaeoceros ...-----------------------------------.G.......... [190]

Megaceros ...-----------------------------------.G.......... [190]

Physcomitrella ...-----------------------------------.G.......... [205]

Anomodon ...-----------------------------------.G.......... [205]

Treubia ...-----------------------------------.G.......... [190]

Marchantia ...-----------------------------------.A.......... [190]

[ 260 270 280 290 300]

[ . . . . .]

Huperzia_atp1 CTTTGAACCTAGAAAATG--AAAATGTAGGAATCGTGATATTTGGTAGTG [241]

Huperzia_atp1_238360_237880 -------------------------------------------------- [0]

Huperzia_atp1_4707_5191 ......CT.CGA.....TTT.............T.C........C....A [273]

Huperzia_atp1_240083_239893 -------------------------------------------------- [191]

Huperzia_atp1_235705_235625 -------------------------------------------------- [81]

Huperzia_atp1_292893_293673 ------------------------.........T..A............A [26]

Huperzia_atp1_129376_129434 -------------------------------------------------- [0]

Huperzia_atp1_330104_330468 -------------------------------------------------- [0]

Isoetes_HQ616419 .......TT.G....CC.--.C.........G.T..T...........C. [229]

Zea_perennis .C.....T..T..G....--.G........T..T..TG.C.......... [241]

Arabidopsis .C.....T..T..G....--.G.....C..G..T..TG.C......G... [241]

Cycas .G.....T.CT..G....--.G........T..T..TG...C........ [241]

Phaeoceros .......T........G.--.............T..TG............ [238]

Megaceros .......T.....G..G.--.............T..TG............ [238]

Physcomitrella .......T..........--.G...........T..T..C.......... [253]

Anomodon .......T..........--.G...........T..T............. [253]

Treubia ....A..T..........--.............T..T............. [238]

Marchantia ....A..T..........--.............T..T............. [238]

[ 310 320 330 340 350]

[ . . . . .]

Huperzia_atp1 ATACTGCCATTAAAGAAGGAGATATTGTCAAACGCACTGGATCTATTGTA [291]

Huperzia_atp1_238360_237880 -------------------------------------------------- [0]

Huperzia_atp1_4707_5191 .....C.........G....A..........G..............G..G [323]

Huperzia_atp1_240083_239893 -------------------------------------------------- [191]

Huperzia_atp1_235705_235625 -------------------------------------------------- [81]

Huperzia_atp1_292893_293673 ....................A..........G......TA......GA.G [76]

Huperzia_atp1_129376_129434 -------------------------------------------------- [0]

Huperzia_atp1_330104_330468 -------------------------------------------------- [0]

Isoetes_HQ616419 ............G...G........G.....G.....G..G.TC..C..C [279]

Zea_perennis ....C..T...............C.......G.................G [291]

Arabidopsis ....C..T..A............C.......G.................G [291]

Cycas ....C..................C.......G.................G [291]

Phaeoceros ....C..T....................A.GG.................G [288]

Megaceros ....C..T....................A.GG.................G [288]

Physcomitrella .......T..............C........G.................G [303]

Anomodon .......T..............C........G.................T [303]

Treubia ............................A..G.................G [288]

Marchantia .C..........................A..G.................G [288]

[ 360 370 380 390 400]

[ . . . . .]

Huperzia_atp1 GATGTTCCCGT---AGGAAAGGCCATGTTAGGTCGTGTAGTTGATGCGTT [338]

Huperzia_atp1_238360_237880 -------------------------------------------------- [0]

Huperzia_atp1_4707_5191 ........A..---..............C.............A....... [370]

Huperzia_atp1_240083_239893 -------------------------------------------------- [191]

Huperzia_atp1_235705_235625 -------------------------------------------------- [81]

Huperzia_atp1_292893_293673 ..A...A.T.CTGC.......A............-------.A...T... [119]

Huperzia_atp1_129376_129434 -------------------------------------------------- [0]

Huperzia_atp1_330104_330468 -------------------------------------------------- [0]

Isoetes_HQ616419 ..C.....T..---........................G........... [326]

Zea_perennis ........T.C---G.................C.....G..C..C..C.. [338]

Arabidopsis ..........C---G........T...C....G.....G..C..C...A. [338]

Cycas ........T..---G.......................G..C........ [338]

Phaeoceros ........T..---............A....................C.. [335]

Megaceros ........T..---............A....................... [335]

Physcomitrella ........T..---........G.T.............G........C.. [350]

Anomodon ........T..---..........T.............G........C.. [350]

Treubia ...........---........................G........... [335]

Marchantia ........T..---........G...............G........... [335]

[ 410 420 430 440 450]

[ . . . . .]

Huperzia_atp1 GGGAATACCTATT-GATGGAAAAGGTGCTTTAAACGCTGTAGAACGAAGA [387]

Huperzia_atp1_238360_237880 -------------------------------------------------- [0]

Huperzia_atp1_4707_5191 ....G........-------G.T..........G.A..........G... [413]

Huperzia_atp1_240083_239893 -------------------------------------------------- [191]

Huperzia_atp1_235705_235625 -------------------------------------------------- [81]

Huperzia_atp1_292893_293673 A...TCGAAG...T.................................... [169]

Huperzia_atp1_129376_129434 -------------------------------------------------- [0]

Huperzia_atp1_330104_330468 -------------------------------------------------- [0]

Isoetes_HQ616419 A...G.G......-.....G..G.......GTCG...C.AC...A..... [375]

Zea_perennis ....G........-...........G...C...G..A.CAC......... [387]

Arabidopsis ....G........-...........G...C...G..A.CAC..G.A.... [387]

Cycas ....G........-...........G.......G..A.CAC......... [387]

Phaeoceros A...G........-..............C...GG..........T..... [384]

Megaceros A...G........-..............C...GG..........T..... [384]

Physcomitrella A...G........-...................G.....C......C..G [399]

Anomodon A...G........-...................G.A...C........AG [399]

Treubia A...G....C...-...................G................ [384]

Marchantia A...G....C...-...................G................ [384]

[ 460 470 480 490 500]

[ . . . . .]

Huperzia_atp1 CGTGTTGAAGTTAAAGCTCCTGGGATTATTGCGCGTAAATCTGT------ [431]

Huperzia_atp1_238360_237880 -------------------------------------------------- [0]

Huperzia_atp1_4707_5191 .....AT...........T.............A...........CTGTGT [463]

Huperzia_atp1_240083_239893 -------------------------------------------------- [191]

Huperzia_atp1_235705_235625 -------------------------------------------------- [81]

Huperzia_atp1_292893_293673 .....A.C.C.CC--.T...A...........A...........------ [211]

Huperzia_atp1_129376_129434 -------------------------------------------------- [0]

Huperzia_atp1_330104_330468 -------------------------------------------------- [0]

Isoetes_HQ616419 .....A.....G....................A........C..------ [419]

Zea_perennis .....C.....G.....C..A..........AA...........------ [431]

Arabidopsis .....C.....G.....C.........C...AA........A..------ [431]

Cycas .....C.....G.....C.............AA...........------ [431]

Phaeoceros .....A...........C.....A........A..C.....C..------ [428]

Megaceros .....A...........C.....A........A..C.....C..------ [428]

Physcomitrella .....A..............C...........A...........------ [443]

Anomodon .....A..............C...........A...........------ [443]

Treubia .....A.....G.....C..A...........A...........------ [428]

Marchantia .....A.....G.....C..A...........A...........------ [428]

[ 510 520 530 540 550]

[ . . . . .]

Huperzia_atp1 GCACGAACCCATGCAAACAGGATTAAAAGCAGTAGATAGCCCGGTTCC-- [479]

Huperzia_atp1_238360_237880 -------------------------------------------------- [0]

Huperzia_atp1_4707_5191 ..........C...........---------------------------- [485]

Huperzia_atp1_240083_239893 -------------------------------------------------- [191]

Huperzia_atp1_235705_235625 -------------------------------------------------- [81]

Huperzia_atp1_292893_293673 ..........C...............................--.C..AA [259]

Huperzia_atp1_129376_129434 -------------------------------------------------- [0]

Huperzia_atp1_330104_330468 -------------------------------------------------- [0]

Isoetes_HQ616419 .............G....G......................T......-- [467]

Zea_perennis C........T...........C...........G.......T......-- [479]

Arabidopsis ......G..T...........G........G..........T......-- [479]

Cycas .........T...........G..G........G..............-- [479]

Phaeoceros ........T.....................G.....C....T......-- [476]

Megaceros .........A....................G..G..C....T......-- [476]

Physcomitrella .........G...............................T......-- [491]

Anomodon .........................................T......-- [491]

Treubia .........A....................T..G.......T......-- [476]

Marchantia A........A.......................G.......T......-- [476]

[ 560 570 580 590 600]

[ . . . . .]

Huperzia_atp1 TATAGGTCGTGGTCAACGAGAACTTATAATAGGAGACAGACAAACTGGAA [529]

Huperzia_atp1_238360_237880 -------------------------------------------------- [0]

Huperzia_atp1_4707_5191 -------------------------------------------------- [485]

Huperzia_atp1_240083_239893 -------------------------------------------------- [191]

Huperzia_atp1_235705_235625 -------------------------------------------------- [81]

Huperzia_atp1_292893_293673 ...................A.....GCC.........G...G.....A.. [309]

Huperzia_atp1_129376_129434 -------------------------------------------------- [0]

Huperzia_atp1_330104_330468 -------------------------------------------------- [0]

Isoetes_HQ616419 ......C...............T.A......................... [517]

Zea_perennis ......C.......................C..G................ [529]

Arabidopsis ......C..................C....C..T.G.C............ [529]

Cycas ......C................CG.....C..G...C....G....... [529]

Phaeoceros ..C..........T..T.....T........................... [526]

Megaceros .......................C.......................... [526]

Physcomitrella .................................................. [541]

Anomodon .................................................. [541]

Treubia .................................................. [526]

Marchantia .................................................. [526]

[ 610 620 630 640 650]

[ . . . . .]

Huperzia_atp1 AGACTGCTATAGCTATTGATACCATATTGAACCAAAAACGAATCAACGCG [579]

Huperzia_atp1_238360_237880 -------------------------------------------------- [0]

Huperzia_atp1_4707_5191 -------------------------------------------------- [485]

Huperzia_atp1_240083_239893 -------------------------------------------------- [191]

Huperzia_atp1_235705_235625 -------------------------------------------------- [81]

Huperzia_atp1_292893_293673 ....---................T...CA.....G....A......T..A [356]

Huperzia_atp1_129376_129434 -------------------------------------------------- [0]

Huperzia_atp1_330104_330468 -------------------------------------------------- [0]

Isoetes_HQ616419 ..........................C.......G...A.......T..A [567]

Zea_perennis .A..A..A........C.....T.....A........G.A...G...T.A [579]

Arabidopsis .A..GA....T.....C...........A........G.A...A...T.A [579]

Cycas .A.........................C......G..G.....G...T.A [579]

Phaeoceros ........G..................C....T.....GA...A.....A [576]

Megaceros ........G..................C......G...GA.........A [576]

Physcomitrella .A..A.....T.......................G..G.A.......A.A [591]

Anomodon .A........C.......................G..G.A.......A.A [591]

Treubia ....C.............................G....A...A.....A [576]

Marchantia ....C.............................G....A.........A [576]

[ 660 670 680 690 700]

[ . . . . .]

Huperzia_atp1 CAGGGCACCTCTGAGAGTGAAAAATTGTATTGTGTGTATGTAGCAATTGG [629]

Huperzia_atp1_238360_237880 -------------------------------------------------- [0]

Huperzia_atp1_4707_5191 -------------------------------------------------- [485]

Huperzia_atp1_240083_239893 -------------------------------------------------- [191]

Huperzia_atp1_235705_235625 -------------------------------------------------- [81]

Huperzia_atp1_292893_293673 .G.............G......G.......G...........C.G..... [406]

Huperzia_atp1_129376_129434 -------------------------------------------------- [0]

Huperzia_atp1_330104_330468 -------------------------------------------------- [0]

Isoetes_HQ616419 ..............T.......G.....................G..C.. [617]

Zea_perennis AG......AAA.........G.C............C.....T..G..... [629]

Arabidopsis AG..C...............G.C.A..........C........G..... [629]

Cycas A.............T...............C....C........G..... [629]

Phaeoceros ..A.........A.T.....T............................. [626]

Megaceros ............A.T.....G............................. [626]

Physcomitrella ...........G..T.............................G..... [641]

Anomodon ...........G..T.............................G..... [641]

Treubia ........T.....T.............................G..... [626]

Marchantia ..............T....................A........G..... [626]

[ 710 720 730 740 750]

[ . . . . .]

Huperzia_atp1 ACAGAAACGTTCAACCGTGGCACAATTAGTTAAGATTCTT---TCAGAAG [676]

Huperzia_atp1_238360_237880 -------------------------------------------------- [0]

Huperzia_atp1_4707_5191 -------------------------------------------------- [485]

Huperzia_atp1_240083_239893 -------------------------------------------------- [191]

Huperzia_atp1_235705_235625 -------------------------------------------------- [81]

Huperzia_atp1_292893_293673 .G.T.......................G............CCAG...... [456]

Huperzia_atp1_129376_129434 -------------------------------------------------- [0]

Huperzia_atp1_330104_330468 -------------------------------------------------- [0]

Isoetes_HQ616419 G........C..............................---....... [664]

Zea_perennis ...A.....C..G..T...............C.A.....G---....... [676]

Arabidopsis .........C..G..T....G.......A..C.A.C....---GA..... [676]

Cycas .........C.......C.............C.A......---....G.. [676]

Phaeoceros .T................A........G............---....... [673]

Megaceros .T................A........G.......CC...---....... [673]

Physcomitrella ........................................---....... [688]

Anomodon ........................................---....... [688]

Treubia ..............................G.........---G...... [673]

Marchantia ........................................---....... [673]

[ 760 770 780 790 800]

[ . . . . .]

Huperzia_atp1 CAGGTGCTTTAGAATATTCCGTTATTGTAGCAGC---CACTGCTTCGGAT [723]

Huperzia_atp1_238360_237880 -------------------------------------------------- [0]

Huperzia_atp1_4707_5191 -------------------------------------------------- [485]

Huperzia_atp1_240083_239893 -------------------------------------------------- [191]

Huperzia_atp1_235705_235625 -------------------------------------------------- [81]

Huperzia_atp1_292893_293673 .G........G.......T.A......C......AGCT.......G.... [506]

Huperzia_atp1_129376_129434 -------------------------------------------------- [0]

Huperzia_atp1_330104_330468 -------------------------------------------------- [0]

Isoetes_HQ616419 .G................G.A....A........---............. [711]

Zea_perennis .GAA......G.........A.GC..........---...C......... [723]

Arabidopsis .GAA......G.........A..C..........---...C......... [723]

Cycas .G.A.....CG......C.AA....C........---...C......... [723]

Phaeoceros .G................G.A.....C.......---..G..T..TA... [720]

Megaceros .G........G.......G.A.....C.......---..G......A... [720]

Physcomitrella .G.....G..........G.A.............---T............ [735]

Anomodon .G................G.A.............---T............ [735]

Treubia .G..................A.............---............. [720]

Marchantia .G..................A.............---............. [720]

[ 810 820 830 840 850]

[ . . . . .]

Huperzia_atp1 CCTGCTCCTCTGCAA---TTCCTGGCACCATACTCAGGTTGTGCTATGGG [770]

Huperzia_atp1_238360_237880 -------------------------------------------------- [0]

Huperzia_atp1_4707_5191 -------------------------------------------------- [485]

Huperzia_atp1_240083_239893 -------------------------------------------------- [191]

Huperzia_atp1_235705_235625 -------------------------------------------------- [81]

Huperzia_atp1_292893_293673 .T.......AC....GAA...T..........T............G.... [556]

Huperzia_atp1_129376_129434 -------------------------------------------------- [0]

Huperzia_atp1_330104_330468 -------------------------------------------------- [0]

Isoetes_HQ616419 ...............---..............T...........A..... [758]

Zea_perennis ..A............---..T.....C.....T..T..G.....C..... [770]

Arabidopsis ...............---..TT....C.....T..C..G.....C..... [770]

Cycas ...............---..T.....C.....TC.T..G......GC... [770]

Phaeoceros .A..T....T.A...---.....T.....C..T...........G..... [767]

Megaceros .A.......T.A...---..T..T.....C..T........C..G..... [767]

Physcomitrella ...............---..............T........C........ [782]

Anomodon ...............---..............T........C........ [782]

Treubia ..A............---..............T................. [767]

Marchantia ..A......T.....---..............T................. [767]

[ 860 870 880 890 900]

[ . . . . .]

Huperzia_atp1 AGAATATTTTCGAGATAATGGAATGCACGCATTAATAATATATGATGATC [820]

Huperzia_atp1_238360_237880 -------------------------------------------------- [0]

Huperzia_atp1_4707_5191 -------------------------------------------------- [485]

Huperzia_atp1_240083_239893 -------------------------------------------------- [191]

Huperzia_atp1_235705_235625 -------------------------------------------------- [81]

Huperzia_atp1_292893_293673 ........C.............................GT.....G.... [606]

Huperzia_atp1_129376_129434 -------------------------------------------------- [0]

Huperzia_atp1_330104_330468 -------------------------------------------------- [0]

Isoetes_HQ616419 ...G......A.........................C............. [808]

Zea_perennis G........C..C..............T........T............. [820]

Arabidopsis G........C..C..........................C.......... [820]

Cycas .........C..C...................C......C.......... [820]

Phaeoceros ....................................C..C.......... [817]

Megaceros .....................G..............C..C.......... [817]

Physcomitrella ...G................................C..T........C. [832]

Anomodon ...G................................C..T........C. [832]

Treubia .........CA............................C.......... [817]

Marchantia ..........A............................C.......... [817]

[ 910 920 930 940 950]

[ . . . . .]

Huperzia_atp1 TGAGTAAGCAATCAGTGGCATATCGCCAAATGTCATTGTTATTACGTCGA [870]

Huperzia_atp1_238360_237880 -------------------------------------------------- [0]

Huperzia_atp1_4707_5191 -------------------------------------------------- [485]

Huperzia_atp1_240083_239893 -------------------------------------------------- [191]

Huperzia_atp1_235705_235625 -------------------------------------------------- [81]

Huperzia_atp1_292893_293673 A......................A.......AATG.C............. [656]

Huperzia_atp1_129376_129434 -------------------------------------------------- [0]

Huperzia_atp1_330104_330468 -------------------------------------------------- [0]

Isoetes_HQ616419 .....................................A............ [858]

Zea_perennis .A.....A..GG.G...........A...........A..G.....C... [870]

Arabidopsis .T.....A..GG.G...........A...........A..G.....C... [870]

Cycas CT.....A..GG.G...........A...........A..GC....C... [870]

Phaeoceros ...........GT.....T......G..G........A............ [867]

Megaceros ...........G.............G..G........A............ [867]

Physcomitrella .TTCG....................A...........A............ [882]

Anomodon .TTCA....................A...........A............ [882]

Treubia ...................G.................A............ [867]

Marchantia ...................G.................A............ [867]

[ 960 970 980 990 1000]

[ . . . . .]

Huperzia_atp1 CCTCCGGGTCGTGAGGCGT------TCCCAGGAGATGTTTTCTATCCACA [914]

Huperzia_atp1_238360_237880 -------------------------------------------------- [0]

Huperzia_atp1_4707_5191 -------------------------------------------------- [485]

Huperzia_atp1_240083_239893 -------------------------------------------------- [191]

Huperzia_atp1_235705_235625 -------------------------------------------------- [81]

Huperzia_atp1_292893_293673 ..A.G...G.T.....TTCGAAGAACGG..........G......T..-- [704]

Huperzia_atp1_129376_129434 -------------------------------------------------- [0]

Huperzia_atp1_330104_330468 -------------------------------------------------- [0]

Isoetes_HQ616419 ..A.....C..........------.............G..T...TT... [902]

Zea_perennis ..A..A..C........T.------....C..G............TT... [914]

Arabidopsis ..A..A..C........T.------.......T............TT... [914]

Cycas ..A..A..C........T.------.......G......C.....TT... [914]

Phaeoceros ..A..T..CT.........------.......G.....G......TT... [911]

Megaceros ..A..T..C..........------.......G.....C........... [911]

Physcomitrella ..A..A.............------.....................T... [926]

Anomodon ..A..A.............------....T...........T...TT... [926]

Treubia ..A..A.............------.............G......TT... [911]

Marchantia ..A..A.............------.............G......TT... [911]

[ 1010 1020 1030 1040 1050]

[ . . . . .]

Huperzia_atp1 TTCTCGTTTATTAGAAAGAGCCGCTAAAATGTCAGACCGAACTGGTGCGG [964]

Huperzia_atp1_238360_237880 -------------------------------------------------- [0]

Huperzia_atp1_4707_5191 -------------------------------------------------- [485]

Huperzia_atp1_240083_239893 -------------------------------------------------- [191]

Huperzia_atp1_235705_235625 -------------------------------------------------- [81]

Huperzia_atp1_292893_293673 -.......C............T...G..GG......TTCTT.C.A..... [753]

Huperzia_atp1_129376_129434 -------------------------------------------------- [0]

Huperzia_atp1_330104_330468 -------------------------------------------------- [0]

Isoetes_HQ616419 .....................T....................A....... [952]

Zea_perennis ...C...C.C..................CGA..G....AG..A.....A. [964]

Arabidopsis ...C...C.C...........G......CGA..G....AG..A.....A. [964]

Cycas ...C...CC..C................CGA..G....AG..A....... [964]

Phaeoceros ...C.....G............................AG........A. [961]

Megaceros .........G.C...............G..........AG.....C..A. [961]

Physcomitrella ......................................AG........A. [976]

Anomodon .......C...C..........................A.........A. [976]

Treubia ......................................AG.....C.... [961]

Marchantia ......................................AG.......... [961]

[ 1060 1070 1080 1090 1100]

[ . . . . .]

Huperzia_atp1 GTAGCTTGACAGCATTACCTGTCATTGAAACACAAGCTGGAGACGTATCT [1014]

Huperzia_atp1_238360_237880 -------------------------------------------------- [0]

Huperzia_atp1_4707_5191 -------------------------------------------------- [485]

Huperzia_atp1_240083_239893 -------------------------------------------------- [191]

Huperzia_atp1_235705_235625 -------------------------------------------------- [81]

Huperzia_atp1_292893_293673 ....G.C...C.......T..CG.....---------------------- [781]

Huperzia_atp1_129376_129434 ------------------------------...G.....T......G... [20]

Huperzia_atp1_330104_330468 ----------------------------..................G.T. [22]

Isoetes_HQ616419 ..........T...........G.......................G... [1002]

Zea_perennis ..........T..G.....C..G..........................G [1014]

Arabidopsis ..........C..C.....C.............................G [1014]

Cycas ..........C..G.....C..........................G..G [1014]

Phaeoceros ..........T...........G........................... [1011]

Megaceros ..........T...........G........................... [1011]

Physcomitrella ..........C...........A....................T...... [1026]

Anomodon ..........T...........A....................T...... [1026]

Treubia ..........T..GC.......G..........................C [1011]

Marchantia ..........T..GC.......T..........................C [1011]

[ 1110 1120 1130 1140 1150]

[ . . . . .]

Huperzia_atp1 GCTTATATTCCTACCAATGTAATTTCCATTACAGATGGACAAATCTTTTT [1064]

Huperzia_atp1_238360_237880 ---------------------------------------......C..C. [11]

Huperzia_atp1_4707_5191 -------------------------------------------------- [485]

Huperzia_atp1_240083_239893 -------------------------------------------------- [191]

Huperzia_atp1_235705_235625 -------------------------------------------------- [81]

Huperzia_atp1_292893_293673 -------------------------------------------------- [781]

Huperzia_atp1_129376_129434 A......C............G...C..............----------- [59]

Huperzia_atp1_330104_330468 .......C...........CG...C......................... [72]

Isoetes_HQ616419 ....................G.....T....................... [1052]

Zea_perennis ..C.....C..C........G..C......................G... [1064]

Arabidopsis ..C........C........G..C........T.............G... [1064]

Cycas ..C.....C...........G..CC....................CG..C [1064]

Phaeoceros ...........C........G............................. [1061]

Megaceros ...........C........G...C......................... [1061]

Physcomitrella ....................T............................. [1076]

Anomodon ...........G........T...C......................... [1076]

Treubia ....................G.....T.................A..... [1061]

Marchantia ....................G............................. [1061]

[ 1160 1170 1180 1190 1200]

[ . . . . .]

Huperzia_atp1 GGAAACAGAACTCTTTTATCGTGGAATTCGACCTGCTATTAACGTGGGAT [1114]

Huperzia_atp1_238360_237880 ................CG....A...............C........A.. [61]

Huperzia_atp1_4707_5191 -------------------------------------------------- [485]

Huperzia_atp1_240083_239893 -------------------------------------------------- [191]

Huperzia_atp1_235705_235625 -------------------------------------------------- [81]

Huperzia_atp1_292893_293673 -------------------------------------------------- [781]

Huperzia_atp1_129376_129434 -------------------------------------------------- [59]

Huperzia_atp1_330104_330468 ......................................C........... [122]

Isoetes_HQ616419 .................................................. [1102]

Zea_perennis .........G...........C......A....A...........T..C. [1114]

Arabidopsis .........G...........C......A................C..C. [1114]

Cycas .........G...........C.....CA................C..C. [1114]

Phaeoceros ............................T...T................. [1111]

Megaceros ............................................C..... [1111]

Physcomitrella ............T................................A.... [1126]

Anomodon ............T.............................T..A.... [1126]

Treubia ..........................G......C................ [1111]

Marchantia ..........................G....................... [1111]

[ 1210 1220 1230 1240 1250]

[ . . . . .]

Huperzia_atp1 TATCAGTGAGTCGCGTTGGTTCTGCGGCTCAGTTGAAAGCTATGAAACAA [1164]

Huperzia_atp1_238360_237880 ...GT......T...CC..G-.G........................... [110]

Huperzia_atp1_4707_5191 -------------------------------------------------- [485]

Huperzia_atp1_240083_239893 -------------------------------------------------- [191]

Huperzia_atp1_235705_235625 -------------------------------------------------- [81]

Huperzia_atp1_292893_293673 -------------------------------------------------- [781]

Huperzia_atp1_129376_129434 -------------------------------------------------- [59]

Huperzia_atp1_330104_330468 ....T......T....................................G. [172]

Isoetes_HQ616419 .......TTC......C................C......C......... [1152]

Zea_perennis ....C..C........C..G..C..C........................ [1164]

Arabidopsis ....T..C........C..G.....C........................ [1164]

Cycas ....C..C........C..G.....C...................G.... [1164]

Phaeoceros ....T......T.T.......T.......T.A...............T.. [1161]

Megaceros ....T...........C................................. [1161]

Physcomitrella ....T..A.....T..G................................G [1176]

Anomodon ....T..A.....T..GA...............CA..............G [1176]

Treubia ....T...........C.......................C......... [1161]

Marchantia ....T...........C.......................C......... [1161]

[ 1260 1270 1280 1290 1300]

[ . . . . .]

Huperzia_atp1 GTCTGTGGTAGCTTAAAACTAGAAT---TGGCACAATATC---GTGAAGT [1208]

Huperzia_atp1_238360_237880 .........G...C.....C.....---...........T---A...... [154]

Huperzia_atp1_4707_5191 -------------------------------------------------- [485]

Huperzia_atp1_240083_239893 -------------------------------------------------- [191]

Huperzia_atp1_235705_235625 -------------------------------------------------- [81]

Huperzia_atp1_292893_293673 -------------------------------------------------- [781]

Huperzia_atp1_129376_129434 -------------------------------------------------- [59]

Huperzia_atp1_330104_330468 .............C...........AAT............GTA....... [222]

Isoetes_HQ616419 ..T...........G..........---....G.......---....... [1196]

Zea_perennis .....C.....T.C......G....---............---.C..... [1208]

Arabidopsis ..A..C.....T.C......G....---............---.C..... [1208]

Cycas ..AC.......T.C.....C.....---............---....... [1208]

Phaeoceros ..A......................---.......G....---....G.. [1205]

Megaceros ..A......................---.......G....---....G.. [1205]

Physcomitrella ..A......................---....G.......---.C..... [1220]

Anomodon ..A..C........G..........---.A..G.......---....... [1220]

Treubia ..A..C..............G....---............---....... [1205]

Marchantia ..A..C...................---............---....... [1205]

[ 1310 1320 1330 1340 1350]

[ . . . . .]

Huperzia_atp1 AGCCGCTTTTGCTCAATTTGGTT---CAGACCTCGACGCGGCTACTCAGT [1255]

Huperzia_atp1_238360_237880 ........C........C....GCAG......CT.....T.......... [204]

Huperzia_atp1_4707_5191 -------------------------------------------------- [485]

Huperzia_atp1_240083_239893 -------------------------------------------------- [191]

Huperzia_atp1_235705_235625 -------------------------------------------------- [81]

Huperzia_atp1_292893_293673 -------------------------------------------------- [781]

Huperzia_atp1_129376_129434 -------------------------------------------------- [59]

Huperzia_atp1_330104_330468 ...T....C........C..A..TAG......CG..---T.......C.. [269]

Isoetes_HQ616419 T............G....C....---.......T.....T......A... [1243]

Zea_perennis G.....C..C...........G.---.......T..T..T..G......G [1255]

Arabidopsis G.....C..............C.---.......T..T..T..G......G [1255]

Cycas G.....C.C........CC..G.---.......T..T..T.........G [1255]

Phaeoceros ...T....C..............---.......T..T..T....T.A... [1252]

Megaceros ........C.........C....---......CT..T..T......A... [1252]

Physcomitrella ..................C....---.......T..T..T.......... [1267]

Anomodon .................C.....---.......T..T............. [1267]

Treubia .......................---.......T..T..T..G....... [1252]

Marchantia .......................---.......T..T..T..G....... [1252]

[ 1360 1370 1380 1390 1400]

[ . . . . .]

Huperzia_atp1 ATTTGTTAAATCGTGGGGCTA---------GGCTAACAGAGGTTCTTAAA [1296]

Huperzia_atp1_238360_237880 ...C..C....T..A......---------...CG.ACTTC..G.C.... [245]

Huperzia_atp1_4707_5191 -------------------------------------------------- [485]

Huperzia_atp1_240083_239893 -------------------------------------------------- [191]

Huperzia_atp1_235705_235625 -------------------------------------------------- [81]

Huperzia_atp1_292893_293673 -------------------------------------------------- [781]

Huperzia_atp1_129376_129434 -------------------------------------------------- [59]

Huperzia_atp1_330104_330468 ...C..C....T...A...A.ATAGGCGGC.......GC........... [319]

Isoetes_HQ616419 ....A..G.............---------.................... [1284]

Zea_perennis CA..AC.C...A.A..T..A.---------....T.....A..G.CC... [1296]

Arabidopsis CA..AC.C...A.A..T..A.---------....G.....A..A.CG... [1296]

Cycas CACCA.C....A.A..T....---------....T..T..A....CG... [1296]

Phaeoceros ....A...........A....---------....C..G..A...TC.... [1293]

Megaceros ....A.C.........A....---------.......G..A....C.... [1293]

Physcomitrella ....A................---------.................... [1308]

Anomodon ..C.A..............G.---------.................... [1308]

Treubia ....A................---------..T.G..T...A.A...... [1293]

Marchantia ....A...........A....---------..T....T...A.A...... [1293]

[ 1410 1420 1430 1440 1450]

[ . . . . .]

Huperzia_atp1 CAACCACAATATAGCCCACTTCCTATTGAAAAACAAATTGTGGTTATTTA [1346]

Huperzia_atp1_238360_237880 .................................-..G............. [294]

Huperzia_atp1_4707_5191 -------------------------------------------------- [485]

Huperzia_atp1_240083_239893 -------------------------------------------------- [191]

Huperzia_atp1_235705_235625 -------------------------------------------------- [81]

Huperzia_atp1_292893_293673 -------------------------------------------------- [781]

Huperzia_atp1_129376_129434 -------------------------------------------------- [59]

Huperzia_atp1_330104_330468 ......G.TAGCC......CA.A.TCCT..C..---..C..........- [365]

Isoetes_HQ616419 .................................G....C........... [1334]

Zea_perennis ............GAG........A.................T........ [1346]

Arabidopsis ............GCA........A..............AC.A..C..... [1346]

Cycas ............CCA........................CCA......C. [1346]

Phaeoceros ....T...G.......T.A....A.....G..................C. [1343]

Megaceros ........G.........A....A.....G..................C. [1343]

Physcomitrella ..................A..T................A........... [1358]

Anomodon ..................A...................A..T........ [1358]

Treubia ..................A...................C........... [1343]

Marchantia ...G..............A...................C........... [1343]

[ 1460 1470 1480 1490 1500]

[ . . . . .]

Huperzia_atp1 TGCTGCTGTCAAAGGTTA-TTTGGATCAAATTCCTATTTCGATCATTAAT [1395]

Huperzia_atp1_238360_237880 ..................A.CCA...............C........... [344]

Huperzia_atp1_4707_5191 -------------------------------------------------- [485]

Huperzia_atp1_240083_239893 -------------------------------------------------- [191]

Huperzia_atp1_235705_235625 -------------------------------------------------- [81]

Huperzia_atp1_292893_293673 -------------------------------------------------- [781]

Huperzia_atp1_129376_129434 -------------------------------------------------- [59]

Huperzia_atp1_330104_330468 ---------...GCTC.TG.....T.T----------------------- [383]

Isoetes_HQ616419 C.................-...A...G....A......C.......CG.G [1383]

Zea_perennis ............C..C.T-C.GT....G...G..AC.AGAC.GA...TC. [1395]

Arabidopsis ...A........T..A.T-C.GT....G...G..AC.AGAC.GA..CTC. [1395]

Cycas C..A.....G.....G.T-.CGT....G...G..AC.AGAC.GA...CC. [1395]

Phaeoceros ...A..............-...A...G.............A..T...... [1392]

Megaceros ...A..............-...A...G.............A..T...... [1392]

Physcomitrella ...A..............-...A...................G....... [1407]

Anomodon ...A..............-...A...................G...C... [1407]

Treubia ...A.....A........-C..A..C.....A..CG.GGTTC....A.CG [1392]

Marchantia ...A..............-C..A..C.....A..CG.CG.TC....A.CG [1392]

[ 1510 1520 1530 1540 1550]

[ . . . . .]

Huperzia_atp1 AAGTATGAACAGGAGCTATTGAAGTCTATTGACCCAGGTATACTTTCTGC [1445]

Huperzia_atp1_238360_237880 C.A........A....-----............................. [389]

Huperzia_atp1_4707_5191 -------------------------------------------------- [485]

Huperzia_atp1_240083_239893 -------------------------------------------------- [191]

Huperzia_atp1_235705_235625 -------------------------------------------------- [81]

Huperzia_atp1_292893_293673 -------------------------------------------------- [781]

Huperzia_atp1_129376_129434 -------------------------------------------------- [59]

Huperzia_atp1_330104_330468 -------------------------------------------------- [383]

Isoetes_HQ616419 ..A........A.......................T............T. [1433]

Zea_perennis C.A......A.AA.CA.TC.A.GTA.....A.T..T.AAT....-CAAAT [1444]

Arabidopsis C.A.....GA.A.CCA.TCCA..TAG.G.CA.A..T.AAT....ACAA.. [1445]

Cycas CG......GAGA.CCA.T.CA.GTAG...A..T..C.AAT....ACAAT. [1445]

Phaeoceros G.A........T....C.......C.......T...A.........T..T [1442]

Megaceros G.A........T....C...............T...A............. [1442]

Physcomitrella C.A........T.....T...........A.......A............ [1457]

Anomodon C.A........T.....T...........A...T...A............ [1457]

Treubia C.C........A...........A.......................... [1442]

Marchantia C.C........A...........A...............C.......... [1442]

[ 1560 1570 1580 1590 1600]

[ . . . . .]

Huperzia_atp1 TATTGT-ACAACAAAAAAGCATCACTGAGCAAATAAACACTCAACTGGCT [1494]

Huperzia_atp1_238360_237880 ......-...........A............................... [438]

Huperzia_atp1_4707_5191 -------------------------------------------------- [485]

Huperzia_atp1_240083_239893 -------------------------------------------------- [191]

Huperzia_atp1_235705_235625 -------------------------------------------------- [81]

Huperzia_atp1_292893_293673 -------------------------------------------------- [781]

Huperzia_atp1_129376_129434 -------------------------------------------------- [59]

Huperzia_atp1_330104_330468 -------------------------------------------------- [383]

Isoetes_HQ616419 ...C.C-GG..G.G..G.....A................G.......... [1482]

Zea_perennis CC..C.T.G..A..GGTG..T.A...A.CG...G...G.TGG...CT.A. [1494]

Arabidopsis CC..-------A..GGTG.AT.A...A.CG...G...A.TGG...CA.A. [1488]

Cycas CG..CC-GG..A..GGTGAGT.A...A.CG......GG.TGA...CA.A. [1494]

Phaeoceros ......-GA..T...........T.....G....T....G......T..G [1491]

Megaceros ......-TA..............T.....G....T....G......T..G [1491]

Physcomitrella ...C..-...........A...T........G..T....G.......... [1506]

Anomodon ...C..-...........A...T........G..TG.T.G.......... [1506]

Treubia ......-...........AT..............T.G..G.......... [1491]

Marchantia ......-...........A...............T.GT.G.......... [1491]

[ 1610 1620 1630 1640 1650]

[ . . . . .]

Huperzia_atp1 ACCTTTTGCCAAAGATTTACACAGAGCTTCCTAGCTACTCATTCGGTTTA [1544]

Huperzia_atp1_238360_237880 ......G..............A.C...................------- [481]

Huperzia_atp1_4707_5191 -------------------------------------------------- [485]

Huperzia_atp1_240083_239893 -------------------------------------------------- [191]

Huperzia_atp1_235705_235625 -------------------------------------------------- [81]

Huperzia_atp1_292893_293673 -------------------------------------------------- [781]

Huperzia_atp1_129376_129434 -------------------------------------------------- [59]

Huperzia_atp1_330104_330468 -------------------------------------------------- [383]

Isoetes_HQ616419 .......A.A..GA..................TCT.TTC..CGGACC.AG [1532]

Zea_perennis G.T.C..TAA..GA.AGCG.TTTA.AT..A--T.A--------------- [1527]

Arabidopsis G.T..C.TAA..GA.AGAG.TTTAGCT..AA.TTAG-------------- [1524]

Cycas G.TCC..CAA..GA.AGCG--TTA.C...TGCT.A--------------- [1527]

Phaeoceros .........A...A................T....A.............. [1541]

Megaceros ......C..A...A...G............T....A.............G [1541]

Physcomitrella ........T....A................T....G........A..... [1556]

Anomodon ........T....A................T....A........A...C. [1556]

Treubia .............A......G.........T....A......CAATCA.. [1541]

Marchantia .............A......G.........T....A......CAATCA.. [1541]

[ 1660]

[ . ]

Huperzia_atp1 A------------ [1545]

Huperzia_atp1_238360_237880 ------------- [481]

Huperzia_atp1_4707_5191 ------------- [485]

Huperzia_atp1_240083_239893 ------------- [191]

Huperzia_atp1_235705_235625 ------------- [81]

Huperzia_atp1_292893_293673 ------------- [781]

Huperzia_atp1_129376_129434 ------------- [59]

Huperzia_atp1_330104_330468 ------------- [383]

Isoetes_HQ616419 .AAAAGAGCCTAG [1545]

Zea_perennis ------------- [1527]

Arabidopsis ------------- [1524]

Cycas ------------- [1527]

Phaeoceros .------------ [1542]

Megaceros .------------ [1542]

Physcomitrella .------------ [1557]

Anomodon .------------ [1557]

Treubia .------------ [1542]

Marchantia .------------ [1542]

;

END;

**atp4**

#NEXUS

[MacClade 4.05 registered to Yin-Long Qiu Lab, EEB, University of Michigan]

BEGIN DATA;

DIMENSIONS NTAX=16 NCHAR=704;

FORMAT DATATYPE=DNA MISSING=? GAP=- MATCHCHAR=. INTERLEAVE ;

MATRIX

[ 10 20 30 40 50]

[ . . . . .]

Huperzia_atp4 ATGCGTGA------------------------ATTGGTTATATTTGCTAT [26]

Huperzia_atp4_182713_182577 -------------------------------------------------- [0]

Huperzia_atp4_83845_83491 --......------------------------.................. [24]

Huperzia_atp4_319599_319953 --......------------------------.................. [24]

Huperzia_atp4_331669_332179 ......A.------------------------..C.............C. [26]

Huperzia_atp4_275637_275582 -------------------------------------------------- [0]

Isoetes ...AACA.CAA---------------------...CT..G....C..CG. [29]

Zea_perennis ...A.ATTTAGTGGAATGGATATGAAGGGTAT.AATA.GC........GC [50]

Vitis ...A.ATTGAGTTCCACGAATATGCAGGCTAG.AA.A.GC........GC [50]

Cycas ...ATATTTAGTTCCACGAATAGGAAGGAGAG.AATA.GC........GC [50]

Megaceros ........------------------------....A.A........... [26]

Phaeoceros ........------------------------....A.A........... [26]

Physcomitrella ........------------------------...TA........A.... [26]

Anomodon ......A.------------------------...TA.......C..... [26]

Marchantia ........------------------------.A.AT.A........A.. [26]

Treubia ........------------------------.A.AT.A.......TC.. [26]

[ 60 70 80 90 100]

[ . . . . .]

Huperzia_atp4 TT---CAATTCTTAGTGTTTCGAGTCCAAAACAAATCTTAATTTATAATG [73]

Huperzia_atp4_182713_182577 -------------------------------------------------- [0]

Huperzia_atp4_83845_83491 ..---......C...................................... [71]

Huperzia_atp4_319599_319953 ..---......C...................................... [71]

Huperzia_atp4_331669_332179 ..TA-...C.T....C.........TA.....G......G...C...... [75]

Huperzia_atp4_275637_275582 -------------------------------------------------- [0]

Isoetes ------GG..A..CT....CTT...T.....A------.G....C..... [67]

Zea_perennis .ATTC..TC.A..T...CA..A.....G..GA.G....C...C....... [100]

Vitis .ATTC..TC.A..T...CA..A...T.G..GA.G....C...C....... [100]

Cycas .ATTCT.T..A..C....A.TA..CT.G..GA.G....C...A....... [100]

Megaceros .C---.....TC...............T...T.................. [73]

Phaeoceros .C---T....TC........T....T.T...T.................. [73]

Physcomitrella ..---T....T.........TA...T........................ [73]

Anomodon ..---T....TC........TA...T........................ [73]

Marchantia ..---T..GCT....C....TA...T.....A..........A....... [73]

Treubia ..---T..GCT....C....TA...T.G...A..G.A.....A....... [73]

[ 110 120 130 140 150]

[ . . . . .]

Huperzia_atp4 AAGAAATTGTGGTAGCTTTAAGTTTTGTGCTTTTTGT--TATATTTAGTC [121]

Huperzia_atp4_182713_182577 -------------------------------------------------- [0]

Huperzia_atp4_83845_83491 ........T................G...G.......CTC.......... [121]

Huperzia_atp4_319599_319953 ........T................G...G.......CTC.......... [121]

Huperzia_atp4_331669_332179 ....G...A........CCT.....G..AG.......--CG......... [123]

Huperzia_atp4_275637_275582 -------------------------------------------------- [0]

Isoetes .G....GCC.T..............G...G.......--.......G..G [115]

Zea_perennis .......GA.A......CGTT.....A.AGGC...C.--C.....A.... [148]

Vitis .......GA.A......CGTT.....A.AGGC...A.--C.....A.... [148]

Cycas .......GA.A.......GTC.....A.AGGC...A.--C....CC.... [148]

Megaceros .....G..C.T......CGT......A.TGG......--..........G [121]

Phaeoceros .....G..T.T.......GT......A.TGG......--..........G [121]

Physcomitrella ........A.T.................AGG......--..........A [121]

Anomodon ........A.T.................AGG......--........... [121]

Marchantia .....G..A.T..................TG......--........... [121]

Treubia .....G..A.T..................TG...C..--..........A [121]

[ 160 170 180 190 200]

[ . . . . .]

Huperzia_atp4 AAAAAACTTTTGGTGAAACTTTCAAAGCGACTTTGGAGGCGAGAAGCGAA [171]

Huperzia_atp4_182713_182577 -------------------------------------------------- [0]

Huperzia_atp4_83845_83491 ...........C----.................................. [167]

Huperzia_atp4_319599_319953 ...........C----.................................. [167]

Huperzia_atp4_331669_332179 ..G.......C...C................................... [173]

Huperzia_atp4_275637_275582 -------------------------------------------------- [0]

Isoetes ..TTT..G..G....G...............CC.T..T............ [165]

Zea_perennis GG..G.G...A...A.G..........AA...C.C..C.G.....T...G [198]

Vitis GG..G.G...A...A.G.....A....T....C.C..C.G.....T.C.G [198]

Cycas .G..G.G..CG...A.T...C.........T...C..C.G.....T..G. [198]

Megaceros ......................TG....T....CTA.T............ [171]

Phaeoceros ......................TG....T....CTA.T............ [171]

Physcomitrella .......C..............A.....T....CT..T...C........ [171]

Anomodon .......C..........T...A.....T....CT..T...C........ [171]

Marchantia .......A............A.A.....T.T...T..T............ [171]

Treubia .......A............A.A...........T..T............ [171]

[ 210 220 230 240 250]

[ . . . . .]

Huperzia_atp4 GCTATTCTTTCAGAGTTACAGCATTTGATGAGTTC---TCAAGAAGCTTT [218]

Huperzia_atp4_182713_182577 -------------------------------------------------- [0]

Huperzia_atp4_83845_83491 .......C...........................---............ [214]

Huperzia_atp4_319599_319953 .......C...........................---............ [214]

Huperzia_atp4_331669_332179 .......C...........................---............ [220]

Huperzia_atp4_275637_275582 -------------------------------------------------- [0]

Isoetes A.C...............G...GGC.C...G....AAC.G........AA [215]

Zea_perennis T......AGGA.TCA..G.....A..CT.C.A.C.---.A.C....TCA. [245]

Vitis .......AGGA...A.CG.....A..CCCC.A.C.---.A.C....TAG. [245]

Cycas .......AGAA...A..G.....A..CCCC.A.C.---CA.C....T.G. [245]

Megaceros ..A...T................A.....A.....---.A....G...C. [218]

Phaeoceros ..A...T................A.....A.....---.A....G...C. [218]

Physcomitrella ...C...................A...........---..........C. [218]

Anomodon ...C...........C.......A...........---..........C. [218]

Marchantia ...C.......C..T........A.G........A---..........A. [218]

Treubia ...C.......C..T........A.G........A---..........A. [218]

[ 260 270 280 290 300]

[ . . . . .]

Huperzia_atp4 GTGGTCCGAATCAAAGAAACAGCACGAATTACGTA------GTATCAGCT [262]

Huperzia_atp4_182713_182577 -------------------------------------------------- [0]

Huperzia_atp4_83845_83491 ........GG.T.C..........T..........------......... [258]

Huperzia_atp4_319599_319953 ........GG.T.C..........T..........------......... [258]

Huperzia_atp4_331669_332179 ........................T..........------......... [264]

Huperzia_atp4_275637_275582 -------------------------------------------------- [0]

Isoetes .A-----.T..TTT...T.T.A-.TC...CG....------CGGAAT... [253]

Zea_perennis TCT.GAG.....C..TG....A..ACG.....T..ATCTAC.G....... [295]

Vitis TCCTC.G.....C..TG....A..ACG.....T..------.G....... [289]

Cycas T.CTC.G.....C..TG....A..AC......T..------.G....... [289]

Megaceros ..T......G.T............T......T.C.------....A.... [262]

Phaeoceros ..T......G.T.......T....TA.....T...------....A..T. [262]

Physcomitrella ..T......G.TG...........T..........------....A..T. [262]

Anomodon ..T......G.TG...........T..........------....A..T. [262]

Marchantia ..T........T...A........T..........------....A.... [262]

Treubia ..T.........G...........T..........------....A.... [262]

[ 310 320 330 340 350]

[ . . . . .]

Huperzia_atp4 TGCGTTCAAGC----ACG-CAAATGATTGGA-GAATCATGTATAAAT-AA [305]

Huperzia_atp4_182713_182577 -------------------------------------------------- [0]

Huperzia_atp4_83845_83491 ...........----...-............-....T..........-G. [301]

Huperzia_atp4_319599_319953 ...........----...-............-....T..........-G. [301]

Huperzia_atp4_331669_332179 ...........----..A-............TA...T.C---.....-G. [305]

Huperzia_atp4_275637_275582 -------------------------------------------------- [0]

Isoetes ...TC.T..AT----...G.C.G.CT.A..TTT...TT.AGGCT.C.AG. [299]

Zea_perennis ....---..TTTGCAG.AC.GT.AA.G.A.TA.......TACC.G----- [337]

Vitis ....---..TTTGTGG.AC.GT.------.TA.......TACC..----- [325]

Cycas .........TT----...C..G.GA--.T.TA.......TACC....--- [330]

Megaceros ........G.T----...-............-........C......-G. [305]

Phaeoceros ........G.T----...-............-........C......-G. [305]

Physcomitrella ..........T----...-............-.....T.........-G. [305]

Anomodon ..........T----...-............-.....T.........-G. [305]

Marchantia ..........T----..A-............-...............-G. [305]

Treubia ..........T----..A-............-...............-G. [305]

[ 360 370 380 390 400]

[ . . . . .]

Huperzia_atp4 TAT--GGTTACGCGCTGTGCACCTAAGTGCAAACAAACAGTGCAAGCTGC [353]

Huperzia_atp4_182713_182577 -------------------------------------------------- [0]

Huperzia_atp4_83845_83491 ...--............................---..C.........T. [346]

Huperzia_atp4_319599_319953 ...--............................---..C.........T. [346]

Huperzia_atp4_331669_332179 ...AT............................----.......G....T [351]

Huperzia_atp4_275637_275582 -------------------------------------------------- [0]

Isoetes ...--...GG------AA.G........A....G.G..C..........T [341]

Zea_perennis -------CGG.A........G.........G..A.G............TT [380]

Vitis -------.GG.A........G.........G..A.G............TT [368]

Cycas ---GA.ACGG.A........G.........G..A.G.............T [377]

Megaceros ...--...............G..............G............AT [353]

Phaeoceros ...--............C..G..............G.............T [353]

Physcomitrella .C.--.C..........C.................G.............T [353]

Anomodon .C.--.C..........C.................G.............T [353]

Marchantia ...--...............G..A...........G.....AA..T...T [353]

Treubia ...--............C..G..A...........G......A..T...T [353]

[ 410 420 430 440 450]

[ . . . . .]

Huperzia_atp4 GTTAGGCCAGCAAATAGAGCTTCAGTTAAAAACACTGTTA---GCTATGA [400]

Huperzia_atp4_182713_182577 -------------..GA..--------------------G---.TC..-- [12]

Huperzia_atp4_83845_83491 ....T....----------------------------------------- [355]

Huperzia_atp4_319599_319953 ....T....----------------------------------------- [355]

Huperzia_atp4_331669_332179 ....T...CA.C......C.....A...............---..G.... [398]

Huperzia_atp4_275637_275582 -------------------------------------------------- [0]

Isoetes ....T....AGGG..G......A....CG...........---..C.... [388]

Zea_perennis ....T...GAA.CC..A.TG.CA...C.GC......TC..AAT..C.CT- [429]

Vitis ....T...GAA.CC..A.TG..A...C.GC......TCC.AAT..C.CT- [417]

Cycas ....T...GAA.CC..A.TG.AA.....GC......TCC.AAT..C..T- [426]

Megaceros A...T....A.....G..A.AGA.................---.....TC [400]

Phaeoceros A..GT....A.....G..A.AGA.................---.....TC [400]

Physcomitrella ....T....A...G......AAA.................---.....TC [400]

Anomodon ....T....A...G......AAA....G........A...---.....TC [400]

Marchantia ....T....A..........AAA.................---.....TC [400]

Treubia ....T....A.....G....AAA...........T.....---.....TC [400]

[ 460 470 480 490 500]

[ . . . . .]

Huperzia_atp4 AAGAGCATTATTCTCGCATCCGTTTTCAAGACAAGATAGTAACTTGTTTC [450]

Huperzia_atp4_182713_182577 -----T...T.CG---.................................. [54]

Huperzia_atp4_83845_83491 -------------------------------------------------- [355]

Huperzia_atp4_319599_319953 -------------------------------------------------- [355]

Huperzia_atp4_331669_332179 ...G.....C.CG-------.....................--------- [432]

Huperzia_atp4_275637_275582 -------------------------------------------------- [0]

Isoetes .........C.CA---................G..G............C. [435]

Zea_perennis -----TC..CCCG..........C....G...G.T.....C..AG....T [474]

Vitis -----TC..CCCG..........C....G...G.TC....C..AG....T [462]

Cycas -----T...CCCG..........C...GG...G.T.....C..AG....T [471]

Megaceros .........C.CG---...GA....A..G..G........C........T [447]

Phaeoceros .........C.CG---...GA....A..G..G.................T [447]

Physcomitrella .........C.CG---..G.A....A.....G........C........T [447]

Anomodon .........C.CG---..G.A....A.....G........C..C.....T [447]

Marchantia .........C.CG---T...A....A..G..G.................T [447]

Treubia .........C.CG---T...A....A..G.GGG................T [447]

[ 510 520 530 540 550]

[ . . . . .]

Huperzia_atp4 CGCTTCTCAGTGGATGACGAATTTCGCTTTTCGAAATTGC---------G [491]

Huperzia_atp4_182713_182577 .....T....................A..........G..---------. [95]

Huperzia_atp4_83845_83491 -------------------------------------------------- [355]

Huperzia_atp4_319599_319953 -------------------------------------------------- [355]

Huperzia_atp4_331669_332179 -....................................G..---------. [472]

Huperzia_atp4_275637_275582 ----------------...........G.......G.G..---------. [25]

Isoetes .....TGTT..T.G.........C.....C..T.......---------. [476]

Zea_perennis .A...T......AG...AAG....--G.A.C..GGTC.A.AACTTTGGTA [522]

Vitis .A..........AG...AAG....--G.CCC..GGTG.A.------GTT. [504]

Cycas .A..........AG...AA.....--G.CCC.TGC.CCA.CGATACGAAA [519]

Megaceros ...GAAA....TTG..................C.......---------. [488]

Phaeoceros ...GAAA....TTG..................C.......---------. [488]

Physcomitrella ...GAAA....TTG..................T.......---------. [488]

Anomodon ...GAAA....TTG..................T.......---------. [488]

Marchantia ...GAAA....TTG..................C.......---------. [488]

Treubia ...GAAA....TTG..................A.......---------. [488]

[ 560 570 580 590 600]

[ . . . . .]

Huperzia_atp4 AAAACATCAGTC--AAAAC------------------------------- [508]

Huperzia_atp4_182713_182577 ............--.....------------------------------- [112]

Huperzia_atp4_83845_83491 -------------------------------------------------- [355]

Huperzia_atp4_319599_319953 -------------------------------------------------- [355]

Huperzia_atp4_331669_332179 ----....T...--G....------------------------------- [485]

Huperzia_atp4_275637_275582 G....C......--.....------------------------------- [42]

Isoetes G...GC......--..CCT------------------------------- [493]

Zea_perennis G..G.T..TAC.GT.G...AAATTCGAGAGGCCTTCTTATTAGAACCCAG [572]

Vitis ...G.T..TA.AGT.G...------------------------------- [523]

Cycas ...G.T.TTA..GC.G...------------------------------- [538]

Megaceros ............--T....------------------------------- [505]

Phaeoceros ............--T....------------------------------- [505]

Physcomitrella ............--.....------------------------------- [505]

Anomodon .........A..--G....------------------------------- [505]

Marchantia ............--.....------------------------------- [505]

Treubia ............--G....------------------------------- [505]

[ 610 620 630 640 650]

[ . . . . .]

Huperzia_atp4 -----TGGTTCAACAAAGCATGGTACTATTGCAAAGGAAAAAACACAATG [553]

Huperzia_atp4_182713_182577 -----.A..................T....-------------------- [137]

Huperzia_atp4_83845_83491 -------------------------------------------------- [355]

Huperzia_atp4_319599_319953 -------------------------------------------------- [355]

Huperzia_atp4_331669_332179 -----....................T.....------------------- [511]

Huperzia_atp4_275637_275582 -----.........G....------------------------------- [56]

Isoetes -----......G............CT.....A..GATGGGGCCA.G.TGC [538]

Zea_perennis AGACC.AA...G.G..G..T.TA..G.CC.AAG..A---GGTGAGGGTG. [619]

Vitis -----.CA...G.G.GG..T...CGG.C..AAG..T---GGTT.GGGTG. [565]

Cycas -----C.A.C.G.G..G..T....GG...CAAT..C---GGT-------- [572]

Megaceros -----.A..................T.....A..GATGGGGTT.TG..AT [550]

Phaeoceros -----.A..................T.....A..GATGGGGTT.TG..AT [550]

Physcomitrella -----.A..................T.....A..GATGG.GTT.TG..AT [550]

Anomodon -----.A..................T.....A..GATGG.GTT.TG..AT [550]

Marchantia -----.A..................T.....A..GATGGGGTT.CG..AT [550]

Treubia -----.A..................T.....A..GATGGGGTT.CG..AT [550]

[ 660 670 680 690 700]

[ . . . . .]

Huperzia_atp4 AAAACACAAATGAACTTTAA------------------------------ [573]

Huperzia_atp4_182713_182577 -------------------------------------------------- [137]

Huperzia_atp4_83845_83491 -------------------------------------------------- [355]

Huperzia_atp4_319599_319953 -------------------------------------------------- [355]

Huperzia_atp4_331669_332179 -------------------------------------------------- [511]

Huperzia_atp4_275637_275582 -------------------------------------------------- [56]

Isoetes ..GCA.TTCTC.GT.GAGG.----GAAGAGATTGGAAAAACAAGTAAAGT [584]

Zea_perennis GGGGT.TCCCC.GGAAGAG.TCCAGTGGAGACGGGGTGGGCCTGTAG--- [666]

Vitis GGGGTT.TCT.A.GAA.A..------GAAGACGAA---------TAG--- [597]

Cycas ------TTCTC..-AA.GG.------GGGCATCGA--------------- [594]

Megaceros G.------------------------------------------------ [552]

Phaeoceros G.------------------------------------------------ [552]

Physcomitrella G.------------------------------------------------ [552]

Anomodon G.------------------------------------------------ [552]

Marchantia G.------------------------------------------------ [552]

Treubia G.------------------------------------------------ [552]

[ ]

[ ]

Huperzia_atp4 ---- [573]

Huperzia_atp4_182713_182577 ---- [137]

Huperzia_atp4_83845_83491 ---- [355]

Huperzia_atp4_319599_319953 ---- [355]

Huperzia_atp4_331669_332179 ---- [511]

Huperzia_atp4_275637_275582 ---- [56]

Isoetes ATGA [588]

Zea_perennis ---- [666]

Vitis ---- [597]

Cycas ---- [594]

Megaceros ---- [552]

Phaeoceros ---- [552]

Physcomitrella ---- [552]

Anomodon ---- [552]

Marchantia ---- [552]

Treubia ---- [552]

;

END;

**atp6**

#NEXUS

[MacClade 4.05 registered to Yin-Long Qiu Lab, EEB, University of Michigan]

BEGIN DATA;

DIMENSIONS NTAX=12 NCHAR=1263;

FORMAT DATATYPE=DNA MISSING=? GAP=- MATCHCHAR=. INTERLEAVE ;

MATRIX

[ 10 20 30 40 50]

[ . . . . .]

Huperzia_atp6 ATGGCTT------------------------------------------- [7]

Huperzia_atp6_183913_183495 .......------------------------------------------- [7]

Isoetes .C.....------------------------------------------- [7]

Vitis ...T.GCCGACAGAAATAGGCACCTACGTAAGTAGCTTGGGACTGTGCAA [50]

Zea_perennis_atp6_1 ...ATGATGATGACTAGATGGAGTTCCACTGATA---------TGAAGAG [41]

Cycas -------------------------------------------------- [0]

Megaceros .C.....------------------------------------------- [7]

Phaeoceros .C.....------------------------------------------- [7]

Anomodon .......------------------------------------------- [7]

Physcomitrella .......------------------------------------------- [7]

Treubia .....G.------------------------------------------- [7]

Marchantia .....G.------------------------------------------- [7]

[ 60 70 80 90 100]

[ . . . . .]

Huperzia_atp6 -------------------------------------------------- [7]

Huperzia_atp6_183913_183495 -------------------------------------------------- [7]

Isoetes -------------------------------------------------- [7]

Vitis ATCTATCAGTACTTTGATTTTTATCCGAGCCGCATGGATTCCTGATCTAG [100]

Zea_perennis_atp6_1 AAGAAATAGAATATTGGCTAATATG------GTGCCAATTCGTAATTTAA [85]

Cycas -------------------------------------------------- [0]

Megaceros -------------------------------------------------- [7]

Phaeoceros -------------------------------------------------- [7]

Anomodon -------------------------------------------------- [7]

Physcomitrella -------------------------------------------------- [7]

Treubia -------------------------------------------------- [7]

Marchantia -------------------------------------------------- [7]

[ 110 120 130 140 150]

[ . . . . .]

Huperzia_atp6 -------------------------------------------------- [7]

Huperzia_atp6_183913_183495 -------------------------------------------------- [7]

Isoetes -------------------------------------------------- [7]

Vitis ATCAAACTAATTACCGCGAAAAGATTTCTGAGATTGGTGCCGAGACTCTC [150]

Zea_perennis_atp6_1 GTTTACCTGATTATTATGAAT--ATGAAGAAGAATACCATCCAGTTTCAA [133]

Cycas -------------------------------------------------- [0]

Megaceros -------------------------------------------------- [7]

Phaeoceros -------------------------------------------------- [7]

Anomodon -------------------------------------------------- [7]

Physcomitrella -------------------------------------------------- [7]

Treubia -------------------------------------------------- [7]

Marchantia -------------------------------------------------- [7]

[ 160 170 180 190 200]

[ . . . . .]

Huperzia_atp6 -------------------------------------------------- [7]

Huperzia_atp6_183913_183495 -------------------------------------------------- [7]

Isoetes -------------------------------------------------- [7]

Vitis AGGGGACAACT-------------------CTCTGATAGATTCGAATTTC [181]

Zea_perennis_atp6_1 GAGAGGCAACCAGAGGGGTCTGTATACTCCTACGAATAGACAGATATTTA [183]

Cycas -------------------------------------------------- [0]

Megaceros -------------------------------------------------- [7]

Phaeoceros -------------------------------------------------- [7]

Anomodon -------------------------------------------------- [7]

Physcomitrella -------------------------------------------------- [7]

Treubia -------------------------------------------------- [7]

Marchantia -------------------------------------------------- [7]

[ 210 220 230 240 250]

[ . . . . .]

Huperzia_atp6 -------------------------------------------------- [7]

Huperzia_atp6_183913_183495 -------------------------------------------------- [7]

Isoetes -------------------------------------------------- [7]

Vitis TACTCAAAGAGAAGGGAC-TTCAACAC-------------------TTGC [211]

Zea_perennis_atp6_1 TCTTCAATTGGAAGGAGCATTCAAGACCGTGAGGTTCTACGCGATTTCCG [233]

Cycas -------------------------------------------------- [0]

Megaceros -------------------------------------------------- [7]

Phaeoceros -------------------------------------------------- [7]

Anomodon -------------------------------------------------- [7]

Physcomitrella -------------------------------------------------- [7]

Treubia -------------------------------------------------- [7]

Marchantia -------------------------------------------------- [7]

[ 260 270 280 290 300]

[ . . . . .]

Huperzia_atp6 -------------------------------------------------- [7]

Huperzia_atp6_183913_183495 -------------------------------------------------- [7]

Isoetes -------------------------------------------------- [7]

Vitis CTGAAGGCTATACAATTCCGTACGTGATGAATCAGGTCCATAACAACAAC [261]

Zea_perennis_atp6_1 CCAACGGTTACTCTTTCCCCAACGCGAGG--CTGGGTACAGCTTTTCCGA [281]

Cycas -------------------------------------------------- [0]

Megaceros -------------------------------------------------- [7]

Phaeoceros -------------------------------------------------- [7]

Anomodon -------------------------------------------------- [7]

Physcomitrella -------------------------------------------------- [7]

Treubia -------------------------------------------------- [7]

Marchantia -------------------------------------------------- [7]

[ 310 320 330 340 350]

[ . . . . .]

Huperzia_atp6 -------------------------------------------------- [7]

Huperzia_atp6_183913_183495 -------------------------------------------------- [7]

Isoetes -------------------------------------------------- [7]

Vitis AAGAC-CAATGATATACCCACACTAAAGG--GAATCTGGGAAAATGTG-- [306]

Zea_perennis_atp6_1 AATATATGATGATATACGAGCGCATGGGGTAGAAGCAAGTCGATTGGGTC [331]

Cycas -------------------------------------------------- [0]

Megaceros -------------------------------------------------- [7]

Phaeoceros -------------------------------------------------- [7]

Anomodon -------------------------------------------------- [7]

Physcomitrella -------------------------------------------------- [7]

Treubia -------------------------------------------------- [7]

Marchantia -------------------------------------------------- [7]

[ 360 370 380 390 400]

[ . . . . .]

Huperzia_atp6 -------------------------------------------------- [7]

Huperzia_atp6_183913_183495 -------------------------------------------------- [7]

Isoetes -------------------------------------------------- [7]

Vitis AATATCTATGGGCTTCATA-------------------GTCAATATTATC [337]

Zea_perennis_atp6_1 AGCCTCTAAGAGATCTGTACGATGAGATGGAAAGGAACGGCGAGATAGTA [381]

Cycas ----------------AT--------------------GCCAA------- [7]

Megaceros -------------------------------------------------- [7]

Phaeoceros -------------------------------------------------- [7]

Anomodon -------------------------------------------------- [7]

Physcomitrella -------------------------------------------------- [7]

Treubia -------------------------------------------------- [7]

Marchantia -------------------------------------------------- [7]

[ 410 420 430 440 450]

[ . . . . .]

Huperzia_atp6 ----------------------------------------------GGAG [11]

Huperzia_atp6_183913_183495 ----------------------------------------------.... [11]

Isoetes ----------------------------------------------.... [11]

Vitis AAGAAGCGCTCGA-CATAATGGATGATTTGTGGGGCGCGAGAACCCAC.. [386]

Zea_perennis_atp6_1 AATAACGGCTCAATCATTATCCCTGGAGGCGGCGGACCAGTAACAGAA.. [431]

Cycas ----------------------------------------------AC.. [11]

Megaceros ----------------------------------------------.C.. [11]

Phaeoceros ----------------------------------------------.C.. [11]

Anomodon ----------------------------------------------.C.. [11]

Physcomitrella ----------------------------------------------.C.. [11]

Treubia ----------------------------------------------.C.. [11]

Marchantia ----------------------------------------------.C.. [11]

[ 460 470 480 490 500]

[ . . . . .]

Huperzia_atp6 TCCACTAGAACAATTTGCCATTATTTCATTGATTCC---TATTCATATAG [58]

Huperzia_atp6_183913_183495 ...........................C.C......---..........T [58]

Isoetes .....C....T.........C......G.C...A..---...C....... [58]

Vitis C..C..T..G......T.....C.CC..........---...GA...... [433]

Zea_perennis_atp6_1 C...T.G..T......T.....CACC..---....TGGA.C.GA....T. [478]

Cycas C.....T..G..............CC..........---...GG...... [58]

Megaceros .....C............A......C..........---........... [58]

Phaeoceros .....C............A......C..........---........... [58]

Anomodon ...G..............A......C..........---........... [58]

Physcomitrella ...G..............A......C..........---........... [58]

Treubia ...G..............A......CA.........---........... [58]

Marchantia ...G..............A......CA.........---........... [58]

[ 510 520 530 540 550]

[ . . . . .]

Huperzia_atp6 GAAACTTGTATCTTTCATTTACCAATTCATCTTTGTTTATGCCACTAACT [108]

Huperzia_atp6_183913_183495 A.....C..C.........C.......T..............T..CG... [108]

Isoetes ...........T...TG.C.........CC...C........T..C...C [108]

Vitis ...........T.C.....C..A...C...............TG...... [483]

Zea_perennis_atp6_1 .C..G.AC...G.C.....C..A...CT...C....C.....T...C... [528]

Cycas ...........T.C.....C..A...C.........CC.....G...... [108]

Megaceros ...........TC.C...C..................C.......C.... [108]

Phaeoceros ...........TC.CT..C..T........T......C............ [108]

Anomodon ....T..C..................................T.T..... [108]

Physcomitrella ....T..C..................................T.T..... [108]

Treubia ...........T.............C..C.............T....... [108]

Marchantia ...........T.............C..C.............T....... [108]

[ 560 570 580 590 600]

[ . . . . .]

Huperzia_atp6 ATCAATTTAGTATTGCTTTTAGTCAATTTTGTCACCCG---AAATGGAGG [155]

Huperzia_atp6_183913_183495 C.T...............CG...T.............CAAG...A.TGT. [158]

Isoetes ......A...........C....TC...C.......T---G......G.. [155]

Vitis C...G...G..CC.A...C.G..TC.......T..TAAAAACGGA..... [533]

Zea_perennis_atp6_1 C..GG...G..CC.A...C.G..TTT.G....T..GAAAAA.GGA..G.. [578]

Cycas ....G..C...CC...........C..C....T..TAAGAACGGA..G.. [158]

Megaceros C...G.C...........C....TT....C.......C---G........ [155]

Phaeoceros ....G.C...........C....TT............C---G........ [155]

Anomodon ....G.C...........C....TC....C..T..TTT---......... [155]

Physcomitrella ....G.C...........C....TC....C.....TTT---......... [155]

Treubia ...GG.......C..........TC............T---G........ [155]

Marchantia ....G..................TC............T---G........ [155]

[ 610 620 630 640 650]

[ . . . . .]

Huperzia_atp6 ACAC--TTAGTACCAAATGCATGGCAATCCTTGGTGGAAATGATTTATGA [203]

Huperzia_atp6_183913_183495 .T..AT..TA.............A..T.TTC--------......C.... [200]

Isoetes .T..--.......T.......C.....C.................C.C.. [203]

Vitis .A..--CC............T..............A..GT.T...C.... [581]

Zea_perennis_atp6_1 .A.G--.C...G..........TT..............GC.T........ [626]

Cycas .A..--.C............T.........C....A..GC.T........ [206]

Megaceros ....--.......T......T......CT...C................. [203]

Phaeoceros ....--......TT......T.......T...C................. [203]

Anomodon ....--..............T...........T..T.....T........ [203]

Physcomitrella ....--..............T...........T........T........ [203]

Treubia ....--..............T...........T................. [203]

Marchantia .A..--..............T...........T................. [203]

[ 660 670 680 690 700]

[ . . . . .]

Huperzia_atp6 TTTTGTGCT---TAACTTGGTGAACGAACAAATAAGTGGTGCTTCTTCG- [249]

Huperzia_atp6_183913_183495 ........C---............T...............A...A.C..- [246]

Isoetes .CC.....CGCGC....C..........T...C.........A...C..- [252]

Vitis ...C....CG---...CC...A...........TG.....CT...CGGAA [628]

Zea_perennis_atp6_1 ...C....CG---...C....A............G.....CT...CGGAA [673]

Cycas ...C....CG---...CC................G.....CT...CGGAA [253]

Megaceros .......T.---................T...........C......TA- [249]

Phaeoceros .......T.---................T...........C......TA- [249]

Anomodon .........---.........A..T...........C...C........- [249]

Physcomitrella .........---.........A..T...........C...C........- [249]

Treubia .........---................................T....- [249]

Marchantia .........---.....................................- [249]

[ 710 720 730 740 750]

[ . . . . .]

Huperzia_atp6 --GTGAAACAACGGTTTTTTCCTCTGATCTTTGTCACTTTTACTTTTTTA [297]

Huperzia_atp6_183913_183495 --..AG.......AG.....TTC.........................C. [294]

Isoetes --...........A..C.C...C......CC.......CCC....CC... [300]

Vitis AT..T......AA......C....GC....CG................CG [678]

Zea_perennis_atp6_1 AT........CAA......C...TGC....CG................CG [723]

Cycas AT..T......AA....CCC....GC....CG.............C..CG [303]

Megaceros --......T...........TTGTC...A...........C....G.C.. [297]

Phaeoceros --......T.......C...T.GT....A...........C....G.C.. [297]

Anomodon --A.A........C...........A..T.................C... [297]

Physcomitrella --A..........C........C..A........................ [297]

Treubia --.................C...T......A......C........C... [297]

Marchantia --.................C..CT.A....A......C............ [297]

[ 760 770 780 790 800]

[ . . . . .]

Huperzia_atp6 TTATTTTGTAATCTTATCGGTATGATACCATATAGTTTTACAGTAACAAG [347]

Huperzia_atp6_183913_183495 ...CAAA......C.....A.......T..C.......G........... [344]

Isoetes .C....C...............C.............C............. [350]

Vitis ......C......CCCAG...........T.....C..C.....T..... [728]

Zea_perennis_atp6_1 ......C......CCCAG...........C.T...C..C.....G..... [773]

Cycas ......C......CCCAG.................C.CC.....T..... [353]

Megaceros CC....C.....T................T.................... [347]

Phaeoceros ......C.....T..............TTT................T... [347]

Anomodon ......A..........T...........T.................... [347]

Physcomitrella ..............C..T...........C.................... [347]

Treubia .................................................. [347]

Marchantia .................T................................ [347]

[ 810 820 830 840 850]

[ . . . . .]

Huperzia_atp6 TCATTTTATAATTACTTTGGGTCTTTCATTATCTCTCTTTATTGGAATAA [397]

Huperzia_atp6_183913_183495 .......G.C......C......C..T.......TCTCC.---....... [391]

Isoetes .....C..........C..................CTCC........... [400]

Vitis .......C.C......C.T.....C.....T...A.T........C..T. [778]

Zea_perennis_atp6_1 .......C.C..........C.........T..CA.T.....A..C..T. [823]

Cycas ......C..C.............CCC....TC.GA.T.C.........T. [403]

Megaceros .......TC.T....C.........C....C....CT.C.TC.....CG. [397]

Phaeoceros .T.....T..T....C.........CT...C....CT.C.CC......G. [397]

Anomodon ...............CC...................T.....C.....C. [397]

Physcomitrella ...............CC.........................C.....C. [397]

Treubia ......C........CC.A...........T.....T............. [397]

Marchantia ...............CC.A...........C.....T............. [397]

[ 860 870 880 890 900]

[ . . . . .]

Huperzia_atp6 CTATAGTTGGATTCCAAACACATGGGCTTCATTTTTTCAGTTTCTTATTA [447]

Huperzia_atp6_183913_183495 T.G-....................C....--------------------- [419]

Isoetes .....A.C....CTTG.GA......A...............CC.CC.CC. [450]

Vitis .......G.....T....G.A................A..C....C.... [828]

Zea_perennis_atp6_1 .G..C........T....G..................T..C......... [873]

Cycas .......G....CT....G.A.............C.C...C.C..C..C. [453]

Megaceros ..T..CC......T....T...........................C... [447]

Phaeoceros ..T..CC.......A...T...........................T... [447]

Anomodon ............C........................T.....T..C..G [447]

Physcomitrella .............T.......................T.....T.....G [447]

Treubia .............T..............C..............A...... [447]

Marchantia .............T..............C............A........ [447]

[ 910 920 930 940 950]

[ . . . . .]

Huperzia_atp6 CCTCAAGGAGTACCCTTGGCGTTAGCACCTTTCCTAGTACTTCTTGAGCT [497]

Huperzia_atp6_183913_183495 -------------------------------------------------- [419]

Isoetes ........G..C.................C..TT.........C.....C [500]

Vitis ..CGC......C..AC..C.............TT.......C........ [878]

Zea_perennis_atp6_1 ..AGCG.....C..AC..C.A...........TT.......C........ [923]

Cycas ..CGC.........AC..C..............TC......C........ [503]

Megaceros ....G.......G.A...C.......T......T.....A.G..C....C [497]

Phaeoceros ....G.......G.A...C.......T......T.....A.G..C....C [497]

Anomodon ..G...............C..........C..............C..AT. [497]

Physcomitrella ..G...............C..........C.................... [497]

Treubia ..G............C..C..............T................ [497]

Marchantia ..G...............C..............T................ [497]

[ 960 970 980 990 1000]

[ . . . . .]

Huperzia_atp6 AATCTCTTATTGTTTTCGCGCATTAAGCTCAGGAATACGTTCATTTGCCA [547]

Huperzia_atp6_183913_183495 -------------------------------------------------- [419]

Isoetes ...TC.CC..C.....T......C....C........T......CC.... [550]

Vitis ....C..C.................................T......T. [928]

Zea_perennis_atp6_1 .......C..........T......................T......T. [973]

Cycas ....C..C......C........C.................T......T. [553]

Megaceros GT.TC..C..A..C...............T...T.......T..CC.... [547]

Phaeoceros GT.TC..C..A..C......T........T.......T...T...C.... [547]

Anomodon ..........C................TCT...........T...C.... [547]

Physcomitrella ......C....................TCT...........T........ [547]

Treubia .........CG..................T...........T........ [547]

Marchantia .............................T...........T........ [547]

[ 1010 1020 1030 1040 1050]

[ . . . . .]

Huperzia_atp6 ATATGATGGCTGGTCATAGTTTAGTCAAGATTTCAAGTGGCTTTGCTTGG [597]

Huperzia_atp6_183913_183495 -------------------------------------------------- [419]

Isoetes ..............T......C..........C........CC...C... [600]

Vitis ..........C..........C...A.......T......G..C...... [978]

Zea_perennis_atp6_1 ..........C..........C...A.......T......G......... [1023]

Cycas .....................C...A.....C........G.CC...C.. [603]

Megaceros .....................C...G..............G......C.. [597]

Phaeoceros .....................C...G..............G......C.. [597]

Anomodon .........................A......CT...C..G......... [597]

Physcomitrella ................................CT...C..G......... [597]

Treubia .................................T...C..G......... [597]

Marchantia .................................T...C..G......... [597]

[ 1060 1070 1080 1090 1100]

[ . . . . .]

Huperzia_atp6 ACTATGCTATCTATGGGGGGTATTATGTATTT---AGCATATCTAGCTCC [644]

Huperzia_atp6_183913_183495 -------------------------------------------------- [419]

Isoetes ..C.C.TC.C.......A.............C---G..G....C...... [647]

Vitis ..........G....AAT.A.C..T.A.....CAT..GGG...CT.G... [1028]

Zea_perennis_atp6_1 ..........T.C..AATAA....T.C.....CTT..G.G....T.G... [1073]

Cycas ..........G....AATAA...A........CAT..G.G....T.G... [653]

Megaceros ................................---....C....T..C.. [644]

Phaeoceros ................................---....C....T...TT [644]

Anomodon ................................---...GC....T..... [644]

Physcomitrella ................................---...CC....T..... [644]

Treubia ...........G.........C..C.......---...GC.G..T..... [644]

Marchantia ...........C.....T......C.......---..GGC.G..T..... [644]

[ 1110 1120 1130 1140 1150]

[ . . . . .]

Huperzia_atp6 TTCTTCGATAGTATTCGCATTAACTGGTTTAGAATTAGGTGTTGCTATAT [694]

Huperzia_atp6_183913_183495 -------------------------------------------------- [419]

Isoetes ...CC......C..C...G.C...A....C.....C.....CA.T..... [697]

Vitis ..T..TT.....TC.T........C...CCG...........A....... [1078]

Zea_perennis_atp6_1 C.TA.TT.....TC.A........C...C.G...........A....... [1123]

Cycas ...A..A.....T..T...CCG..C....CG...........A....... [703]

Megaceros .CT..T.G............C.....................C.G.G... [694]

Phaeoceros ..T.CT.G.........T..C........C............C.G..... [694]

Anomodon ..T..TA.......................G................... [694]

Physcomitrella ..T..T........................G................... [694]

Treubia A.T.CTT....................G.................C..TC [694]

Marchantia C.T..TT......................................C..TC [694]

[ 1160 1170 1180 1190 1200]

[ . . . . .]

Huperzia_atp6 TACAAGCTTATGTTTCTACTATTTCAATTTGTATTTACTCAAATGATGCT [744]

Huperzia_atp6_183913_183495 -------------------------------------------------- [419]

Isoetes C.T.....C...C.C...T..C.......C.................... [747]

Vitis C.......C..........G..C.....C..........TG......... [1128]

Zea_perennis_atp6_1 C.......C..........G..C................TG......... [1173]

Cycas C.......C..........A..C..........C......G......... [753]

Megaceros C.......C.....C......C.C...A.C.C.C....CTC......... [744]

Phaeoceros C.T...T..............C.C...A...C.C.....TC......... [744]

Anomodon ...............T........T...C..........T.......... [744]

Physcomitrella ...............T........T..............T.......... [744]

Treubia .C..G..........TC..A....TGC.A.........CT...C..G... [744]

Marchantia .C..G..........TC..A....TGC.A.........CT.......... [744]

[ 1210 1220 1230 1240 1250]

[ . . . . .]

Huperzia_atp6 ATAAACCTTCATTAA----------------------------------- [759]

Huperzia_atp6_183913_183495 -------------------------------------------------- [419]

Isoetes ............CG.----------------------------------- [762]

Vitis .C...T..C...C..AGGTGGTTCTTTATTTATAATTGA----------- [1167]

Zea_perennis_atp6_1 .C...T..C...C..AATGAGTCATT---TCATAATTGCATAAAAACGAG [1220]

Cycas .C...T.C....CG.----------------------------------- [768]

Megaceros ...............----------------------------------- [759]

Phaeoceros ...............----------------------------------- [759]

Anomodon ...............----------------------------------- [759]

Physcomitrella ...............----------------------------------- [759]

Treubia ........C......----------------------------------- [759]

Marchantia ...............----------------------------------- [759]

[ 1260]

[ . ]

Huperzia_atp6 ------------- [759]

Huperzia_atp6_183913_183495 ------------- [419]

Isoetes ------------- [762]

Vitis ------------- [1167]

Zea_perennis_atp6_1 GAGCCAATCATAG [1233]

Cycas ------------- [768]

Megaceros ------------- [759]

Phaeoceros ------------- [759]

Anomodon ------------- [759]

Physcomitrella ------------- [759]

Treubia ------------- [759]

Marchantia ------------- [759]

;

END;

**atp8**

#NEXUS

[MacClade 4.05 registered to Yin-Long Qiu Lab, EEB, University of Michigan]

BEGIN DATA;

DIMENSIONS NTAX=14 NCHAR=606;

FORMAT DATATYPE=DNA MISSING=? GAP=- MATCHCHAR=. INTERLEAVE ;

MATRIX

[ 10 20 30 40 50]

[ . . . . .]

Huperzia_atp8 ---ATGCCTCAACTAGATCAATTTACGTATTTGACGCAATTTGTTTGGTT [47]

Huperzia_atp8_57764_57329 -------------................................C.... [37]

Huperzia_atp8_333789_334235 -----------..................................C.... [39]

Huperzia_atp8_79339_79153 -------------------------------------------------- [0]

Huperzia_atp8_399636_399714 -------------------------------------------------- [0]

Isoetes ---.C........CG.................C............C...C [47]

Vitis ---...........G...A....C..T.....C..A.....CT.C....C [47]

Cycas ATG..T.......CG...A....C..T....CC..A.....CT.CC.... [50]

Oryza_NC_011033 ---...........T...A....G..T.....CT.A.....CT.C..... [47]

Anomodon ---.......................C....................... [47]

Physcomitrella ---............................................... [47]

Pleurozia ---................................A.....C........ [47]

Marchantia ---................................A.....C........ [47]

Treubia ---................................A.....C........ [47]

[ 60 70 80 90 100]

[ . . . . .]

Huperzia_atp8 ATGTGTGTTTTATATGAGTTTTTATGTTTTATTATATAACGAGG------ [91]

Huperzia_atp8_57764_57329 .................C....G......C..C.C....T..T.------ [81]

Huperzia_atp8_333789_334235 .................C....G......C..C.C....T..T.------ [83]

Huperzia_atp8_79339_79153 -------------------------------------------------- [0]

Huperzia_atp8_399636_399714 -------------------------------------------------- [0]

Isoetes .C.....CC.C......C.CCCC......C...G.....TA.T.------ [91]

Vitis ...CC.T..CCTCT.T.C...C...A..CCCA...GC..T..T.GAGATG [97]

Cycas ...CC..A.C.TCT.C.C..C....A..CCCCC..GC..T..T.------ [94]

Oryza_NC_011033 ...CC.T..CCTCT.T.........A..C.C....TA..TA.TAATAATG [97]

Anomodon ......C.........GCC........A...........T..T.------ [91]

Physcomitrella ......T.........GCC........A...........T..T.------ [91]

Pleurozia ...C.............C.........A...........T..T.------ [91]

Marchantia ...C.............C.........A...........T..T.------ [91]

Treubia .................C.........A..........GT..T.------ [91]

[ 110 120 130 140 150]

[ . . . . .]

Huperzia_atp8 GATTACCCAAAATAAGTCGAATTCTAAAACC--ACGAAAACAACTGGTTT [139]

Huperzia_atp8_57764_57329 .......TTTT.C............CTCT.T--.............A... [129]

Huperzia_atp8_333789_334235 .......TTTT.C............CTCT.TCT..........T..A... [133]

Huperzia_atp8_79339_79153 -------------------------------------------------- [0]

Huperzia_atp8_399636_399714 -------------------------------------------------- [0]

Isoetes ...C...T.G..C......G....CC.....--.A.G...A.G.AAC... [139]

Vitis ..G...TTGGG..C..CA............T--...G..C.......... [145]

Cycas ..G...TT.GG..C..CA......CC.....--...G..C.........C [142]

Oryza_NC_011033 ..A...TTGG...T..CA.......C....T--...G..C......C... [145]

Anomodon ...................C.....C...TT--.........G...A... [139]

Physcomitrella ................C..C.....C...TT--.........G...A... [139]

Pleurozia .......................A.C....T--.A......G......A. [139]

Marchantia .......................A.C....T--.A......G......A. [139]

Treubia .............G.........A.C....T--.A.G....G...A..A. [139]

[ 160 170 180 190 200]

[ . . . . .]

Huperzia_atp8 TGCCTTCTCCAAAA--CGG-GACGTTGAGCAAAGCATTTACAGTTTGGAA [186]

Huperzia_atp8_57764_57329 C...-----..TTG--..C-T..C.CA.-------...GG..AC.CC--. [162]

Huperzia_atp8_333789_334235 C...-----..TTG--..C-T..C.CA.-------...GG..AC.CC--. [166]

Huperzia_atp8_79339_79153 -------------------------------------------------- [0]

Huperzia_atp8_399636_399714 -------------------------------------------------- [0]

Isoetes C..ACCT.ATGGGGAT..AGAGT..C..AT...-..A.C.T...CCCT-. [187]

Vitis CA.ACC------GG---..GA..AACATC.GG....ACG..----CCC.. [182]

Cycas C..ACCAA.G..GG---..GA..AACATC.GG....ACG..----CCC.. [185]

Oryza_NC_011033 C..ACC------GG---..GA..AAGATC.GTTT..AGG..----CCT.. [182]

Anomodon CA.A.----..GG.--T.TA.G.ACA....CG...GA.......G.T... [183]

Physcomitrella C..A.----.....--T.TA.G.ACA....CG...GA.......G.T... [183]

Pleurozia C..AG----G....--A.TA.G..CGA.........A.G..C..G.A... [183]

Marchantia C..AG----G....--A.TA.G..CG.....G....A.G..C..G.A... [183]

Treubia C..AG----A.G..--A.TA.G..CA.....G....A.G..C..G.A... [183]

[ 210 220 230 240 250]

[ . . . . .]

Huperzia_atp8 CTTTTTTT----AGTTATTTTCAAAGAATGCTTTA-ACACCAGTGTATCC [231]

Huperzia_atp8_57764_57329 .......---GGC..GG.....------CC.....-...T.......... [202]

Huperzia_atp8_333789_334235 ........TCGGC..GG....T------CC.....-...T.......... [209]

Huperzia_atp8_79339_79153 -------------------------------..C.-...T.......... [18]

Huperzia_atp8_399636_399714 ------------------------------------------........ [8]

Isoetes .-------------.GGA..-----...G.T..C.CGTG..CA.A..... [219]

Vitis .AG...G---GA..A...C..G.G.A..G.T....-G....G........ [228]

Cycas .AG...G---GA..AG....CG.G.A..G.T....-G....G........ [231]

Oryza_NC_011033 GAA...G---GA..A...C.CG.G.A..G.T....-G....G..C.C..A [228]

Anomodon .AG-------GAT...G.................G-.T......A..... [225]

Physcomitrella .AG-------GAT...G.................G-.T......A..... [225]

Pleurozia .AG-------GAT..GG.............T...C-.AG......C.AA. [225]

Marchantia .AG-------GAT..GG.............T...C-.AG......C.AA. [225]

Treubia .AG-------GAT..GG.............T...C-.AGTA....C.AA. [225]

[ 260 270 280 290 300]

[ . . . . .]

Huperzia_atp8 TACCCGTACTCAAGTGTATCTGGAGCA---TCCAAGT---GGTGTAATTC [275]

Huperzia_atp8_57764_57329 C.T......C.................---.......---.........T [246]

Huperzia_atp8_333789_334235 C.T......C.................---.......---.........T [253]

Huperzia_atp8_79339_79153 C.T......C.................---.......---.........T [62]

Huperzia_atp8_399636_399714 ..T......C.........T.......---.....AAAAG.A.......T [55]

Isoetes ..TA.........---...TC......GTGC.....C---...T.C...T [263]

Vitis ..TAT......T...T...TC.A..T.---...C.A.---.......CG. [272]

Cycas C.TAT......T...TC..TC.A..T.---...C.A.---.......CG. [275]

Oryza_NC_011033 ..TAT......C...T....C.A..T.---...C.A.---.......GA. [272]

Anomodon ..T.T......GG..............---.......---.......CGA [269]

Physcomitrella ..T.T.......G..............---.......---.......CGA [269]

Pleurozia ..T.T...............C......---.......---.......AGG [269]

Marchantia ..T.T...............C......---.......---.......AGG [269]

Treubia ..T.T............C..C......---.......---.......AGG [269]

[ 310 320 330 340 350]

[ . . . . .]

Huperzia_atp8 CATGACAAAAAGCTTGAATGCTAAT--CAATGG-CCGCGATTGAATAAAT [322]

Huperzia_atp8_57764_57329 .C...----......A......C.C--TCGG..-..A..CAGA....... [289]

Huperzia_atp8_333789_334235 .C...----......A......C.C--TCGG..-..A..CA......... [296]

Huperzia_atp8_79339_79153 .C...----......A......C.C--TCGG..-..A..CAGA....... [105]

Huperzia_atp8_399636_399714 ...AGT.........A........-------------------------- [79]

Isoetes ....TT.G.......A...A.C...ACT...TAAT.AT..A....G.... [313]

Vitis .G.CG--------------A..T..--TGGGAAAAA.GAG.CA.....C. [306]

Cycas .G.CG--------------A..T..--TGGG.AAAA.GA.GA....C.C. [309]

Oryza_NC_011033 .G.CG--------------A...T.--TGGGAAAAA.GAGGAA...C.C. [306]

Anomodon A...GT.........A.........--....TA-AAA...C......... [316]

Physcomitrella A...GT.........A.........--....TA-AAA.A.A......... [316]

Pleurozia A...GTCC.GCT.GCA.........--A...T.-.AA...A........G [316]

Marchantia A...GTCC..CT.GCA......C..--A...T.-.AA...A........G [316]

Treubia A...GTCC..CT.GCA.........--A...T.-.AA...A......... [316]

[ 360 370 380 390 400]

[ . . . . .]

Huperzia_atp8 CTCATGT-GTGTT----------CCT----------TGGGAGAGATTA-G [350]

Huperzia_atp8_57764_57329 ..T....-.....----------TA.----------C.A..AG...C.C. [318]

Huperzia_atp8_333789_334235 ..T....-.....CCTCGGGTCTTA.GTGTGTTACTC....T......C. [345]

Huperzia_atp8_79339_79153 ..T....-.....----------TA.----------C.A..AG...C.C. [134]

Huperzia_atp8_399636_399714 -------------------------------------------------- [79]

Isoetes ..T...C-..A..----------.T.----------C...G..A....-. [341]

Vitis T.G..C.CT....-----------------------.C.....A..A.-. [332]

Cycas TAT..A.CC....-----------------------.C.....A..A.-. [335]

Oryza_NC_011033 ..G..C.CAGA..-----------------------.C.....A..A.-. [332]

Anomodon ..T....-A....----------...----------.A.....A....-. [344]

Physcomitrella ..T....-A....----------...----------.......A....-. [344]

Pleurozia A.T....-A....----------.T.----------............-. [344]

Marchantia A.T....-A....----------.T.----------............-. [344]

Treubia A.T....-A....----------GT.----------.A.........T-. [344]

[ 410 420 430 440 450]

[ . . . . .]

Huperzia_atp8 TATCTCACCAGTG--ATAAAAAAAAACACACTCT---CCACCATGG-GTC [394]

Huperzia_atp8_57764_57329 ...A.T.......--..C....G...........---.T....C.A-... [362]

Huperzia_atp8_333789_334235 .G.A.A.......TA..C....-.......--------..TTT.C.-... [385]

Huperzia_atp8_79339_79153 ...A.T......A--..-.G.........---------T.T.CA..-A.. [171]

Huperzia_atp8_399636_399714 -------------------------------------------------- [79]

Isoetes .G...........--..C.G..-..G...CTC.AG--......C..-... [385]

Vitis .GG.....G..GA--..GG...G.....T.T...ATTTG.T.TC.AA... [380]

Cycas .GG.....G..GA--..GG...GG....TT..A.ATCTG.TTTC.AA..A [383]

Oryza_NC_011033 .GG.....G..GA--..GG.G.G.C.G.TT....ATTTG.T.TC.AA... [380]

Anomodon .G......A....--............G....T.---.A.TT...A-... [388]

Physcomitrella .G......A...A--............G....T.---.A.TT...A-... [388]

Pleurozia .G.A....A....--..C.....G...G....T.---.G...T..A-... [388]

Marchantia .G.A....A....--..C.....G...G....T.---.A...T..A-... [388]

Treubia .G.A....A....--..C.GG......G....T.---.A...T..A-... [388]

[ 460 470 480 490 500]

[ . . . . .]

Huperzia_atp8 CTT-CGTATTATCAAACCGCTTATCT-AGCT--------AGGAGTAAAAC [434]

Huperzia_atp8_57764_57329 ...-.A.T...C......A...C.TG-....--------......C.... [402]

Huperzia_atp8_333789_334235 ...ATACT.CTCGT.C.T.T..C.TCT....--------......C...T [427]

Huperzia_atp8_79339_79153 ...-...G.A.......--------------------------------- [187]

Huperzia_atp8_399636_399714 -------------------------------------------------- [79]

Isoetes ...-------------.GAT..C.A.----.--------G....C---.. [407]

Vitis ..CATA.--------.G.A...C.TCC.ATCCT------G.AT.GGGG.T [416]

Cycas TCCATA.--------.G.A.....TCC.ATCCTATATCTG.AT.G.GG.T [425]

Oryza_NC_011033 ..CATA.--------.A.A...C.TCC..TC---------------GG.T [407]

Anomodon .C.CTAC-......T.TAA...C.T.-...G--------TCAC..CG... [428]

Physcomitrella .C.CTAC-......T.T.A...C.T.-...G--------TCAC..C.... [428]

Pleurozia ...CTAC-.........AA...C...-....--------TCAC..C.... [428]

Marchantia .C.CTAC-..........A...C...-....--------TCAC..C.... [428]

Treubia ...CTAC-.........TA.......-...C--------TCAC..C.... [428]

[ 510 520 530 540 550]

[ . . . . .]

Huperzia_atp8 CAGTGCTCCGAACAACAT---------CTATGTTTTACGCGGACGGAGGG [475]

Huperzia_atp8_57764_57329 ..CCTGAAA.........---------................------- [436]

Huperzia_atp8_333789_334235 ..CA..........C.C.---------..--------------------- [447]

Huperzia_atp8_79339_79153 -------------------------------------------------- [187]

Huperzia_atp8_399636_399714 -------------------------------------------------- [79]

Isoetes TGC..T........T...---------.C............C.TA..AA. [448]

Vitis ..C.TG.AG...TG....AATGCTAAT.C.....CC..A...C.AAG.AA [466]

Cycas TCC.TG.AG...TG....AATGCTGAT.C.........A...C.A.GTAA [475]

Oryza_NC_011033 ..C.TG.TG...A..A..AATGCTCACAC.....CC..A...G.AAG.AA [457]

Anomodon ..CA....TT.....A..---------.................AA...A [469]

Physcomitrella ..CA....TT.....A..---------.................AA...A [469]

Pleurozia ..CC....TT.....A..---------.................AA.A.A [469]

Marchantia ..CC....TT.....A..---------.................AA.A.A [469]

Treubia ...C....TT.....A..---------....T............AA.A.A [469]

[ 560 570 580 590 600]

[ . . . . .]

Huperzia_atp8 CACTTCAAAAAAATAAGTAA------------------------------ [495]

Huperzia_atp8_57764_57329 -------------------------------------------------- [436]

Huperzia_atp8_333789_334235 -------------------------------------------------- [447]

Huperzia_atp8_79339_79153 -------------------------------------------------- [187]

Huperzia_atp8_399636_399714 -------------------------------------------------- [79]

Isoetes ---C.GG..TGCGCGCTG.GGGGGTGCATACCGAAAACGAATAA------ [489]

Vitis G-----C.TCGTT.TTTA.------------------------------- [480]

Cycas A-----C.TCGTT.GTCA.------------------------------- [489]

Oryza_NC_011033 G-----C.T..TC.---A.------------------------------- [468]

Anomodon ACACC.T..TG....TCA.GAACGGACCAAGAAAAAAGAAAAATACGAAT [519]

Physcomitrella ACACC.T.GT.....TCA.GAACGGACAAAAAAAAAAGAAAAATACGAAT [519]

Pleurozia G.AC..TGGCG..G.TAA.GAACGGACCAAGAAAAAA-AAAAAT-----T [513]

Marchantia G.AC..T.GC...G.TAA.GAACGGACCAAGAAAAAA-AAAAAT-----T [513]

Treubia G.AC..TGGCG..G.TAA.GAACGGACCAAGAAAAAA-TAAAAT-----T [513]

[ ]

[ ]

Huperzia_atp8 ------ [495]

Huperzia_atp8_57764_57329 ------ [436]

Huperzia_atp8_333789_334235 ------ [447]

Huperzia_atp8_79339_79153 ------ [187]

Huperzia_atp8_399636_399714 ------ [79]

Isoetes ------ [489]

Vitis ------ [480]

Cycas ------ [489]

Oryza_NC_011033 ------ [468]

Anomodon GCGTAA [525]

Physcomitrella GCGTGA [525]

Pleurozia TCATGA [519]

Marchantia TCATGA [519]

Treubia TCGTGA [519]

;

END;

**cob**

#NEXUS

[MacClade 4.05 registered to Yin-Long Qiu Lab, EEB, University of Michigan]

BEGIN DATA;

DIMENSIONS NTAX=17 NCHAR=1179;

FORMAT DATATYPE=DNA MISSING=? GAP=- MATCHCHAR=. INTERLEAVE ;

MATRIX

[ 10 20 30 40 50]

[ . . . . .]

Huperzia_cob ATG---------GCCAGACGACTATCGATTCTTGAACAACCTATATTTTC [41]

Huperzia_cob_230202_229896 -------------------------------------------------- [0]

Huperzia_cob_142451_142637 -------------------------------------------------- [0]

Huperzia_cob_49245_49173 -------------------------------------------------- [0]

Huperzia_cob_30629_30854 -------------------------------------------------- [0]

Huperzia_cob_30906_31002 -------------------------------------------------- [0]

Isoetes ...---------..........C.........CA..T.....C.....C. [41]

Cycas ...ACTATAATAAAACA....T.CC.T....CC........C.....AC. [50]

Arabidopsis ...ACTATAAGGAA.CA....T.C..TC.....A............CC.. [50]

Zea_perennis ...ACTATAAGGAA.CA....T.C..TC.....A............AC.. [50]

Megaceros .C.---------..A........T.........A................ [41]

Phaeoceros .C.---------..A........T.........A..T...T......... [41]

Anomodon ...---------..A..................A................ [41]

Physcomitrella ...---------..A..................A................ [41]

Marchantia ...---------..A..................A................ [41]

Pleurozia ...---------..A..................A................ [41]

Treubia ...---------..A..................A................ [41]

[ 60 70 80 90 100]

[ . . . . .]

Huperzia_cob TACACTTAACCATCATTTGATAGATTATCCAACCCCGAGCAATTTAAGTT [91]

Huperzia_cob_230202_229896 -------------------------------------------------- [0]

Huperzia_cob_142451_142637 -------------------------------------------------- [0]

Huperzia_cob_49245_49173 -------------------------------------------------- [0]

Huperzia_cob_30629_30854 -------------------------------------------------- [0]

Huperzia_cob_30906_31002 -------------------------------------------------- [0]

Isoetes .........................C...TG..T.T.............. [91]

Cycas C...........G............C.................A...... [100]

Arabidopsis C........T..G.....AG.......................C.T.... [100]

Zea_perennis C...........G.....A........................C.T.... [100]

Megaceros ....G.....A......C......C........T.TA............. [91]

Phaeoceros ....G.....A..T..........C...TT..TTT.A............. [91]

Anomodon ....T.....A.......A.................A......A...... [91]

Physcomitrella ....T.....A................................A...... [91]

Marchantia ....T.....A......................T.........A...... [91]

Pleurozia ..........A......................T..T......A...... [91]

Treubia ..........A.......A..............T.........A...... [91]

[ 110 120 130 140 150]

[ . . . . .]

Huperzia_cob ATTGGTGGGGCTTTGGTTCGTTAGCAGGTATTTGTTTAGTTATTTAGATA [141]

Huperzia_cob_230202_229896 -------------------------------------------------- [0]

Huperzia_cob_142451_142637 -------------------------------------------------- [0]

Huperzia_cob_49245_49173 -------------------------------------------------- [0]

Huperzia_cob_30629_30854 -------------------------------------------------- [0]

Huperzia_cob_30906_31002 -------------------------------------------------- [0]

Isoetes ..C..C...T.C.....CT.....TT...CC..T..C...C......... [141]

Cycas ..........G..............T......C...C...C...C..... [150]

Arabidopsis ..........G..C...C.......T..............C...C..... [150]

Zea_perennis ..........G..C...........T..............C...C..... [150]

Megaceros ..........T...........G..T.C....C....G....C....... [141]

Phaeoceros ..........T.......T...G..T.C....C....G............ [141]

Anomodon ........A.T..C........G..T...C....C..GT.C...C..... [141]

Physcomitrella ........A.T...........G..T...C....C..GT.C...C..... [141]

Marchantia ..........T...........G..T...C.......G.....CC.A... [141]

Pleurozia ..........T...........G..T...C.......G.....CC.A... [141]

Treubia ..........T...........G..T...C.......GA....CC.A... [141]

[ 160 170 180 190 200]

[ . . . . .]

Huperzia_cob ATTACAGGCGTTTTCTTAGCTATGCATTATACACCTCATGTGGATCTTGC [191]

Huperzia_cob_230202_229896 -------------------------------------------------- [0]

Huperzia_cob_142451_142637 -------------------------------------------------- [0]

Huperzia_cob_49245_49173 -------------------------------------------------- [0]

Huperzia_cob_30629_30854 -------------------------------------------------- [0]

Huperzia_cob_30906_31002 -------------------------------------------------- [0]

Isoetes ....T...A.....TC...........C..................CA.. [191]

Cycas G.G..C.......CTCC............CG................A.. [200]

Arabidopsis G.G..T........T..............C...............T.A.. [200]

Zea_perennis G.G..T........T..............C.................A.. [200]

Megaceros ........T....C.............C...................A.. [191]

Phaeoceros ........T....C..C.......T.........T............A.. [191]

Anomodon ..............T.................G............T.A.. [191]

Physcomitrella ..............T.................G............T.A.. [191]

Marchantia C.......T....................................T.A.. [191]

Pleurozia C.......T....................................T.A.. [191]

Treubia C.......T......................................A.. [191]

[ 210 220 230 240 250]

[ . . . . .]

Huperzia_cob TTTCTTAAGCGTAGAACACATAATGAGAGATGTGAAGGGAGGCTGGTGGC [241]

Huperzia_cob_230202_229896 -------------------------------------------------- [0]

Huperzia_cob_142451_142637 -------------------------------------------------- [0]

Huperzia_cob_49245_49173 -------------------------------------------------- [0]

Huperzia_cob_30629_30854 -------------------------------------------------- [0]

Huperzia_cob_30906_31002 -------------------------------------------------- [0]

Isoetes .....C..............................A............. [241]

Cycas ....AAC.....G........C...........TG.A..G...C...T.. [250]

Arabidopsis ....AAC..............T...........TG.A..G.......T.. [250]

Zea_perennis ....AAC..............T...........TG.A..G.......T.. [250]

Megaceros .........T..........................A..........TA. [241]

Phaeoceros .........T........T..............T..A..........TA. [241]

Anomodon ...T.................C..............A..C.......T.. [241]

Physcomitrella .....................C..............A..C.......T.. [241]

Marchantia .....................C..............A..........T.. [241]

Pleurozia .....C...............C..............A..........T.. [241]

Treubia ...T.C...............C...........T..A.........CT.. [241]

[ 260 270 280 290 300]

[ . . . . .]

Huperzia_cob TTCGTTATATGCATGCTAATGGGGCCAGTATGTTTTTCATTGTGGTTTAT [291]

Huperzia_cob_230202_229896 -------------------------------------------------- [0]

Huperzia_cob_142451_142637 -------------------------------------------------- [0]

Huperzia_cob_49245_49173 -------------------------------------------------- [0]

Huperzia_cob_30629_30854 -------------------------------------------------- [0]

Huperzia_cob_30906_31002 -------------------------------------------------- [0]

Isoetes .C...C.......................CC................C.. [291]

Cycas .........................A.......................C [300]

Arabidopsis .C.......................A.........C.T...........C [300]

Zea_perennis .C.......................A.........C...........C.C [300]

Megaceros .......C..............T...........C..T...AC....... [291]

Phaeoceros ..T....C...T..........T.TA........CC.T...A........ [291]

Anomodon .........................A...........T............ [291]

Physcomitrella .........................A...........T............ [291]

Marchantia ......................A..............T............ [291]

Pleurozia ......................A..............T.....C...... [291]

Treubia ......................A..............T............ [291]

[ 310 320 330 340 350]

[ . . . . .]

Huperzia_cob CTTCATATTTTTCGTGGTTTATACTACGGGAGTTATGCGAGTCCTAGGGA [341]

Huperzia_cob_230202_229896 -------------------------------------------------- [0]

Huperzia_cob_142451_142637 -------------------------------------------------- [0]

Huperzia_cob_49245_49173 -------------------------------------------------- [0]

Huperzia_cob_30629_30854 ------------------------------------.....AAG..T..G [14]

Huperzia_cob_30906_31002 -------------------------------------------------- [0]

Isoetes TC.T.....C..T.C....C.C.TC.T................T...... [341]

Cycas ..C.......C........C...TC.T.C....C..AGC........... [350]

Arabidopsis ..................C....TC.T.C.......AGC........... [350]

Zea_perennis ..................C....TC.T.C.......AGC........... [350]

Megaceros .........C.............T..T..A.................... [341]

Phaeoceros ...T........T..........T..T..A.................... [341]

Anomodon ..................C....T..T..A..C...T............. [341]

Physcomitrella ..................C....T..T..A......T............. [341]

Marchantia ......T................T..T..A.................A.. [341]

Pleurozia ..C...T................T..T..A.................A.. [341]

Treubia ..C...T................T.....A..............C..A.. [341]

[ 360 370 380 390 400]

[ . . . . .]

Huperzia_cob ATTAGTTTGGTGTATTGGAGTGATCATTTTGTTACTAATGATCGTCACAG [391]

Huperzia_cob_230202_229896 -------------------------------------------------- [0]

Huperzia_cob_142451_142637 -------------------------------------------------- [0]

Huperzia_cob_49245_49173 -------------------------------------------------- [0]

Huperzia_cob_30629_30854 ..AT...........G......G.....-.A...T..G..........C. [63]

Huperzia_cob_30906_31002 -------------------------------------------------- [0]

Isoetes ..C....C............C.G.....C.C.C.TC.......A.A.... [391]

Cycas ...C...C....CC.C.....TG....C..CC..T.......T..G.... [400]

Arabidopsis ...T.........C.......TG.A..C..CC..T.......T..G.... [400]

Zea_perennis ...T.........C.C.....TG....A..CC..T.......T..G.... [400]

Megaceros .............C.C......G.......A...T.......TA.A.... [391]

Phaeoceros .............C.C......G.......A...TC......TA...... [391]

Anomodon .......C.....C.......TG.......AC.TT.......TA.A.... [391]

Physcomitrella .............C.......TG.......AC.TT.......TA.A.... [391]

Marchantia .............C........G.A.....A...T.......T..A.... [391]

Pleurozia ............CC........G.A.....A...TC......TA.A.... [391]

Treubia .............C........G.A....CA...T.......T..A.... [391]

[ 410 420 430 440 450]

[ . . . . .]

Huperzia_cob -----CTTTTACAGGATACGTACTTCCATGGGGTCAGATGAGCTTTTG-- [434]

Huperzia_cob_230202_229896 -------------------------------------------------- [0]

Huperzia_cob_142451_142637 -------------------------------------------------- [0]

Huperzia_cob_49245_49173 -------------------------------------------------- [0]

Huperzia_cob_30629_30854 CTTTTT....CT..A.........C...A.T..C-.T...........TT [112]

Huperzia_cob_30906_31002 -------------------------------------------------- [0]

Isoetes -----T.C...TG..........CCT........T.A.........C.-- [434]

Cycas -----...C..T............A..TC...............C.C.-- [443]

Arabidopsis -----......T......T.....A..T....................-- [443]

Zea_perennis -----......T...........CA..T....................-- [443]

Megaceros -----......T...C........A..TC.......A...........-- [434]

Phaeoceros -----......T...........CAT.TC.......A.......C...-- [434]

Anomodon -----......T............A..G........A.....T.....-- [434]

Physcomitrella -----......T............A..G........A.....T.....-- [434]

Marchantia -----......T..........T.A..T........A...........-- [434]

Pleurozia -----......T............A..T........A...........-- [434]

Treubia -----......T..........T.A..T........A...........-- [434]

[ 460 470 480 490 500]

[ . . . . .]

Huperzia_cob -GGGAGCTACAGTCATTACCAGCTTAGCTAGCGCCATACCCGTAGT---- [479]

Huperzia_cob_230202_229896 -------------------------------------------------- [0]

Huperzia_cob_142451_142637 -------------------------------------------------- [0]

Huperzia_cob_49245_49173 -------------------------------------------------- [0]

Huperzia_cob_30629_30854 G.......G....A......G...C...............T.....AAGT [162]

Huperzia_cob_30906_31002 -------------------------------------------------- [0]

Isoetes -.....TG....CG.....G...CC...........C......C..---- [479]

Cycas -............A.....G....C...C...........T.....---- [488]

Arabidopsis -............A.....A....................T.....---- [488]

Zea_perennis -......A.....A.....A....................A.....---- [488]

Megaceros -......A..G..A.....A....................T.....---- [479]

Phaeoceros -......A..G..A.....A..................T.T.....---- [479]

Anomodon -..................G...........T..A.....T..G..---- [479]

Physcomitrella -..................A.....G.....T..A.....T..G..---- [479]

Marchantia -...G..C...........A..............A.....T..C..---- [479]

Pleurozia -...G..C.....A.....A..............A.....T..C..---- [479]

Treubia -...G..C...........A..............A.....T..C..---- [479]

[ 510 520 530 540 550]

[ . . . . .]

Huperzia_cob AGGAGACACTATAGTAACTTGGCTTCGGGGTGGCTTCTCCGTAGACAATG [529]

Huperzia_cob_230202_229896 -------------------------------------------------- [0]

Huperzia_cob_142451_142637 -------------------------------------------------- [0]

Huperzia_cob_49245_49173 -------------------------------------------------- [0]

Huperzia_cob_30629_30854 ....T.........CC.T--.A.................T......G... [210]

Huperzia_cob_30906_31002 -------------------------------------------------- [0]

Isoetes ...................C...C.T........................ [529]

Cycas ......T........G...C...C.........T........G....... [538]

Arabidopsis ......T..C.....G.........T.......T........G....... [538]

Zea_perennis ......T..C.....G.........T.......T........G....... [538]

Megaceros ...................C.....T..........TC............ [529]

Phaeoceros .........................T..........T............. [529]

Anomodon ......T..................T.......T.....G.....T.... [529]

Physcomitrella .........................T.......T.....G.....T.... [529]

Marchantia .........................T........................ [529]

Pleurozia G..G.....................T........................ [529]

Treubia ...............G........CT........................ [529]

[ 560 570 580 590 600]

[ . . . . .]

Huperzia_cob CCACCTTAAATCGTTTCTTTAGCCTCCATTACTTACTTCCTTTTTTAATA [579]

Huperzia_cob_230202_229896 -------------------------------------------------- [0]

Huperzia_cob_142451_142637 -------------------------------------------------- [0]

Huperzia_cob_49245_49173 -------------------------------------------------. [1]

Huperzia_cob_30629_30854 ..G.A.C.........---------------------------------- [226]

Huperzia_cob_30906_31002 -------------------------------------------------- [0]

Isoetes ......C.........T.C.....CT..............C...CC.... [579]

Cycas ......CG..............T.CT.....T.....C......A.TTC. [588]

Arabidopsis ................T.....T..T...C.T.....C..C...A.TT.. [588]

Zea_perennis ................T.....T......C.T.....C..CC..A.TT.. [588]

Megaceros .T..............T.C......T..CC......C.......C.C..T [579]

Phaeoceros .A.........T....T........T..C...............A.C..T [579]

Anomodon .T..............T........T.................CA..... [579]

Physcomitrella .T..............T........T..................A..... [579]

Marchantia ................T........T..................A..... [579]

Pleurozia ................T........T..................A..... [579]

Treubia ................T........T..................G..... [579]

[ 610 620 630 640 650]

[ . . . . .]

Huperzia_cob GTAGGCGCTAGTATTCTTCATCTGGCAGCATTACATCAATATGGATCCAA [629]

Huperzia_cob_230202_229896 -------------------------------------------------- [0]

Huperzia_cob_142451_142637 -------------------------------------------------- [0]

Huperzia_cob_49245_49173 ................C...G.....TA...................... [51]

Huperzia_cob_30629_30854 -------------------------------------------------- [226]

Huperzia_cob_30906_31002 -------------------------------------------------- [0]

Isoetes ..............CG......C...T.T.CCGT..A....C........ [629]

Cycas ........C...CC........C...C....CG..............A.. [638]

Arabidopsis ........C...C.............C.....G..............A.. [638]

Zea_perennis .C......C...C.............T.....G..............A.. [638]

Megaceros .C...T.T..............C...T...........G.....T..... [629]

Phaeoceros .C...T.T..........T.......T....C.T..T.......T..... [629]

Anomodon .CT.C....GC....A.......T..T.................C..T.. [629]

Physcomitrella TCT.C...GGT....A.......T..T.....G...........C..T.. [629]

Marchantia .C...T....................T.................T..... [629]

Pleurozia .C...T....................C.....G...........T..... [629]

Treubia .C...T....................T.................T..... [629]

[ 660 670 680 690 700]

[ . . . . .]

Huperzia_cob TAATCCATTGGGTATAAATTCTTCTGCGGATAAAATAGCTTTTTATCCTT [679]

Huperzia_cob_230202_229896 -------------------------------------------------- [0]

Huperzia_cob_142451_142637 -------------------------------------------------- [0]

Huperzia_cob_49245_49173 ...A....C----..C..........------------------------ [73]

Huperzia_cob_30629_30854 -------------------------------------------------- [226]

Huperzia_cob_30906_31002 -------------------------------------------------- [0]

Isoetes ...C....CA.....C...................C....CC.....TCC [679]

Cycas ........C....G..C....AAAGAT.....G...C....C...C..A. [688]

Arabidopsis .............G..C.....GAGAT..................C.... [688]

Zea_perennis .............G..C.....GAGAT.........T....C...C.... [688]

Megaceros ...............C..........TA.............CG....TC. [679]

Phaeoceros .....T.........C..........TA..............G.....C. [679]

Anomodon ...............C..........T.....................C. [679]

Physcomitrella ...............C..........T.....................C. [679]

Marchantia ...............C..........TA....................C. [679]

Pleurozia ...............C..........TA....................C. [679]

Treubia ...............C........C.TA..............C.....C. [679]

[ 710 720 730 740 750]

[ . . . . .]

Huperzia_cob ATTTTTACGTCAAAGATCTAGTGGGTTGGGTAGCATTTGCCATCTTTTTT [729]

Huperzia_cob_230202_229896 -------------------------------------------------- [0]

Huperzia_cob_142451_142637 -------------------------------------------------- [0]

Huperzia_cob_49245_49173 -------------------------------------------------- [73]

Huperzia_cob_30629_30854 -------------------------------------------------- [226]

Huperzia_cob_30906_31002 -------------------------------------------------- [0]

Isoetes ...CCC............C..CA......................C.C.. [729]

Cycas ....C.....A..G....C...A...C.......T.....T....C.... [738]

Arabidopsis .......T.....G........T...........T.....T......... [738]

Zea_perennis .......T..A..G.....T..A...C.......T.C...T......... [738]

Megaceros ...C......A.......C.......C......................C [729]

Phaeoceros ..........A...............C......................C [729]

Anomodon ..A.G.....A......T....AT..........T........T...... [729]

Physcomitrella ..A.G.....A......T....AT..........T........T...... [729]

Marchantia ..A.......A......T....A...........T.....A......... [729]

Pleurozia ..A.......A......T....A...........T..C..A......... [729]

Treubia ..A.......A......T....A...........T.....A....C.... [729]

[ 760 770 780 790 800]

[ . . . . .]

Huperzia_cob TCCATTTTTGTTTTTTATGCACCTAATTTATTAGGGCATCCCGACAATTA [779]

Huperzia_cob_230202_229896 -------------------------------------------------- [0]

Huperzia_cob_142451_142637 -------------------------------------------------- [0]

Huperzia_cob_49245_49173 -------------------------------------------------- [73]

Huperzia_cob_30629_30854 -------------------------------------------------- [226]

Huperzia_cob_30906_31002 -------------------------------------------------- [0]

Isoetes ..TC...CG......C...........C.TC.G........T.......C [779]

Cycas .......GGA..........T..C...GCT..G................. [788]

Arabidopsis ..T....GGA..........T......G.T..G..A.............. [788]

Zea_perennis .......GGA......T...T..A...G.T..G................. [788]

Megaceros ..T..........C...C.........G.C..G..............C.. [779]

Phaeoceros ..T..............C.........G.C..G..............C.. [779]

Anomodon ..T..C...A.................G.T..G..............C.. [779]

Physcomitrella ..T......A.................G.TC.G..............C.. [779]

Marchantia ..T........................G....G..............C.. [779]

Pleurozia ..T.....C....C.........C...G....G..............C.. [779]

Treubia ..T.....CA.................G....G..T...........C.. [779]

[ 810 820 830 840 850]

[ . . . . .]

Huperzia_cob TATACCCGCAAATCCGATGTCCACTCCGGCTCATATTGTGCCAGAATGGT [829]

Huperzia_cob_230202_229896 -------------------------------------------------- [0]

Huperzia_cob_142451_142637 -------------------------------------------------- [0]

Huperzia_cob_49245_49173 -------------------------------------------------- [73]

Huperzia_cob_30629_30854 -------------------------------------------------- [226]

Huperzia_cob_30906_31002 -------------------------------------------------- [0]

Isoetes ......T..G.....................T........T.....C..C [829]

Cycas ......T..T..............C...C.............G...C... [838]

Arabidopsis ......T..T..............C...C.............G....... [838]

Zea_perennis ......T..T.........C....C...C.............G....... [838]

Megaceros .....................A..C......................... [829]

Phaeoceros .....................A..C.T....................... [829]

Anomodon C........G.....C.....A..............A............. [829]

Physcomitrella C........G.....T.....A..............A............. [829]

Marchantia ...............C.....A..C...........A.....G....... [829]

Pleurozia ...............C.....A..C...........A.....G....... [829]

Treubia ...............C.....G..C...........A.....G....... [829]

[ 860 870 880 890 900]

[ . . . . .]

Huperzia_cob ATTTCTCACCTATCTATGCAATTCTTCGAAGTATACCTAACAAATTAGGG [879]

Huperzia_cob_230202_229896 --------..A...G.....................T.TGGG----G... [38]

Huperzia_cob_142451_142637 -------------------------------------------------- [0]

Huperzia_cob_49245_49173 -------------------------------------------------- [73]

Huperzia_cob_30629_30854 -------------------------------------------------- [226]

Huperzia_cob_30906_31002 -------------------------------------------------- [0]

Isoetes ..CC......C...C....G..C.CCT..................C.... [879]

Cycas .....C....A........C....C...C....................A [888]

Arabidopsis .....CT...G...C....C........T.........G.....GCG..A [888]

Zea_perennis .....CT...G...C....C........C.........G.....GCG... [888]

Megaceros ......T...AG.......T.............................. [879]

Phaeoceros ......T...AG.......T......T....................... [879]

Anomodon ......T...GG.T.....G.............................. [879]

Physcomitrella ......T...AG.T.....G.............................. [879]

Marchantia ......T...AG.......G.............................. [879]

Pleurozia ......T...AG.......G.............................. [879]

Treubia ......T...AG.......G....................T......... [879]

[ 910 920 930 940 950]

[ . . . . .]

Huperzia_cob GGTGTAGCTGCCATAGGACTAGTTTTCGTGTCATTATTGG--CTTCACCT [927]

Huperzia_cob_230202_229896 ...................C.....CT...-..C...AT.GA.C.T.... [87]

Huperzia_cob_142451_142637 -------------------------------------------------- [0]

Huperzia_cob_49245_49173 -------------------------------------------------- [73]

Huperzia_cob_30629_30854 -------------------------------------------------- [226]

Huperzia_cob_30906_31002 -------------------------------------------------- [0]

Isoetes ........C.........T......CT.......C.CC..--.CC.G... [927]

Cycas ......A.C..A....C..C.....CTA.A..TCCG....--..C..... [936]

Arabidopsis ........C..A....C..C......TA.A.GTC.C....--...T.... [936]

Zea_perennis ........C..A....C..C......TA.A..TC.C....--...T.... [936]

Megaceros ...................C.....C............T.--..CT.... [927]

Phaeoceros ...................C.....C............T.--..C...TC [927]

Anomodon ..........................T...........T.--...T...G [927]

Physcomitrella ..........................T...........T.--...T...G [927]

Marchantia ........C.................T.............--...T.... [927]

Pleurozia ........C.................T.............--..CT.... [927]

Treubia ....................C.....T.............--...T.... [927]

[ 960 970 980 990 1000]

[ . . . . .]

Huperzia_cob TATATTAACACTTCATAT--GTACGTAGTTCAAGTTCTCGACCTATTCAC [975]

Huperzia_cob_230202_229896 .T.CC.......AA.A.AAG................T..........T.G [137]

Huperzia_cob_142451_142637 ---------..ACTTC..AT...........................T.G [41]

Huperzia_cob_49245_49173 -------------------------------------------------- [73]

Huperzia_cob_30629_30854 -------------------------------------------------- [226]

Huperzia_cob_30906_31002 -------------------------------------------------- [0]

Isoetes CC..CG..T...G.....--...........G........C.....C..T [975]

Cycas .T...G..A.G..TG...--..G......C..........C..G...TG. [984]

Arabidopsis .T.T....A.G.ATG...--..G.............T......G...... [984]

Zea_perennis .T.T....AGAAATG...--..G.............T......G...... [984]

Megaceros .T.........A......--................TCT....G...... [975]

Phaeoceros .T.........A......--...T............TCT....G...... [975]

Anomodon .C......T..A..G...--................T......A...... [975]

Physcomitrella .TC........A..G...--................T......G...... [975]

Marchantia .TC...............--................T......A...... [975]

Pleurozia .TC...............--................T......A...... [975]

Treubia .TC...............--................T......A...... [975]

[ 1010 1020 1030 1040 1050]

[ . . . . .]

Huperzia_cob CAAAGATTGTTTTGGTTGCTTTTGGCAGATTGTTTACTTTCAGGTTGGAT [1025]

Huperzia_cob_230202_229896 ..............C...............C................... [187]

Huperzia_cob_142451_142637 ..............C......C........C................... [91]

Huperzia_cob_49245_49173 -------------------------------------------------- [73]

Huperzia_cob_30629_30854 -------------------------------------------------- [226]

Huperzia_cob_30906_31002 -------------------------------------------------- [0]

Isoetes G.C.A.............T...CTAT....C...C..C...G........ [1025]

Cycas ......A.A...C.......C.....G...C.C.....ACT....C.... [1034]

Arabidopsis ...G..A...................G.....C.....ACT......... [1034]

Zea_perennis ...G..A.A.................G.....C.....ACT......... [1034]

Megaceros ....A.A............CC...........CC......T......... [1025]

Phaeoceros T...A.A.........................CC......T......... [1025]

Anomodon ....A...A............C..........C.......T...C..... [1025]

Physcomitrella ....A...A.......................C.......T...C..... [1025]

Marchantia ....A...T............G.A........C..G....T......... [1025]

Pleurozia ....A................G.A......C.C..G....T......... [1025]

Treubia ....A................G.A........C..G....T......... [1025]

[ 1060 1070 1080 1090 1100]

[ . . . . .]

Huperzia_cob TGGATGTCAACCCGTAG-----AAGCACCAT-------ATGTAACTATTG [1063]

Huperzia_cob_230202_229896 ...........T...G.-----.G...T...CCTTCGG............ [232]

Huperzia_cob_142451_142637 ............T..G.TGTGG.G.......CCCTCGA............ [141]

Huperzia_cob_49245_49173 -------------------------------------------------- [73]

Huperzia_cob_30629_30854 -------------------------------------------------- [226]

Huperzia_cob_30906_31002 ------------------------------.-------.C..T....... [13]

Isoetes ............T..G.-----.G...T...-------............ [1063]

Cycas C......A.......G.-----.G.......-------....T....... [1072]

Arabidopsis C...........T..G.-----.G..T....-------T...T....... [1072]

Zea_perennis C...........T..G.-----.G.......-------T...T....... [1072]

Megaceros .......T....T..G.-----.G.......-------....T....... [1063]

Phaeoceros ............T..G.-----.G.......-------....T....... [1063]

Anomodon C...........T..G.-----.........-------....T....... [1063]

Physcomitrella ............T..G.-----.........-------....T....... [1063]

Marchantia ...............G.-----.........-------....T....... [1063]

Pleurozia ............T..G.-----.........-------....T....... [1063]

Treubia ...............G.-----.........-------....T....... [1063]

[ 1110 1120 1130 1140 1150]

[ . . . . .]

Huperzia_cob GACAAATAGCCTCAGTAGGCTTTTTCTTTTATTTCGCCATTA---CGCCT [1110]

Huperzia_cob_230202_229896 .......TA----......T.A...A........T..T....---....C [275]

Huperzia_cob_142451_142637 .......G..TC.......T.C..C..C......T.......---....- [187]

Huperzia_cob_49245_49173 -------------------------------------------------- [73]

Huperzia_cob_30629_30854 -------------------------------------------------- [226]

Huperzia_cob_30906_31002 ......AG.TG.A......TCC..-...C........T....TTA...GC [62]

Isoetes ..T.......T.TG......CCCC.....C...C...T....---..... [1110]

Cycas .......TC.T.......CTCCC.C...G.TC........A.---....C [1119]

Arabidopsis .......TT.TC.TT.G.TT..C.....G.TC..T.....A.---....C [1119]

Zea_perennis .......TT.T..TT.CTTT..C.....G.TC..T.....A.---....C [1119]

Megaceros .C.....T..T..G..T.CT.C......A..C..T.TT....---..... [1110]

Phaeoceros .CT....T..T..G..T..T.C....C.A..C..T.TT....---..... [1110]

Anomodon .......T..T.....T..T........C.....T..T....---....C [1110]

Physcomitrella .......T..T.....T..T........C.....T..T....---....C [1110]

Marchantia .......T..T.....G..T........C.....T..T..A.---....C [1110]

Pleurozia .......T..T.....G..T........C.....T..T..A.---....C [1110]

Treubia .......T..T.....G..T........C.....T..T..A.---....C [1110]

[ 1160 1170 ]

[ . . ]

Huperzia_cob ATTCTTGG---CAAATTAGAAGCCAAATT [1136]

Huperzia_cob_230202_229896 .AAAAA..---....A..T.......... [301]

Huperzia_cob_142451_142637 ----------------------------- [187]

Huperzia_cob_49245_49173 ----------------------------- [73]

Huperzia_cob_30629_30854 ----------------------------- [226]

Huperzia_cob_30906_31002 ........CCG.C.T...A......G... [91]

Isoetes ....C...---.G...........TTC.. [1136]

Cycas ....CG..--A.G.G..G....AAG.... [1146]

Arabidopsis .....G..--A.G.G..G....AGG.... [1146]

Zea_perennis ....CG..--A.G.G..G....AGG.... [1146]

Megaceros ........---...........A..G... [1136]

Phaeoceros ........---...........A..G... [1136]

Anomodon .....C..---.G..........T.G... [1136]

Physcomitrella .....C..---.G..........T.G... [1136]

Marchantia ........---.....GT.......G... [1136]

Pleurozia ........---.....GT.......G... [1136]

Treubia ........---.....GT.......G... [1136]

;

END;

**cox1**

#NEXUS

[MacClade 4.05 registered to Yin-Long Qiu Lab, EEB, University of Michigan]

BEGIN DATA;

DIMENSIONS NTAX=16 NCHAR=1678;

FORMAT DATATYPE=DNA MISSING=? GAP=- MATCHCHAR=. INTERLEAVE ;

MATRIX

[ 10 20 30 40 50]

[ . . . . .]

Huperzia_cox1 ACGAACAATTTTGCACAAAGATGGCTTTTTCCCACGAACCACAAAGATAT [50]

Huperzia_cox1_4187_4471 -------------------------------------------------- [0]

Huperzia_cox1_81728_81627 -------------------------------------------------- [0]

Huperzia_cox1_321716_321817 -------------------------------------------------- [0]

Huperzia_cox1_326924_327263 -------------------------------------------------- [0]

Huperzia_cox1_332216_332361 ---------------------------..G...............A.A.G [23]

Arabidopsis .T...A...C.G.TT.---.......G..CT....A........G..... [47]

Zea_perennis .T..CA...C.G.TC.---.......C..CT....T........G..... [47]

Cycas ....CA.....G.TC.---......CG..C.....C.......GG..... [47]

Osmunda_JF681372 .T.........A..............G...T................... [50]

Phaeoceros .T...A........................T....A.............. [50]

Megaceros .T...A........................T....A.............. [50]

Physcomitrella .T........................G...T.T................. [50]

Anomodon .T........................G...T................... [50]

Treubia .T...............G........G...T................... [50]

Marchantia .T........................G...T................... [50]

[ 60 70 80 90 100]

[ . . . . .]

Huperzia_cox1 AGGTACTCCA--TATCGTATTTTTGGTGCCATTGCTGGAGTAATGGGTAC [98]

Huperzia_cox1_4187_4471 -------------------------------------------------- [0]

Huperzia_cox1_81728_81627 -------------------------------------------------- [0]

Huperzia_cox1_321716_321817 -------------------------------------------------- [0]

Huperzia_cox1_326924_327263 -------------------------------------------------- [0]

Huperzia_cox1_332216_332361 .........CCC...T......CC....T..................... [73]

Arabidopsis ...G....TC--...TTC.....C.................G.....C.. [95]

Zea_perennis C.......T.--...TTC..C..C...........A.....G.....C.. [95]

Cycas ...G....T.--...TCA..C....................G.....C.. [95]

Osmunda_JF681372 ..........--...TCA..C..C........G..............C.. [98]

Phaeoceros ..........--...TCA.....C.....T.................... [98]

Megaceros ..........--C..TCA.....C.....T.................... [98]

Physcomitrella ...G....T.--...TTA.................C.....C........ [98]

Anomodon ...G....T.--...T.C.......................T........ [98]

Treubia ........T.--...TTA.....C.......................... [98]

Marchantia ........T.--...TTA.....C.......................... [98]

[ 110 120 130 140 150]

[ . . . . .]

Huperzia_cox1 ATGCTTTTC-CGTACTAATTCGTATGGAATTAGCACAACCTGGCAATCAA [147]

Huperzia_cox1_4187_4471 -------------------------------------------------- [0]

Huperzia_cox1_81728_81627 -------------------------------------------------- [0]

Huperzia_cox1_321716_321817 -------------------------------------------------- [0]

Huperzia_cox1_326924_327263 -------------------------------------------------- [0]

Huperzia_cox1_332216_332361 ........TTA....C..............................---. [120]

Arabidopsis ......C..-A.....G...................G...C...G..... [144]

Zea_perennis ......C..-......G.................C.G...C...G..... [144]

Cycas ......C..-A....C...............G........C...G..... [144]

Osmunda_JF681372 ......CC.-A........C........................C..... [147]

Phaeoceros .........-A...T.....T..............T...TC......... [147]

Megaceros ......C.T-A...TC........................C......... [147]

Physcomitrella ......C..-A..........................G............ [147]

Anomodon .........-G..........................G............ [147]

Treubia ......C..-A.............................C......... [147]

Marchantia .........-A.............................C.....C... [147]

[ 160 170 180 190 200]

[ . . . . .]

Huperzia_cox1 ATTCCTGGTGGGAATCATCAACTTTATAATGTGTTAATTACAGCTCATGC [197]

Huperzia_cox1_4187_4471 -------------------------------------------------- [0]

Huperzia_cox1_81728_81627 -------------------------------------------------- [0]

Huperzia_cox1_321716_321817 -------------------------------------------------- [0]

Huperzia_cox1_326924_327263 -------------------------------------------------- [0]

Huperzia_cox1_332216_332361 ..A.T-..........C...GT.....----------------------- [146]

Arabidopsis ....T...........................T.....A........... [194]

Zea_perennis ....T...........................T.....A..G.....C.. [194]

Cycas ....T......A............C.............A..G.....C.. [194]

Osmunda_JF681372 ....T......A..........................A..C.....C.. [197]

Phaeoceros ....TCA....A..........................A......T.C.T [197]

Megaceros ....TCA....A...........C..............A........C.. [197]

Physcomitrella ....T......A..........................A........C.. [197]

Anomodon ....T......A..........................A........C.. [197]

Treubia ....T......A....................A.....A........C.. [197]

Marchantia ....T......A..........................A........C.. [197]

[ 210 220 230 240 250]

[ . . . . .]

Huperzia_cox1 TTTTCCAATGATCTTTTTTATGGTTATGCCAGCGATGATAGGTGGATTTG [247]

Huperzia_cox1_4187_4471 -------------------------------------------------- [0]

Huperzia_cox1_81728_81627 -------------------------------------------------- [0]

Huperzia_cox1_321716_321817 -------------------------------------------------- [0]

Huperzia_cox1_326924_327263 -------------------------------------------------- [0]

Huperzia_cox1_332216_332361 -------------------------------------------------- [146]

Arabidopsis ....TT........................G................... [244]

Zea_perennis ....TT........................G................... [244]

Cycas ..C.TT........C.........C.....G................... [244]

Osmunda_JF681372 ....TT......A..C...CC.......T.G................C.. [247]

Phaeoceros ....TT......A................TT..C................ [247]

Megaceros ....TT......A.C...........C...T..C................ [247]

Physcomitrella ....TT............C...........T................... [247]

Anomodon ....TT.........C..............T................... [247]

Treubia ....T.........CC..C...........G..............T.... [247]

Marchantia ....TT.........C..............G..............T.... [247]

[ 260 270 280 290 300]

[ . . . . .]

Huperzia_cox1 GTAATTGGTTCGTTCCGATTCTTATAGGTGCACCTGACATGGCATTTCCA [297]

Huperzia_cox1_4187_4471 -------------------------------------------------- [0]

Huperzia_cox1_81728_81627 -------------------------------------------------- [0]

Huperzia_cox1_321716_321817 -------------------------------------------------- [0]

Huperzia_cox1_326924_327263 -------------------------------------------------- [0]

Huperzia_cox1_332216_332361 -------------------------------------------------- [146]

Arabidopsis ..........T...........G........................... [294]

Zea_perennis .G........T...........G........................... [294]

Cycas .................................................. [294]

Osmunda_JF681372 .................................................. [297]

Phaeoceros ..........T...TT...............................TT. [297]

Megaceros ..........T...........C........................... [297]

Physcomitrella ............................C........T............ [297]

Anomodon .....................................T............ [297]

Treubia ................T...........AAGT..G..T........C..C [297]

Marchantia ..........T.....T...........AAGT..G..T........C..T [297]

[ 310 320 330 340 350]

[ . . . . .]

Huperzia_cox1 CGATTAAATAATATTCCCTTTTGGTTGTTGCCACCTTCGCTGTTGCTTTT [347]

Huperzia_cox1_4187_4471 -------------------------------------------------- [0]

Huperzia_cox1_81728_81627 -------------------------------------------------- [0]

Huperzia_cox1_321716_321817 -------------------------------------------------- [0]

Huperzia_cox1_326924_327263 -------------------------------------------------- [0]

Huperzia_cox1_332216_332361 -------------------------------------------------- [146]

Arabidopsis ...............T.A..C..............AAGT..C.....CC. [344]

Zea_perennis ..............AT.A..C..............AAGT..C.....CC. [344]

Cycas ..............CT.A..CC.....C...................CC. [344]

Osmunda_JF681372 T..............T.A.................C.............. [347]

Phaeoceros T....G........CAGT......C.A..A..G..G..A...C.A...C. [347]

Megaceros .....G........CAGT.C....C.A..A..G..G..A...C.A...C. [347]

Physcomitrella ...........C...AGT...........A.....G..A..C..A...C. [347]

Anomodon ...........C...AGT...........A.....G..A.....A...C. [347]

Treubia A..............T.A......C.T........G..AT....A...C. [347]

Marchantia A..............T.A......C.T........G..AT....A...C. [347]

[ 360 370 380 390 400]

[ . . . . .]

Huperzia_cox1 ATTAAGCTCAGCCTTGGTAGAGGTGGGTAGCGGCACTGGGTGGACGGTCT [397]

Huperzia_cox1_4187_4471 -------------------------------------------------- [0]

Huperzia_cox1_81728_81627 -------------------------------------------------- [0]

Huperzia_cox1_321716_321817 -------------------------------------------------- [0]

Huperzia_cox1_326924_327263 -------------------------------------------------- [0]

Huperzia_cox1_332216_332361 -------------------------------------------------- [146]

Arabidopsis ...............A.....A..A......................... [394]

Zea_perennis ...............A.....A.....C...................... [394]

Cycas .............C.......A...........................C [394]

Osmunda_JF681372 .................................................. [397]

Phaeoceros T.....T..C..T........A..T..CGCA..T.....C....T..... [397]

Megaceros T.....TC.T..T........A..T..CGCA..T..C..C.......... [397]

Physcomitrella C.....T..T..T........A.....CGCT..T..C..A.......... [397]

Anomodon C.....T..T..TC.......A.....CGCT..T..C..A........T. [397]

Treubia T.C............A............T....TT.G............. [397]

Marchantia TC.............A............T....TT.A............. [397]

[ 410 420 430 440 450]

[ . . . . .]

Huperzia_cox1 ATCCGCCCTTAAGCGGTATAACCAGCCATTCCGGAGGATCCGTTGATTCA [447]

Huperzia_cox1_4187_4471 -------------------------------------------------- [0]

Huperzia_cox1_81728_81627 -------------------------------------------------- [0]

Huperzia_cox1_321716_321817 -------------------------------------------------- [0]

Huperzia_cox1_326924_327263 -------------------------------------------------- [0]

Huperzia_cox1_332216_332361 -------------------------------------------------- [146]

Arabidopsis .............T.....T.....T.....T......G.A.......T. [444]

Zea_perennis .............T.....T...........T......G.A.......T. [444]

Cycas .............T.....T.....T.....T......G.T.C....... [444]

Osmunda_JF681372 ....C.....G..T...........T....T.......G.T......... [447]

Phaeoceros ..TT.TTTC....T........G..TT...........G.........T. [447]

Megaceros .......TC....T........G..T............G.........T. [447]

Physcomitrella .......G.....T...........T.....T........T..G....T. [447]

Anomodon .......G.....T...........T.....T........T..G....T. [447]

Treubia ....A........T...........T.....T........T.......T. [447]

Marchantia ....A........T...........T.....T........T.......T. [447]

[ 460 470 480 490 500]

[ . . . . .]

Huperzia_cox1 GCAATTTTTAGTCCTCATTTATCAGGTGTTTCATCTATTTTAGGTTCTAT [497]

Huperzia_cox1_4187_4471 -------------------------------------------------- [0]

Huperzia_cox1_81728_81627 -------------------------------------------------- [0]

Huperzia_cox1_321716_321817 -------------------------------------------------- [0]

Huperzia_cox1_326924_327263 -------------------------------------------------- [0]

Huperzia_cox1_332216_332361 -------------------------------------------------- [146]

Arabidopsis .............T....C....T...........C.............. [494]

Zea_perennis .............T....C................A.............. [494]

Cycas .......C.....T.....................C....C......... [494]

Osmunda_JF681372 ..C....C.....T.....C..........C.CCT.....C......... [497]

Phaeoceros ............T..T...............T..T.....C....G.... [497]

Megaceros ..C..........T....C..............CT.....C....G.... [497]

Physcomitrella ..C.....C....T.........G........C................. [497]

Anomodon ..C.....C....TC.................C................. [497]

Treubia ..C....C...C.T..................T................. [497]

Marchantia .............T..................C................. [497]

[ 510 520 530 540 550]

[ . . . . .]

Huperzia_cox1 AAATTTTATTACTACTATTTTCAACATGCGCGGCCCTGGAATGACCATGC [547]

Huperzia_cox1_4187_4471 -------------------------------------------------- [0]

Huperzia_cox1_81728_81627 -------------------------------------------------- [0]

Huperzia_cox1_321716_321817 -------------------------------------------------- [0]

Huperzia_cox1_326924_327263 -------------------------------------------------- [0]

Huperzia_cox1_332216_332361 -------------------------------------------------- [146]

Arabidopsis C........A..A.....C...........T..A...........T.... [544]

Zea_perennis C........A........C...........T..A...........T.... [544]

Cycas C....C...A........CCC............A...........T.... [544]

Osmunda_JF681372 T....C...A..C.....C.C............A................ [547]

Phaeoceros T........C.G......C..T..T....................T.... [547]

Megaceros T...C....C.G......C..T..T....................T.... [547]

Physcomitrella C........C....................T..G..A............. [547]

Anomodon C........C....................T..G..A............. [547]

Treubia T........A..A.....C.....T...A.G.........T....T.... [547]

Marchantia T........A..A.....C.....T...A.G.C.......T......... [547]

[ 560 570 580 590 600]

[ . . . . .]

Huperzia_cox1 ATAGATTACCTCTATTTGTTTGGTCTGTTTCAGTCACCGCATTCTCACCT [597]

Huperzia_cox1_4187_4471 -------------------------------------------------- [0]

Huperzia_cox1_81728_81627 -------------------------------------------------- [0]

Huperzia_cox1_321716_321817 -------------------------------------------------- [0]

Huperzia_cox1_326924_327263 -------------------------------------------------- [0]

Huperzia_cox1_332216_332361 -------------------------------------------------- [146]

Arabidopsis ..........C........G.....C...CT...G..A......CT..T. [594]

Zea_perennis ..........A..T.....G.....C....T...G..A......CT..T. [594]

Cycas ......C...C.....C..GC....C...C....G..A......CT..T. [594]

Osmunda_JF681372 ..........C.C.......C.............G..A......C..TT. [597]

Phaeoceros ...A...G.T.T.......G....TC....T...G..A.......T..T. [597]

Megaceros ...A...G...T.......G...C......T...G..A......CT..TC [597]

Physcomitrella ..........C.....C..A.....C..A.T...G..A......CT..T. [597]

Anomodon ..........C.....C..A.....C..A.T...G..A......CT..T. [597]

Treubia ...................G..........T...G..A..T...CT..T. [597]

Marchantia ...................G..........T...A..A..T...CT..T. [597]

[ 610 620 630 640 650]

[ . . . . .]

Huperzia_cox1 TCATTATCCCTTCCTGTATTGGCAGGTGCAATTACCATGTTATTAACCGA [647]

Huperzia_cox1_4187_4471 -------------------------------------------------- [0]

Huperzia_cox1_81728_81627 -------------------------------------------------- [0]

Huperzia_cox1_321716_321817 -------------------------------------------------- [0]

Huperzia_cox1_326924_327263 -------------------------------------------------- [0]

Huperzia_cox1_332216_332361 -------------------------------------------------- [146]

Arabidopsis .T......A..C..G...C.......G....................... [644]

Zea_perennis .T......A.....G...C.......G........A.............. [644]

Cycas CT......A.....G...CC......G.................G..... [644]

Osmunda_JF681372 .T................C............................... [647]

Phaeoceros .T.....T.T..TTA...C..........C.................T.. [647]

Megaceros .T............A...C..........C.................T.. [647]

Physcomitrella .T......T.....A................................T.. [647]

Anomodon .T......T.....A................................T.. [647]

Treubia .T............A...C............................T.. [647]

Marchantia .T............A...C............................T.. [647]

[ 660 670 680 690 700]

[ . . . . .]

Huperzia_cox1 TAGAAATTTTAATACAACCTTTTTTGATCCTGCTGGAGGGGGAGATCCAA [697]

Huperzia_cox1_4187_4471 -------------------------------------------------- [0]

Huperzia_cox1_81728_81627 -------------------------------------------------- [0]

Huperzia_cox1_321716_321817 -------------------------------------------------- [0]

Huperzia_cox1_326924_327263 -------------------------------------------------- [0]

Huperzia_cox1_332216_332361 -------------------------------------------------- [146]

Arabidopsis .C....C.......................C..............C.... [694]

Zea_perennis .C....C.......................A..A...........C.... [694]

Cycas .C....C................C.....................C..G. [694]

Osmunda_JF681372 .C....C.......TC...C...C......C..............C..T. [697]

Phaeoceros ......C........C.............TC..A.....A.......TG. [697]

Megaceros ......C........C....C.........C..G.....A........G. [697]

Physcomitrella ...G..C..........................A.....A.......... [697]

Anomodon ...G..C..........................A.....A.......... [697]

Treubia ....................................T..C..G.....C. [697]

Marchantia ......C.............................T..C..G.....C. [697]

[ 710 720 730 740 750]

[ . . . . .]

Huperzia_cox1 TTTTATACCAGCATCTTTTTTGGTTCTTCGGTCATCCAGAGGTTTATATT [747]

Huperzia_cox1_4187_4471 -------------------------------------------------- [0]

Huperzia_cox1_81728_81627 -------------------------------------------------- [0]

Huperzia_cox1_321716_321817 -------------------------------------------------- [0]

Huperzia_cox1_326924_327263 -------------------------------------------------- [0]

Huperzia_cox1_332216_332361 -------------------------------------------------- [146]

Arabidopsis ................C..........................G...... [744]

Zea_perennis .A..............C..........................G...... [744]

Cycas .A..............C...C...C..................GC..... [744]

Osmunda_JF681372 .C................C........C................C..... [747]

Phaeoceros ...........T....A........T..T...T................. [747]

Megaceros ...............CA.......CT..................C..... [747]

Physcomitrella ................G........T...........T.....C...... [747]

Anomodon .A..............C........T..T........T.....C...... [747]

Treubia ..C.........................T.....C..T..A......... [747]

Marchantia .....................................T............ [747]

[ 760 770 780 790 800]

[ . . . . .]

Huperzia_cox1 CCAATTTTGCCAGGATTTGGTATTATTAGTCATATCGTTTCTACCTTTTC [797]

Huperzia_cox1_4187_4471 -------------------------------------------------- [0]

Huperzia_cox1_81728_81627 -------------------------------------------------- [0]

Huperzia_cox1_321716_321817 -------------------------------------------------- [0]

Huperzia_cox1_326924_327263 -------------------------------------------------- [0]

Huperzia_cox1_332216_332361 -------------------------------------------------- [146]

Arabidopsis .TC...C....T.....C.....C..A..............G..T..... [794]

Zea_perennis .TC...C....T.....C....................A..G........ [794]

Cycas ..C...CC...C....CC.....C..............A..G..T..... [794]

Osmunda_JF681372 T.T...CC.........C.....C..............C.....T..CC. [797]

Phaeoceros .T.......T.......C.....A................TC..G....T [797]

Megaceros .................C.....A.................C..G...C. [797]

Physcomitrella .T.....C.........C.....C.......................... [797]

Anomodon .T...C...........C.....C.......................... [797]

Treubia .T....C..........C.....C..............C........... [797]

Marchantia .T....C................C..............C........... [797]

[ 810 820 830 840 850]

[ . . . . .]

Huperzia_cox1 AAGAAAACCTGTATTCGGTTATCTAGGCATGGTTTATGCCATGATCAGTA [847]

Huperzia_cox1_4187_4471 -------------------------------------------------- [0]

Huperzia_cox1_81728_81627 -------------------------------------------------- [0]

Huperzia_cox1_321716_321817 -------------------------------------------------- [0]

Huperzia_cox1_326924_327263 -------------------------------------------------- [0]

Huperzia_cox1_332216_332361 -------------------------------------------------- [146]

Arabidopsis GG.......G..C.....G............................... [844]

Zea_perennis .........G..C.....G..........................A.... [844]

Cycas GG.......G..C.....G............................... [844]

Osmunda_JF681372 C...........C..........C.........G................ [847]

Phaeoceros ........TC............................TT.......... [847]

Megaceros ......................................T..C........ [847]

Physcomitrella .........C.....T........................T......... [847]

Anomodon .........C.....T........................T......... [847]

Treubia .........C.......................G..C............. [847]

Marchantia .........C.......................G..C........T.... [847]

[ 860 870 880 890 900]

[ . . . . .]

Huperzia_cox1 TTGGAGTTCTTGGATTTATTGCGCGGGCCCATCATATGTTTACTGTAGGT [897]

Huperzia_cox1_4187_4471 -------------------------------------------------- [0]

Huperzia_cox1_81728_81627 -------------------------------------------------- [0]

Huperzia_cox1_321716_321817 -------------------------------------------------- [0]

Huperzia_cox1_326924_327263 -------------------------------------------------- [0]

Huperzia_cox1_332216_332361 -------------------------------------------------- [146]

Arabidopsis ....T..CT.A......C...TTT....T.................G..C [894]

Zea_perennis .A..T............C.A.TTT....T.................G..C [894]

Cycas ....T....C.....C.CC..TT.....T..........C......G..C [894]

Osmunda_JF681372 ........TC...........TT.....T..............C.....C [897]

Phaeoceros ..........C........C.T.T....A..CT.C..............C [897]

Megaceros ..........C........C.T.T....A..C..C..............C [897]

Physcomitrella .....................T.T....G..C..C..............C [897]

Anomodon .....................T.T....G..C..C........C.....C [897]

Treubia .....................TAT....T..............C...... [897]

Marchantia .....................TAT....T.....C............... [897]

[ 910 920 930 940 950]

[ . . . . .]

Huperzia_cox1 TTAGACGTTGATACACGTGCTTACTTTACCGCAGCTACAATGATTACTGC [947]

Huperzia_cox1_4187_4471 -------------------------------------------------- [0]

Huperzia_cox1_81728_81627 -------------------------------------------------- [0]

Huperzia_cox1_321716_321817 -------------------------------------------------- [0]

Huperzia_cox1_326924_327263 -------------------------------------------------- [0]

Huperzia_cox1_332216_332361 -------------------------------------------------- [146]

Arabidopsis ........A.....C.....C.....C...........C.....C.TA.. [944]

Zea_perennis ..............G.....C.....C...........C.....C.TA.. [944]

Cycas .C............G......C...C............C.....C.TA.. [944]

Osmunda_JF681372 .C......G......T.............G..G..C..C.......TC.. [947]

Phaeoceros ..............TT....C.......TT..G.T..TC.......T..T [947]

Megaceros ..............T.....C........T..G.....C........... [947]

Physcomitrella .....T....................C..T..G.....C.......T... [947]

Anomodon .....T....................C..T..G.....C.....C.T... [947]

Treubia .....T....................C..T..G.....C.......TA.. [947]

Marchantia .....T..........................G.....C.......TA.. [947]

[ 960 970 980 990 1000]

[ . . . . .]

Huperzia_cox1 TGTGCCTACTGGAATAAAGATTTTTAGTTGGATCGCTACCATGTGGGGAG [997]

Huperzia_cox1_4187_4471 -------------------------------------------------- [0]

Huperzia_cox1_81728_81627 -------------------------------------------------- [0]

Huperzia_cox1_321716_321817 -------------------------------------------------- [0]

Huperzia_cox1_326924_327263 -------------------------------------------------- [0]

Huperzia_cox1_332216_332361 -------------------------------------------------- [146]

Arabidopsis ...C..C........C..A..C..........................G. [994]

Zea_perennis ......C...........A..C............................ [994]

Cycas ...C..C........C..A..C.C....C..................... [994]

Osmunda_JF681372 C..............C.......C....C....T................ [997]

Phaeoceros ....T..........T......C........GG..T..TT........G. [997]

Megaceros ...A...........T...............GG......T........G. [997]

Physcomitrella C.................A..............T................ [997]

Anomodon C.................A..............T................ [997]

Treubia C..............C..A.....C........T..A...........G. [997]

Marchantia .................................T..A...........G. [997]

[ 1010 1020 1030 1040 1050]

[ . . . . .]

Huperzia_cox1 GTTCAATACAATATAAAACACCCATGTTATTCGCAGTAGGTTTTATTTTC [1047]

Huperzia_cox1_4187_4471 -------------------------------------------------- [0]

Huperzia_cox1_81728_81627 -------------------------------------------------- [0]

Huperzia_cox1_321716_321817 -------------------------------------------------- [0]

Huperzia_cox1_326924_327263 -------------------------------------------------- [0]

Huperzia_cox1_332216_332361 -------------------------------------------------- [146]

Arabidopsis ....G........C.................T..T.....A..C..C..T [1044]

Zea_perennis ....G........C.................T..T.....G..C..C..T [1044]

Cycas ....G....G...C...............CCT..T.....G.C...C.CT [1044]

Osmunda_JF681372 .............C....................G.....G.....C... [1047]

Phaeoceros ....G...G.G..C.CCT...TTC.T..T..T.................. [1047]

Megaceros ....G...G.G..C.CCT....TC.T..T..T.........C........ [1047]

Physcomitrella .............C.................T..T.....A......... [1047]

Anomodon .............C.................T..T.....A.C...C..T [1047]

Treubia .G...........C....................C.....G.....A... [1047]

Marchantia .G...........C....................T.....G.....A..T [1047]

[ 1060 1070 1080 1090 1100]

[ . . . . .]

Huperzia_cox1 TTGTTCACCATAGGAGGCCTTACTGGAATAGTCTTGGCCAATTCTGGGCT [1097]

Huperzia_cox1_4187_4471 -------------------------------------------------- [0]

Huperzia_cox1_81728_81627 -------------------------------------------------- [0]

Huperzia_cox1_321716_321817 -------------------------------------------------- [0]

Huperzia_cox1_326924_327263 -------------------------------------------------- [0]

Huperzia_cox1_332216_332361 -------------------------------------------------- [146]

Arabidopsis .................A..C............C....A.....A..... [1094]

Zea_perennis .................G..C...........TC.A..A..C........ [1094]

Cycas .C..C............A.CC............CC...A........... [1094]

Osmunda_JF681372 CC......T........A.CC.T...T.C....CC.......C.C...A. [1097]

Phaeoceros ..A..T..TG.G......TG..T.....C...A.C.........C..... [1097]

Megaceros ..AC.....G.G......TG............A...........C..... [1097]

Physcomitrella .....T..TG....G..T..............A................. [1097]

Anomodon .....T..TG....G..T..............A................. [1097]

Treubia ........GG....G..G..............A................. [1097]

Marchantia ........GG....G..G..............A...............G. [1097]

[ 1110 1120 1130 1140 1150]

[ . . . . .]

Huperzia_cox1 GGACATTGCTCTACATGATACTTATTATGTTGTTGCACATTTCCATTATG [1147]

Huperzia_cox1_4187_4471 -------------------------------------------------- [0]

Huperzia_cox1_81728_81627 -------------------------------------------------- [0]

Huperzia_cox1_321716_321817 -------------------------------------------------- [0]

Huperzia_cox1_326924_327263 -------------------------------------------------- [0]

Huperzia_cox1_332216_332361 -------------------------------------------------- [146]

Arabidopsis A.............................G................... [1144]

Zea_perennis A.............................G................... [1144]

Cycas A..........C..................G..........C........ [1144]

Osmunda_JF681372 A.............................G......T........C... [1147]

Phaeoceros ...........................C..G......T....TT...... [1147]

Megaceros ...........C...............C..G.........C.T....... [1147]

Physcomitrella ......C.......................G...........T....... [1147]

Anomodon ......C.......................G................... [1147]

Treubia A.............................G................... [1147]

Marchantia A..T..........................G................... [1147]

[ 1160 1170 1180 1190 1200]

[ . . . . .]

Huperzia_cox1 TACTTTCTATGGGAGCTGTTTTTGCTTTATTTGCAGGATCCTATTATTGG [1197]

Huperzia_cox1_4187_4471 -------------------------------------------------- [0]

Huperzia_cox1_81728_81627 -------------------------------------------------- [0]

Huperzia_cox1_321716_321817 -------------------------------------------------- [0]

Huperzia_cox1_326924_327263 -------------------------------------------------- [0]

Huperzia_cox1_332216_332361 -------------------------------------------------- [146]

Arabidopsis ................C......................TT..C...... [1194]

Zea_perennis ................C.................T....TT..C...... [1194]

Cycas ................C.........CC............TC.C.C.C.. [1194]

Osmunda_JF681372 ....C...........C..........C..CC..C......C.C...C.. [1197]

Phaeoceros .TTC..T.........C.......T..............TT......... [1197]

Megaceros .T...C..........C....C.................TT......... [1197]

Physcomitrella .T.....................................T...C...... [1197]

Anomodon .T.........................C...........T...C...... [1197]

Treubia .T..............C.....C...........G....T.......... [1197]

Marchantia .T..............C.................G....T......C... [1197]

[ 1210 1220 1230 1240 1250]

[ . . . . .]

Huperzia_cox1 ATAGGTAAAATCCCTGGTCTTCAATATCCAGAGACTTTAGGTCAAATTCA [1247]

Huperzia_cox1_4187_4471 -------------------------------------------------- [0]

Huperzia_cox1_81728_81627 -------------------------------------------------- [0]

Huperzia_cox1_321716_321817 -------------------------------------------------- [0]

Huperzia_cox1_326924_327263 -------------------------------------------------- [0]

Huperzia_cox1_332216_332361 -------------------------------------------------- [146]

Arabidopsis G.G.........TT.....GGAC...C..T..A..............C.. [1244]

Zea_perennis G.G.........TT.....GGAC......T..A........C.....C.. [1244]

Cycas GCG.........T......GAAC...C..T..A..............C.. [1244]

Osmunda_JF681372 ..G..G.....AT..................................... [1247]

Phaeoceros ...........AA.A.....C......TT.....T........T...AT. [1247]

Megaceros ...........GA.G.....C......................T...A.. [1247]

Physcomitrella ...........AA..................................... [1247]

Anomodon .....G.....AA..................................... [1247]

Treubia ...........AA.............C....................... [1247]

Marchantia ...........AA.............C....................... [1247]

[ 1260 1270 1280 1290 1300]

[ . . . . .]

Huperzia_cox1 TTTTTGGATTACTTTCTTTGGAGTAAATTCGACTTCCTTTCCTATGCATT [1297]

Huperzia_cox1_4187_4471 -------------------------------------------------- [0]

Huperzia_cox1_81728_81627 -------------------------------------------------- [0]

Huperzia_cox1_321716_321817 -------------------------------------------------- [0]

Huperzia_cox1_326924_327263 -------------------------------------------------- [0]

Huperzia_cox1_332216_332361 -------------------------------------------------- [146]

Arabidopsis .........C.....T..C..G..T....T...C.T.............. [1294]

Zea_perennis .........C.....T..C..G..T...CT...C.T......C....... [1294]

Cycas ...CC....C....CT..C..G..T........C.T......C....... [1294]

Osmunda_JF681372 ..CCC..G.......T........G....T....CT.............. [1297]

Phaeoceros .....................T..C..CCTA..........TA....... [1297]

Megaceros .....................T..T..C.TA........C..A.C.T... [1297]

Physcomitrella .........C...........T..G..C.T.....TT............. [1297]

Anomodon .........C........C..C..G..C.T.....T.............. [1297]

Treubia .....................T......CT.....T.............. [1297]

Marchantia .....................T..G....T.....T.............. [1297]

[ 1310 1320 1330 1340 1350]

[ . . . . .]

Huperzia_cox1 TTCTAGGTCTTGCGGGTATGCCACGTCGTATTCCAGATTATCCA----GA [1343]

Huperzia_cox1_4187_4471 ----------...A.A..........T.C.........C.....TCCG.. [40]

Huperzia_cox1_81728_81627 -------------------------------------------------- [0]

Huperzia_cox1_321716_321817 -------------------------------------------------- [0]

Huperzia_cox1_326924_327263 -----------............T..............CG..T.GATGCT [39]

Huperzia_cox1_332216_332361 -------------------------------------------------- [146]

Arabidopsis .CT....G...T.A.............................G----.. [1340]

Zea_perennis .CT....G...T................C...............----.. [1340]

Cycas .CT.G..G...T................C...............----.. [1340]

Osmunda_JF681372 .C.C.......T....G...........C.....T.........----.. [1343]

Phaeoceros .AGA.CCG.....A..............C..............G----.. [1343]

Megaceros .AGA.CCG.....A..............C...............----.. [1343]

Physcomitrella ..T..........A..............C...............----.. [1343]

Anomodon ..T..........A..............C...............----.. [1343]

Treubia .C.C.........A..............C.....T.........----.. [1343]

Marchantia .C...........A..............C.....T.........----.. [1343]

[ 1360 1370 1380 1390 1400]

[ . . . . .]

Huperzia_cox1 TGCTTATGCTGGATGGAATGCCTTTA------------------------ [1369]

Huperzia_cox1_4187_4471 ......C...................------------------------ [66]

Huperzia_cox1_81728_81627 -------------------------------------------------- [0]

Huperzia_cox1_321716_321817 -------------------------------------------------- [0]

Huperzia_cox1_326924_327263 .ATG.TG.AC...AT.CC.T......GTACTTCCGGCCGGCTGCCCATGT [89]

Huperzia_cox1_332216_332361 -------------------------------------------------- [146]

Arabidopsis ......C...............C..T------------------------ [1366]

Zea_perennis ......C..C...........TC.G.------------------------ [1366]

Cycas ......C...............C...------------------------ [1366]

Osmunda_JF681372 ......C.................G.------------------------ [1369]

Phaeoceros ......C..C..G.............------------------------ [1369]

Megaceros ......C..C..G.............------------------------ [1369]

Physcomitrella ......C.................C.------------------------ [1369]

Anomodon ......C...................------------------------ [1369]

Treubia ......C...................------------------------ [1369]

Marchantia ......C...................------------------------ [1369]

[ 1410 1420 1430 1440 1450]

[ . . . . .]

Huperzia_cox1 ---------------------------GTAGTTTCGGCTCATA-----TG [1387]

Huperzia_cox1_4187_4471 ------------------CTTTACTTA............GCC.ATGCT.C [98]

Huperzia_cox1_81728_81627 -------------------------------------------------- [0]

Huperzia_cox1_321716_321817 -------------------------------------------------- [0]

Huperzia_cox1_326924_327263 TTCTGTAGTAGGGGGGCAAAGCCCACCCAGTGGG.....----------- [128]

Huperzia_cox1_332216_332361 -------------------------------------------------- [146]

Arabidopsis ---------------------------CC.....T.....T..-----.A [1384]

Zea_perennis ---------------------------.C........T..T..-----.A [1384]

Cycas ---------------------------.C.........C.T..-----.A [1384]

Osmunda_JF681372 ---------------------------...........C.TC.-----.. [1387]

Phaeoceros ---------------------------............T...-----C. [1387]

Megaceros ---------------------------.......T........-----C. [1387]

Physcomitrella ---------------------------.............G..-----.. [1387]

Anomodon ---------------------------.............G..-----.. [1387]

Treubia ---------------------------.......T........-----.. [1387]

Marchantia ---------------------------.......T........-----.. [1387]

[ 1460 1470 1480 1490 1500]

[ . . . . .]

Huperzia_cox1 TCTCTGTAGTAGGGATTTCTTGTTTCTTT---GTCGTGGTTTTTCTTACT [1434]

Huperzia_cox1_4187_4471 CT...........T....T.C......CC---....C....C.....CT. [145]

Huperzia_cox1_81728_81627 -------------------------------------------------- [0]

Huperzia_cox1_321716_321817 -------------------------------------------------- [0]

Huperzia_cox1_326924_327263 .T..CAACCA...CT...T..C.......GTA..G..TT..C...C..T. [178]

Huperzia_cox1_332216_332361 -------------------------------------------------- [146]

Arabidopsis .A..C.....T.......G.........C---..G..C..AACAA.C... [1431]

Zea_perennis .A..C.....T......CG.C.......C---..A..T..CGCAA.C... [1431]

Cycas .A..C.....T......CG.C.......C---..G..C..AACAA.C... [1431]

Osmunda_JF681372 .TC.....CCG.....C.T.C.......CTAC..G..TT.CAAA---... [1434]

Phaeoceros .T.T...............CC...CT..C---..A..TA......CC..C [1434]

Megaceros .T.................CC..CCT.CC---..A...A..........C [1434]

Physcomitrella .T......A....A....T..........---..A.........T..... [1434]

Anomodon .T...........A....TG.........---..A.........TCC..C [1434]

Treubia .T...........T....T..........---..A............... [1434]

Marchantia .T................T..........---..A............... [1434]

[ 1510 1520 1530 1540 1550]

[ . . . . .]

Huperzia_cox1 TTAACCAGTGAAAACAA------GTGTGCTCCAAGTCCTTGGGCTGTTGA [1478]

Huperzia_cox1_4187_4471 CC...............------........................... [189]

Huperzia_cox1_81728_81627 -------------------------------------------------- [0]

Huperzia_cox1_321716_321817 -------------------------------------------------- [0]

Huperzia_cox1_326924_327263 CC.............G.------.....................--..T. [220]

Huperzia_cox1_332216_332361 -------------------------------------------------- [146]

Arabidopsis ....G.....G...T..CAAAAGA........G............C.... [1481]

Zea_perennis .C..G.....G...G..CAAAAGA.....GGA.................. [1481]

Cycas ....G.....G......CAAAAGA..C...........C........... [1481]

Osmunda_JF681372 ....G......G.....------.......................G... [1478]

Phaeoceros CC....G..........------..A........................ [1478]

Megaceros CC....G..........------..A........................ [1478]

Physcomitrella C................------........................... [1478]

Anomodon C................------.......T................... [1478]

Treubia C....T...........------........................... [1478]

Marchantia .....T...........------........................... [1478]

[ 1560 1570 1580 1590 1600]

[ . . . . .]

Huperzia_cox1 ACAGAATTCAACCACGCTTGAATGGATGGTACAAAGCCCTCCAGCTTTTC [1528]

Huperzia_cox1_4187_4471 .................................................. [239]

Huperzia_cox1_81728_81627 ----------------................G............A.... [34]

Huperzia_cox1_321716_321817 ----------------................G............A.... [34]

Huperzia_cox1_326924_327263 ....G.............C.............G............A.... [270]

Huperzia_cox1_332216_332361 -------------------------------------------------- [146]

Arabidopsis ..T.........T..A..G.................T.....T....... [1531]

Zea_perennis G......C.......A..A......T...................C.... [1531]

Cycas ...T...C.......A.CG............................... [1531]

Osmunda_JF681372 .T..........T.TA..C..........................G.... [1528]

Phaeoceros ............G..AT.....................T......A.... [1528]

Megaceros .T..........G..A.C...........................A.... [1528]

Physcomitrella ............G..A..............CA..........G..A.... [1528]

Anomodon ............G..A..............CA..........G..A.... [1528]

Treubia ............G..A................C................. [1528]

Marchantia ............G..A..............C.C.........G..A.... [1528]

[ 1610 1620 1630 1640 1650]

[ . . . . .]

Huperzia_cox1 ATACCTTTGAAGAACTTCCAGCTATCAAAGAAAG-------CATTGGCAC [1571]

Huperzia_cox1_4187_4471 ...........................G......-------.......CT [282]

Huperzia_cox1_81728_81627 ..................................-------......... [77]

Huperzia_cox1_321716_321817 ..................................-------......... [77]

Huperzia_cox1_326924_327263 ...........................G......-------.......-T [312]

Huperzia_cox1_332216_332361 -------------------------------------------------- [146]

Arabidopsis ....T....G..................G..G.CGAAAAGCT..G----- [1576]

Zea_perennis ....T....G..........A............C-------T.--.AA.. [1572]

Cycas ....T.C................G....G..G.C------CT-.G----- [1569]

Osmunda_JF681372 ....TC....C.......T.........G.....-------....----- [1566]

Phaeoceros ..............A..T.......A........-------T...----- [1566]

Megaceros ..............A..........A........-------T...----- [1566]

Physcomitrella .C..T...TC.........G.T......G.....-------....----- [1566]

Anomodon .C..T...TC.........G.T......G.....-------....----- [1566]

Treubia ....T.......................G.....-------....----- [1566]

Marchantia ....T.......................G.....-------....----- [1566]

[ 1660 1670 ]

[ . . ]

Huperzia_cox1 TTTTCCGCTCCGCCCTCGTTGGACCTAG [1599]

Huperzia_cox1_4187_4471 ...------------------------- [285]

Huperzia_cox1_81728_81627 .........................--- [102]

Huperzia_cox1_321716_321817 .........................--- [102]

Huperzia_cox1_326924_327263 ................T.......T... [340]

Huperzia_cox1_332216_332361 ---------------------------- [146]

Arabidopsis -------------------..A.GTA.- [1584]

Zea_perennis CAA...A----------....---.... [1587]

Cycas -------------------..A------ [1572]

Osmunda_JF681372 -------------------.A.------ [1569]

Phaeoceros -------------------.A.------ [1569]

Megaceros -------------------.A.------ [1569]

Physcomitrella -------------------.A.------ [1569]

Anomodon -------------------.A.------ [1569]

Treubia -------------------.A.------ [1569]

Marchantia -------------------.A.------ [1569]

;

END;

**cox2**

#NEXUS

[MacClade 4.05 registered to Yin-Long Qiu Lab, EEB, University of Michigan]

BEGIN DATA;

DIMENSIONS NTAX=12 NCHAR=850;

FORMAT DATATYPE=DNA MISSING=? GAP=- MATCHCHAR=. INTERLEAVE ;

MATRIX

[ 10 20 30 40 50]

[ . . . . .]

Huperzia_cox2 ATGATTTTGAG------AAACATATGGCTATTTGTTCCAATTGCTTATCG [44]

Huperzia_cox2_268943_268396 ...........------................................. [44]

Huperzia_cox2_380468_380592 -------------------------------------------------- [0]

Huperzia_cox2_112293_112626 -------------------------------------------------- [0]

Huperzia_cox2_259801_259731 -------------------------------------------------- [0]

Vitis_cox2 ......G.TC------T.G.---.........CC.CA........CC.T. [41]

Zea_perennis ......C.TC.TTCATT.G.---...T.G...CC.CA....C...CT.T. [47]

Cycas ......G.T.------T...---.........CC.CA....C....C.T. [41]

Phaeoceros ....A------------.....C..........---.............. [35]

Anomodon ....G.....A------...T.C..........---.......G....T. [41]

Marchantia ....A...---------G.T---....A.....---..........T.T. [35]

Treubia ....A...---------G.T---....AG....TG...........T.T. [38]

[ 60 70 80 90 100]

[ . . . . .]

Huperzia_cox2 TGATGCTGCGGAACCTTGGCAATTAGGATTTCAAGACGCAGCAACACCTA [94]

Huperzia_cox2_268943_268396 .............T......G..G..A.......A.....AA........ [94]

Huperzia_cox2_380468_380592 -------------------------------------------------- [0]

Huperzia_cox2_112293_112626 -------------------------------------------------- [0]

Huperzia_cox2_259801_259731 -------------------------------------------------- [0]

Vitis_cox2 ......A........A.............C.................... [91]

Zea_perennis ...............A.............C.................... [97]

Cycas ...............A.............C.................... [91]

Phaeoceros .........C.........T....G......T...............TA. [85]

Anomodon ...C.............................................. [91]

Marchantia ...........................T.........C.T..C..T.... [85]

Treubia ...........................G.........C.T..C..T.... [88]

[ 110 120 130 140 150]

[ . . . . .]

Huperzia_cox2 TGATGCAAGGAATAATTGACTTGCATCATGATATTTTTTTCTCTTTAATG [144]

Huperzia_cox2_268943_268396 ...C.....................................CT.CC...C [144]

Huperzia_cox2_380468_380592 -----------------------------------------------..T [3]

Huperzia_cox2_112293_112626 ----------------..CA..--..G......-.....C..T.C....T [31]

Huperzia_cox2_259801_259731 -------------------------------------------------- [0]

Vitis_cox2 .A.........G....G.....A.....C.....C.......TCC.C..T [141]

Zea_perennis .............C........A.....C.....C.......TCC.C..T [147]

Cycas .................T....A...........AGC..CT.TCCC...T [141]

Phaeoceros ......................A...................T.C..C.C [135]

Anomodon ......................A...................T......A [141]

Marchantia ......................A...A...............T......C [135]

Treubia ......................A...................T.C...CC [138]

[ 160 170 180 190 200]

[ . . . . .]

Huperzia_cox2 ATTATATTGATCTTCGTTTTATGGATGTTGGTTCGCGCTTTATGGCATTT [194]

Huperzia_cox2_268943_268396 ...G.......T..T..C---............T................ [191]

Huperzia_cox2_380468_380592 ..............TC.A..................A.C.---....... [50]

Huperzia_cox2_112293_112626 ...G.............C...C............................ [81]

Huperzia_cox2_259801_259731 -------------------------------------------------- [0]

Vitis_cox2 C.C..T...G.T.....A.C.C....C....................... [191]

Zea_perennis C.G..T...G.T.....A.C.C............................ [197]

Cycas .....T.C.G.TC....A.C....................C......... [191]

Phaeoceros .....TC.......T..A.C.............T..............C. [185]

Anomodon .....T........T..A................................ [191]

Marchantia G....T........T..A................................ [185]

Treubia G....T........T..A................................ [188]

[ 210 220 230 240 250]

[ . . . . .]

Huperzia_cox2 TCACTATGAAAGAAATCCTATTCCAGAAAGGATTGTTCATGGAACTACTA [244]

Huperzia_cox2_268943_268396 ......GA........G.G............................... [241]

Huperzia_cox2_380468_380592 ...T.GGA...C....T.A............................... [100]

Huperzia_cox2_112293_112626 .....---.T.T......G............................... [128]

Huperzia_cox2_259801_259731 -------------------------------------------------- [0]

Vitis_cox2 C......A...A......A..C..GC....A................... [241]

Zea_perennis CA..G.GC...CT.....A..C..GC........................ [247]

Cycas .......AG..TC.....G..C..GC........................ [241]

Phaeoceros .......A.G........A............................... [235]

Anomodon ....C..A..........A.....G.....A...........G....... [241]

Marchantia .......A..........A............................... [235]

Treubia .....G.A..........A.....G......................... [238]

[ 260 270 280 290 300]

[ . . . . .]

Huperzia_cox2 TAGAGATTATTT----GGACCATTTTTCCTAGTATTATTCTGATGTTTAT [290]

Huperzia_cox2_268943_268396 ....C.......----...TT....C...C...G......C...A..... [287]

Huperzia_cox2_380468_380592 C........C..ATTC.........------------------------- [125]

Huperzia_cox2_112293_112626 ...........C----...TT...C..G.C..-................. [173]

Huperzia_cox2_259801_259731 -------------------------------------------------- [0]

Vitis_cox2 .C......C..C----.......A...........C..C.C......C.. [287]

Zea_perennis .C..A......C----.................G.C....CAT....C.. [293]

Cycas C..........C----.......A................C......C.. [287]

Phaeoceros ............----....T.......T.........CTC......... [281]

Anomodon ............----......................C........... [287]

Marchantia ....A.......----....T........A.................... [281]

Treubia ....A.......----....T........A.................... [284]

[ 310 320 330 340 350]

[ . . . . .]

Huperzia_cox2 TGCTATACCATCTTTTGCCTTATTATATTCAATGGACGAGGTAGTAG--- [337]

Huperzia_cox2_268943_268396 G.......T.CT......T.C.....C.C......T...TAA.....--- [334]

Huperzia_cox2_380468_380592 -------------------------------------------------- [125]

Huperzia_cox2_112293_112626 G.......T.C...C...T.C.......CT.......A..A.G....--- [220]

Huperzia_cox2_259801_259731 -------------------------------------------------- [0]

Vitis_cox2 ............A....TTC.C.....C...................TAG [337]

Zea_perennis ............G.....TC.G.....C..........G....T...TAG [343]

Cycas ............A.....TCCG.....C...................TCA [337]

Phaeoceros ..................T....................A..T....--- [328]

Anomodon ..................T............................--- [334]

Marchantia ...A.....T..G..C...C.TC.T......................--- [328]

Treubia ...A.....T..G......C.TC.T......................--- [331]

[ 360 370 380 390 400]

[ . . . . .]

Huperzia_cox2 ATCCAGCCATTACTATCAAAGCTATTGGACATCAATGGTATTGGACT--- [384]

Huperzia_cox2_268943_268396 ........T...G..G......C........C..........T....ACT [384]

Huperzia_cox2_380468_380592 -------------------------------------------------- [125]

Huperzia_cox2_112293_112626 ...............G.........................C.....--- [267]

Huperzia_cox2_259801_259731 -------------------------------------------------- [0]

Vitis_cox2 ....T....................................C.....--- [384]

Zea_perennis .........................................C.....--- [390]

Cycas ................A........................C....C--- [384]

Phaeoceros .......TG.......................T............TA--- [375]

Anomodon .....A.A...........................C...........--- [381]

Marchantia .......T.......................................--- [375]

Treubia .......T........A..............................--- [378]

[ 410 420 430 440 450]

[ . . . . .]

Huperzia_cox2 ---TATGAATATTCAGACT---ATAACCGTTCTGATGAACAGTCATTAAC [428]

Huperzia_cox2_268943_268396 TAT.......C..TTTG..TACGC..TA.......C......C....... [434]

Huperzia_cox2_380468_380592 -------------------------------------------------- [125]

Huperzia_cox2_112293_112626 ---.....G.C.------.TAC.CC..A.................C.... [308]

Huperzia_cox2_259801_259731 -------------------------------------------------- [0]

Vitis_cox2 ---.....G.....G....---.....A....C......G.....C.C.. [428]

Zea_perennis ---.....G.....G....---.....A....C............C.C.. [434]

Cycas ---.....G..........---.....A....C............C.... [428]

Phaeoceros ---.CCT.T.....C....---.....A....C......T.....C.... [419]

Anomodon ---.....G..........---.....A.................C.... [425]

Marchantia ---.....G..........---.....A.................C.... [419]

Treubia ---.....G..........---.....A.................C.... [422]

[ 460 470 480 490 500]

[ . . . . .]

Huperzia_cox2 CTTTGACAGTTATAT--------GATTCCAGAAGATGACTCAGAATTAGG [470]

Huperzia_cox2_268943_268396 T.....T......T.AGTTATTT.....TG......A.T...T....... [484]

Huperzia_cox2_380468_380592 -------------------------------------------------- [125]

Huperzia_cox2_112293_112626 T...A..........----ATAT.......-------------------- [334]

Huperzia_cox2_259801_259731 -------------------------------------------------- [0]

Vitis_cox2 T.............C--------...............TC.......G.. [470]

Zea_perennis T.............C--------...............TC.......G.. [476]

Cycas T.............C--------...............TC.......G.. [470]

Phaeoceros T..............--------....A.G..G.....G........... [461]

Anomodon T.....T........--------.................T......G.. [467]

Marchantia T..............--------.........G.....T.T......G.. [461]

Treubia T..............--------.........G.....T.T......G.. [464]

[ 510 520 530 540 550]

[ . . . . .]

Huperzia_cox2 TCAATTACGCTTATTAGAAGTGGACAATCGAATGGTTGTACCAGCAAAAA [520]

Huperzia_cox2_268943_268396 .T.......T......A........G.....G.............C..C. [534]

Huperzia_cox2_380468_380592 -------------------------------------------------- [125]

Huperzia_cox2_112293_112626 -------------------------------------------------- [334]

Huperzia_cox2_259801_259731 -------------------------------------------------- [0]

Vitis_cox2 .....C...T..................A..G.............C.... [520]

Zea_perennis .....C...T...........T......A..G.............C.... [526]

Cycas .....C...T.....................G.............C.G.. [520]

Phaeoceros .TC....T.T...........A..T...A..G..A.....T....G.... [511]

Anomodon ......G..............A.........G.A................ [517]

Marchantia .........T.....................G.................. [511]

Treubia .........T..G..................G.................. [514]

[ 560 570 580 590 600]

[ . . . . .]

Huperzia_cox2 CTCATCTACGTATGATTATAACATCTGCTGATGTACTTCATAGTTGGGCT [570]

Huperzia_cox2_268943_268396 .....AC..-.....----------------------------------- [548]

Huperzia_cox2_380468_380592 -------------------------------------------------- [125]

Huperzia_cox2_112293_112626 -------------------------------------------------- [334]

Huperzia_cox2_259801_259731 -------------------------------------------------- [0]

Vitis_cox2 ..A..........T...G.....C............C............. [570]

Zea_perennis .................G.....C.C..........C............. [576]

Cycas .................G...............C..C.......C..... [570]

Phaeoceros .....A.C...C.......C....T...........C.T........... [561]

Anomodon .................................................. [567]

Marchantia ...................T..T........................... [561]

Treubia ......................T........................... [564]

[ 610 620 630 640 650]

[ . . . . .]

Huperzia_cox2 GTACCTTCCTTGGGTGTAAAATGTGATGCTGTACCTGGTCGTTTGAATCA [620]

Huperzia_cox2_268943_268396 -------------------------------------------------- [548]

Huperzia_cox2_380468_380592 -------------------------------------------------- [125]

Huperzia_cox2_112293_112626 -------------------------------------------------- [334]

Huperzia_cox2_259801_259731 -------------------------------------------------- [0]

Vitis_cox2 ..........CA.....C.........................CA..... [620]

Zea_perennis ..........CA.....C.........................CA....T [626]

Cycas ..........CA.....C................................ [620]

Phaeoceros ...........A...........C..........TC...T....A..... [611]

Anomodon .....C.....A..........................C.....A..... [617]

Marchantia ...........A................................A..... [611]

Treubia ........T..A................................A..... [614]

[ 660 670 680 690 700]

[ . . . . .]

Huperzia_cox2 GACTTCCATCTTTATTAAACGAGAAGGAGTTTACTATGGTCAGTGCAGTG [670]

Huperzia_cox2_268943_268396 -------------------------------------------------- [548]

Huperzia_cox2_380468_380592 -------------------------------------------------- [125]

Huperzia_cox2_112293_112626 -------------------------------------------------- [334]

Huperzia_cox2_259801_259731 ---------...C..C.................................A [41]

Vitis_cox2 ...C..T..T.CGG.AC................................. [670]

Zea_perennis ...C.......CGG.AC................................. [676]

Cycas .........T.CGG.GC................................. [670]

Phaeoceros .........T............A.................T.AC...... [661]

Anomodon .........T.................G...................... [667]

Marchantia .........T.................T...................... [661]

Treubia .......T.T.................T...................... [664]

[ 710 720 730 740 750]

[ . . . . .]

Huperzia_cox2 AAATTTGTGGAACCAATCATGCGTTTATGCCTATTGTCGTAGAAGCTGTT [720]

Huperzia_cox2_268943_268396 -------------------------------------------------- [548]

Huperzia_cox2_380468_380592 -------------------------------------------------- [125]

Huperzia_cox2_112293_112626 -------------------------------------------------- [334]

Huperzia_cox2_259801_259731 ..T..........T................-------------------- [71]

Vitis_cox2 .G...C.......T........C.C..C......C............... [720]

Zea_perennis .G...........T........C....C......C...........A..G [726]

Cycas .G...C.......T........C.C..C..G.GCGCC..G.T.CATA.GC [720]

Phaeoceros .....C...........T................................ [711]

Anomodon ..C..........T....................C..T............ [717]

Marchantia ..C..........T.......GC....................G..A... [711]

Treubia .............T........C....................G...... [714]

[ 760 770 780 790 800]

[ . . . . .]

Huperzia_cox2 TCTTTGGATGCTTATGTTTCTCGGGTATCCCATAAATTA----------- [759]

Huperzia_cox2_268943_268396 -------------------------------------------------- [548]

Huperzia_cox2_380468_380592 -------------------------------------------------- [125]

Huperzia_cox2_112293_112626 -------------------------------------------------- [334]

Huperzia_cox2_259801_259731 -------------------------------------------------- [71]

Vitis_cox2 C..AG.A.A.A.....G.............A..C.....ATCCCCCAAAC [770]

Zea_perennis A.....A.A.A.....CGGA.T........A..C.....ATCCTCCAAAC [776]

Cycas C----.AC....G.GCCCA...T..C------.C.GCCGCACCTTCTCGA [760]

Phaeoceros ..........A..................TA..G.....----------- [750]

Anomodon ..........A..........T..A....AA........----------- [756]

Marchantia ..C.......A..........T........A........----------- [750]

Treubia ..C.......A..........T........A........----------- [753]

[ 810 820 830 840 850]

[ . . . . .]

Huperzia_cox2 -GACTGA------------------------------------------- [765]

Huperzia_cox2_268943_268396 -------------------------------------------------- [548]

Huperzia_cox2_380468_380592 -------------------------------------------------- [125]

Huperzia_cox2_112293_112626 -------------------------------------------------- [334]

Huperzia_cox2_259801_259731 -------------------------------------------------- [71]

Vitis_cox2 CA..CA.ACCGGGGAAGCTTAAGCGGAAATGAAAGAGTAGGGTGAGGGAA [820]

Zea_perennis CA...A.------------------------------------------- [783]

Cycas AC.---GGCCAATTTGGTTTAGGTGCGAGCTACCTAAGGAGCTATTATAG [807]

Phaeoceros -......------------------------------------------- [756]

Anomodon -....A.------------------------------------------- [762]

Marchantia -....AG------------------------------------------- [756]

Treubia -....A.------------------------------------------- [759]

;

END;

**cox3**

#NEXUS

[MacClade 4.05 registered to Yin-Long Qiu Lab, EEB, University of Michigan]

BEGIN DATA;

DIMENSIONS NTAX=17 NCHAR=821;

FORMAT DATATYPE=DNA MISSING=? GAP=- MATCHCHAR=. INTERLEAVE ;

MATRIX

[ 10 20 30 40 50]

[ . . . . .]

Huperzia_cox3 ATGAATGTCTCTCA---AAAGCATCCTTATCATTTAGTAGATCCAAGTCC [47]

Huperzia_cox3_29792_30078_End -------------------------------------------------- [0]

Huperzia_cox3_380263_380400_Middle -------------------------------------------------- [0]

Huperzia_cox3_82538_82427_Front -------------------------------------------------- [0]

Huperzia_cox3_320906_321017_Front -------------------------------------------------- [0]

Huperzia_cox3_379357_379534_Front --......G.....AAAG................C............... [48]

Isoetes_HQ616428 ........GC---------G.............................. [41]

Zea_perennis ....T..AA.....---G.G....T..........G.............. [47]

Arabidopsis ....T..AA.....---G.G....T..........G.............. [47]

Cycas ....T.TCA.....---G.G....T.........CG.............. [47]

Megaceros ....G...T...T.---............C.................... [47]

Phaeoceros ....G.......T.---........T........................ [47]

Anomodon ....G.........---................................. [47]

Physcomitrella ....G.........---................................. [47]

Pleurozia ...------.....---...A.......T...............C..C.. [41]

Marchantia ....G.........---...A.......T...............C..C.. [47]

Treubia ....G.........---...A.......T...............C..C.. [47]

[ 60 70 80 90 100]

[ . . . . .]

Huperzia_cox3 ATGGCCTATTTTGGGTTCACTGGGAGCTTTGGCAAGCACTATGGGTGGCG [97]

Huperzia_cox3_29792_30078_End -------------------------------------------------- [0]

Huperzia_cox3_380263_380400_Middle -------------------------------------------------- [0]

Huperzia_cox3_82538_82427_Front ---------------........................C.......... [35]

Huperzia_cox3_320906_321017_Front ---------------........................C.......... [35]

Huperzia_cox3_379357_379534_Front ...A.......CTA.G.T.......TT............C..A....... [98]

Isoetes_HQ616428 ..........C........T........C....G.....C.C...C..T. [91]

Zea_perennis ...........C.........C.............C...CG.A..A..T. [97]

Arabidopsis ...........C.........C.............C...CG.A..A..T. [97]

Cycas ...........C.........C.............C...CG.T..A..T. [97]

Megaceros .....TA............T...................CG.T.....T. [97]

Phaeoceros .C...TA.......T..T.T...................CG.T.....T. [97]

Anomodon .......C...........T...................C..T.....T. [97]

Physcomitrella .......C...........T...................C..T.....T. [97]

Pleurozia .......C.............C.....C..............T.....T. [91]

Marchantia .......C.............C....................T.....T. [97]

Treubia G......T.............C....................T.....T. [97]

[ 110 120 130 140 150]

[ . . . . .]

Huperzia_cox3 TTATGTACATGCACTCTTTTGCGGGAGGTGGAACA-----CTTCTTAGTT [142]

Huperzia_cox3_29792_30078_End -------------------------------------------------- [0]

Huperzia_cox3_380263_380400_Middle -------------------------------------------------- [0]

Huperzia_cox3_82538_82427_Front .........C......C..GC..............-----....C..... [80]

Huperzia_cox3_320906_321017_Front .........C......C..GC..............-----....C..... [80]

Huperzia_cox3_379357_379534_Front ................C..GC.....--....C.TTCGGC.C..C..... [146]

Isoetes_HQ616428 ...............TC...A.......C....TG-----......G... [136]

Zea_perennis .G..............A...CAA..G....C....-----.....C.... [142]

Arabidopsis .G............C.A...CAA..G....C..G.-----.....A.... [142]

Cycas ................A...A.A..G....C....-----.....CG... [142]

Megaceros .............T..C...A..............-----.......... [142]

Phaeoceros ...........T.T..G...A..............-----.......... [142]

Anomodon .......T............AT.............-----........C. [142]

Physcomitrella ....................A..............-----........C. [142]

Pleurozia ...................CA.......C......-----......T... [136]

Marchantia ....................A.......C......-----......T... [142]

Treubia ....................A.A.....C......-----......T... [142]

[ 160 170 180 190 200]

[ . . . . .]

Huperzia_cox3 CAGGCTTGGGAATA---ATCTTATACACCATGTTTTTATGGTGGCGCGAT [189]

Huperzia_cox3_29792_30078_End -------------------------------------------------- [0]

Huperzia_cox3_380263_380400_Middle ------------------------------------..G.......T... [14]

Huperzia_cox3_82538_82427_Front .........A...GATTT.A.....T......------------------ [112]

Huperzia_cox3_320906_321017_Front .........A...GATTT.A.....T......------------------ [112]

Huperzia_cox3_379357_379534_Front ......C.......ATG........T....C.------------------ [178]

Isoetes_HQ616428 .............G---..T.....T..T..................... [183]

Zea_perennis TG...C.---....TTTC..C.T..T........CG..........G... [189]

Arabidopsis TG...C.---C...TTT...C....T........CG.............. [189]

Cycas TG.....---....TTT...CC...T........CG..C........... [189]

Megaceros T......A.....G---...CC...T......A..G.....C.......C [189]

Phaeoceros T......A.....G---...C....T......A..G.............C [189]

Anomodon T............G---...C....T..T......G.............. [189]

Physcomitrella T............G---...C....T..T......G.............. [189]

Pleurozia T............G---........T.........G.............. [183]

Marchantia T............G---........T.........G.............. [189]

Treubia T............G---........T.........G.C............ [189]

[ 210 220 230 240 250]

[ . . . . .]

Huperzia_cox3 GTTATACGTGAATCCACTTACGAAGGACATCATACATTTGTGGTACAATT [239]

Huperzia_cox3_29792_30078_End -------------------------------------------------- [0]

Huperzia_cox3_380263_380400_Middle .......A.A...T.................................... [64]

Huperzia_cox3_82538_82427_Front -------------------------------------------------- [112]

Huperzia_cox3_320906_321017_Front -------------------------------------------------- [112]

Huperzia_cox3_379357_379534_Front -------------------------------------------------- [178]

Isoetes_HQ616428 ..............T..CC.............C..CAAAA.......... [233]

Zea_perennis ...C.............G.TG.....G.........AAA.CT........ [239]

Arabidopsis ...C.............G.TG..............CAAA..C........ [239]

Cycas .................G.TG..............CAAG..C........ [239]

Megaceros ..C....................................A....C..... [239]

Phaeoceros ..C...T......T.........................A....C..... [239]

Anomodon ............................................C..... [239]

Physcomitrella ............................................C..... [239]

Pleurozia ..CG.............C....................C.....C..... [233]

Marchantia .................C..........................C..... [239]

Treubia .................C..........................C..... [239]

[ 260 270 280 290 300]

[ . . . . .]

Huperzia_cox3 AGGACTTCGCTACGGTTTTATTTTGTTCATTGTCTCAGAGGTTATGTCTT [289]

Huperzia_cox3_29792_30078_End -------------------------------------------------- [0]

Huperzia_cox3_380263_380400_Middle ...G.CAA.GGC.....G............CA..C.G.G...C.C..A.C [114]

Huperzia_cox3_82538_82427_Front -------------------------------------------------- [112]

Huperzia_cox3_320906_321017_Front -------------------------------------------------- [112]

Huperzia_cox3_379357_379534_Front -------------------------------------------------- [178]

Isoetes_HQ616428 ............T.....C...........C...........C....T.. [283]

Zea_perennis .....C...A..T....C....C.C.....A.....G..........TCC [289]

Arabidopsis .....C...A..T....C....C.......C..A..G..........TC. [289]

Cycas .........A..T.........CC..C...C..A..G.....C....TCC [289]

Megaceros .........T..T...A.G........T..C..G..T..A..C.C..T.. [289]

Phaeoceros .........T..T...A.G........T.....G.TT..A..C....T.. [289]

Anomodon ............T...A.G...........C..G..T..A..C....T.. [289]

Physcomitrella ............T...A.G...........C..G..T..A..C....T.. [289]

Pleurozia ............T...A.G...C.T.....C..T..T..A..C....T.. [283]

Marchantia ............T...A.A...C.T.....C..T..T..A..C....T.. [289]

Treubia ............T...A.G...C.T.....C..T..T..A..C....T.. [289]

[ 310 320 330 340 350]

[ . . . . .]

Huperzia_cox3 TTCTAGCTTTCTTTTGGGCTTTTTTCCATTCTTCTCTAGCACCTACAGTA [339]

Huperzia_cox3_29792_30078_End -------------------------------------------------- [0]

Huperzia_cox3_380263_380400_Middle ..T......C..C.C.........-------------------------- [138]

Huperzia_cox3_82538_82427_Front -------------------------------------------------- [112]

Huperzia_cox3_320906_321017_Front -------------------------------------------------- [112]

Huperzia_cox3_379357_379534_Front -------------------------------------------------- [178]

Isoetes_HQ616428 ..T....C..T..............T.........T.G............ [333]

Zea_perennis ..T.T.....T..........C..CT.........T.G........G... [339]

Arabidopsis ..T.T.....T..........C..CT.........T.G......G.G... [339]

Cycas ..TCT...C.T.C.C......C...T......C..TCG........G... [339]

Megaceros ..T........C.......C..CC.T..CC..C..T.G.....C..G... [339]

Phaeoceros ..T...........C..........T.........T.G........G... [339]

Anomodon ..T................C.....T.........T.G.....C..G... [339]

Physcomitrella ..T......................T.........T.G........G... [339]

Pleurozia ..T......C....C.........C..........T.G........G..T [333]

Marchantia ..T......................T.........T.G...........T [339]

Treubia .........................T.........T.G........G..T [339]

[ 360 370 380 390 400]

[ . . . . .]

Huperzia_cox3 GATATTGGAGCTATTTGGCCCCCCGAGGGAATTGAGGTGTTAGATCCTTG [389]

Huperzia_cox3_29792_30078_End -------------------------------------------------- [0]

Huperzia_cox3_380263_380400_Middle -------------------------------------------------- [138]

Huperzia_cox3_82538_82427_Front -------------------------------------------------- [112]

Huperzia_cox3_320906_321017_Front -------------------------------------------------- [112]

Huperzia_cox3_379357_379534_Front -------------------------------------------------- [178]

Isoetes_HQ616428 ..G....................AA.A....................... [383]

Zea_perennis ..G..C....G............AA.A..G....G...T........... [389]

Arabidopsis ..G..C....G............AA.A..G........T........... [389]

Cycas ..G..C....G.............A.A..G....G...T...A....... [389]

Megaceros ..G...............T.T..TA..........T.............. [389]

Phaeoceros ..G.................TT.TA..........T.............. [389]

Anomodon ..A............C........A.A........T......A....... [389]

Physcomitrella ..A....................TA.A........T......A....... [389]

Pleurozia ..G..C........C........AA.A..T...TCT..T........... [383]

Marchantia ..G..C.................AA.A..G...TCT..T........... [389]

Treubia ..G..C........C........AA.A..T...TCT..T........... [389]

[ 410 420 430 440 450]

[ . . . . .]

Huperzia_cox3 GGGGATTCCTTTTTTAAATACTCTTATCCTACTTTCATCTGGAGCTGCCG [439]

Huperzia_cox3_29792_30078_End -------------------------------------------------- [0]

Huperzia_cox3_380263_380400_Middle -------------------------------------------------- [138]

Huperzia_cox3_82538_82427_Front -------------------------------------------------- [112]

Huperzia_cox3_320906_321017_Front -------------------------------------------------- [112]

Huperzia_cox3_379357_379534_Front -------------------------------------------------- [178]

Isoetes_HQ616428 ............CC.............T...................... [433]

Zea_perennis ..AA..C...C..C.T.....C.C...T..C.C......C.......... [439]

Arabidopsis ..AA..C......C.T.....C.C...T..C.C......C.......... [439]

Cycas ..AA..C......CCC.....C.....T..C................... [439]

Megaceros ...A.................A.C...T....C......G.....C...A [439]

Phaeoceros ...A.................A.....TT.....CT...G.....C...A [439]

Anomodon ...A.........C.......C.....TT....C.....C.......... [439]

Physcomitrella ...A.........C.......C.....T...................... [439]

Pleurozia ...A.........C.......C.....T...................... [433]

Marchantia ...A.........C.......C.....T...................... [439]

Treubia ...A.........C.......C.....TT....G................ [439]

[ 460 470 480 490 500]

[ . . . . .]

Huperzia_cox3 TAACTTGGGCTCATCATGCTATATTAGCGGGATTAAAACAGCAAGCCGTT [489]

Huperzia_cox3_29792_30078_End -------------------------------------------------- [0]

Huperzia_cox3_380263_380400_Middle -------------------------------------------------- [138]

Huperzia_cox3_82538_82427_Front -------------------------------------------------- [112]

Huperzia_cox3_320906_321017_Front -------------------------------------------------- [112]

Huperzia_cox3_379357_379534_Front -------------------------------------------------- [178]

Isoetes_HQ616428 ...................C........A.....C....G.G....T... [483]

Zea_perennis .......................C.C.....GAAGG..A.A.G...A... [489]

Arabidopsis .......................C.C.....GAA.G..A.A.G...A... [489]

Cycas .......................CCC.....GAA.G....A.....A... [489]

Megaceros ..................................G...G.AG....T... [489]

Phaeoceros ..................................G...G.AG....T... [489]

Anomodon .G..........................T.....C...A.A.....T... [489]

Physcomitrella .G..........................T.....C...A.A.....T... [489]

Pleurozia .......................C.C..A..T........A.....A... [483]

Marchantia .......................C.C..A..T........A.....A... [489]

Treubia .......................C.C..A..T........A.....A... [489]

[ 510 520 530 540 550]

[ . . . . .]

Huperzia_cox3 TACGCTTTAGTAGCTACCATTTGGCTGGCTTTAGTCTTTACAGGGCTTCA [539]

Huperzia_cox3_29792_30078_End ---------------------.........C....A.AC.TT..T....G [29]

Huperzia_cox3_380263_380400_Middle -------------------------------------------------- [138]

Huperzia_cox3_82538_82427_Front -------------------------------------------------- [112]

Huperzia_cox3_320906_321017_Front -------------------------------------------------- [112]

Huperzia_cox3_379357_379534_Front -------------------------------------------------- [178]

Isoetes_HQ616428 ..T..........T...T..C.................C........C.. [533]

Zea_perennis ..............A...G...TA......C....A.CC..T..CT.... [539]

Arabidopsis ..T...............G...TAT.....C....A.....T..CT.... [539]

Cycas C......C..........G...CA......C....A..C..T...T.... [539]

Megaceros .................TG...C............T..C..T........ [539]

Phaeoceros .................TG...C...A........T..C..T........ [539]

Anomodon ..T...................T.......C.......C..C...T.... [539]

Physcomitrella ..T...................T...A...C.......C..C...T.... [539]

Pleurozia .........A........G...TC......C..........T...T.... [533]

Marchantia .........A........G...TC......C..........T...T.... [539]

Treubia .........A........G...TC......C.......C..T...T.... [539]

[ 560 570 580 590 600]

[ . . . . .]

Huperzia_cox3 AGTAATGGAATATGT----AGAAGCTCCTTTCACTATTTCCGATGGTATT [585]

Huperzia_cox3_29792_30078_End ..G.....G......GGAT......A.............A.C.C.A.T.G [79]

Huperzia_cox3_380263_380400_Middle -------------------------------------------------- [138]

Huperzia_cox3_82538_82427_Front -------------------------------------------------- [112]

Huperzia_cox3_320906_321017_Front -------------------------------------------------- [112]

Huperzia_cox3_379357_379534_Front -------------------------------------------------- [178]

Isoetes_HQ616428 ...G...........----......A.....T.................. [579]

Zea_perennis ..G..........TA----CC....A..C.C.........G...A..... [585]

Arabidopsis ..G..........TA----TC....A..C...........G...A..... [585]

Cycas ..G..........CA----TC....A...C........C.G......... [585]

Megaceros ..G............----......A........C.....T......... [585]

Phaeoceros ..G............----......A........A...C.T......... [585]

Anomodon ..G............----......A........G.....T......... [585]

Physcomitrella ..G............----......A..C.....G............... [585]

Pleurozia ..G...T......A.----...G..C..C.................A... [579]

Marchantia ..G...T......A.----...G..C..C.................A... [585]

Treubia ..G...T......A.----...G.....C.................A... [585]

[ 610 620 630 640 650]

[ . . . . .]

Huperzia_cox3 TATGGTTCTACCTTTTTCTT----AGCCACAGGATTTCATGGGTTTCATG [631]

Huperzia_cox3_29792_30078_End .CA....A.....C...A..TATA.............TC...T.C..... [129]

Huperzia_cox3_380263_380400_Middle -------------------------------------------------- [138]

Huperzia_cox3_82538_82427_Front -------------------------------------------------- [112]

Huperzia_cox3_320906_321017_Front -------------------------------------------------- [112]

Huperzia_cox3_379357_379534_Front -------------------------------------------------- [178]

Isoetes_HQ616428 .................TC.----......G.....C.....T....... [625]

Zea_perennis ....................----...A..T..C........T....... [631]

Arabidopsis ....................----...A.....C........T....... [631]

Cycas ................C..C----...A..T..G........T....... [631]

Megaceros .................T..----...T..G..T.C......T....... [631]

Phaeoceros .......T........CT.C----...T.TG..T........T....... [631]

Anomodon ....................----.........G........T....... [631]

Physcomitrella ....................----......T..G........T....... [631]

Pleurozia ...........G.....T..----...T.....G........T....... [625]

Marchantia ...........G.....T..----...T.....G........T....... [631]

Treubia ........C..A.....T..----...T..G..G........T....... [631]

[ 660 670 680 690 700]

[ . . . . .]

Huperzia_cox3 TTATTATAGGTACTATTTTTCCAATAACATGTGGTATTCGTCAATATTTG [681]

Huperzia_cox3_29792_30078_End CC.................C....C..T..............G....... [179]

Huperzia_cox3_380263_380400_Middle -------------------------------------------------- [138]

Huperzia_cox3_82538_82427_Front -------------------------------------------------- [112]

Huperzia_cox3_320906_321017_Front -------------------------------------------------- [112]

Huperzia_cox3_379357_379534_Front -------------------------------------------------- [178]

Isoetes_HQ616428 ..............T......T.....TC................T.... [675]

Zea_perennis .G............C....CTTG..CGT............C......C.T [681]

Arabidopsis .G............C....CTTG..T.T............G......C.T [681]

Cycas .G................CCT....C.T............C......C.. [681]

Megaceros C....G.G.....C.....CTT..C................G.....C.. [681]

Phaeoceros .....G.G.....C.....CTT.....T..........T..G.....C.. [681]

Anomodon .............C.....C.T.....T...C.C.............C.A [681]

Physcomitrella .C...........C.....C.T.....T...C.C.............C.A [681]

Pleurozia .C.................CTT.....T......G............C.. [675]

Marchantia .C.................CTT.....T......G............C.. [681]

Treubia .C.................C.T.....T......G............C.. [681]

[ 710 720 730 740 750]

[ . . . . .]

Huperzia_cox3 GGTCATTTCACCCAAAAGCATCACTTTGGCTTTGAAGCAGCTGCTTGGTA [731]

Huperzia_cox3_29792_30078_End ..C....C...........G..........................A... [229]

Huperzia_cox3_380263_380400_Middle -------------------------------------------------- [138]

Huperzia_cox3_82538_82427_Front -------------------------------------------------- [112]

Huperzia_cox3_320906_321017_Front -------------------------------------------------- [112]

Huperzia_cox3_379357_379534_Front -------------------------------------------------- [178]

Isoetes_HQ616428 ........G...G..................................... [725]

Zea_perennis ......C.G...A.G.........G...................A..... [731]

Arabidopsis ......C.G..GA.GG........G...................A..... [731]

Cycas ........G...A.GG........G.......C...........A..... [731]

Megaceros ........T..TG.............C.....................C. [731]

Phaeoceros ........T..TG..................................... [731]

Anomodon ..G.....T.......C................................. [731]

Physcomitrella ..G.....T.......C................................. [731]

Pleurozia ........T....CG.......................T.......TT.. [725]

Marchantia ........T....C................................TT.. [731]

Treubia ........T....C................................TT.. [731]

[ 760 770 780 790 800]

[ . . . . .]

Huperzia_cox3 CTGGCATTTTGTAGACGTGGTTTGGT----TATTCTCATTTGTCTCTATT [777]

Huperzia_cox3_29792_30078_End ..................A...C...CGGT....T......-----.... [274]

Huperzia_cox3_380263_380400_Middle -------------------------------------------------- [138]

Huperzia_cox3_82538_82427_Front -------------------------------------------------- [112]

Huperzia_cox3_320906_321017_Front -------------------------------------------------- [112]

Huperzia_cox3_379357_379534_Front -------------------------------------------------- [178]

Isoetes_HQ616428 T........C................----......T......A.....C [771]

Zea_perennis ......................C...----.....C.............. [777]

Arabidopsis ..................A.......----.....CT............C [777]

Cycas .C.......C............C...----C....CT.......C....C [777]

Megaceros .C.......C..G..T......C...----....TCTGC.CC.AC.C... [777]

Phaeoceros ............G..T..........----....TCTG...T.A...... [777]

Anomodon .C..........T.............----.....CT...C..A..C... [777]

Physcomitrella .C..........T.............----.....CT......A...... [777]

Pleurozia T...........C..T..T..A...C----.T..TCTC.....T.....A [771]

Marchantia T...........T..T..T..A...C----.T..TCTC.....T.....A [777]

Treubia T...........T..T..T..A...C----.T..TCTC.....T.....A [777]

[ 810 820]

[ . . ]

Huperzia_cox3 TATTGGTGGGGTGGTCATCAA [798]

Huperzia_cox3_29792_30078_End .C..CA....-...------- [287]

Huperzia_cox3_380263_380400_Middle --------------------- [138]

Huperzia_cox3_82538_82427_Front --------------------- [112]

Huperzia_cox3_320906_321017_Front --------------------- [112]

Huperzia_cox3_379357_379534_Front --------------------- [178]

Isoetes_HQ616428 ...........C.....CT.. [792]

Zea_perennis ...........A...ATATG. [798]

Arabidopsis ...........A...ATATG. [798]

Cycas ...........A....TATG. [798]

Megaceros C..C..C....A...A..T.. [798]

Phaeoceros ...........A...A..T.. [798]

Anomodon ...........A...A..... [798]

Physcomitrella ...........A...A..T.. [798]

Pleurozia ...........C..GA..T.G [792]

Marchantia ...........C..AA..T.G [798]

Treubia ...........C..AA..T.G [798]

;

END;

**nad1**

#NEXUS

[MacClade 4.05 registered to Yin-Long Qiu Lab, EEB, University of Michigan]

BEGIN DATA;

DIMENSIONS NTAX=12 NCHAR=1002;

FORMAT DATATYPE=DNA MISSING=? GAP=- MATCHCHAR=. INTERLEAVE ;

MATRIX

[ 10 20 30 40 50]

[ . . . . .]

Huperzia_nad1 ACGAGACCGTATATCCTTGGTATCTTAGCTAAAATACTTGGAATAATAAT [50]

Huperzia_nad1_129448_129695 -------------------------------------------------- [0]

Isoetes ........A......A.....G.........G.G.G.C............ [50]

Zea_perennis_nad1 ...------..C---A.A.C.G.TCC...GG........T.TT....TC. [41]

Arabidopsis_nad1 ...------..C---A.A.C.G.TCC....G................TC. [41]

Cycas ...------..C---A...C...T.C....G......C.........C.. [41]

Megaceros .T..A..TT......A.......T.....A...............G.... [50]

Phaeoceros .T..A..TT......A.......T.....G...............G.... [50]

Anomodon .T.....T.......A.......T.....G..............C..... [50]

Physcomitrella .T.....T.......A.......T.....G.................... [50]

Treubia .T..A.ATT...C.AA.....C.TG.C.................TC.... [50]

Marchantia .T....ATT...C.AA.....C.TG.C.....G...........T..... [50]

[ 60 70 80 90 100]

[ . . . . .]

Huperzia_nad1 ACCACTTTCACCAGGAGTAGCTTTTTCAGTTTCAGCTGAACGTAAAGTAA [100]

Huperzia_nad1_129448_129695 -------------------------------------------------- [0]

Isoetes .....C.C.................CGC............T.C....... [100]

Zea_perennis_nad1 .......CT..T.........C....T...GCT................. [91]

Arabidopsis_nad1 .......CT..T.........C....T...GCT................. [91]

Cycas .......C...T.........C....T...GCTG................ [91]

Megaceros .....C..T..T.........CC..CT.............T......... [100]

Phaeoceros .....C..T..T.........CC..CT.............T......... [100]

Anomodon ...C....T..T.........C..C.T....CT...C.........A... [100]

Physcomitrella ...C....T..T.........C..C.T....CT................. [100]

Treubia ...C..G.T..TG..C..C..G..C.T...GCT...G.....A.....C. [100]

Marchantia ...C..G.T..TG..T..C..G..C.T...ACT...A.....A.....C. [100]

[ 110 120 130 140 150]

[ . . . . .]

Huperzia_nad1 TGGCTTCTATGCAACGTAGAAAGGGTCCTAATGTAGTAGGATTGTTTGGG [150]

Huperzia_nad1_129448_129695 ------------------------------------------...C...A [8]

Isoetes C....C.C.C.T.......G........CG.............CCGG..A [150]

Zea_perennis_nad1 ......T.G........C...........G.......G....C...C..A [141]

Arabidopsis_nad1 ......T.G....................G.......G....C...C..A [141]

Cycas .....C..G........C...........G.......T....C...C..A [141]

Megaceros C...C......................T..................C..A [150]

Phaeoceros C...C....C.................T..................C..A [150]

Anomodon .....................................G...........A [150]

Physcomitrella .....................................G...........A [150]

Treubia ....G.........A.A.....A..A..G..C.....C..CA.T..A..A [150]

Marchantia ....G.........A.A.....A..A..G..C.....C..CA.T..A..A [150]

[ 160 170 180 190 200]

[ . . . . .]

Huperzia_nad1 TTGTTACAACCTCTAGCAGATGGTTTTAAATTAATTATAAAAGAACCTAT [200]

Huperzia_nad1_129448_129695 ............T........A...CGG..--G.AG.A....A..TTG.- [55]

Isoetes ....C.T..T..TC...........CG....C....C............. [200]

Zea_perennis_nad1 ................................G...C............. [191]

Arabidopsis_nad1 .........................CG.....G...C............. [191]

Cycas ............T....G.......CG.....GG..C.G........... [191]

Megaceros .C.........CT.............G........G..........T... [200]

Phaeoceros .C.........CT.............G........G..........T... [200]

Anomodon ............T.............G........G.............. [200]

Physcomitrella ............T.............G........G.............. [200]

Treubia C............................GC.CG.G........G..C.. [200]

Marchantia ............T.............G..GC.C..G........G..C.. [200]

[ 210 220 230 240 250]

[ . . . . .]

Huperzia_nad1 TTTACCAAGTAGTGCTAATTTA--------TTCATTTTTTCAATGGCTCC [242]

Huperzia_nad1_129448_129695 -------.............A.ATCGTTAC...G...C.C.......... [98]

Isoetes ......G..............C--------CCTCC.CC.C...C.....T [242]

Zea_perennis_nad1 ..C..................C--------.C.C.....AG......... [233]

Arabidopsis_nad1 ..C..................T--------...C.....AG......... [233]

Cycas ..C...................--------..TC...CCAG......... [233]

Megaceros .C....G...............--------..T....C.AT......... [242]

Phaeoceros .C...TG...............--------..T....C.AT.......T. [242]

Anomodon ......................--------..T.....CCT......... [242]

Physcomitrella ......................--------..T......AT......... [242]

Treubia ...G..T.....C..G...C.T--------.........CT......A.. [242]

Marchantia ...G..T.....C..G...A.T--------..T......CT......A.. [242]

[ 260 270 280 290 300]

[ . . . . .]

Huperzia_nad1 AGTAATTACATTTACGTCAAGTTTGGTTGCTCGGGCTGTTACACCTTTCG [292]

Huperzia_nad1_129448_129695 CA.........C...........C.................T.....C.. [148]

Isoetes .A.........C..T....G................C..C.T.T..C.T. [292]

Zea_perennis_nad1 ...GGC........T..T....C....C...T....C...GT......T. [283]

Arabidopsis_nad1 ...GGC........T..T....C....C........C...GT......T. [283]

Cycas ...GG......C..T........C............C...GT......T. [283]

Megaceros ..............T........C.......T.........T......T. [292]

Phaeoceros ..............T........C.......T.........T..T...T. [292]

Anomodon ......G.......T..T.............T.........T......T. [292]

Physcomitrella ..............T..T.............T.........T......T. [292]

Treubia C..TC.G..G.....A.TGGC....TGC...T....C..G.TC....... [292]

Marchantia C..TC.G..G.....ACTGGC....TGC...T.......G.TC....... [292]

[ 310 320 330 340 350]

[ . . . . .]

Huperzia_nad1 ATTATGGTATGGTATTGTCAGATTCAAAT---GTAGGTATACTTTATTTA [339]

Huperzia_nad1_129448_129695 .............C.G.C...........AAT.................G [198]

Isoetes ............C...AC......TC...---.......C....C..... [339]

Zea_perennis_nad1 .......................C.G..C---A....GC..........G [330]

Arabidopsis_nad1 .......................C.G..C---A....GC..........G [330]

Cycas ...............C.C.......GG.C---A...........C..C.G [330]

Megaceros .............................---............C..C.. [339]

Phaeoceros .............................---............C..C.. [339]

Anomodon ........................T....---...............C.. [339]

Physcomitrella ........................T....---...............C.. [339]

Treubia .............T..T............---..G..GG.TT.G...C.. [339]

Marchantia .......C.....T..T.......T....---A.T..GG.TT.G...C.. [339]

[ 360 370 380 390 400]

[ . . . . .]

Huperzia_nad1 TTTGCTATTTCTTCTCTAGGTGTTTATGGAATTATTACGGCGGGTTGGTC [389]

Huperzia_nad1_129448_129695 C..T..-----.....C..........A.G.................... [243]

Isoetes ..C..C...C...T..C............................C..C. [389]

Zea_perennis_nad1 .....C..A.....G.................A....TA..A........ [380]

Arabidopsis_nad1 .....C..A.....G......................TA......C.... [380]

Cycas .....C..A.....G.........C....G........A........... [380]

Megaceros ..C.....A....................G..........T....C..GT [389]

Phaeoceros ..C.....A..C.................G..........T....C..GT [389]

Anomodon ........A...........C..G.....T........A..A..C..... [389]

Physcomitrella ........A...........C........T........A.....C..... [389]

Treubia .....G..A.....A..C..A..G.................A..C...G. [389]

Marchantia .....G..A.....A..T..A..G.................A..C...G. [389]

[ 410 420 430 440 450]

[ . . . . .]

Huperzia_nad1 TAGTAATTCTAAATATGCTTTTTCAGGAGCATTACGATCTGCAGCTCAAA [439]

Huperzia_nad1_129448_129695 .....--------------------------------------------- [248]

Isoetes .......C.C.....C..........................G...T... [439]

Zea_perennis_nad1 .........G........C...CT.......................... [430]

Arabidopsis_nad1 .........G........C...CT.......................... [430]

Cycas .......C.G............C........................... [430]

Megaceros .......C.A........G.C...........C.T...........T... [439]

Phaeoceros .......CTA.......TG.C...........C.T...T.....T.T... [439]

Anomodon C.....................CT.......................... [439]

Physcomitrella C......................T.......................... [439]

Treubia A.....C..C..G..........TG.................C....... [439]

Marchantia A.....C..C..G..........TG.................C....... [439]

[ 460 470 480 490 500]

[ . . . . .]

Huperzia_nad1 TGGTTTCTTATGAAGTCTCTATTGGTCTTATTATTATTACTGTACTAATC [489]

Huperzia_nad1_129448_129695 -------------------------------------------------- [248]

Isoetes ...................C........C.............C.T..... [489]

Zea_perennis_nad1 ....C...........................C................A [480]

Arabidopsis_nad1 ....C...........A...............C................A [480]

Cycas ....C.....C....CA..........C............C....C...A [480]

Megaceros C....CT.........T..........CC...............TC.... [489]

Phaeoceros C....CT.........T..........CC...............T..... [489]

Anomodon ................T.................C.....C.....G..T [489]

Physcomitrella ................T.....C..........................T [489]

Treubia .......C..C.....T..C..A..AT.GC..C.G..AT.C..CA..C.A [489]

Marchantia .......C..C.....T..C..A..AT.GC..C.G..AT.C..CA..C.A [489]

[ 510 520 530 540 550]

[ . . . . .]

Huperzia_nad1 CGTGTAGGTTCTTGCAATTTCAGTGAGATTGTCATAGCATAAAAGCAAAT [539]

Huperzia_nad1_129448_129695 -------------------------------------------------- [248]

Isoetes T.............T....................C..GC.G.....G.. [539]

Zea_perennis_nad1 T........C.C..T....CG..............G..GC.......G.. [530]

Arabidopsis_nad1 T........C.CC.T....CG..............G..GC.......G.. [530]

Cycas T........C..C.T.....G..............G..GC.......G.. [530]

Megaceros .........C..C.T...C.T..............G..GC.......G.. [539]

Phaeoceros T...........C.T.....T.................G......T.G.. [539]

Anomodon T...........C.T..C..T...........A..C..GC.......G.. [539]

Physcomitrella T.......C.....T.....T...........A..C..GC.......G.. [539]

Treubia T.C.C...............T..C.........C.C..GC...CA.G... [539]

Marchantia T.C.C...............T..C.........C....GC...CA.GG.. [539]

[ 560 570 580 590 600]

[ . . . . .]

Huperzia_nad1 ATGGTTTGGCATTCCCTTGTTTCCTGTATTGATTATGTTTTTTATTTCTC [589]

Huperzia_nad1_129448_129695 -------------------------------------------------- [248]

Isoetes .....................C......C..........C........CT [589]

Zea_perennis_nad1 .....CC..T...........C..C......G.......CC.....C... [580]

Arabidopsis_nad1 .....C...T......C....C.........G.......CC......... [580]

Cycas .........T...........C..C......G........C....CC... [580]

Megaceros ......C........T..........................C....... [589]

Phaeoceros ......C........T........A.................C......T [589]

Anomodon ........CTGCG.................C........C..C......T [589]

Physcomitrella ..............................T........C.........T [589]

Treubia ........TTT................G..TC.................T [589]

Marchantia ........TTT................G..TC.................T [589]

[ 610 620 630 640 650]

[ . . . . .]

Huperzia_nad1 GTCTAGCAGAAACTAATCGGGCCCCGTTTGATCTACCAGAAGCAGAAGCT [639]

Huperzia_nad1_129448_129695 -------------------------------------------------- [248]

Isoetes .................A....T........................... [639]

Zea_perennis_nad1 .................A.A..T....C......C........G...... [630]

Arabidopsis_nad1 ...................A..T...........C........G...... [630]

Cycas ...C...............A..T..T.C......C........G...... [630]

Megaceros .................A....TTTT.C.....C...............G [639]

Phaeoceros ............T....A....TTTT.......C..T.....T......G [639]

Anomodon ..T..........G.....A..T..T......T................G [639]

Physcomitrella ..T................A..T..T......T................. [639]

Treubia ..T................A..T..T..............G..C..G..A [639]

Marchantia ..T................A..T..T..............G..C..G..A [639]

[ 660 670 680 690 700]

[ . . . . .]

Huperzia_nad1 GAATTAGTCGCGGGCTATAATGTGGAATATTCTTCAATGGGGTTTGCTCT [689]

Huperzia_nad1_129448_129695 -------------------------------------------------- [248]

Isoetes ........T..........................C........C..... [689]

Zea_perennis_nad1 ....C...T..A...........A...................C...... [680]

Arabidopsis_nad1 ....C...T..A...........A...................C...... [680]

Cycas ....C...T.............C....................C.....C [680]

Megaceros ....C..................A......C..CT..............C [689]

Phaeoceros ....C..................A.......T..T..............C [689]

Anomodon ....C...G..............A...........G.....T......T. [689]

Physcomitrella .......................A...........G.....T........ [689]

Treubia ...C.C..A........C..............G................. [689]

Marchantia ...C.C..A........C.....A........G..C.............. [689]

[ 710 720 730 740 750]

[ . . . . .]

Huperzia_nad1 TTTTTTTTCAGGTGAGTATGCTAATACGATCTTAATGAGTAGTCTATGTA [739]

Huperzia_nad1_129448_129695 -------------------------------------------------- [248]

Isoetes .......CT.................T............C........C. [739]

Zea_perennis_nad1 ........TG..A........C....T............CG.......C. [730]

Arabidopsis_nad1 ........TG..A........C....T.............G...C...C. [730]

Cycas ...C.CCC....G........C....T.................C..... [730]

Megaceros CCC..CCCT...A.............T...T................... [739]

Phaeoceros .C.C.C..T...A.............T...T................... [739]

Anomodon G.......T...G..A..........T....................... [739]

Physcomitrella ........T...G..A..........T....................... [739]

Treubia ......C.T...G.............T....................... [739]

Marchantia ........T...G.............T....................... [739]

[ 760 770 780 790 800]

[ . . . . .]

Huperzia_nad1 CATTGCTTTTTTTGGGAGGTTGGCTGCCCATCCTAGATATTCCTATTCT- [788]

Huperzia_nad1_129448_129695 -------------------------------------------------- [248]

Isoetes .......C.....A..............---..CTC..TC.T.C...T.C [786]

Zea_perennis_nad1 ...C...C...CCA..........C...T.........C....C...T-- [778]

Arabidopsis_nad1 .....T.C...CCA..........C...T.........C....C...T-- [778]

Cycas .......C.C.CCA......C...C...T....C....C....C...T-- [778]

Megaceros ......CC.C...A..........C...T....C....T........T-- [787]

Phaeoceros .......CC....A..G.......C...T...TC....T.C......T-- [787]

Anomodon ...........C.A..............T..................T-- [787]

Physcomitrella .......C...C.A.................................T-- [787]

Treubia ..........CC....G.................G............T-- [787]

Marchantia ...........C.A....................G............T-- [787]

[ 810 820 830 840 850]

[ . . . . .]

Huperzia_nad1 --CCGGGT-GATTCCGGGCTCGATCTGGTTCAGTATCAAGGTTCTTCTCT [835]

Huperzia_nad1_129448_129695 -------------------------------------------------- [248]

Isoetes TT..AA..-..........C..........TG.............CT.T. [835]

Zea_perennis_nad1 --..AA.AA...C..TT.T...........T.................T. [826]

Arabidopsis_nad1 --T.AA.AA...C.................T................... [826]

Cycas --..AA.AA...C.................T...........GA..T... [826]

Megaceros --T..A.GC....T.......T..T.....................T... [835]

Phaeoceros --T..A.GG....T.......T..T...................C.T... [835]

Anomodon --T.TATGT.........T.....TC....T...............T... [835]

Physcomitrella --T.CATGT.........T..T..T.....T...............T... [835]

Treubia --TAC.TGGA...........T........T...............T... [835]

Marchantia --T.CA.GTA...........T........T.....A.........T... [835]

[ 860 870 880 890 900]

[ . . . . .]

Huperzia_nad1 TTCTGTTTGTATATATATGGGTCCGTGCGGCATTTCCACGATATCGTTAT [885]

Huperzia_nad1_129448_129695 -------------------------------------------------- [248]

Isoetes ..T.........................A.........T........... [885]

Zea_perennis_nad1 ........C...................T..................... [876]

Arabidopsis_nad1 .......CC...................A..................... [876]

Cycas .C.C....C...................A..................... [876]

Megaceros ...C.CCCA..C.....C..........A.........A........... [885]

Phaeoceros ..TC...CA..C.....C.....T....A......T..A........... [885]

Anomodon ..T...................T.....A..................... [885]

Physcomitrella .................................................. [885]

Treubia ........T................C..A..................... [885]

Marchantia .........................C..A..................... [885]

[ 910 920 930 940 950]

[ . . . . .]

Huperzia_nad1 GATCAATTAATGAGACTTGGCTGGAAAGTATTTTTGCCTTTATCATTAGC [935]

Huperzia_nad1_129448_129695 -------------------------------------------------- [248]

Isoetes .............................C..C......C.C.....G.. [935]

Zea_perennis_nad1 ............G........C.......G..C......C.......... [926]

Arabidopsis_nad1 ............G........C.......G..C......C.......... [926]

Cycas .......C....G...C....C.......C..C......C.....C.... [926]

Megaceros .......C........................C................. [935]

Phaeoceros ...T...C........................C................. [935]

Anomodon ..C.....G.....G.....T...........C..A.............. [935]

Physcomitrella ..C...........G.....T...........C..A.............. [935]

Treubia .............................C..C..............G.. [935]

Marchantia .............................C..C................. [935]

[ 960 970 980 990 1000]

[ . . . . .]

Huperzia_nad1 ATGGGTAGTCTTTGTATCTGGTGTTCTAATAACCTTTGACTGGCTTCCTC [985]

Huperzia_nad1_129448_129695 -------------------------------------------------- [248]

Isoetes ..........C....C..C......T...G.G................CT [985]

Zea_perennis_nad1 TC........CCC..T..A......TC.G.C......CGA.....C...T [976]

Arabidopsis_nad1 TC........CC...T......C..T..G.C......C.A.........T [976]

Cycas T..........CC..T..........C.G.C......C.A.....C...T [976]

Megaceros .C....................A...C.G..G.............C..GT [985]

Phaeoceros ......................A...C.G..G.............C..G. [985]

Anomodon T........T..................G..G.............C...T [985]

Physcomitrella T........T..................G..G.............C...T [985]

Treubia ............C...............G..G.......A.....C...T [985]

Marchantia ...............T............G..G.......A.........T [985]

[ ]

[ ]

Huperzia_nad1 GA [987]

Huperzia_nad1_129448_129695 -- [248]

Isoetes A. [987]

Zea_perennis_nad1 A. [978]

Arabidopsis_nad1 A. [978]

Cycas A. [978]

Megaceros A. [987]

Phaeoceros A. [987]

Anomodon A. [987]

Physcomitrella A. [987]

Treubia A. [987]

Marchantia A. [987]

;

END;

**nad3**

#NEXUS

[MacClade 4.05 registered to Yin-Long Qiu Lab, EEB, University of Michigan]

BEGIN DATA;

DIMENSIONS NTAX=14 NCHAR=383;

FORMAT DATATYPE=DNA MISSING=? GAP=- MATCHCHAR=. INTERLEAVE ;

MATRIX

[ 10 20 30 40 50]

[ . . . . .]

Huperzia_nad3 ATG---GAATTTGCACCTATTTGTGTCTATTTAGTAATCAGTTTGCTACT [47]

Huperzia_nad3_128097_128416 -..---...............C.........C...G.............. [46]

Huperzia_nad3_235967_235728 -..---...............C.........C.................. [46]

Huperzia_nad3_63988_64073 --------........T..............C.............TG.T. [42]

Isoetes ...---....CC.G.......C.....C...C...G.............. [47]

Vitis ...TCA..................A..........G......CC....G. [50]

Zea_perennis ...TCG..................A..........G......CC....G. [50]

Cycas ...TTG...............C..A..................C....G. [50]

Megaceros .C.---.......T.............................C...... [47]

Phaeoceros .C.---.......T.............................C...... [47]

Physcomitrella ...---................................A........... [47]

Anomodon ...---........................C.......A.........T. [47]

Marchantia ...---................T........................... [47]

Treubia ...---................C........................... [47]

[ 60 70 80 90 100]

[ . . . . .]

Huperzia_nad3 TTCTTTGATCTCAATTGGTGTTTCTCTTTTATTTGCTTCTTCT---TCTG [94]

Huperzia_nad3_128097_128416 ..TG---...CTC............T...C........T.C..---.--- [87]

Huperzia_nad3_235967_235728 ..TG---...CTC..........T..............T..AC---.--- [87]

Huperzia_nad3_63988_64073 ..T...T...CT.................CT.GA.G.......---.--- [86]

Isoetes C.TCCC...................TCC.C..CC..CC..G.CCCA..CA [97]

Vitis ......A...C..C.C......C..T..CC.........CA----A.--A [94]

Zea_perennis .........TC..C.C......C..T..CC.........CA----A.--A [94]

Cycas .C........C..C........C..T..CC...C...C.CA----A.--A [94]

Megaceros .C..C......T..........................TCC..---..CA [94]

Phaeoceros ..T.C......T.............T.C..........TC...---..CA [94]

Physcomitrella ...........T...C........CT..C..............---...A [94]

Anomodon ...........T...C........CT..C..............---...A [94]

Marchantia ...........T.............T.................---..CA [94]

Treubia ...........T.............T.................---..CA [94]

[ 110 120 130 140 150]

[ . . . . .]

Huperzia_nad3 GTTCGGCGTATCCAGAGA-------AATTGTCAGCTCACGAATGTGGTTT [137]

Huperzia_nad3_128097_128416 C..T...T.--...A.TGAGGAATT.......C...T.G........... [135]

Huperzia_nad3_235967_235728 ..GT...T......A.TGGGATGTC.GC.TA.----T............. [133]

Huperzia_nad3_63988_64073 -------------------------------------------------- [86]

Isoetes ...---.T..........-------..CC.C...........C......C [137]

Vitis .....A.C........A.-------.......G..CT............. [137]

Zea_perennis .....A.C........A.-------.......G..CT............C [137]

Cycas .....A.T..........-------.......G..C.............C [137]

Megaceros .......T..........-------........................C [137]

Phaeoceros ...T...T..........-------........................C [137]

Physcomitrella A......T..........-------...........T.......C..G.. [137]

Anomodon A......T.....G....-------...........T.......C..... [137]

Marchantia ...T...T..........-------...........T............. [137]

Treubia .......T........A.-------...........T............. [137]

[ 160 170 180 190 200]

[ . . . . .]

Huperzia_nad3 TGATCCTTTTGAT--GATGCCAGAAGTCGTTCCGATATAAGATTTTATCC [185]

Huperzia_nad3_128097_128416 ......C....G.--........C.AAA..AGT.GGGGCT.GC.G.---- [179]

Huperzia_nad3_235967_235728 .....T.......AT................TT..G.C..A.......T. [183]

Huperzia_nad3_63988_64073 -------------------------------------------------- [86]

Isoetes C..C....C....--.................T....C....CCCC..T. [185]

Vitis C........C.G.--................TT......C.........T [185]

Zea_perennis C.....C.CC.G.--................T.......C.......... [185]

Cycas C.......CC.G.--................T.......C...C.C.... [185]

Megaceros C.......C....--................TT......T..C.C....T [185]

Phaeoceros C.......C....--....T...........TT......T....C...TT [185]

Physcomitrella .............--................TT......C.........T [185]

Anomodon .........C...--................T.......C.........T [185]

Marchantia .............--.....T..........TT......C.........T [185]

Treubia .............--................T.......C.........T [185]

[ 210 220 230 240 250]

[ . . . . .]

Huperzia_nad3 TGTTTCTATTTCATCCATTACATTCGATTTGGAAGTCACCTTTTCATT-- [233]

Huperzia_nad3_128097_128416 ....C........C.T........TT.........C.......CTC..AA [229]

Huperzia_nad3_235967_235728 C.....C........T.......CT..........C..T.C...TC..-- [231]

Huperzia_nad3_63988_64073 -------------------------------------------------- [86]

Isoetes C...C.C.C.C....T.......C....CC..........CC......-- [233]

Vitis ......A....T..TT....TTCCT...CC......A........C..-- [233]

Zea_perennis G...C......T..TT....TCCCT..CCC...............T..-- [233]

Cycas G...C.........T.....TC.CT...CC......A........T..-- [233]

Megaceros ....CT..C......T....T..C...C.C...........C......-- [233]

Phaeoceros ....CT.........T....T..C...C.............C..T...-- [233]

Physcomitrella C..........T..TT....T...T.......................-- [233]

Anomodon C.........CT..T.....T.......CC..............T...-- [233]

Marchantia ...........T..TT....T...T...................T...-- [233]

Treubia C..........T..TT..A.T...T..............T....T...-- [233]

[ 260 270 280 290 300]

[ . . . . .]

Huperzia_nad3 -----TCCTTGGGCAATCTCTCTTAA-CAAGATTGGTTTGTTT---GGAT [274]

Huperzia_nad3_128097_128416 TTATT.A............T..C...A....G...A..G....---.... [276]

Huperzia_nad3_235967_235728 -----....C....------------------------------------ [240]

Huperzia_nad3_63988_64073 -------------------------------------------------- [86]

Isoetes -----...C.....C..T....C...-..G......---....CGG..G. [274]

Vitis -----..........G.AC...CC..-........A.CC....---.... [274]

Zea_perennis -----..........G.AC...C...-........A.C.....---.... [274]

Cycas -----...CC.....G..C...CC..-........A.CC....---.... [274]

Megaceros -----..........G.TC...C...-................---.... [274]

Phaeoceros -----..........G.T....C...-................---.... [274]

Physcomitrella -----..........G.T........-................---.... [274]

Anomodon -----..........G.T........-...A............---.... [274]

Marchantia -----..........G.T........-T...............---.... [274]

Treubia -----..........G.T.....C..-................---.... [274]

[ 310 320 330 340 350]

[ . . . . .]

Huperzia_nad3 TTTGGTCTATGATGGTATTTCTATTGATTTCAACGATTGGATTTGCATAT [324]

Huperzia_nad3_128097_128416 ....C......T........TC...A...CTC..C......G..------ [320]

Huperzia_nad3_235967_235728 -------------------------------------------------- [240]

Huperzia_nad3_63988_64073 -------------------------------------------------- [86]

Isoetes .CC....C..........C..C..C.................C.AT.... [324]

Vitis C......C.......CC...T.........TG..........C.CTC... [324]

Zea_perennis C......C.......CC...T.........TG..........C.CTC... [324]

Cycas CCC....C........C...TC.......C.G..........C.C.CC.. [324]

Megaceros C..................CTC.........G.............T.... [324]

Phaeoceros ...................CTC.........G.............T.... [324]

Physcomitrella .............A......T.........TG............TT...C [324]

Anomodon .............A......T...C.....TG............TT...C [324]

Marchantia ....................T....T....TG.............T.... [324]

Treubia ....................T....T....TG.............T.... [324]

[ 360 370 380 ]

[ . . . ]

Huperzia_nad3 GAATGGAAAAAGGGCGCTTTAGATTGGGAGTAA [357]

Huperzia_nad3_128097_128416 --------------------------------- [320]

Huperzia_nad3_235967_235728 --------------------------------- [240]

Huperzia_nad3_63988_64073 --------------------------------- [86]

Isoetes .......C...........CG.........C.. [357]

Vitis ..........G...T....CG...C........ [357]

Zea_perennis ..........G...T....CG...C........ [357]

Cycas ..............T....CG............ [357]

Megaceros ...................C............. [357]

Phaeoceros ................................. [357]

Physcomitrella ................................. [357]

Anomodon ........G........................ [357]

Marchantia ................................. [357]

Treubia ..............T.................. [357]

;

END;

**nad4**

#NEXUS

[MacClade 4.05 registered to Yin-Long Qiu Lab, EEB, University of Michigan]

BEGIN DATA;

DIMENSIONS NTAX=12 NCHAR=1507;

FORMAT DATATYPE=DNA MISSING=? GAP=- MATCHCHAR=. INTERLEAVE ;

MATRIX

[ 10 20 30 40 50]

[ . . . . .]

Huperzia_nad4 ATGTTACAATCTCTAGCTCCATTTCATTCCAATCTCAGTGGTCTTATTTT [50]

Huperzia_nad4_354133_353684 -...............................................C. [49]

Isoetes .C....G.................T....T......G......CC...CC [50]

Zea_perennis ......G..CA.T.CAG.GA..GCT...T.G....A.......C....C. [50]

Arabidopsis ......G..CA.T.CTG.GA..GCT....T.....A............C. [50]

Cycas ....C....CAAT.TCG.GA..GCT..........A.......C....CC [50]

Megaceros .C........T..C....T..CC....CT......A.............. [50]

Phaeoceros .C........T..C....TT..C....CT......A.............. [50]

Anomodon ..........T.T...........T...................C...C. [50]

Physcomitrella ..........T.T...........T.......................C. [50]

Treubia .........GT.T...........TC..T......A.............. [50]

Marchantia .........GT.T...........T........T.A.............. [50]

[ 60 70 80 90 100]

[ . . . . .]

Huperzia_nad4 GTGC---CCTTTGTTAGGAAGCATTATTATTTTTGCTCTCCCTGATTCAA [97]

Huperzia_nad4_354133_353684 .C..CGC....C..G.................CC................ [99]

Isoetes ...T---..CCC...G.......CC...C.C....T.A............ [97]

Zea_perennis C..T---..CG..C............C.CC.C..TTCA.T..AA....TT [97]

Arabidopsis ...T---...G..C............C.C..C..TTCA.T..AA...... [97]

Cycas ...T---..CG..C..............CC.C..TTC..T..AA....G. [97]

Megaceros .C.T---........G............C..C.C.T.A............ [97]

Phaeoceros .C.T---.T....C.G............C..C.CCT.A.T.......... [97]

Anomodon ...T---.....A...........C...C....C.T.A....C...C.C. [97]

Physcomitrella ...T---.....................C......T.A........C.C. [97]

Treubia .CTT---...............C..........G.TGA....GA...... [97]

Marchantia .CTT---......C........C..........G.TGA....GA...... [97]

[ 110 120 130 140 150]

[ . . . . .]

Huperzia_nad4 GAATACGACTGATACAAAGTATTGGTCTGTGCACTTCTTTGATTACTTTT [147]

Huperzia_nad4_354133_353684 ............G.....................C.....T......... [149]

Isoetes .....T........TG..................C...........C.C. [147]

Zea_perennis C....A...C.....G.TTG............GT....C.T......... [147]

Arabidopsis .........C.....G.TTA...........TG.C...C.T......... [147]

Cycas ....G..........G.TC.............G.C...C.TC........ [147]

Megaceros ....C.A........G.............C....C.T......C.....C [147]

Phaeoceros ......A.......TG...........C.C....C.T......C...... [147]

Anomodon ...............G..........T.......C..............C [147]

Physcomitrella ...............G..........T.......C............... [147]

Treubia ..G....T.......G.GA...CACA..T..G..C........G...... [147]

Marchantia ..G....T.......G.G....CACAA.T..G..C............... [147]

[ 160 170 180 190 200]

[ . . . . .]

Huperzia_nad4 TTGTATCCTCTTATTTTTTGGGTAAGGTTTGATAA---TTCTACTGCCAA [194]

Huperzia_nad4_354133_353684 ..T...G.C-----....C.....G.....A....TAA..T...C..... [194]

Isoetes .C........C.T....CC................---......A..... [194]

Zea_perennis ........C.C.G..CC.C..A..CAA..C...CC---......G..... [194]

Arabidopsis ......T...C.G..CC.C..A..CAA..C..CTC---......G..... [194]

Cycas .C...C..C.C.G..CC.C..A..CAA..C...TC---......G..... [194]

Megaceros ..A...T...CCT..C.....A..GAA..C.....---......A..... [194]

Phaeoceros ..A...T...C.T........A..GAA..C.....---......A..... [194]

Anomodon ..A...T.C...C....C...A..CAA........---......A..... [194]

Physcomitrella ..A...T.C...C........A..CAA........---......A..... [194]

Treubia C.A...T.....T........A..C.C..C..G..---.GA...A..A.. [194]

Marchantia ..A...T.....T........A..C.C.....G..---.GA...A..A.. [194]

[ 210 220 230 240 250]

[ . . . . .]

Huperzia_nad4 ATTTCAATTTGTGCAAACCATTCGATGGCTTCCTGATTCAAACATCAATT [244]

Huperzia_nad4_354133_353684 .................................................C [244]

Isoetes .CC.T..CCC...G.......................C............ [244]

Zea_perennis T.C..........G...G.C..............T..GA.......C... [244]

Arabidopsis ..C..........G...G.C..............T..GA........... [244]

Cycas ..CC.........G....................T..GG..G........ [244]

Megaceros ...CT........G...G................T............... [244]

Phaeoceros ....T........G...G....T......C....T............... [244]

Anomodon .........C...G....................T.C............. [244]

Physcomitrella .........C...G....................T.C............. [244]

Treubia ......G......G....T..............GT.......T....... [244]

Marchantia ......G......G....T..............GT.......T....... [244]

[ 260 270 280 290 300]

[ . . . . .]

Huperzia_nad4 TTTCTATAGGTATAGATGGTATCTCTCCATTCTTTGTGGTCTTGACCA-- [292]

Huperzia_nad4_354133_353684 .......................CT...C.C.................TG [294]

Isoetes .C.....................C..T.G.............C.....-- [292]

Zea_perennis .G.A...G........C...C.T..ATT......C...A.A.......-- [292]

Arabidopsis ...A.T.G........C.........TT......C...A.A.......-- [292]

Cycas .G.A...G...............C..T...C..CC...A.A.......-- [292]

Megaceros .C.A......................TT..CT................-- [292]

Phaeoceros .A.A....................T.TT..CT.C..C...........-- [292]

Anomodon ...A............C.........TT...T..C.............-- [292]

Physcomitrella ...A............C.........T....T..C.............-- [292]

Treubia ...A...............G......TT..........A.T.......-- [292]

Marchantia ...A......................TT..........A.........-- [292]

[ 310 320 330 340 350]

[ . . . . .]

Huperzia_nad4 -CGTTTTTAATTCCTATTTGCATTTCAGTAGGTTGGTACAGTA---TCAA [338]

Huperzia_nad4_354133_353684 AT....C.C....T...........G...........C...C.GTC.... [344]

Isoetes -.....CC...C......C...C.C............C.....---..G. [338]

Zea_perennis -.A...C.G..C.................G.......CTG...---.G.G [338]

Arabidopsis -.A...C.G..C.................G.......CTG...---.G.G [338]

Cycas -.A....C...C......C......T...........C.....---.G.. [338]

Megaceros -.....C...........C....C.T...........C.....---C... [338]

Phaeoceros -.................C.......G..........C.....---.... [338]

Anomodon -.A.................T..C.T...........C.....---.T.. [338]

Physcomitrella -.A......................T...........C.....---.T.. [338]

Treubia -.........C.............CTT..T....TT..T..CG---.... [338]

Marchantia -.........C.............CTT..T..C.TT..T..CG---.... [338]

[ 360 370 380 390 400]

[ . . . . .]

Huperzia_nad4 GAATTATAAAAAGGAATATACGATAGCATTTCCAATTCGTGAATCTATCA [388]

Huperzia_nad4_354133_353684 ..G....G..G.A......G.A.........TA............C.... [394]

Isoetes A.G....G.G..A..G...............T.................. [388]

Zea_perennis A.G..T.GGG..A..G....TT.C........T...........T.C.A. [388]

Arabidopsis A.G....GGG..A..G....TT.C.......TT...........T.C.A. [388]

Cycas A.G....GGG.....G....TT......................T.CCA. [388]

Megaceros ..G......G..A..G....T......G...TT....T........C.T. [388]

Phaeoceros ..G......G..A..G....T......G...TT.............C.T. [388]

Anomodon A.G.........A..G....T..........TT....T........T... [388]

Physcomitrella A.G.........A..G....T...........T....TTA......T... [388]

Treubia A.G.....G......G....T......G...TTC...T........T..C [388]

Marchantia A.G.........A.......T......G...TTC...T........T..C [388]

[ 410 420 430 440 450]

[ . . . . .]

Huperzia_nad4 TGATTGCTGTTTTTTG-CATGCTGGATCTTTTAC---TTTCTTATGTTTT [434]

Huperzia_nad4_354133_353684 ................G.....A....-...C..CTT..CT.C.G..... [443]

Isoetes ..........GCC.C.-.....C.....CC.C..---CAC..C....... [434]

Zea_perennis ....C..C..G.CC..-...........C.C...---.A.TC......C. [434]

Arabidopsis ....C..C..G..CC.-......A......C...---.A.TC........ [434]

Cycas ....C..C..G...C.-.....C.....C.....---.A.T.C.....C. [434]

Megaceros ..........G.C.C.-.....C.....C.....---.A...C....C.. [434]

Phaeoceros ..........G.C.C.-....TC....T......---.A.T.C....C.. [434]

Anomodon ..........G.....-.....C.......C..T---.A.T......... [434]

Physcomitrella ..........G.....-............C....---.A.T......... [434]

Treubia .C........G.....-.TCA..C.........A---.A.T...C..... [434]

Marchantia .A........G.....-.TCA..C.........A---.C.T...C..... [434]

[ 460 470 480 490 500]

[ . . . . .]

Huperzia_nad4 TCCTGAAAGCGTGTTAATCCCTATGTTC---ATTATTATAGGGATATGGG [481]

Huperzia_nad4_354133_353684 .......------------------------------------------- [450]

Isoetes .T.................T......CT---...........A...C... [481]

Zea_perennis .T.C.........CC.............---..C.........G...... [481]

Arabidopsis ...C.........CC.............---...........AG...... [481]

Cycas ...C.........C.............T---............G...... [481]

Megaceros ..T...........C...T.TC.....TCTT..C................ [484]

Phaeoceros .TT...........C...TT.C.....TCTT..C................ [484]

Anomodon ..T.........T..............T---.....C......G...... [481]

Physcomitrella .TT.........T..............T---.....C......G...... [481]

Treubia .TTC..............T..C.....T---...........TG.T.... [481]

Marchantia .TT...............T..C.....T---...........TG.T.... [481]

[ 510 520 530 540 550]

[ . . . . .]

Huperzia_nad4 GTTCTAGACAGAGAAAGATACAAGCAGCATATCAGTTTTTTTTATATACT [531]

Huperzia_nad4_354133_353684 -------------------------------------------------- [450]

Isoetes ........T..........CA....G..............C.C......A [531]

Zea_perennis ....G.....A........CA.G.................CC........ [531]

Arabidopsis ....G.....A........CA.G.................CC.T...... [531]

Cycas ....G.....A........CA.G......C........C.CCC......C [531]

Megaceros .C........A.....A........G..............C........C [534]

Phaeoceros ..CT....T.A.....A........G..........C...C........C [534]

Anomodon ..........A.....A..C....................C......... [531]

Physcomitrella ..........A.....A.......................C......... [531]

Treubia ....C.....A..G..A...A................C..C..G..C..C [531]

Marchantia ....C.....A..G..A..CA................C..C..G..C..C [531]

[ 560 570 580 590 600]

[ . . . . .]

Huperzia_nad4 TTACTTGGATCCGTCTTCATGCTCTTAGCTATTTTACTTATTTTTTTCCA [581]

Huperzia_nad4_354133_353684 -------------------------------------------------- [450]

Isoetes .C...C........G..........C........CG.C.....CC.CTGG [581]

Zea_perennis ..............T..T.....A.........C.GT.G...C..C.... [581]

Arabidopsis ...........TC.T..T.....A.........C.GT.G...C....... [581]

Cycas .C...C.....T..TC.T.....AC........CCGT.......C.C... [581]

Megaceros ...........T..G..T...........C..C.C.TC.....C...... [584]

Phaeoceros ...TC.....TT.....T.....T.....C....C.TC.....C....T. [584]

Anomodon .....C.....T............C....C......T.C........... [581]

Physcomitrella ...........T.................C......T............. [581]

Treubia ...A.G......T.G..............C......T..........T.. [581]

Marchantia ..GA.G......T.G..............C......T..........T.. [581]

[ 610 620 630 640 650]

[ . . . . .]

Huperzia_nad4 AACAGGAACCACCGATGTACAAATATTGTTAACCACAGAGTTTAGTGAGC [631]

Huperzia_nad4_354133_353684 -------------------------------------------------- [450]

Isoetes ...G.....A.................A........G.GA.......... [631]

Zea_perennis ................T.......T..A...........A.......... [631]

Arabidopsis ................T.........CA...........A.......... [631]

Cycas ................TC......C..A...........A..C....... [631]

Megaceros .........T..T...T..TG......A...........A.......... [634]

Phaeoceros .........T..T...T..TG......A...........A.C.......T [634]

Anomodon ................T..........A...........A.......... [631]

Physcomitrella ............T...T..........A...........A.......... [631]

Treubia ............T...C..........A...........A.......... [631]

Marchantia ............T...C..........A...........A.......... [631]

[ 660 670 680 690 700]

[ . . . . .]

Huperzia_nad4 GGCGCCAAATCCTGCTATGGATTGCTTTTTTTGCTTCTTTTTCCGTAAAA [681]

Huperzia_nad4_354133_353684 -------------------------------------------------- [450]

Isoetes ...........TC..CGC....C..C.C.CCC......CCCC....T... [681]

Zea_perennis .............T...........C.....C..C......G....C... [681]

Arabidopsis ...........T.T.............C...C..C.....CG....C... [681]

Cycas ...........T.T.C...........C..CC...C....CC........ [681]

Megaceros ........G.TT.....C.............C.........G.T..G... [684]

Phaeoceros ........G.TT.....C.............C.........G.T..G... [684]

Anomodon .A........AT...............................T..C... [681]

Physcomitrella ..........AT......................C............... [681]

Treubia ...........T...................C.....C..C..T..G... [681]

Marchantia ...........T............................C.....G... [681]

[ 710 720 730 740 750]

[ . . . . .]

Huperzia_nad4 GTGCCTATGGTACCAGTTCATATTTGGTTACCCGAAGCTCATGTAGAGGC [731]

Huperzia_nad4_354133_353684 -------------------------------------------------- [450]

Isoetes ........................C...C.T........T.......... [731]

Zea_perennis ......................................C........... [731]

Arabidopsis ................................T................. [731]

Cycas .......................C....C..................... [731]

Megaceros ..............T................................... [734]

Phaeoceros ..............T.........C..............T.......... [734]

Anomodon ................................T........C........ [731]

Physcomitrella .........A......................T........C........ [731]

Treubia ................................T...........G..... [731]

Marchantia ................................T...........G..... [731]

[ 760 770 780 790 800]

[ . . . . .]

Huperzia_nad4 ACCTACGGCTGGATCTGTAATCTCGGCAGGAATTCTTTCAAAATTGGGAA [781]

Huperzia_nad4_354133_353684 -------------------------------------------------- [450]

Isoetes ..............TC...................C...G.......... [781]

Zea_perennis ...............C..C....T..............T........... [781]

Arabidopsis .........A.....C..C....T...........C..T......T.... [781]

Cycas ...............C..C................C...G.......... [781]

Megaceros .....................T.T..........T.A............. [784]

Phaeoceros ..................................T.A............. [784]

Anomodon .......................T..............TG.....A.... [781]

Physcomitrella .......................T..............T......A.... [781]

Treubia G......................T..............T........... [781]

Marchantia .......................T..............T........... [781]

[ 810 820 830 840 850]

[ . . . . .]

Huperzia_nad4 CCCATGGTTTTTTAAGATTCAGCATACCCATGTGTCCTGTAGCGACACTC [831]

Huperzia_nad4_354133_353684 -------------------------------------------------- [450]

Isoetes ............C........A........C.C....C..........CT [831]

Zea_perennis ..T.C..G...........TTCA..........T...C.A.........T [831]

Arabidopsis ....C..G...........TTCA..........T...C.A.........T [831]

Cycas ..T....G.....G.....TTC...........T...C.A......G..T [831]

Megaceros ..T................TTC...........T.....A......CT.T [834]

Phaeoceros ..T......C.........TTC.....T.....T.....A..T...CT.T [834]

Anomodon ..T.................TCT..........T.....A.......... [831]

Physcomitrella ..T................TTC...........T.....A...A.....T [831]

Treubia ..T..........G.....TTC...........T.....A.........T [831]

Marchantia ..T................TTC...C.......T.....A.........T [831]

[ 860 870 880 890 900]

[ . . . . .]

Huperzia_nad4 TATTTCACTCCTTTCATTTATACTCTAAGCGTAATTGCTATTATATATAC [881]

Huperzia_nad4_354133_353684 -------------------------------------------------- [450]

Isoetes C...C..................C.CG.....G............C.... [881]

Zea_perennis .G...........................T.CG........A........ [881]

Arabidopsis .G..CT..................T......CG........A........ [881]

Cycas .G.C.........C.................CG..C....C......... [881]

Megaceros .T.....T...C.......G............G................. [884]

Phaeoceros .T.....T.T.........G............G................. [884]

Anomodon .....T.................CT.......C................. [881]

Physcomitrella Y....T..C.......................T................. [881]

Treubia .................C.....C........G................. [881]

Marchantia ................................G................. [881]

[ 910 920 930 940 950]

[ . . . . .]

Huperzia_nad4 TTCCTCGACTACAATAAGACAAATTGATCTGAAGAAAAATATTGCCTACT [931]

Huperzia_nad4_354133_353684 -------------------------------------------------- [450]

Isoetes CC.TC.......G...........C......................... [931]

Zea_perennis .....T...C..TT.......G..C.....T.....G.TC.......... [931]

Arabidopsis .....T...C..TTC......G..C.....A.....G.TC.....T.... [931]

Cycas ....C....C..TCCG........C....CT.....G.TC.......... [931]

Megaceros .C...T.......G.G........C........A....TG.......... [934]

Phaeoceros .C...T.......G.G...T....C.............TG.......... [934]

Anomodon .....T..................C.............T........... [931]

Physcomitrella .....T..................C.............T........... [931]

Treubia .....TA.................C.....A.......T......T.... [931]

Marchantia .....TA.................C.............T......T.... [931]

[ 960 970 980 990 1000]

[ . . . . .]

Huperzia_nad4 CTTCAGTAGCCCATATGAATTTTGGGACTATTGGTATGTTCAGTCTAAAC [981]

Huperzia_nad4_354133_353684 -------------------------------------------------- [450]

Isoetes ....G.................C........................... [981]

Zea_perennis .C....................G.T...............T....CG... [981]

Arabidopsis .C..................C.G.T...............T....CG... [981]

Cycas .CC............C......C.T..............C.....CG... [981]

Megaceros ..........T.............T......................... [984]

Phaeoceros TC......................T......................... [984]

Anomodon ..........T.............T......C.................. [981]

Physcomitrella ..........T.............T......................... [981]

Treubia ........................T......................... [981]

Marchantia ........................T...............T.....G... [981]

[ 1010 1020 1030 1040 1050]

[ . . . . .]

Huperzia_nad4 GTACAGGGAATTGAAGGTAGCATTTCACTCATGTTAAGTCATGGACCAGT [1031]

Huperzia_nad4_354133_353684 -------------------------------------------------- [450]

Isoetes ..G........C............CT...T...C.G..........TG.. [1031]

Zea_perennis A...C........G...........T...T................TG.. [1031]

Arabidopsis A............G..........CT..CG................TG.. [1031]

Cycas A............G..........C...CG....C............G.. [1031]

Megaceros A............................T...........C....TT.. [1034]

Phaeoceros A...........................CT...........C....TG.. [1034]

Anomodon A....A..............T....T...T...............ATG.. [1031]

Physcomitrella A....A..............T....T...T................TG.. [1031]

Treubia A........................T...T................TG.. [1031]

Marchantia A........................T...T................TG.. [1031]

[ 1060 1070 1080 1090 1100]

[ . . . . .]

Huperzia_nad4 TCCTTCAGCTCTTTTTTTATGTGTTGGTGCTTTATATGACCGACATAAGA [1081]

Huperzia_nad4_354133_353684 -------------------------------------------------- [450]

Isoetes .T.......C.....C.....C..C......................G.. [1081]

Zea_perennis .T.......C......C............T.C.................. [1081]

Arabidopsis .........C......C............T.................... [1081]

Cycas .........C.C....CC...........T.CC................. [1081]

Megaceros .T.C.T...C.C.CC.CC............CC........A......... [1084]

Phaeoceros ..TC.T...C.C.CC................C........A......... [1084]

Anomodon .T.......C...................T.................... [1081]

Physcomitrella .T.......C...................T.................... [1081]

Treubia .T.......C......C..........C...C.................. [1081]

Marchantia .T.......C.................C...C....C............. [1081]

[ 1110 1120 1130 1140 1150]

[ . . . . .]

Huperzia_nad4 CTCGACTTGTTAAATATTATGGAGGTTTAGTAAGCACCATGCCAATTTTT [1131]

Huperzia_nad4_354133_353684 -------------------------------------------------- [450]

Isoetes .C............C..C.......G.....C.......C.TT....CC. [1131]

Zea_perennis ............G..................G...........G.A...C [1131]

Arabidopsis ............G......C...........G...........G.A.C.C [1131]

Cycas ......C..C.................C.................A.CCC [1131]

Megaceros .........................G.....T.................C [1134]

Phaeoceros ..T.......G..............G.....T.........T.......C [1134]

Anomodon ...............................C...........C..G..C [1131]

Physcomitrella ...............................C..............G..C [1131]

Treubia .AA..A.........................C...........T...... [1131]

Marchantia .AA..A.........................C...........T.....C [1131]

[ 1160 1170 1180 1190 1200]

[ . . . . .]

Huperzia_nad4 TCTACCATTTCTTTATTTTTTACTTTAGCCAATATGAGTTTACCAGGTAC [1181]

Huperzia_nad4_354133_353684 -------------------------------------------------- [450]

Isoetes CTC......C.C.C..C...C...CC........C.........T..C.. [1181]

Zea_perennis ..........TC..C.....C.......................C..C.. [1181]

Arabidopsis ..........TC..T.C.........G.............C...T..... [1181]

Cycas ..........TC..C....CC...............G.......T..... [1181]

Megaceros .G.........C........C........G..............G....G [1184]

Phaeoceros .G.........C........C........G............TTG....G [1184]

Anomodon .....G..C.TC.....C.C...C....................T..... [1181]

Physcomitrella .....G....TC.....C.....C.......................... [1181]

Treubia ....T.....TC................................C..... [1181]

Marchantia ..........TC................................C..... [1181]

[ 1210 1220 1230 1240 1250]

[ . . . . .]

Huperzia_nad4 TAGCAGCTTTATTGGGGAATTCCTTATTTTGGTAGGAGCTTTCCAAAGAA [1231]

Huperzia_nad4_354133_353684 -------------------------------------------------- [450]

Isoetes .......CC...........C..C.G....A..........C.G...... [1231]

Zea_perennis ............C........T..A..C..A................... [1231]

Arabidopsis ............C........T..C..C..A................... [1231]

Cycas ........C...C........T.CC..C..A................... [1231]

Megaceros ............C........T.CC..CCCAAC..........G....G. [1234]

Phaeoceros ............C........T..C..CC.AA...........G....G. [1234]

Anomodon ............C..A.....T........A..............G.... [1231]

Physcomitrella ...............A.....T........A................... [1231]

Treubia ............C........T........A...............C... [1231]

Marchantia ............C........T........A................... [1231]

[ 1260 1270 1280 1290 1300]

[ . . . . .]

Huperzia_nad4 ATAGCTTAGTGGCCACATTAGCAGCACTTGGGATGATTTTAGGTGCAGCT [1281]

Huperzia_nad4_354133_353684 -------------------------------------------------- [450]

Isoetes ..............G............................C.....C [1281]

Zea_perennis ..........A..............G.................C..G..G [1281]

Arabidopsis ..........A..............G....................G..C [1281]

Cycas ..G.......A.......C......G.............C...C.....G [1281]

Megaceros .................C....G.....C.........C....C...... [1284]

Phaeoceros .................CC...G.....C.........C....C...... [1284]

Anomodon ......................G........A...........C...... [1281]

Physcomitrella .............T........G........A...........C...... [1281]

Treubia ..........A....................A...........C..C... [1281]

Marchantia ...........................................C..C... [1281]

[ 1310 1320 1330 1340 1350]

[ . . . . .]

Huperzia_nad4 TATTCTCTTTGGCTATATAATCGTGTGGTTTTTGGTAATTTCAAACCCAA [1331]

Huperzia_nad4_354133_353684 -------------------------------------------------- [450]

Isoetes C..CT..CCC...C................CC.................. [1331]

Zea_perennis .....C.........................C...A.....A......G. [1331]

Arabidopsis .....C.........................C...A.....A.....TG. [1331]

Cycas ...C.C.C.....C...........C....C....A....CA........ [1331]

Megaceros .......C.C..T..C...................G...........T.. [1334]

Phaeoceros ....T.......T........T.............G...........T.. [1334]

Anomodon .............C............AA.......G.............. [1331]

Physcomitrella ............T..............A.......G.............. [1331]

Treubia .....C.........................................A.. [1331]

Marchantia .................................................. [1331]

[ 1360 1370 1380 1390 1400]

[ . . . . .]

Huperzia_nad4 TTTCCTCCAGAAATTTTCCGATTCAGATAGAAGGGAAGTTCTAATATTTT [1381]

Huperzia_nad4_354133_353684 -------------------------------------------------- [450]

Isoetes C.CA.C.........C.........A.......A.....C.C.......C [1381]

Zea_perennis .......T.T.....C......CT.A..G.C..A......TCC......C [1381]

Arabidopsis .........T.....C.........A..G.C..A......TCC......A [1381]

Cycas .....C...T....CC......C..A..G....A.......CG....C.C [1381]

Megaceros ......AA.A...............A.......A......T.C..T.... [1384]

Phaeoceros ......AA.A.............T.A.......A......T.C..T.C.. [1384]

Anomodon A........A.....C.......T.A.......A................ [1381]

Physcomitrella A........A.....C.......T.A.......A................ [1381]

Treubia ...TA...TC.....C.......T.A.......A........C.CC.... [1381]

Marchantia ...TA...TC.....C.......T.A.......A........C..C.... [1381]

[ 1410 1420 1430 1440 1450]

[ . . . . .]

Huperzia_nad4 TACCTTTTATTGTTGGAGTTATTTGGATGGGTGTTTACCCCGAAGTGTTC [1431]

Huperzia_nad4_354133_353684 -------------------------------------------------- [450]

Isoetes ..T...CC..C..C.....................C...TT.......CT [1431]

Zea_perennis ........C...........G..C...........C.....A........ [1431]

Arabidopsis ........C........C.CG..C...........C.....A........ [1431]

Cycas C....C...C..........G..C...........C.....A.G...... [1431]

Megaceros ....C..................C................T......... [1434]

Phaeoceros ...TC..................................TT......... [1434]

Anomodon .C................................C...........T... [1431]

Physcomitrella .C...........C................................T... [1431]

Treubia .................A............................A... [1431]

Marchantia .............G..........................T......... [1431]

[ 1460 1470 1480 1490 1500]

[ . . . . .]

Huperzia_nad4 CCAGAGTGTATGCATACTTCCGTAAGTAACTTAGTGCAACATGGAAGATT [1481]

Huperzia_nad4_354133_353684 -------------------------------------------------- [450]

Isoetes ..G...C...C.T.....C...CGG......C..............A..C [1481]

Zea_perennis ..G..C..C........A..........................C.A... [1481]

Arabidopsis ..G..C..C........A..........................C.A... [1481]

Cycas ..G..CC..........CC............C............C.A... [1481]

Megaceros TT.................GT..G......................A... [1484]

Phaeoceros TT..........T......GT..G............T.........A... [1484]

Anomodon TT............................................A... [1481]

Physcomitrella .T............................................A... [1481]

Treubia .T............................................A... [1481]

Marchantia .T............................................A... [1481]

[ ]

[ ]

Huperzia_nad4 TGATTAA [1488]

Huperzia_nad4_354133_353684 ------- [450]

Isoetes C...C.. [1488]

Zea_perennis .C...G. [1488]

Arabidopsis .C...G. [1488]

Cycas CC..CG. [1488]

Megaceros ....... [1491]

Phaeoceros ....... [1491]

Anomodon .....G. [1488]

Physcomitrella ....... [1488]

Treubia ....... [1488]

Marchantia ....... [1488]

;

END;

**nad4L**

#NEXUS

[MacClade 4.05 registered to Yin-Long Qiu Lab, EEB, University of Michigan]

BEGIN DATA;

DIMENSIONS NTAX=13 NCHAR=317;

FORMAT DATATYPE=DNA MISSING=? GAP=- MATCHCHAR=. INTERLEAVE ;

MATRIX

[ 10 20 30 40 50]

[ . . . . .]

Huperzia_nad4L ATGGATCTAGTCAAATATTTAACATTTTCTATGATACTTTTTCTCTTAGG [50]

Huperzia_nad4L_160617_160531 -------------------------------------------------- [0]

Huperzia_nad4L_284686_284575 -------------------------------------------------- [0]

Isoetes .C.................C......CC............A...T..... [50]

Arabidopsis ........TA..........C..............TA...C.A.T..... [50]

Zea_perennis .C.....CTA.A........C..T...........CA.C.C.A.T..... [50]

Cycas ........T..................C.......CA...C.....C... [50]

Megaceros ..............C...........C..C.....T.C..C.T.T..G.. [50]

Phaeoceros ..............C...........C........T.C....T.T..G.. [50]

Anomodon ...........A.......................T........T.C... [50]

Physcomitrella ...........A.......................T........T..... [50]

Marchantia ...........A.......................T........T..... [50]

Treubia ...........A.......................T........TC.... [50]

[ 60 70 80 90 100]

[ . . . . .]

Huperzia_nad4L TATTTGGGGAATTTTTTTAAATAGAAAAAACATTCTTATTTTGTTAATGC [100]

Huperzia_nad4L_160617_160531 -------------------------------------------------- [0]

Huperzia_nad4L_284686_284575 -------------------------------------------------- [0]

Isoetes .C.......G.....C.C..................C............. [100]

Arabidopsis ....C.......CC.CC.T......CG...T....C....A...C..... [100]

Zea_perennis ....C.......CC.CC.T......CG...T.........A...C..... [100]

Cycas ....C.......C..CC.G......CG...T...A.....A...C....T [100]

Megaceros .............C..CCG....A..G...T....C....A..CC..C.. [100]

Phaeoceros ................CCG....A..G...T....C....A..CC..C.. [100]

Anomodon .........G.....C..G...........T..C......A........T [100]

Physcomitrella .........G.....C..G..............C......A........T [100]

Marchantia ...............C..............T.........A........T [100]

Treubia ...............C..............T.........A........T [100]

[ 110 120 130 140 150]

[ . . . . .]

Huperzia_nad4L CGATTGAGTTAATGTTACTGGCTGTCAATTTGAACTTTTCGGTCTTTTCT [150]

Huperzia_nad4L_160617_160531 -------------------------------------------------- [0]

Huperzia_nad4L_284686_284575 -------------------------------------------------- [0]

Isoetes .A.......C.........C..........C.C...C..T...A...CTC [150]

Arabidopsis .A.....A.C.......T.A.....G....C........T...A.....C [150]

Zea_perennis .A.....A.C.......T.A...................T...A.....C [150]

Cycas .A.....A.C.........A....C.....C........T...A...C.C [150]

Megaceros T........C.........T..A...........T.CC.T...A...CTG [150]

Phaeoceros T.................CT.TA...........T.CC.T...A...CTG [150]

Anomodon .A.................T..G...G....A......CT...A.....G [150]

Physcomitrella .A.................T..A...G....A.......T...A.....G [150]

Marchantia .A.................T...................T...A...... [150]

Treubia .A........G........T...................T...A...... [150]

[ 160 170 180 190 200]

[ . . . . .]

Huperzia_nad4L GTTTATTTGGATGATACGATGGGTCAATTATTTGCTCTATTTGTGTTAAC [200]

Huperzia_nad4L_160617_160531 -------------------------------------......CA..... [13]

Huperzia_nad4L_284686_284575 -----------------.....C...T.....G...T...C....C.... [33]

Isoetes ...C...............CA.......C........C..CC..A.CG.. [200]

Arabidopsis ....C..C........T..........G........TC...G..TCC... [200]

Zea_perennis ....C...........T...........C.......TC...A..TCC... [200]

Cycas ......CC........T...................TC..CG........ [200]

Megaceros ...C............T..............CC..........CT..... [200]

Phaeoceros ...C............T..............CC..........CT..... [200]

Anomodon ........A.......T...................T.......T..G.. [200]

Physcomitrella ................T...................T.......T..... [200]

Marchantia ................T...................T.......T..... [200]

Treubia ...C............T...................T.......T..... [200]

[ 210 220 230 240 250]

[ . . . . .]

Huperzia_nad4L GGTGGCAGCTGCAGAATCC---GCTATTGGGCT--------GGCCATT-- [237]

Huperzia_nad4L_160617_160531 ....A...TC...A...T.---.....G..------------......-- [46]

Huperzia_nad4L_284686_284575 ......T.....GA....TTATAT...G.....TTAATTAA.......AT [83]

Isoetes A...............C.T---..........C--------.......-- [237]

Arabidopsis ............G......---.........T.--------A......-- [237]

Zea_perennis A...........G.....T---........AT.--------A......-- [237]

Cycas ............G.....T---.........TC--------.......-- [237]

Megaceros .A....T....TG.....T---..........C--------.......-- [237]

Phaeoceros .A....T....TG.....T---..........C--------.......-- [237]

Anomodon ............G......---...........--------.......-- [237]

Physcomitrella .........A..G......---...........--------.......-- [237]

Marchantia ......T.....G......---.........T.--------.......-- [237]

Treubia ......T.....G......---.........T.--------.......-- [237]

[ 260 270 280 290 300]

[ . . . . .]

Huperzia_nad4L -CTGGTTATTACTTTTAGAATTCGCGGGACTATTGCAGTGGAATTTATTA [286]

Huperzia_nad4L_160617_160531 -T.........T...G......A......T.........A..-------- [87]

Huperzia_nad4L_284686_284575 T......C........C..C.........--------------------- [112]

Isoetes -AC...........C.C...............CC..G..........C.. [286]

Arabidopsis -T.C.....A.....CC..G.C..A..T........T..A.......... [286]

Zea_perennis -T.C...............G.C..A...........T..C........A. [286]

Cycas -.C............CC.......A.....C........A....C..... [286]

Megaceros -.....C.........T..........A........T........G.... [286]

Phaeoceros -.....C.........T..........A.................G.... [286]

Anomodon -........A......C.......T........C................ [286]

Physcomitrella -........A......C.......T......................... [286]

Marchantia -...............C..........T...................... [286]

Treubia -T..............C..........T...........A.......... [286]

[ 310 ]

[ . ]

Huperzia_nad4L ATGGCATGAAAGGTTAA [303]

Huperzia_nad4L_160617_160531 ----------------- [87]

Huperzia_nad4L_284686_284575 ----------------- [112]

Isoetes G.....C.......C.G [303]

Arabidopsis ..A....TC........ [303]

Zea_perennis ..T....TC........ [303]

Cycas ..T.......G...C.. [303]

Megaceros G.T.......G...C.. [303]

Phaeoceros ..T.......G...C.. [303]

Anomodon ..T.......G..A... [303]

Physcomitrella ..T.......G..A... [303]

Marchantia ..T.......G...... [303]

Treubia ..T.......G...... [303]

;

END;

**nad5**

#NEXUS

[MacClade 4.05 registered to Yin-Long Qiu Lab, EEB, University of Michigan]

BEGIN DATA;

DIMENSIONS NTAX=15 NCHAR=2089;

FORMAT DATATYPE=DNA MISSING=? GAP=- MATCHCHAR=. INTERLEAVE ;

MATRIX

[ 10 20 30 40 50]

[ . . . . .]

Huperzia_nad5 ACGTATTTACTCATAGTATTGTTGCCCCTGCTAGGTAGCTTCGTTGCAGG [50]

Huperzia_nad5_307277_307833 -------------------------------------------------- [0]

Huperzia_nad5_182779_182719 -------------------------------------------------- [0]

Huperzia_nad5_241849_241050 -------------------------------------------------- [0]

Huperzia_nad5_108187_107647 --..........CC....C........T...CT........T........ [48]

Isoetes .T.........T.......C..C....TC..CG.......C...A..... [50]

Zea_perennis .T....C....T..T..C..T.....TT....C.....T.C...A..C.. [50]

Arabidopsis .T....C....T..C.....T...........C.....T.C...A..... [50]

Cycas .T.C..C....A.......C.......T....C.....T.C...A..... [50]

Megaceros .T.........A..C..CACT..A..TT...C......T.....A..... [50]

Phaeoceros .T.........A..C...ACT..A.TTT...C......T..T..A..... [50]

Anomodon .T.......T.A..T...ACT..A..TT.A........T.GT..A..... [50]

Physcomitrella .T.......T.A..T...ACT..A..TT.A........T.GT..A..... [50]

Treubia .T.........T.....CA.T..A..GT..A.T..G..TG.T.CG..... [50]

Marchantia .T.........T.....CA.T..A..GT..A.T..G..T..T.CG..... [50]

[ 60 70 80 90 100]

[ . . . . .]

Huperzia_nad5 AGCTTTTGGTCGTTTTCTGGGTTC----------AAAAGGAACCGCTATA [90]

Huperzia_nad5_307277_307833 -------------------------------------------------- [0]

Huperzia_nad5_182779_182719 -------------------------------------------------- [0]

Huperzia_nad5_241849_241050 -------------------------------------------------- [0]

Huperzia_nad5_108187_107647 .................C.....GTTAAAAAAAA...G............ [98]

Isoetes ....CC...........C......----------..G............. [90]

Zea_perennis TTT...C..A........A..A..----------TG.............. [90]

Arabidopsis TTT...C..A........A..A..----------.G......G....... [90]

Cycas T.....C..A.......CA.....----------.G.............. [90]

Megaceros T..C.............CT.....----------.TG......T....C. [90]

Phaeoceros T..C..............T.....----------.TG......T....C. [90]

Anomodon T............C....T...CT----------.CG......T...... [90]

Physcomitrella T.................T....T----------.CG......T...... [90]

Treubia TTT...............T.....----------..GG...GTG...G.. [90]

Marchantia GTT...............T.....----------..GG...GTG...G.. [90]

[ 110 120 130 140 150]

[ . . . . .]

Huperzia_nad5 GTAACCACCACGTGCGTTTCATTATCTTCCATTTTATCTTTGATTGCTTT [140]

Huperzia_nad5_307277_307833 -------------------------------------------------- [0]

Huperzia_nad5_182779_182719 -------------------------------------------------- [0]

Huperzia_nad5_241849_241050 -------------------------------------------------- [0]

Huperzia_nad5_108187_107647 ............................AT.................... [148]

Isoetes ............C.....C..........T.....CC...C......... [140]

Zea_perennis A.G....................T.....G..C................. [140]

Arabidopsis A.G.....T..............C.....G..C................. [140]

Cycas .............................T..C.C..............C [140]

Megaceros A.C..A....T...T..C..........GT.....CC..........C.. [140]

Phaeoceros A.C..A....T...T..C.......G...T.....CCT.........C.. [140]

Anomodon ..C..A........T.............T......C.............. [140]

Physcomitrella ..C..A........T.............TT.........C.......... [140]

Treubia ..C..A........T..G...........T..G.......GT.....A.. [140]

Marchantia ..C..A........T..............T.....C....GT.....A.. [140]

[ 160 170 180 190 200]

[ . . . . .]

Huperzia_nad5 CTATGAAGTTGCACTGGGAGCCAGTGCTTGCTATATCAAGATTGCTCCCT [190]

Huperzia_nad5_307277_307833 -------------------------------------------------- [0]

Huperzia_nad5_182779_182719 -------------------------------------------------- [0]

Huperzia_nad5_241849_241050 -------------------------------------------------- [0]

Huperzia_nad5_108187_107647 T.C...C............................C............T. [198]

Isoetes T.C...................G...T.C.T...........C.T...AC [190]

Zea_perennis T........C...........T............C...GA..A.....A. [190]

Arabidopsis T........C....C......T............C.A.GA........A. [190]

Cycas T........C....C......T............CCA...........A. [190]

Megaceros T....................T....T.........A.........T.A. [190]

Phaeoceros T....................T....T.........A.........T.A. [190]

Anomodon T...................................G..A........A. [190]

Physcomitrella T...................................A..A........A. [190]

Treubia T........C..G...T.T.................A..A........T. [190]

Marchantia T........C..G...T.T.................A...........T. [190]

[ 210 220 230 240 250]

[ . . . . .]

Huperzia_nad5 GGATTTTATCGGAGATGTTTGATGCTTCTTGGGGCTTCTTGTTTGATAGT [240]

Huperzia_nad5_307277_307833 -------------------------------------------------- [0]

Huperzia_nad5_182779_182719 -------------------------------------------------- [0]

Huperzia_nad5_241849_241050 -------------------------------------------------- [0]

Huperzia_nad5_108187_107647 .....G...TTTC.........C.T....C.......T...C........ [248]

Isoetes ....C.CCC....A..........T.C..C......CA.C..C....... [240]

Zea_perennis ....C.C......A..........................T..C.....C [240]

Arabidopsis ....C.C......A.............................C.....C [240]

Cycas .......CC.T..A...............C......C...T........C [240]

Megaceros .....A.C...................T.C......C.....C....... [240]

Phaeoceros .....A.C...................T.C......C............. [240]

Anomodon .......T..C.............................T......... [240]

Physcomitrella .......T..C.............................T......... [240]

Treubia .......T.....AC.C.....C...G..........T..A.....C..C [240]

Marchantia .......T.....AC.C.....C...G..........T..A.....C..C [240]

[ 260 270 280 290 300]

[ . . . . .]

Huperzia_nad5 CTGACTGTAGTTATGTTAATTGTGGTTACATTTGCAAGTAGCTTAGTTCA [290]

Huperzia_nad5_307277_307833 -------------------------------------------------- [0]

Huperzia_nad5_182779_182719 -------------------------------------------------- [0]

Huperzia_nad5_241849_241050 -------------------------------------------------- [0]

Huperzia_nad5_108187_107647 .CA.........GC..C.....C........................... [298]

Isoetes .............C..C.........C...C.C.TC...........C.. [290]

Zea_perennis .....C.....G....................CAT.........G..C.. [290]

Arabidopsis .C...C.....G...................CCAT.........G..C.. [290]

Cycas .C...C.........CC.....C.........C.TG.......CG..C.. [290]

Megaceros ...............C..................T.........G..... [290]

Phaeoceros ..................................T.........G..... [290]

Anomodon .C................................T............... [290]

Physcomitrella ..................................T............... [290]

Treubia ..C..A..CA..T.A...T.G..C..C...A...T............... [290]

Marchantia ..T..A..CA..T.A...T.G..C..C...A...T............... [290]

[ 310 320 330 340 350]

[ . . . . .]

Huperzia_nad5 TCTTTATTCTATTTCATATATGTCCGAGGATCCACATAGCCCTCGATTTA [340]

Huperzia_nad5_307277_307833 -------------------------------------------------- [0]

Huperzia_nad5_182779_182719 -------------------------------------------------- [0]

Huperzia_nad5_241849_241050 -------------------------------------------------- [0]

Huperzia_nad5_108187_107647 ..CC...CT.......G.GG.....................T........ [348]

Isoetes C......CTC......C...C............................. [340]

Zea_perennis .........C..............T........G................ [340]

Arabidopsis .........C.......................G................ [340]

Cycas ...C...C.C........................................ [340]

Megaceros .......C.C......C................G......T.AT...... [340]

Phaeoceros ........T.......C................G......T.CT...... [340]

Anomodon ...C.....C..............T...........C............. [340]

Physcomitrella .........C..........................C............. [340]

Treubia CA.......A.....C........T........G.....T..A..T..CT [340]

Marchantia .A.......A.....C........T........G.....T..A..T...T [340]

[ 360 370 380 390 400]

[ . . . . .]

Huperzia_nad5 TGTGTTATTTATCCATTCCTACTTTTTTTATGCTAATGCTGGTTACCGGA [390]

Huperzia_nad5_307277_307833 -------------------------------------------------- [0]

Huperzia_nad5_182779_182719 -------------------------------------------------- [0]

Huperzia_nad5_241849_241050 -------------------------------------------------- [0]

Huperzia_nad5_108187_107647 .............................C.....T.............G [398]

Isoetes ...........C.......C.T.C......C..C....T..........G [390]

Zea_perennis .................TT...................T....G..T... [390]

Arabidopsis ..................T..............C....T....G..T... [390]

Cycas .......C.C.................C..C..C....TC.......... [390]

Megaceros .............T...................C.....C.......... [390]

Phaeoceros ............TT.........CC......................... [390]

Anomodon ....C............TT..............C....T....C...... [390]

Physcomitrella ....C............TT...................T........... [390]

Treubia .T..C.....G..A...TT...............T...T....A..T... [390]

Marchantia .T..C.....G..A...TT...............T...T....A..T... [390]

[ 410 420 430 440 450]

[ . . . . .]

Huperzia_nad5 GATAACTTTATTCAATTATTCCTA-GGATGGGAGGG-CGTGGGTCTCGCT [438]

Huperzia_nad5_307277_307833 -------------------------------------------------- [0]

Huperzia_nad5_182779_182719 -------------------------------------------------- [0]

Huperzia_nad5_241849_241050 --...............T..ATGGC..........TTACCT..GG.AA.. [48]

Huperzia_nad5_108187_107647 .----..CC.......CCA.T.A.-...A..TG..TTACCA..GG.AA.. [443]

Isoetes .......C.C..T.....C.T.C.-...........-A........T... [438]

Zea_perennis .........C.............G-...........-A..A.....T... [438]

Arabidopsis .......C.C.............G-...........-A..A.....T... [438]

Cycas ......................CG-...........-G..A......... [438]

Megaceros .......C....T.......T...-...........-A..C....CT... [438]

Phaeoceros .......C....T.......T...-...........-A..C.....T... [438]

Anomodon ...........C........TT..-........A..-A..A......... [438]

Physcomitrella ....................T...-........A..-A..A......... [438]

Treubia .....T........G.....T..G-...........-A..T..A.....G [438]

Marchantia .....T........G.....TT..-...........-A..T..A.....G [438]

[ 460 470 480 490 500]

[ . . . . .]

Huperzia_nad5 TCATATTTGTCAATTAATTTT----TGGTTTACACGAC---TTCCGGCAA [481]

Huperzia_nad5_307277_307833 -------------------------------------------------- [0]

Huperzia_nad5_182779_182719 -------------------------------------------------- [0]

Huperzia_nad5_241849_241050 C....................----.............---......... [91]

Huperzia_nad5_108187_107647 C.......A.TTGCC.....AGGGA...........GTGGT......... [493]

Isoetes CTG....C......C...C.C----C........T...---..TA..... [481]

Zea_perennis ..........T....C....C----.............---...A...GG [481]

Arabidopsis ..........T....C.....----.............---...A....G [481]

Cycas C.........T.........C----C...CC.....G.---.C.A....G [481]

Megaceros .T...................----.....C..GA...---...A...C. [481]

Phaeoceros .T...................----.....C..GT...---..TA..... [481]

Anomodon ..........T.........C----.............---...A...C. [481]

Physcomitrella ..........T.........C----.............---...A...C. [481]

Treubia ........A.TG..C......----.....C..G..CA---...A..... [481]

Marchantia ........A.TG..A......----........G..CA---...A..... [481]

[ 510 520 530 540 550]

[ . . . . .]

Huperzia_nad5 ATAAAGCAGCTATCAAAGCTATGCTTGTCAA---TCGAGTAGGTGATTTT [528]

Huperzia_nad5_307277_307833 -------------------------------------------------- [0]

Huperzia_nad5_182779_182719 -------------------------------------------------- [0]

Huperzia_nad5_241849_241050 ...............................---..AG......A..... [138]

Huperzia_nad5_108187_107647 ..............G................GAA.T............-- [541]

Isoetes .C.......................C.....---.T.............C [528]

Zea_perennis .............A.................---................ [528]

Arabidopsis ............CA.................---................ [528]

Cycas .............A.................---................ [528]

Megaceros ........................C...G..---.T..........C... [528]

Phaeoceros ....G.............T.........G..---............C... [528]

Anomodon .............A.................---...............C [528]

Physcomitrella .......G.....A.................---...............C [528]

Treubia .......G.....T...........CA.A..---...C............ [528]

Marchantia .......G.....T...........CA.A..---...C............ [528]

[ 560 570 580 590 600]

[ . . . . .]

Huperzia_nad5 GGATT-----AGCTCTCGGGATTATGGGTCGTTTTACTATTTCTCAAAC- [572]

Huperzia_nad5_307277_307833 -------------------------------------------------- [0]

Huperzia_nad5_182779_182719 -------------------------------------------------- [0]

Huperzia_nad5_241849_241050 TTT..TTCTT............CGCC.-.T.C........C.TG.C...A [187]

Huperzia_nad5_108187_107647 -------------------------------------------------- [541]

Isoetes ....C-----............G......T........C.....G....- [572]

Zea_perennis .....-----......T......T.T...T........C.C.T......- [572]

Arabidopsis .....-----......T......TC.............C.C.T......- [572]

Cycas ....C-----.....C.......T.........C....C.C.T......- [572]

Megaceros .....-----.....C.............G.......CC...T......- [572]

Phaeoceros .....-----...................G.......CC...T.T...T- [572]

Anomodon .....-----...................T............T......- [572]

Physcomitrella .....-----...................T............T......- [572]

Treubia .....-----...................T....C.....C.T......- [572]

Marchantia .....-----.........A.........T............T......- [572]

[ 610 620 630 640 650]

[ . . . . .]

Huperzia_nad5 ------AGTAGACTTTTCTACTATTTTTGCTTGTGCCAGTGTCTTTTCCG [616]

Huperzia_nad5_307277_307833 -------------------------------------------------- [0]

Huperzia_nad5_182779_182719 -------------------------------------------------- [0]

Huperzia_nad5_241849_241050 ACAGTA.........C..........A....C....T....C........ [237]

Huperzia_nad5_108187_107647 -------------------------------------------------- [541]

Isoetes ------........C.C.A...C........C.....TC----------- [605]

Zea_perennis ------............A..C..............T....CT------- [609]

Arabidopsis ------............A..C.........C....T....C.------- [609]

Cycas ------............G..C....C....C....T....C........ [616]

Megaceros ------.........................C....T....CT..C..T. [616]

Phaeoceros ------..............................T....C...C..T. [616]

Anomodon ------............G............C....T..C.CT....... [616]

Physcomitrella ------............G............C.......C.C......T. [616]

Treubia ------...T........C.................T....C......T. [616]

Marchantia ------...T........C.................T....C........ [616]

[ 660 670 680 690 700]

[ . . . . .]

Huperzia_nad5 AACCCAACCATTATTTCATTTTTTGTGAGATGAAATTCCATGCCATAACT [666]

Huperzia_nad5_307277_307833 -------------------------------------------------- [0]

Huperzia_nad5_182779_182719 -------------------------------------------------- [0]

Huperzia_nad5_241849_241050 .......T....................CT...G....T........... [287]

Huperzia_nad5_108187_107647 -------------------------------------------------- [541]

Isoetes -......T.......C............T....G..CT.......C..T. [654]

Zea_perennis --....GAA..G.A.GG........CA.T....G...TA........... [657]

Arabidopsis --....GAA...C..GG....C...CA.T....G...GA.........G. [657]

Cycas .......GA...C.......CC.C.CA.T....G..CT............ [666]

Megaceros ............TC..TG........A.T....G...T.....T...... [666]

Phaeoceros ............T...TC........A.T....G...T.....T...... [666]

Anomodon .....C.T........T....C...CA.T....G...T............ [666]

Physcomitrella .....C.T........T........CA.T....G...T............ [666]

Treubia .....C.T.........C........A.T...GG...T..C........C [666]

Marchantia .....C.T.........C........A.C...GG...T............ [666]

[ 710 720 730 740 750]

[ . . . . .]

Huperzia_nad5 GTTATTTGTATTTTACTTCCTACTGGCGCTGTTGGAAAATCCGCACAAAT [716]

Huperzia_nad5_307277_307833 -------------------------------------------------- [0]

Huperzia_nad5_182779_182719 -------------------------------------------------- [0]

Huperzia_nad5_241849_241050 ...............GC........................A.T...G.. [337]

Huperzia_nad5_108187_107647 -------------------------------------------------- [541]

Isoetes ......C..........AT.CC...................T........ [704]

Zea_perennis C.G...............TT..T...T..A.....G.....T.....G.. [707]

Arabidopsis C..................T..T...T........G........G..G.. [707]

Cycas ......C.....CC..CCT...T...T..............T........ [716]

Megaceros ................CG.TC.T.........C........T...T.... [716]

Phaeoceros .................GTTC.T.................TT...T.... [716]

Anomodon ...........C......TTC.TC...........G.....T........ [716]

Physcomitrella ..................TTC.T..................T........ [716]

Treubia ...............G.GTT..TC.................T.....G.. [716]

Marchantia ...............G.GTT..T..................T.....G.. [716]

[ 760 770 780 790 800]

[ . . . . .]

Huperzia_nad5 AGGATTACATACTTGGTTACCCGATGCAATGGAGGGTCCCACTCCAGTAT [766]

Huperzia_nad5_307277_307833 -------------------------------------------------- [0]

Huperzia_nad5_182779_182719 -------------------------------------------------- [0]

Huperzia_nad5_241849_241050 ......G......C..............--.................... [385]

Huperzia_nad5_108187_107647 -------------------------------------------------- [541]

Isoetes G............C...C..............................C. [754]

Zea_perennis ......G........................................... [757]

Arabidopsis .....CG..........C.........T...................... [757]

Cycas .....CG......C...C................................ [766]

Megaceros ......G.................C.TC...................... [766]

Phaeoceros ......G.................C.TC...................... [766]

Anomodon ......G......C.......T.....G.......AG............. [766]

Physcomitrella ......G......C.......T.............AG............. [766]

Treubia T.....G.........C......................T.......... [766]

Marchantia T.....G........................................... [766]

[ 810 820 830 840 850]

[ . . . . .]

Huperzia_nad5 CCGC------TTTGATTCATGCCGCTACTACGGTAACAGCAGGCGTTTTT [810]

Huperzia_nad5_307277_307833 -------------------------------------------------- [0]

Huperzia_nad5_182779_182719 -------------------------------------------------- [0]

Huperzia_nad5_241849_241050 ....CGCCGC..C..................................... [435]

Huperzia_nad5_108187_107647 -------------------------------------------------- [541]

Isoetes ...T------.C..........G..........C.............CC. [798]

Zea_perennis .T..------............A.......T...C..T..T........C [801]

Arabidopsis ....------..C.........A.......T.........T........C [801]

Cycas ....------..C.........A.........................CC [810]

Megaceros ....------..C.........A...........C............... [810]

Phaeoceros ....------............A.......T...C.............C. [810]

Anomodon .T..------............A.......T................... [810]

Physcomitrella .T..------.........C..A.......T................... [810]

Treubia ....------............A.......T................... [810]

Marchantia ....------............A.......T................... [810]

[ 860 870 880 890 900]

[ . . . . .]

Huperzia_nad5 ATGATAGCAAGGTGCTCTCCTTTATTTGAATACTCACCCAATGCTTTGAT [860]

Huperzia_nad5_307277_307833 -------------------------------------------------- [0]

Huperzia_nad5_182779_182719 -------------------------------------------------- [0]

Huperzia_nad5_241849_241050 .........TTC.T.GAA....A.ACCA........G............. [485]

Huperzia_nad5_108187_107647 -------------------------------------------------- [541]

Isoetes ........G...C....C....C..CC............G.....CC... [848]

Zea_perennis .................C....................T.CG........ [851]

Arabidopsis .................C...............C....T.CG........ [851]

Cycas G................C....C.................CG....C... [860]

Megaceros ........G......C....C............C.G..T........... [860]

Phaeoceros ........G......CT..TC.............T...T........... [860]

Anomodon .................C........C.....T...T...C......... [860]

Physcomitrella .................C........C.....T.......C......... [860]

Treubia .................C................................ [860]

Marchantia .................C................................ [860]

[ 910 920 930 940 950]

[ . . . . .]

Huperzia_nad5 TGTTATTACTTTTATAGGAGCTATGACGTCATTCTTCGCGGCAACCATTG [910]

Huperzia_nad5_307277_307833 -------------------------------------------------- [0]

Huperzia_nad5_182779_182719 -------------------------------------------------- [0]

Huperzia_nad5_241849_241050 ............--.......................A.........C.. [533]

Huperzia_nad5_108187_107647 -------------------------------------------------- [541]

Isoetes ..C......C.C.G................G...CC...........C.. [898]

Zea_perennis .............GCG..................C.T..........C.. [901]

Arabidopsis ...........C.GC........C..........C.T..........C.. [901]

Cycas ...........C.GC........C...........C...........C.. [910]

Megaceros ....G.....CC.G..............C.......T..........CA. [910]

Phaeoceros ....G......C.G............T.CT...T..T..........CA. [910]

Anomodon ............CG....G............................C.. [910]

Physcomitrella ............CG....G............................C.. [910]

Treubia .A...........G....C............................C.. [910]

Marchantia .............G....C............................CC. [910]

[ 960 970 980 990 1000]

[ . . . . .]

Huperzia_nad5 GAATATTACAAAACGATT------TAAAGAGGATCATAGCTTATTCGACT [954]

Huperzia_nad5_307277_307833 -------------------------------------------------- [0]

Huperzia_nad5_182779_182719 -------------------------------------------------- [0]

Huperzia_nad5_241849_241050 .........G...AAT.GAATAAA....T...G............AA.T. [583]

Huperzia_nad5_108187_107647 -------------------------------------------------- [541]

Isoetes ......C...........------.G......G........C..C.A... [942]

Zea_perennis ..........G......C------........G.............A... [945]

Arabidopsis ..........G......C------........G....C........A... [945]

Cycas ..........G.......------C.......G...........C.A... [954]

Megaceros ..........G.......------.G......G.............C.T. [954]

Phaeoceros ..........G.......------.G......G............TC.T. [954]

Anomodon ..........G..T....------........G.......C.....A... [954]

Physcomitrella ..........G..T....------........G.......C..C..A... [954]

Treubia ..................------.G..A...G.T.....C.....A... [954]

Marchantia ..................------....A...G.T...........A... [954]

[ 1010 1020 1030 1040 1050]

[ . . . . .]

Huperzia_nad5 TGTAGTCAATTAGGCTATATGATCTTTGCTTGCGGCATTTC-----CAAC [999]

Huperzia_nad5_307277_307833 -------------------------------------------------- [0]

Huperzia_nad5_182779_182719 -------------------------------------------------- [0]

Huperzia_nad5_241849_241050 C.............................CA........TTCCAA.C.. [633]

Huperzia_nad5_108187_107647 -------------------------------------------------- [541]

Isoetes C.........C...............C...C.........T-----A... [987]

Zea_perennis ..C...................................C..-----T... [990]

Arabidopsis ......................................C..-----T... [990]

Cycas C.........C.............C................-----.... [999]

Megaceros ..........C...............C..............-----.... [999]

Phaeoceros ........................................T-----.... [999]

Anomodon .....C.................A...........T.....-----.... [999]

Physcomitrella .....C.................A...........T.....-----.... [999]

Treubia ........G................................-----.... [999]

Marchantia ........G................................-----.... [999]

[ 1060 1070 1080 1090 1100]

[ . . . . .]

Huperzia_nad5 TATTCCGTTAGCGTATTTCATTTAATGAATCATGCTTTTTTCAAAGCATT [1049]

Huperzia_nad5_307277_307833 -------------------------------------------------- [0]

Huperzia_nad5_182779_182719 -------------------------------------------------- [0]

Huperzia_nad5_241849_241050 .................CT.............C........TG....... [683]

Huperzia_nad5_108187_107647 -------------------------------------------------- [541]

Isoetes ....GT..........C.....C.........C...CC.CC........C [1037]

Zea_perennis .....G........C.....C...........C..G.............. [1040]

Arabidopsis .....G........C.....C...........C..C.............. [1040]

Cycas .....G.......CC.C...............C......CC......... [1049]

Megaceros .....T..............C..G...............C.T.......C [1049]

Phaeoceros .....T..............C..G......T..........T....T... [1049]

Anomodon ................................C........T........ [1049]

Physcomitrella ................................C........T........ [1049]

Treubia ................................C..C.GC.........C. [1049]

Marchantia .....T..........................C..C.GC.........C. [1049]

[ 1110 1120 1130 1140 1150]

[ . . . . .]

Huperzia_nad5 ACTTTTTTCGAGTGCAGGTTCAGTGATTCATGCTATGTCGGATGAGCAAG [1099]

Huperzia_nad5_307277_307833 -------------------------------------------------- [0]

Huperzia_nad5_182779_182719 -------------------------------------------------- [0]

Huperzia_nad5_241849_241050 ...A..AC..........................G....AA......... [733]

Huperzia_nad5_108187_107647 -------------------------------------------------- [541]

Isoetes ..CA.CC..............C....C........C.............. [1087]

Zea_perennis ...C..CCT......G.....G...........C................ [1090]

Arabidopsis ...A..CCT......T.....G...........C................ [1090]

Cycas ...A..C............C.............C................ [1099]

Megaceros ..CC....T..........C.............C................ [1099]

Phaeoceros ..CC.C..T..........C.............C............T... [1099]

Anomodon ........T........................C................ [1099]

Physcomitrella ...C...CT........................C................ [1099]

Treubia TT.A..C.T......................................... [1099]

Marchantia TT.A..C.T........................C................ [1099]

[ 1160 1170 1180 1190 1200]

[ . . . . .]

Huperzia_nad5 ATATGCGTAG---GATGGGAGGGCTTGCTTCCTTGTTACCTTTTACCTAT [1146]

Huperzia_nad5_307277_307833 -------------------------------------------------- [0]

Huperzia_nad5_182779_182719 -------------------------------------------------- [0]

Huperzia_nad5_241849_241050 .........ATAA.....A...........T................A.. [783]

Huperzia_nad5_108187_107647 -------------------------------------------------- [541]

Isoetes .........A---.......................C......C...... [1134]

Zea_perennis .......G.A---......G........C....CC..T.....G...... [1137]

Arabidopsis .......G.A---......G........C....CC..C.....G...... [1137]

Cycas .........A---......G.............C................ [1146]

Megaceros .........A---.........A.........C.......C......... [1146]

Phaeoceros .........A---.........A.........C.......C......... [1146]

Anomodon .........A---..............................C..T... [1146]

Physcomitrella .........A---..............................C..T... [1146]

Treubia .........A---............................C.C...... [1146]

Marchantia .........A---..................................... [1146]

[ 1210 1220 1230 1240 1250]

[ . . . . .]

Huperzia_nad5 GCTATGATGCCTATAGGCAGCTTATCTCTAATTGGATTCCCTTTTTTAAC [1196]

Huperzia_nad5_307277_307833 -------------------------------------------------- [0]

Huperzia_nad5_182779_182719 -------------------------------------------------- [0]

Huperzia_nad5_241849_241050 .................--------------------------------- [800]

Huperzia_nad5_108187_107647 -------------------------------------------------- [541]

Isoetes ..........T........T.C..C.CT....C..............C.. [1184]

Zea_perennis ..C.......TC..G..............T........T......C.... [1187]

Arabidopsis ..C.......TC..........................T......C.... [1187]

Cycas ..........TC................C.........T......CC... [1196]

Megaceros ..........T.................C...C.....T........... [1196]

Phaeoceros ..........T.................C...C.....T........... [1196]

Anomodon ..C.......T.........T......T....A..T..T......CGT.. [1196]

Physcomitrella ..A.......T......T.........T....A..T..........GT.. [1196]

Treubia ..........T........................T.............. [1196]

Marchantia ..........T......T.........T.......T.............. [1196]

[ 1260 1270 1280 1290 1300]

[ . . . . .]

Huperzia_nad5 TGGATTTTATTCCAAAGATGTAATTCCAGAGCTTGCTTACACTAAATATA [1246]

Huperzia_nad5_307277_307833 -------------------------------------------------- [0]

Huperzia_nad5_182779_182719 -------------------------------------------------- [0]

Huperzia_nad5_241849_241050 -------------------------------------------------- [800]

Huperzia_nad5_108187_107647 -------------------------------------------------- [541]

Isoetes ......CC..C.T........C...T.G..............C...CG.. [1234]

Zea_perennis .....................G..CTT......C........A..G.... [1237]

Arabidopsis .....................G..CTT......C...........G.... [1237]

Cycas .....................G..CT......CC...C.....C...... [1246]

Megaceros .....................G....TG....C................. [1246]

Phaeoceros .....................G....T.....C................. [1246]

Anomodon ............T........T...TT......C.....T.......... [1246]

Physcomitrella ............T........T...TT......C.....T.......... [1246]

Treubia G...........A........T...TTG..............G....... [1246]

Marchantia G...........A........T....TG..A...........G....... [1246]

[ 1310 1320 1330 1340 1350]

[ . . . . .]

Huperzia_nad5 CCATTAGTGGTAACTTTGCTTTCTGGTTGGGAAGTATTTCTGTTTTTTTT [1296]

Huperzia_nad5_307277_307833 -------------------------------------------------- [0]

Huperzia_nad5_182779_182719 -------------------------------------------------- [0]

Huperzia_nad5_241849_241050 -------------------------------------------------- [800]

Huperzia_nad5_108187_107647 -------------------------------------------------- [541]

Isoetes ...CA......C...C.......C...C......CG.A..C..A.....C [1284]

Zea_perennis ....C.....G........................G.C.....CC....C [1287]

Arabidopsis ....C.....G........................G.C.....CC....C [1287]

Cycas ....C................C.............G.C.....C....CC [1296]

Megaceros .A.............C...................G.C.....C..C..C [1296]

Phaeoceros .A.................................G.C.....C..C..C [1296]

Anomodon .G.................................G.......C..C... [1296]

Physcomitrella .G.........................................C..C... [1296]

Treubia .T..............C...........A......G.A..G..C..C... [1296]

Marchantia .T..........................A......G.A..G..C..C... [1296]

[ 1360 1370 1380 1390 1400]

[ . . . . .]

Huperzia_nad5 ACTTCTTATTATTCTTTTCGTTTACTTTTTCTCACATTTCTAGCACCCAC [1346]

Huperzia_nad5_307277_307833 -------------------------------------------------- [0]

Huperzia_nad5_182779_182719 -------------------------------------------------- [0]

Huperzia_nad5_241849_241050 -------------------------------------------------- [800]

Huperzia_nad5_108187_107647 -------------------------------------------------- [541]

Isoetes ..CC.CC..C.C..C..C............T.G...CC.TC......A.. [1334]

Zea_perennis ...........C..C...........A.....A..........T...A.. [1337]

Arabidopsis ...........C....................A..........T...A.. [1337]

Cycas ...C.....C.C..........C........CA.......CG.....A.. [1346]

Megaceros ........CC.C.TC.....C.....C..CT.A.....CT.......A.. [1346]

Phaeoceros ........CC.C.TC.....C........CT.A......T.......A.. [1346]

Anomodon .....C...............C.....C..T.A..T...T.......A.. [1346]

Physcomitrella ..............................T.A..T...T.......A.. [1346]

Treubia ...........C........G.........T.A..C.............. [1346]

Marchantia ...........C..............G...T.A..C...T.......A.. [1346]

[ 1410 1420 1430 1440 1450]

[ . . . . .]

Huperzia_nad5 TAATTCATTCAAGCGAGACATCTTACGATGTCATGATGCGCCCATTCTTA [1396]

Huperzia_nad5_307277_307833 -------------------------------------------------- [0]

Huperzia_nad5_182779_182719 -------------------------------------------------- [0]

Huperzia_nad5_241849_241050 -------------------------------------------------- [800]

Huperzia_nad5_108187_107647 -------------------------------------------------- [541]

Isoetes ....C....TTC...................................... [1384]

Zea_perennis ..........GG........GA.........................C.. [1387]

Arabidopsis ......G...GG...........C.......................C.. [1387]

Cycas ....C.....GG...................................C.. [1396]

Megaceros C.............A......G..........................C. [1396]

Phaeoceros .............T.......G...T......................C. [1396]

Anomodon A................................................. [1396]

Physcomitrella A................................................. [1396]

Treubia ...................T.AAGTA........................ [1396]

Marchantia ...................T.AAGTA........................ [1396]

[ 1460 1470 1480 1490 1500]

[ . . . . .]

Huperzia_nad5 TGGCCATCCCTTTAATCCTTTTGGCTTTTGGAAGTATTTTCGTAGGATAC [1446]

Huperzia_nad5_307277_307833 -------------------------------------------------- [0]

Huperzia_nad5_182779_182719 -------------------------------------------------- [0]

Huperzia_nad5_241849_241050 -------------------------------------------------- [800]

Huperzia_nad5_108187_107647 -------------------------------------------------- [541]

Isoetes C..........CC.......C..............C....T.C....... [1434]

Zea_perennis .......T........A.........C.C..G...C.C..T......... [1437]

Arabidopsis .......T........A...C.....C.C..G...C.C..T......... [1437]

Cycas .......T....C....T........CCC..G...C....T......... [1446]

Megaceros ....A............TG....................CT......C.. [1446]

Phaeoceros ....A....T.......TG.....................T......... [1446]

Anomodon ....A............T......................T......... [1446]

Physcomitrella ....A............T....A.................T......... [1446]

Treubia ....A..A..C....................G........T......... [1446]

Marchantia ....A..A.......................G........T......... [1446]

[ 1510 1520 1530 1540 1550]

[ . . . . .]

Huperzia_nad5 TTGGCCAAAGATATGATGATTGGTTTAGGTACCAATTTTTGGGCTAATTC [1496]

Huperzia_nad5_307277_307833 -------------------------------................... [19]

Huperzia_nad5_182779_182719 -------------------------------------------------- [0]

Huperzia_nad5_241849_241050 -------------------------------------------------- [800]

Huperzia_nad5_108187_107647 -------------------------------------------------- [541]

Isoetes .................---.........C.......CCC.......... [1481]

Zea_perennis ............................................C..... [1487]

Arabidopsis ............................................C..... [1487]

Cycas ............................................C..... [1496]

Megaceros .......................C........T................. [1496]

Phaeoceros .......................C........T...........C....T [1496]

Anomodon G................................................. [1496]

Physcomitrella G................................................. [1496]

Treubia .................................................. [1496]

Marchantia .................................................. [1496]

[ 1560 1570 1580 1590 1600]

[ . . . . .]

Huperzia_nad5 CCTTTTCATACTACCCCCAAATGAGATTATTGCCGAATCTGAGTTTGCTA [1546]

Huperzia_nad5_307277_307833 .................................................. [69]

Huperzia_nad5_182779_182719 -------------------------------------------------- [0]

Huperzia_nad5_241849_241050 -------------------------------------------------- [800]

Huperzia_nad5_108187_107647 -------------------------------------------------- [541]

Isoetes ..C.............TA.------------........C.......... [1519]

Zea_perennis ..CC...G.......AAA..........C..........C.........G [1537]

Arabidopsis ..CCC..G.......AAA..........C..........C.........G [1537]

Cycas ..C....G........AA.........CC....................G [1546]

Megaceros TG........TC....AA..........C....T................ [1546]

Phaeoceros T.........TC....AA..........C....T................ [1546]

Anomodon T..............AAA......A..............C.......... [1546]

Physcomitrella ...............AAA......A...C..........C.......... [1546]

Treubia A................A......A...C.G................... [1546]

Marchantia A...............AA......A...C.G................... [1546]

[ 1610 1620 1630 1640 1650]

[ . . . . .]

Huperzia_nad5 CTCCAACCATTATCAAACTAATACCTATTTCCTTTAGTACTCTAGGTGCT [1596]

Huperzia_nad5_307277_307833 .................................................. [119]

Huperzia_nad5_182779_182719 -------------------------------------------------- [0]

Huperzia_nad5_241849_241050 -------------------------------------------------- [800]

Huperzia_nad5_108187_107647 -------------------------------------------------- [541]

Isoetes ..................C...........TG.CC.......C....... [1569]

Zea_perennis ............CA...............CTG.........TC....... [1587]

Arabidopsis ............C................CTG.........TC....... [1587]

Cycas ..............G..............C.G.C.......T.......C [1596]

Megaceros .............A........T.....CCTG.........T....C..A [1596]

Phaeoceros ......................T......CTG.........T....C..A [1596]

Anomodon .......A.....T........T.......TG.........TC....... [1596]

Physcomitrella .......A.....T........T...C...TG.........T........ [1596]

Treubia ....................C.T...C...TT.........T.....T.A [1596]

Marchantia ...................C..T.......TG.........T.....T.A [1596]

[ 1660 1670 1680 1690 1700]

[ . . . . .]

Huperzia_nad5 TTTCTGGCGTATAATGTCAATTTTGTAGCAAATTAATTCATTTTCGCTTT [1646]

Huperzia_nad5_307277_307833 .................................................. [169]

Huperzia_nad5_182779_182719 -------------------------------------------------- [0]

Huperzia_nad5_241849_241050 -------------------------------------------------- [800]

Huperzia_nad5_108187_107647 -------------------------------------------------- [541]

Isoetes CC.GC.........C......C.C........GAG..C...........C [1619]

Zea_perennis .C...A...........A...C.C.....GG..C.....CAACGA..C.. [1637]

Arabidopsis ...G.T...........A...CCC.....GG..C.....CAACGA..C.. [1637]

Cycas ...G.............A...C.C.....GG..C.....CAACGA..C.. [1646]

Megaceros ...G....................A........G....T..C........ [1646]

Phaeoceros ...G.............A......A........G....T..C........ [1646]

Anomodon ...A....A......A.A...............CC.........T..... [1646]

Physcomitrella ...A....A......A.A...............CC...A.....T..... [1646]

Treubia ...GC........G...A........G.TG...CC.C.......T..... [1646]

Marchantia ...G.........G...A........G.TT...CC.C............. [1646]

[ 1710 1720 1730 1740 1750]

[ . . . . .]

Huperzia_nad5 GAAAACTAGTACTTTTGGTAATCGACTTTATTGCTTTTTGAATAAGCGCT [1696]

Huperzia_nad5_307277_307833 .................................................. [219]

Huperzia_nad5_182779_182719 -------------------------------------------------- [0]

Huperzia_nad5_241849_241050 -------------------------------------------------- [800]

Huperzia_nad5_108187_107647 -------------------------------------------------- [541]

Isoetes .......G.---...G.......A...CC..C...CC.....C......C [1666]

Zea_perennis TC..............T..........C...A....C..C.....A.... [1687]

Arabidopsis TC..............T..........C...A....C..C.....A.... [1687]

Cycas T.G...........C............C........C..A.....A.... [1696]

Megaceros ......A.C.T...........G....A........C..A........T. [1696]

Phaeoceros ......A.CCT...........G....A........C..A........T. [1696]

Anomodon ...............G.........T.A...........A.........C [1696]

Physcomitrella ...............C.........T.A...........A.......... [1696]

Treubia ...........................A...........C.......... [1696]

Marchantia ...........................A...........C.......... [1696]

[ 1760 1770 1780 1790 1800]

[ . . . . .]

Huperzia_nad5 GGTTTTTTGATAAAGTTTTCCATGACTTTCTAGTGAGATGGTTTTTGCGT [1746]

Huperzia_nad5_307277_307833 .................................................. [269]

Huperzia_nad5_182779_182719 -------------------------------------------------- [0]

Huperzia_nad5_241849_241050 -------------------------------------------------- [800]

Huperzia_nad5_108187_107647 -------------------------------------------------- [541]

Isoetes ...A...C.....G....CGA..............G.....C........ [1716]

Zea_perennis ....C..C...C.......GA.............C....C...CC..... [1737]

Arabidopsis ....C..C...C.......GA.............C....C...CC..... [1737]

Cycas ....C.CC...C.......TA........A....C....C...CCC.... [1746]

Megaceros ....C...........C..TA.......CA.........CT.....A... [1746]

Phaeoceros ....C...........G..TA.......CA.........CC.....A... [1746]

Anomodon ..............C.....A........A....C..G.TC......... [1746]

Physcomitrella ..............A....TA........A..C.T..G.TC......... [1746]

Treubia .......C...........TA.C......T...CT....CA......... [1746]

Marchantia .......C...........TA.C......T...CT....C.......... [1746]

[ 1810 1820 1830 1840 1850]

[ . . . . .]

Huperzia_nad5 TTTGGCTACGAAGTCTCATTCAAAGCTTTAGACAAGGGTGCTATTGAGAT [1796]

Huperzia_nad5_307277_307833 .................................................. [319]

Huperzia_nad5_182779_182719 -------------------------------------------------- [0]

Huperzia_nad5_241849_241050 -------------------------------------------------- [800]

Huperzia_nad5_108187_107647 -------------------------------------------------- [541]

Isoetes C.C...C.T.....A....CT........G........C........... [1766]

Zea_perennis ..C..A..TTC..........G............................ [1787]

Arabidopsis ..C..A..T............G.............A.............. [1787]

Cycas ..C..A..T............G......C......A.............. [1796]

Megaceros .....A..T.....G.....T..........................A.. [1796]

Phaeoceros .....A..T.....G.....T..........................A.. [1796]

Anomodon .....A..T.....T.....T....T.....................A.. [1796]

Physcomitrella .....A..T.....T.....T....T.....................A.. [1796]

Treubia ..C..A..T.....T.....T..............A...........A.. [1796]

Marchantia .....A..T...........T..............A...........A.. [1796]

[ 1860 1870 1880 1890 1900]

[ . . . . .]

Huperzia_nad5 CTTGGGTCCTTATGGAATTTCCGTA-ACATTCCGAAGATTAGCCAAGCAA [1845]

Huperzia_nad5_307277_307833 .........................-........................ [368]

Huperzia_nad5_182779_182719 -------------------------------------------------- [0]

Huperzia_nad5_241849_241050 -------------------------------------------------- [800]

Huperzia_nad5_108187_107647 -------------------------------------------------- [541]

Isoetes ...A.....ACC...G.....-...C....C.T...T...G....G.G.. [1815]

Zea_perennis A.....C........T..C..-...C.........C....G...G...G. [1836]

Arabidopsis A.....C........T..C..-...C.........C....G...G...G. [1836]

Cycas A.....C........T.....-...C.........CA...G.......G. [1845]

Megaceros ...............G.....-...C.....TG..GA......T...T.. [1845]

Phaeoceros ...............G.....-...C.....TG..GA......T...T.. [1845]

Anomodon .C....A..............-...C.....T....A............G [1845]

Physcomitrella ......G..............-...C.....T....A............G [1845]

Treubia .....................-...C...A.T....A.A.G...C..... [1845]

Marchantia .....................-...C...A.T....A.A.G...C..... [1845]

[ 1910 1920 1930 1940 1950]

[ . . . . .]

Huperzia_nad5 ATGAGTCAACTTCAAAGTGGATTTGTTTATCATTACGCCTTTGTTATGTT [1895]

Huperzia_nad5_307277_307833 .................................................. [418]

Huperzia_nad5_182779_182719 -------------.....A.......C.....C..T.......G...... [37]

Huperzia_nad5_241849_241050 -------------------------------------------------- [800]

Huperzia_nad5_108187_107647 -------------------------------------------------- [541]

Isoetes ..A...A...............CC......T........C....C.C..C [1865]

Zea_perennis ..A...................C............T.......CA..... [1886]

Arabidopsis ..A................................T.......CA..... [1886]

Cycas ..A...A................C...........T.......CA..... [1895]

Megaceros ..A...A.....T.........................T..C.C....C. [1895]

Phaeoceros ..A...A.....T.........................T..C.C....C. [1895]

Anomodon ..A...A.............T..............T..T.....A..... [1895]

Physcomitrella ..A...A.............T..............T..T.....A..... [1895]

Treubia ..A......A.........................T.............. [1895]

Marchantia ..A...A..A.........................T.............. [1895]

[ 1960 1970 1980 1990 2000]

[ . . . . .]

Huperzia_nad5 ACTTGGATTAACTATATTTATGACCATTATAGGTCTGTGGGATTTTCTCT [1945]

Huperzia_nad5_307277_307833 .................................................. [468]

Huperzia_nad5_182779_182719 ........................-------------------------- [61]

Huperzia_nad5_241849_241050 -------------------------------------------------- [800]

Huperzia_nad5_108187_107647 -------------------------------------------------- [541]

Isoetes ..CC....C..........................C............T. [1915]

Zea_perennis ......T.C....CC....G.....T..TCTC..A.......C.C...A. [1936]

Arabidopsis ......T.C....C.....G.....T..TCTC..A.......C.C...A. [1936]

Cycas ......TCC..........G.....T..TCTC..A...........A.A. [1945]

Megaceros GA....T.....C........T..T......................... [1945]

Phaeoceros GA....T.....C........T..T.....................A... [1945]

Anomodon GA.C..C.....C........T.....A.......C..........A.T. [1945]

Physcomitrella GA....C.....C........T.....A..................A.T. [1945]

Treubia G..C........G........TT.TG.A.....C............A... [1945]

Marchantia G..C.................TT..G.......C............A... [1945]

[ 2010 2020 2030 2040 2050]

[ . . . . .]

Huperzia_nad5 CTTTTTGGGTAGATAATCGATTGTATTTTATTTACATCGTCAGTTTTCTA [1995]

Huperzia_nad5_307277_307833 .................................................. [518]

Huperzia_nad5_182779_182719 -------------------------------------------------- [61]

Huperzia_nad5_241849_241050 -------------------------------------------------- [800]

Huperzia_nad5_108187_107647 -------------------------------------------------- [541]

Isoetes G...CC...............C......AC.......A------.A...G [1959]

Zea_perennis ...C..........TC.A...C..C...C....TGT.A..G...AG.T.T [1986]

Arabidopsis ...C.................C..C...C....TG..A..G...AG.T.- [1985]

Cycas ...C.................C..C...C....CG..A..G...AG.T.C [1995]

Megaceros .C...............A..................CA..G.C...CTCT [1995]

Phaeoceros .................A..................CA..G.C...CTCT [1995]

Anomodon ....C................................A..G......... [1995]

Physcomitrella .....................................A..G......... [1995]

Treubia .C..C................................A..T..C...T.. [1995]

Marchantia ............................C........A..T..C...T.. [1995]

[ 2060 2070 2080 ]

[ . . . ]

Huperzia_nad5 TTTATTAATTTATCTAAACACCACATTAGCACGAACCAA [2034]

Huperzia_nad5_307277_307833 ....................................... [557]

Huperzia_nad5_182779_182719 --------------------------------------- [61]

Huperzia_nad5_241849_241050 --------------------------------------- [800]

Huperzia_nad5_108187_107647 --------------------------------------- [541]

Isoetes A..C.CC.CAG------------..CAC.A.TT..TT.. [1986]

Zea_perennis A........AAG.------------CA..TCAAG.AT.. [2013]

Arabidopsis -....-.CAAAG.------------CA..TCAAG.AT.. [2010]

Cycas ..CCC------------------------------AT.G [2004]

Megaceros .........C.---.G.GGGAG.....C....A..TT.. [2031]

Phaeoceros .........C.---.G..GGAG..........A..TT.. [2031]

Anomodon ......C....---.G..A..G..............T.. [2031]

Physcomitrella .....NC....---.G..A.AG..........A...T.. [2031]

Treubia .........A.------------------------.T.. [2010]

Marchantia .........A.------------------------.T.. [2010]

;

END;

**nad6**

#NEXUS

[MacClade 4.05 registered to Yin-Long Qiu Lab, EEB, University of Michigan]

BEGIN DATA;

DIMENSIONS NTAX=14 NCHAR=752;

FORMAT DATATYPE=DNA MISSING=? GAP=- MATCHCHAR=. INTERLEAVE ;

MATRIX

[ 10 20 30 40 50]

[ . . . . .]

Huperzia_nad6 ATGATACTT--TTTTCCGTTT---TTTCGAGCATTGCTTTAGTCTCTGGT [45]

Huperzia_nad6_327478_328026 -.....T.C--..........---C.C.................C....C [44]

Huperzia_nad6_289060_288864 -........CT..........CTT.....G........----------AC [39]

Huperzia_nad6_186215_185970 -----....--...G.TC...---..C..................T.... [40]

Isoetes .C.......--..C..T....---..........C....C.....T.... [45]

Arabidopsis .........-----..T....---.G......CC......G......... [42]

Zea_perennis .....T...-----.......---.G......CC......G......... [42]

Cycas .C.......-----..T....---CG......CC......G...C..... [42]

Megaceros .........--.....T...C---C.....................C... [45]

Phaeoceros .........--.....T...C---C.....................C... [45]

Anomodon .........--.....T....---.......................A.. [45]

Physcomitrella .........--.....T....---.......................A.. [45]

Treubia .........--....AT..C.---..GT.GT.C.C........G..A..C [45]

Marchantia .........--....AT....---..GT.GT.C.C........G..A..C [45]

[ 60 70 80 90 100]

[ . . . . .]

Huperzia_nad6 GTTATGGTT--ATACGTGCCAAAAATCCAGTCCATTCAGTTTTATTTCCA [93]

Huperzia_nad6_327478_328026 A........--.C...........T.GAG..G.....-AC.C.T.C.T.G [91]

Huperzia_nad6_289060_288864 ...GC...A--........------.T.TC.T..G..CA........TT. [81]

Huperzia_nad6_186215_185970 ....C....TT...............ATG..T..........A..G.T.- [89]

Isoetes ..G.....G--..G............T.....T..CTG....C.C..... [93]

Arabidopsis T.G......--GC......T........G..A.....C.....G.....C [90]

Zea_perennis T.G......--G.......T........G..A.....C.....G.....C [90]

Cycas T.G......--G.......TG..........A...C.C....CG..C..C [90]

Megaceros ....C..CG--.C........................C.........T.. [93]

Phaeoceros ....C..CG--.C..............T........TC.........TT. [93]

Anomodon ........A--........T.................T.........TTC [93]

Physcomitrella ........A--........T.................T.........TTC [93]

Treubia .C......G--....................T.....T.........TT. [93]

Marchantia .C......G--....................T.....T.........TT. [93]

[ 110 120 130 140 150]

[ . . . . .]

Huperzia_nad6 ATCTC-AGTCTTTTGCAACACTTC----TGGGTTACTTCTTTTGTTAGGT [138]

Huperzia_nad6_327478_328026 CCA.T--T.T......C.G.....CTTCAA.T.G..---......G.... [136]

Huperzia_nad6_289060_288864 T.T.TTCT.T......T..GTC.T----A..T....---......G.... [124]

Huperzia_nad6_186215_185970 -..G.--A.T.A....C.......----C..T..C.C......A...... [132]

Isoetes ...CT-...TCC.C..C....C..----A..TCC..CC...CG..C.... [138]

Arabidopsis ...C.-.......C..G.......----A..T.................. [135]

Zea_perennis ...CT-..........G.......----...T......A........... [135]

Cycas ...C.-...G...C..........----A..T......A......C.... [135]

Megaceros ..AC.-C......C..........----A..T......G.....C..... [138]

Phaeoceros ..AC.-C......C....T.....----A..T......G........... [138]

Anomodon ....T-...T....T.........----G..T......G........... [138]

Physcomitrella ...CT-...T....T.........----G..T......G........... [138]

Treubia ...CT-...T........T.....----C........CG........... [138]

Marchantia ...CT-...T........T.....----C.........G........... [138]

[ 160 170 180 190 200]

[ . . . . .]

Huperzia_nad6 CTTGAC---TTCTTTGCTATGATTTTTCTAGTGGTTTAT---GTAGGAGC [182]

Huperzia_nad6_327478_328026 ......GAC.................AT...C.T.....---..-..... [182]

Huperzia_nad6_289060_288864 ...A..---C.A...............T.........T.---.C...... [168]

Huperzia_nad6_186215_185970 ......---...C..............TC..C.......TAT.C.A.... [179]

Isoetes ......---...CC.C.C.C....C.CTC..C....C..---........ [182]

Arabidopsis ..C...---.....C........C..C.C...A...C..---A....... [179]

Zea_perennis ..C...---....CC........C..C.C...A...C..---A....... [179]

Cycas .C....---C...C.........C.CC.C...A...C..---A....... [179]

Megaceros ..C...---CC...C............T...........---........ [182]

Phaeoceros ..C...---.C...C............T...........---.C...... [182]

Anomodon ..C...---..............C...T...........---........ [182]

Physcomitrella ......---..................T...........---........ [182]

Treubia ......---..................T...........---........ [182]

Marchantia ......---..................T...........---........ [182]

[ 210 220 230 240 250]

[ . . . . .]

Huperzia_nad6 TATTGCCGTTTCATCTTCACCCGTAGCTATGATGTCAAATATAAAAATAG [232]

Huperzia_nad6_327478_328026 ..C...A.........CTTT.T....T....C...T.............. [232]

Huperzia_nad6_289060_288864 .......................C.....--------------------- [197]

Huperzia_nad6_186215_185970 A..........T......C..T.C.....C.GCA.....A.G........ [229]

Isoetes C.....T...C..C..C............C..C.C.......C....... [232]

Arabidopsis ...A..........TCCTTTT...T.T........TCC....TC...... [229]

Zea_perennis ..............TCCT.TT...G.T........TC.....TC...... [229]

Cycas ..........CT..TC...T....G.T........T......TC...... [229]

Megaceros ......T......C.....T.T.......C..C...G.....T....... [232]

Phaeoceros ......T.C.......C..T.T..........C.........T....... [232]

Anomodon ..........CT..T....TTT....T........TG.....T....... [232]

Physcomitrella ...........T..T..T.TTT....T........T......T....... [232]

Treubia ......T....T..T..TGTTT..C.T........T.C.....GG...G. [232]

Marchantia ...........T..T..TGTTT..C.T........T.C......GG.... [232]

[ 260 270 280 290 300]

[ . . . . .]

Huperzia_nad6 CACAAATTCACGAAAATGTATTGCGCTATTTACCAGTAGGTGGTATTATT [282]

Huperzia_nad6_327478_328026 ...G...CG..A.G..................................C. [282]

Huperzia_nad6_289060_288864 -------------------------------------------------- [197]

Huperzia_nad6_186215_185970 ........T....C...--------------------------------- [246]

Isoetes ..G.G.........G...............C................... [282]

Arabidopsis .GG.G.........G.A....................GA....G...... [279]

Zea_perennis .GG.G.........G.A....................GA........... [279]

Cycas ..G.G...........A....C........C......GA.....C..... [279]

Megaceros ..G..........G....................T.............C. [282]

Phaeoceros ..G..........G....................T..G............ [282]

Anomodon ..G..........G...........T........T............... [282]

Physcomitrella ..G..........G...........T........T............... [282]

Treubia A.G..........G....................T............... [282]

Marchantia A.G..........G....................T............... [282]

[ 310 320 330 340 350]

[ . . . . .]

Huperzia_nad6 GGACTAATTTTTCTGTGGGAAATCTTTCTGATTGTAGATAATGATTACAT [332]

Huperzia_nad6_327478_328026 .....T....G.TC....A....T...T.T...........CA.----G. [328]

Huperzia_nad6_289060_288864 -------------------------------------------------- [197]

Huperzia_nad6_186215_185970 -------------------------------------------------- [246]

Isoetes ...TCC....CGGG.C.......G....C..................... [332]

Arabidopsis .....G..C...TG.........G...T.C...T..........AAG... [329]

Zea_perennis .....G..C...TG.........G..CT.C...T..........AAC... [329]

Cycas ....CG..CC...G.........G..C..C..CC...........C.... [329]

Megaceros .....T...C..T...T......A...T.C..C................. [332]

Phaeoceros .....T...C..T..CT..........T.T.................... [332]

Anomodon ...G.T......T...T......T..CT.C.................... [332]

Physcomitrella ...G.T......T...T......T...T.C.................... [332]

Treubia .....T..........T..........T.A..G................. [332]

Marchantia .....T......T...T..........T.A..G................. [332]

[ 360 370 380 390 400]

[ . . . . .]

Huperzia_nad6 CCCAATACTACCAACGGAATTGAATACAACCTATCCAACATATACAGTTT [382]

Huperzia_nad6_327478_328026 T......TC......AA.............TA...T.............. [378]

Huperzia_nad6_289060_288864 -------------------------------------------------- [197]

Huperzia_nad6_186215_185970 -------------------------------------------------- [246]

Isoetes T..G...T..T.....A.TA.......--.TC...----......G.... [376]

Arabidopsis T...T..........CC..AGA.....G....C..TT.G......G.... [379]

Zea_perennis T...T..........CC.CAGA.....G....C..TG.A......G.... [379]

Cycas T...TC.T....G..CT.CA.AGG........C..TG.G..........C [379]

Megaceros T......T........A..C...G...........T.............. [382]

Phaeoceros T......T........A..C...G....G......T.............. [382]

Anomodon T......T........A......G..........TT.............. [382]

Physcomitrella T......T........A......G......T...TT.............. [382]

Treubia T......T........A........G.........T.............. [382]

Marchantia T......T........A......G.G.........T.............. [382]

[ 410 420 430 440 450]

[ . . . . .]

Huperzia_nad6 ATGCTGGAAAATTACAAAGTTGGACTAATATGGAAA-CATTAGGCAATTT [431]

Huperzia_nad6_327478_328026 ...........A.C...............T......A........C.... [428]

Huperzia_nad6_289060_288864 -------------------------------------------------- [197]

Huperzia_nad6_186215_185970 -------------------------------------------------- [246]

Isoetes G......G..GA.CT..............TC.....-..A.......... [425]

Arabidopsis ....C.....GG...G.............T......-....G........ [428]

Zea_perennis ....C.....GG...G.............T......-....G........ [428]

Cycas ........G.GA.................TC.....-....G........ [428]

Megaceros ..........GA..T..............T......-.G........... [431]

Phaeoceros ..........GA..T..............T......-.C........... [431]

Anomodon ......A...GA.................T......-............. [431]

Physcomitrella ......A...GA.................T......-............. [431]

Treubia ..........GA....T............T....G.-............. [431]

Marchantia ..........GA....T............T....G.-............. [431]

[ 460 470 480 490 500]

[ . . . . .]

Huperzia_nad6 ACTTTATACCACCTATTTGGTTCTGTTTCTGGTTTCTAGTCTTATTTTAT [481]

Huperzia_nad6_327478_328026 .........-....C.......T.-.C.......CT....A......A.C [476]

Huperzia_nad6_289060_288864 -------------------------------------------------- [197]

Huperzia_nad6_186215_185970 -------------------------------------------------- [246]

Isoetes ..C........T...........G.....C....C.C.........CC.. [475]

Arabidopsis ..........TA.....CT..CTG....T.....C............... [478]

Zea_perennis ..........TA.....CC..CTG....T.............G....... [478]

Cycas ....C.....TA.....CC....G...C.............C.G..CC.. [478]

Megaceros ..................TAC.TC...CT..T........T.C....C.. [481]

Phaeoceros ..................TAC.T....CT..T........T......... [481]

Anomodon ..................T.........T..................... [481]

Physcomitrella ..................T...T.....T..................... [481]

Treubia .........A........CT.CT.....T..................... [481]

Marchantia .........A........TT.CT.....T.............A....... [481]

[ 510 520 530 540 550]

[ . . . . .]

Huperzia_nad6 TAGTAGCC--ATGATTGGGGCTAT---AGTACTTACTATG---CATAAAA [523]

Huperzia_nad6_327478_328026 ........CC...CA........CTAC...........C.---...G... [523]

Huperzia_nad6_289060_288864 -------------------------------------------------- [197]

Huperzia_nad6_186215_185970 -------------------------------------------------- [246]

Isoetes ........--.C..CC.....A.C---...CA........GAG....G.. [520]

Arabidopsis ........--..............---......G......---....GG. [520]

Zea_perennis .......T--..............---......G......---....GG. [520]

Cycas ........--..............---......G......---....GG. [520]

Megaceros .G......--.............C---.............---....G.. [523]

Phaeoceros .G......--.............C---.............---....... [523]

Anomodon .......T--..............---.............---....... [523]

Physcomitrella ........--........A.....---.............---....... [523]

Treubia .......A--C.T........A..---.........G...---....... [523]

Marchantia .......A--C.T........A..---.........G...---....... [523]

[ 560 570 580 590 600]

[ . . . . .]

Huperzia_nad6 CGACTCAAGTAAAAATACAAGAT--GTGTTCCGACAGAATGCTATAGATT [571]

Huperzia_nad6_327478_328026 .A....G...C...T....G..G--...---------------------- [549]

Huperzia_nad6_289060_288864 -------------------------------------------------- [197]

Huperzia_nad6_186215_185970 -------------------------------------------------- [246]

Isoetes .A..AAG...C....G...GA..TG..CC.TTCT.GA.GAA.CGAGC.AG [570]

Arabidopsis .T...A.G..G....G...G...--..A.......GA........T.... [568]

Zea_perennis .T..AA.G..G....G...G...--..A.......GA.....CT.G.... [568]

Cycas .T...A.G..G..G.G...G...--..A.......GA........T.... [568]

Megaceros .T....GG..C....G.......--...........A.......G..... [571]

Phaeoceros .T....GG..C....G.......--...........A.......G..... [571]

Anomodon .T....G...C....G...G...--...........A............. [571]

Physcomitrella .T........C....G...G...--...........A............. [571]

Treubia .....A.G..C...CGT..G...--..T...AT...A............. [571]

Marchantia .....A.G..C...CGT..G...--..T...AT...A............. [571]

[ 610 620 630 640 650]

[ . . . . .]

Huperzia_nad6 TTCATAAAACTA----------TAAAAGCGGTTACGCACTCACCTAAGTG [611]

Huperzia_nad6_327478_328026 -------------------------------------------------- [549]

Huperzia_nad6_289060_288864 -------------------------------------------------- [197]

Huperzia_nad6_186215_185970 -------------------------------------------------- [246]

Isoetes AGA.GC.GC.AG---------G.T.GG.TTTG.TG..---------.AG. [602]

Arabidopsis ..AGG.GG....-------TAA.G.GGA---------------------. [590]

Zea_perennis C.AGG.GG....CTATTCATAT.C.G.A.--------------------A [598]

Cycas C.AGG.GG..C.-------TAA.G.GGA---------------------. [590]

Megaceros ...GG..T....----------.....A---------------------- [589]

Phaeoceros ....G..T....----------.....A---------------------- [589]

Anomodon ..G.A..T...G----------.C...A---------------------- [589]

Physcomitrella ..A.A..T....----------.C...A---------------------- [589]

Treubia ....G..T....----------.C.G.A---------------------- [589]

Marchantia ....G..T....----------.C...A---------------------- [589]

[ 660 670 680 690 700]

[ . . . . .]

Huperzia_nad6 CAAATCATGTGGCGCATCGGCGAGGGGAGTAGGATTCTTGGCGGGCGGCT [661]

Huperzia_nad6_327478_328026 -------------------------------------------------- [549]

Huperzia_nad6_289060_288864 -------------------------------------------------- [197]

Huperzia_nad6_186215_185970 -------------------------------------------------- [246]

Isoetes G..GGTGCCC.A..GTC.CCAATAA------------------------- [627]

Arabidopsis G.CGA.TGA-----.CCACT.AC.ATCTAC-----.AA------------ [618]

Zea_perennis G..GGTTC------TTCTCC.ACAAA..AAG..GA.GA------------ [630]

Cycas G.CGA.GG---------ACCAACAA------------------------- [606]

Megaceros -......----.AAAGATCTGA---------------------------- [606]

Phaeoceros -......----.AAAGATCTGA---------------------------- [606]

Anomodon -......----.A.ATATTTGA---------------------------- [606]

Physcomitrella -......----.A.ATATTTGA---------------------------- [606]

Treubia -..G.TC----.------TTGA---------------------------- [600]

Marchantia -..G.TC----.------TTGA---------------------------- [600]

[ 710 720 730 740 750]

[ . . . . .]

Huperzia_nad6 TCAACGCATCGTTCATACTGGGCTTTTCATCTACCAACACCGGAGGATAG [711]

Huperzia_nad6_327478_328026 -------------------------------------------------- [549]

Huperzia_nad6_289060_288864 -------------------------------------------------- [197]

Huperzia_nad6_186215_185970 -------------------------------------------------- [246]

Isoetes -------------------------------------------------- [627]

Arabidopsis -------------------------------------------------- [618]

Zea_perennis -------------------------------------------------- [630]

Cycas -------------------------------------------------- [606]

Megaceros -------------------------------------------------- [606]

Phaeoceros -------------------------------------------------- [606]

Anomodon -------------------------------------------------- [606]

Physcomitrella -------------------------------------------------- [606]

Treubia -------------------------------------------------- [600]

Marchantia -------------------------------------------------- [600]

[ ]

[ ]

Huperzia_nad6 TA [713]

Huperzia_nad6_327478_328026 -- [549]

Huperzia_nad6_289060_288864 -- [197]

Huperzia_nad6_186215_185970 -- [246]

Isoetes -- [627]

Arabidopsis -- [618]

Zea_perennis -- [630]

Cycas -- [606]

Megaceros -- [606]

Phaeoceros -- [606]

Anomodon -- [606]

Physcomitrella -- [606]

Treubia -- [600]

Marchantia -- [600]

;

END;

**nad9**

#NEXUS

[MacClade 4.05 registered to Yin-Long Qiu Lab, EEB, University of Michigan]

BEGIN DATA;

DIMENSIONS NTAX=12 NCHAR=598;

FORMAT DATATYPE=DNA MISSING=? GAP=- MATCHCHAR=. INTERLEAVE ;

MATRIX

[ 10 20 30 40 50]

[ . . . . .]

Huperzia_nad9 ACG---AACCAATTGTTTTTCAAATTTATGAAAGCTACTTTACC---TAA [44]

Huperzia_nad9_30298_30655 --.---.....................................T---.G. [42]

Isoetes ...---..............A....C.C...C.....---....---... [41]

Zea_perennis .T.GAT.......CCA.........A..GTTGG.AG.T......CAAG.. [50]

Arabidopsis .T.GAT........CA.........A..GTTGG.AG........CAAG.. [50]

Cycas .T.GAT.........A..CC.....A.C.A.G..A.....C...CAG... [50]

Megaceros .T.GAT..G.........C......C.C...T............---G.. [47]

Phaeoceros .T.GAT..G.........C......C.C...T............---G.. [47]

Anomodon GT.GAT.......C...........C.C...T............---G.. [47]

Physcomitrella GT.GAT.......C...........C.C...T............---A.. [47]

Treubia .T.GAT...................C.C...T...C........---G.. [47]

Marchantia .T.GAT...................C.C...T...C........---A.. [47]

[ 60 70 80 90 100]

[ . . . . .]

Huperzia_nad9 ACGGATAAATCAATGTCAAACATCAAAACAAGAAAATCTATTATATACCA [94]

Huperzia_nad9_30298_30655 .....GG........................................... [92]

Isoetes ..........T.....T.......G..GAG...G................ [91]

Zea_perennis .T..G..C..A..ATGA...G...GG....T.GG...AG..CT....... [100]

Arabidopsis .T..G.C..AA..ATGG...G...GG....T.GG...AG..CTG...... [100]

Cycas .T..G..C..A..ATGG.G.G...GG....T......AG..C........ [100]

Megaceros .......C......T.A.......G.....T......A............ [97]

Phaeoceros .......C..T...T.A.......G.....T......A............ [97]

Anomodon .T.....C......T..G..A.........T......A....C....... [97]

Physcomitrella .T.....C......T.....A.........T......A............ [97]

Treubia .T.....C..A...................T......A............ [97]

Marchantia .T.....C..A...................T......A............ [97]

[ 110 120 130 140 150]

[ . . . . .]

Huperzia_nad9 ATCCAGAC----TACCCATTCCAATTATTATGGTTTTTGAAATATCATAC [140]

Huperzia_nad9_30298_30655 ....G..GGGAG...................................... [142]

Isoetes ....G...----.............C..C.C..CC..C............ [137]

Zea_perennis ..A.T...----........T.C...G..G..C...C.A....GG..... [146]

Arabidopsis ..A.G...----....T...T.....G..G..C...C.C....TG..... [146]

Cycas ..A.G...----........T.....G.........CC.....C...... [146]

Megaceros .......T----....TG..TG.....C.......CCC....C....... [143]

Phaeoceros .......T----....TG..TG.....C........C.....C......T [143]

Anomodon ....T..T----....T...T............................. [143]

Physcomitrella ....T..T----....T...T............................. [143]

Treubia .C..GA..A----GT.T...T..........AT................. [143]

Marchantia .C..GA..A----GT.T...T..........AT................. [143]

[ 160 170 180 190 200]

[ . . . . .]

Huperzia_nad9 CAATACACGTTTTCAAGTTTCGATCGAGATTTGCGGAGTTGATTATCCTT [190]

Huperzia_nad9_30298_30655 ....................T..............A..........T... [192]

Isoetes ...........................T...C.................. [187]

Zea_perennis .T.....A.GG................T...........G...C....C. [196]

Arabidopsis .T.....A.GG................T...............C....C. [196]

Cycas .T.........C...............T...C................C. [196]

Megaceros ....................T...T..T...C................C. [193]

Phaeoceros ........................T..T...C..............T.C. [193]

Anomodon ...............G..C.TA.....T.....T..............C. [193]

Physcomitrella ..................C.T...T..T.....T................ [193]

Treubia ......G.............T...T..T.....T..............C. [193]

Marchantia ......G......A......TA..T..T.....T........C....... [193]

[ 210 220 230 240 250]

[ . . . . .]

Huperzia_nad9 CCCGGAAACAAAGATTTGAAGTAGTTTATAATTCACTTAGTGTTGACTAT [240]

Huperzia_nad9_30298_30655 .T...........GG.....C..........A.T................ [242]

Isoetes .T..A...G.......C.....G.............C...........G. [237]

Zea_perennis .T..A....GC...........T..CC......T...G...AC.CGG... [246]

Arabidopsis .T..A....G............G..C.......T...G...AC.CGG... [246]

Cycas .T..A....GG...........G...C.........CA...AC.CGG... [246]

Megaceros .T..A....G......C.........C.........CG...A..CGG... [243]

Phaeoceros .T..A....G......C.........C..........G...A..CGG... [243]

Anomodon .T..A............................T...A...A..C.A... [243]

Physcomitrella .T..A............................T...A...A..C.A... [243]

Treubia .T..A....G.......................T.......A........ [243]

Marchantia .T..A....G......C................T.......A........ [243]

[ 260 270 280 290 300]

[ . . . . .]

Huperzia_nad9 AATACACGCATACGTATATTAACAAGTTACGATGAAATAACTCCAATTTG [290]

Huperzia_nad9_30298_30655 .................................................. [292]

Isoetes .........G.........C.........T.................... [287]

Zea_perennis ..CT.......T...G..CA.......GCG..C...G....A.G...A.C [296]

Arabidopsis ..CT.......T...G..CA...C...GCA..C...G....A.G...A.C [296]

Cycas ..CC.......T...G..CG...C...GTA..C........A.G...A.C [296]

Megaceros ...T....T..T...G..GA..GC...GTAA................... [293]

Phaeoceros ...T..T.T..T...G..GA..GC...GTAAG.................. [293]

Anomodon ..CT.......T...G..CA...C...GTG..C................. [293]

Physcomitrella ..CT.......T...G..CA...C...GTG..C................. [293]

Treubia ........................G..GTA........C........... [293]

Marchantia ...........................GTA........C........... [293]

[ 310 320 330 340 350]

[ . . . . .]

Huperzia_nad9 TTCGGTAGTTGGTATATTTCCATCCGCCGGCTGGTGGGAGCGAGAAGTAT [340]

Huperzia_nad9_30298_30655 .................................................. [342]

Isoetes ..T......CA.....C.C.T..TA.T....C..C.....T.......T. [337]

Zea_perennis .C.......CA..CC.........A......C.................. [346]

Arabidopsis .C.......AA..C..........A......C................T. [346]

Cycas .C.......AA..CC.........A......C.................. [346]

Megaceros .........C..............A...............T....G.... [343]

Phaeoceros ..T......CAA............A....................G.... [343]

Anomodon .....C...CAC.........G..A.......................T. [343]

Physcomitrella .....C...CA..........G..A.......................T. [343]

Treubia .........CA.............G..............A......ACT. [343]

Marchantia .........CA.............G..............A......ACT. [343]

[ 360 370 380 390 400]

[ . . . . .]

Huperzia_nad9 GGGATATGTTTGGTGTGTTTTTTTCCAATCATCCTGATTTACGTCGTATA [390]

Huperzia_nad9_30298_30655 ................---------------------------------- [358]

Isoetes ......C..CC....C..C.CC.C..........C........C....C. [387]

Zea_perennis .........C......T.C..CCAT.........G........C...... [396]

Arabidopsis ................T.C...CAT.........G...C....C...... [396]

Cycas .........CC.....C.A..CCAT.........G...C....C...... [396]

Megaceros ...............C..A..C...T.............C...C...... [393]

Phaeoceros ...............C..A..C...T.............C...C...... [393]

Anomodon ..........C.....T.A......T........C........C...... [393]

Physcomitrella ................T.A......T........................ [393]

Treubia ..................A...C....................A...... [393]

Marchantia ..................A......T.................A...... [393]

[ 410 420 430 440 450]

[ . . . . .]

Huperzia_nad9 TTAACAGATTATGGTTTTGAGGGTCATCCATTACGAAAAGACTTTCCTTT [440]

Huperzia_nad9_30298_30655 -------------------------------------------------- [358]

Isoetes .C.......C................C....C.T........CC..T..C [437]

Zea_perennis .C...T...........C..............................C. [446]

Arabidopsis .C...T...........C........................C.....C. [446]

Cycas .................C..........................C...C. [446]

Megaceros .C..T...........C...........TG...T........C....... [443]

Phaeoceros .C..T........................G...T........C..T.... [443]

Anomodon .................................................. [443]

Physcomitrella .................................................. [443]

Treubia .................................A................ [443]

Marchantia .......................G.........A................ [443]

[ 460 470 480 490 500]

[ . . . . .]

Huperzia_nad9 GAGTGGATATGTGGAAGTACGTTATGATGATTCAGAGAAACGTGTGGTTT [490]

Huperzia_nad9_30298_30655 -------------------------------------------------- [358]

Isoetes A..............................C.................. [487]

Zea_perennis .....................C.........C.................. [496]

Arabidopsis ............CC.......C.........C.................. [496]

Cycas .....................C......................C..... [496]

Megaceros A........CT.C..G.....C...........G..A.....C....... [493]

Phaeoceros A.........T.C..G.................G................ [493]

Anomodon A................................................. [493]

Physcomitrella A................................................. [493]

Treubia A...........A........G...........G................ [493]

Marchantia A....................G............................ [493]

[ 510 520 530 540 550]

[ . . . . .]

Huperzia_nad9 CTGAACCAATTGAGATGACTCAAGAATTTCGCTATTTTGATTTCGCTAGT [540]

Huperzia_nad9_30298_30655 -------------------------------------------------- [358]

Isoetes .......G.......C....T.G....C........C......G..C... [537]

Zea_perennis .......C...........C.......................T...... [546]

Arabidopsis .......C...........C.................C.....T...... [546]

Cycas ............G......C.................C.....T..C... [546]

Megaceros .......T...................C...............T..C... [543]

Phaeoceros .......T...................C.T.............T..C... [543]

Anomodon .......T...................................T...... [543]

Physcomitrella .......T...................................T...... [543]

Treubia .......T.............................C.....T..A... [543]

Marchantia .......T.......................A...........T..A... [543]

[ 560 570 580 590 ]

[ . . . . ]

Huperzia_nad9 CCTTGGGAACAA------------------------------------ [552]

Huperzia_nad9_30298_30655 ------------------------------------------------ [358]

Isoetes ...C.......GATGTCGTAA--------------------------- [558]

Zea_perennis ...........------GCGTAGCGACGGAT-----AA---------- [573]

Arabidopsis ...........------GCGTAGCGACGGAT-----AA---------- [573]

Cycas ............ATGGCGCGTAGCGACGGATCGGATAATGAAGAATAG [594]

Megaceros .........-----GTCGCGTCGTTACGAATCAAATGA---------- [576]

Phaeoceros .........-----ATCGCGTCGTTACGAATCAAATGA---------- [576]

Anomodon ..C.........AGTTCGCGTAGTGACAAATCGAGGAAAAAGTAA--- [588]

Physcomitrella ............AGTTCGCGTAGTGACAAATCGAGTAAAAAGTAA--- [588]

Treubia ............ATGTCGCGTAGTGACGAATCAAATCAAAAGTAA--- [588]

Marchantia ............ATGTCGCGTAGTGACGAATCAAATCAAAAGTAA--- [588]

;

END;

**rpl2**

#NEXUS

[MacClade 4.05 registered to Yin-Long Qiu Lab, EEB, University of Michigan]

BEGIN DATA;

DIMENSIONS NTAX=11 NCHAR=2205;

FORMAT DATATYPE=DNA MISSING=? GAP=- MATCHCHAR=. INTERLEAVE ;

MATRIX

[ 10 20 30 40 50]

[ . . . . .]

Huperzia_rpl2 ATGATAAATAGTGGCTG---GAAGGGAAAA--GCACTGAAACAATCAACT [45]

Huperzia_rpl2_79548_79488 .....G....A......---.....T....AA.....AG...G..T..A. [47]

Vitis_rpl2 ....G.C.A..CCA------AG....G.G.--.....T.G.....TC... [42]

Carica ....G.C.A..CCA------AG....G.G.--.....T.G.....TC... [42]

Brassica_napus ....G.CCAG.--.------AG..CA-.G.--.....T.G.....TC... [39]

Oryza_sativa_Japonica ....G.C.A..CA------TA.....G.G.--..G..T.G...T.TC... [42]

Cycas ....G...G...T..CTACTATTTTCT...--.....T.G.....TC... [48]

Physcomitrella ....-----------------------------....A.......T.... [21]

Anomodon ....-----------------------------....A.......T.... [21]

Treubia ....G.......T....---..........--.....A.......T.... [45]

Marchantia ....G.......T....---..........--.....A.......T.... [45]

[ 60 70 80 90 100]

[ . . . . .]

Huperzia_rpl2 TTTAGTTTTAAAAAGAGGTCTGCCGGGAGAAATTCATCAGGACGTATTAC [95]

Huperzia_rpl2_79548_79488 ....--C..G....A.---------------------------------- [61]

Vitis_rpl2 ..G...---.C.GG..A......T..T..G.....C.....G........ [89]

Carica ..G...---.C.GGA.A......T..T..G.....C.....G........ [89]

Brassica_napus ..G...---.C.GGA.A......T..T..G.....C.....G........ [86]

Oryza_sativa_Japonica ..G...---.CGGG..A...C..A.....G.....T..C..G........ [89]

Cycas ...G..---.C.G...A...C..T.........C....G..G....C... [95]

Physcomitrella ...C....A.....A.A......T..C..............G........ [71]

Anomodon ...C....A.....A.A......T..C..............G........ [71]

Treubia ...CA...A...CG...T.....T..A....................... [95]

Marchantia ...CA...A...CG..AT.....T..A..............G........ [95]

[ 110 120 130 140 150]

[ . . . . .]

Huperzia_rpl2 GGTTTTTCATCGAGGAGGTGGATCGAAGCGATTGCAGCGAGAAATTGATT [145]

Huperzia_rpl2_79548_79488 -------------------------------------------------- [61]

Vitis_rpl2 .........C.....G........................AG.......C [139]

Carica .........C.....G........................AG.......C [139]

Brassica_napus .........C.....G.....C.............T....AG.......C [136]

Oryza_sativa_Japonica T........C.....G........................A.......CC [139]

Cycas .........C.....G........................AG........ [145]

Physcomitrella C.......................A...........T...A......... [121]

Anomodon T...................................T..GA....G.... [121]

Treubia .........C..............A..............CA......... [145]

Marchantia .........C..............................A......... [145]

[ 160 170 180 190 200]

[ . . . . .]

Huperzia_rpl2 TTAAACGAAACACTTCGTCTATGGGCATTGTAGAAAGAATCGAATATGAC [195]

Huperzia_rpl2_79548_79488 -------------------------------------------------- [61]

Vitis_rpl2 .G.......G.........................T.G..A......... [189]

Carica .G.......G...........................G..A......... [189]

Brassica_napus .G.......G......C.................G..T..A......... [186]

Oryza_sativa_Japonica .........G............T........G.....G............ [189]

Cycas CG.......G...........................G............ [195]

Physcomitrella ..C....G..............T...C.....C..........C.....T [171]

Anomodon ..C....G.G............T...C.....C..........C.....T [171]

Treubia .....A...G........................................ [195]

Marchantia .........G........................................ [195]

[ 210 220 230 240 250]

[ . . . . .]

Huperzia_rpl2 CCAAATCGTTCTTCCTGGATTGCTTTAGTACGTTGGATCGAAGGGGTGCT [245]

Huperzia_rpl2_79548_79488 -------------------------------------------------- [61]

Vitis_rpl2 ..T...........TC....C...CC......A........G.......A [239]

Carica ..T...........TC....C...CC......A........G.......A [239]

Brassica_napus ..T...........TCA...C...CC......A......A.G....---- [232]

Oryza_sativa_Japonica ..T...........TC....C...C.......A................. [239]

Cycas ...........C..TC........C.......A................. [245]

Physcomitrella ..T...........T.................A...C.T....CAA..AA [221]

Anomodon ..T........C..T.................A...C...G..CAA..GG [221]

Treubia ...........G..T.................A..............T.. [245]

Marchantia ...........G..T.................A..............T.. [245]

[ 260 270 280 290 300]

[ . . . . .]

Huperzia_rpl2 ACGCCCCGGAAAACGCTTTGCGCTTCATAATAAAGCGAAAAATCGAGGAG [295]

Huperzia_rpl2_79548_79488 -------------------------------------------------- [61]

Vitis_rpl2 G.TA.--------------------------GCC..C.------.....A [257]

Carica G.TA.--------------------------GCC..C.------.....A [257]

Brassica_napus -------------------------------GCT..C.------..AA.A [245]

Oryza_sativa_Japonica G.C.G--------------------------GCC..C.------.....A [257]

Cycas G.TA.--------------------------GCC..C.------...... [263]

Physcomitrella ..AA..AA---------------------.GGC...C..T.G..A.AC.. [250]

Anomodon ..AAG.G.---------------------.GGC...C..T.G..A.AC.. [250]

Treubia ..--------------------------------..C----------CT. [253]

Marchantia ..--------------------------------..C----------CC. [253]

[ 310 320 330 340 350]

[ . . . . .]

Huperzia_rpl2 A-------GGAAAACGCCAAGAAGCTTTTTATTGTGGGTCGAATCAATGA [338]

Huperzia_rpl2_79548_79488 -------------------------------------------------- [61]

Vitis_rpl2 .------T.C..C...AT.GAGGA-------------------------- [275]

Carica .------TT...G...AT.GAGGA-------------------------- [275]

Brassica_napus .------AT...C...ATCGAGGA-------------------------- [263]

Oryza_sativa_Japonica .------TTC..G...AT.GA.GA-------------------------- [275]

Cycas GACTTGCT.C..C...AG.GA.GA-------------------------- [287]

Physcomitrella .-------A.....A......----------------------------- [264]

Anomodon .-------A............----------------------------- [264]

Treubia ---------....G.A.TTG.----------------------------- [265]

Marchantia ---------....G...TTG.----------------------------- [265]

[ 360 370 380 390 400]

[ . . . . .]

Huperzia_rpl2 AAACTTTCGTTTCCTTGGTCGAAAATATGCTCCACTATCTACACGAAGAA [388]

Huperzia_rpl2_79548_79488 -------------------------------------------------- [61]

Vitis_rpl2 --------...CG..----------------------------------- [282]

Carica --------...CG..----------------------------------- [282]

Brassica_napus --------...AG..----------------------------------- [270]

Oryza_sativa_Japonica --------...CG..----------------------------------- [282]

Cycas --------...CG..----------------------------------- [294]

Physcomitrella -------.....T..----------------------------------- [272]

Anomodon -------.....T..----------------------------------- [272]

Treubia -------.T...T..AAAG....--------------------------- [281]

Marchantia -------.T...T..AAAG....--------------------------- [281]

[ 410 420 430 440 450]

[ . . . . .]

Huperzia_rpl2 ACACCGTAGACCCTATGCTCTCTATTGACGTAGTCGCGGGCTCAGAGATG [438]

Huperzia_rpl2_79548_79488 -------------------------------------------------- [61]

Vitis_rpl2 ------------------------------CC.C....------------ [290]

Carica ------------------------------CC.C....------------ [290]

Brassica_napus ------------------------------CC.C....------------ [278]

Oryza_sativa_Japonica ------------------------------CT.C....------------ [290]

Cycas ------------------------------CC.C....------------ [302]

Physcomitrella ------------------------------.G....-------------- [278]

Anomodon ------------------------------.G....-------------- [278]

Treubia ------------------------------......-------------- [287]

Marchantia ------------------------------......-------------- [287]

[ 460 470 480 490 500]

[ . . . . .]

Huperzia_rpl2 CGCCGCTCCTTACCCACTTTCTTTGCATCGCTCCTTGGCCAGAGGACTTG [488]

Huperzia_rpl2_79548_79488 -------------------------------------------------- [61]

Vitis_rpl2 -------------------------------------------------- [290]

Carica -------------------------------------------------- [290]

Brassica_napus -------------------------------------------------- [278]

Oryza_sativa_Japonica -------------------------------------------------- [290]

Cycas -------------------------------------------------- [302]

Physcomitrella -------------------------------------------------- [278]

Anomodon -------------------------------------------------- [278]

Treubia -------------------------------------------------- [287]

Marchantia -------------------------------------------------- [287]

[ 510 520 530 540 550]

[ . . . . .]

Huperzia_rpl2 CAACACGAGAGAAGAAAACGTGTTCGCTCCGCGTAATCTCTTCGAACCTA [538]

Huperzia_rpl2_79548_79488 -------------------------------------------------- [61]

Vitis_rpl2 -----TA...TCCTCG...C.ACCA------------------------- [310]

Carica -----.A...TCCTCG..TC.ACCA------------------------- [310]

Brassica_napus -----.A...TCCTCG...C.ACCA------------------------- [298]

Oryza_sativa_Japonica -----TA...TCCTCG..TCCACCA------------------------- [310]

Cycas -----T....TCCTCG...C.AC.A------------------------- [322]

Physcomitrella ------A......C....TC.T..T-------------.T..-------- [301]

Anomodon ------.......A....TC.T..T-------------.T..-------- [301]

Treubia ------A......A....AA.....-------------.T..-------- [310]

Marchantia ------A......A....TA.....-------------.T..-------- [310]

[ 560 570 580 590 600]

[ . . . . .]

Huperzia_rpl2 CGGCCATCATTGGCCTCTTATTTTCGTTCTCTTCTCTGCCCAGAAAAGC- [587]

Huperzia_rpl2_79548_79488 -------------------------------------------------- [61]

Vitis_rpl2 ..A...C...CC..GG.C................C......G.G..G.TG [360]

Carica ..A...C...CC..GG.C................C......G.G..G.TG [360]

Brassica_napus ..AA..C...CA..GG.C.C.............TC......G.G..G.TG [348]

Oryza_sativa_Japonica ......CT..CTTTTG.C.T..............C...------------ [348]

Cycas ......C...C...GG.C....C........C..C......G.G....TG [372]

Physcomitrella ---------...................T.....C...T....G.....C [342]

Anomodon ---------.C................CT..C.TCT..TT...G.....C [342]

Treubia ---------.C.......................G.....T...C....T [351]

Marchantia ---------.C.......................G.....T...C....T [351]

[ 610 620 630 640 650]

[ . . . . .]

Huperzia_rpl2 ----GAAGAGC---------------------------------TT-CGC [599]

Huperzia_rpl2_79548_79488 -------------------------------------------------- [61]

Vitis_rpl2 GATCA....AAGGTAGCTTGCTTCTCTCCTGGACCGATGGCGGC..AT.T [410]

Carica GATCA....AAGGTAGCTTGCTTCTCTCCTGGACTGATGGCGGC..AT.T [410]

Brassica_napus GATCA....AAGGTAGCTTGCTTCTCTCCTGGACTGATGGCCGC..AT.T [398]

Oryza_sativa_Japonica -------------------------------------------------- [348]

Cycas GATGAG.------------------------------------------- [379]

Physcomitrella CAGA.....AA---------------------------------..A..T [359]

Anomodon CAGA.....AA---------------------------------..A..T [359]

Treubia CGGG..G..AA---------------------------------A.A..A [368]

Marchantia CAAA...T.AA---------------------------------A.A..A [368]

[ 660 670 680 690 700]

[ . . . . .]

Huperzia_rpl2 TC------------------------------------------------ [601]

Huperzia_rpl2_79548_79488 -------------------------------------------------- [61]

Vitis_rpl2 AGTGGTCGGCCTTCCTACCAGAATGCCTCCTTGGTCG------AAGAGCC [454]

Carica AGTGGTCGGCCTTCCTACCAGAATGCCTCTTTGGTCG------AAGAGCG [454]

Brassica_napus AGTGGTCGGCCTTCCTACCGGAATGCCTCCTTTGTCTTCGTATAAGAGCG [448]

Oryza_sativa_Japonica -------------------------------------------------- [348]

Cycas -------------------------------------------------- [379]

Physcomitrella -------------------------------------------------- [359]

Anomodon -------------------------------------------------- [359]

Treubia AA------------------------------------------------ [370]

Marchantia AA------------------------------------------------ [370]

[ 710 720 730 740 750]

[ . . . . .]

Huperzia_rpl2 ------------------------------------CTTCGGG------- [608]

Huperzia_rpl2_79548_79488 -------------------------------------------------- [61]

Vitis_rpl2 CCTTTACTAGTAAGGGCGCAGGAAGCAAAAAAACTTGCG..AAGGACGT- [503]

Carica CCTTTACTAGTAAGGGCGCAGGAAGCAAAAAAACTTGCG..AAGGACGT- [503]

Brassica_napus CCTTTGCTAGTAAGGGCGCAGGAAGCACAAAAACTTTAGT.AAGGACGT- [497]

Oryza_sativa_Japonica -------------------------------------------------- [348]

Cycas ---------------------------------------TAAAGGACGT- [389]

Physcomitrella -------------------------------------------------- [359]

Anomodon -------------------------------------------------- [359]

Treubia ------------------------------------AAAAAA.GCCCGTA [384]

Marchantia ------------------------------------AAA..A.GGCC--- [381]

[ 760 770 780 790 800]

[ . . . . .]

Huperzia_rpl2 TTTCTTCTCTGC---------CT------TTTCTTA--TGCAGAGGTA-G [640]

Huperzia_rpl2_79548_79488 -------------------------------------------------- [61]

Vitis_rpl2 ............T--------T.------C.C..---C.C..A...C-CA [535]

Carica C...........C--------T.------C.C..---C.C..A...C-CA [535]

Brassica_napus C...........C--------T.------C.C..---C.C..A...C-CA [529]

Oryza_sativa_Japonica ------------------------------.C..---C.C.CTT..C-CC [364]

Cycas .C....A.....C--------.C------C.C..---C.C..AG..C-CA [421]

Physcomitrella ...T........C--------..------...T.---CCCT.A...C.-A [391]

Anomodon ...T........C--------..------.....---C.C.....AC.-A [391]

Treubia C.A.G.AC....GGGCAGAGA..TAAAAG..C..GGGTCCT..G.AC.CA [434]

Marchantia C.C.GG.C....GAGCAGATA..GGAAAG..C..GGGTCTT..G.AC.C. [431]

[ 810 820 830 840 850]

[ . . . . .]

Huperzia_rpl2 AAA-----GAG---GGGCTGCGACCTTCGGCCGCCCTTTTGGTAGCTATT [682]

Huperzia_rpl2_79548_79488 -------------------------------------------------- [61]

Vitis_rpl2 .GG-----.G.---A.A..T.A---.C---..TTT.C..C.......C.- [570]

Carica .GG-----...---A.A....A---.C---..TTT.C..C.C.....C.- [570]

Brassica_napus .G.-----...---A.A....A---.C---..TTG.C..C.C.....C.- [564]

Oryza_sativa_Japonica .GG-----...---A.A....A---.C---..TTT.C..C..C....C.- [399]

Cycas .G.-----...---A......A..T.C---..CTT.C.CC.......C.- [459]

Physcomitrella .G.-----...---A......A.TT..A....---....C.......T.- [429]

Anomodon .G.-----...---A......A.TT..A..G.---....C.......T.- [429]

Treubia .G.CCTTA.G.CCAAA.AAAT.T....A..A.---....A.......T.- [480]

Marchantia .G.CCTTA...CCAAA.AA.T.T....A..A.---....A.......T.- [477]

[ 860 870 880 890 900]

[ . . . . .]

Huperzia_rpl2 ATCTTGGGTTACCCAGGATAGCGGTAGCTGGGGCAAAGCCCGCTTTCTTC [732]

Huperzia_rpl2_79548_79488 -------------------------------------------------- [61]

Vitis_rpl2 --T....T..C..A.................................... [618]

Carica --T...AT..C..A.................................... [618]

Brassica_napus --T....T..C..A...................TTCCTA........... [612]

Oryza_sativa_Japonica --T.A..T..C..A.................................... [447]

Cycas --T.G..T.....A................................A... [507]

Physcomitrella --....AT.....T........TT....C............A.......T [477]

Anomodon --..C.AT.....T........CTC...C...T................T [477]

Treubia --.....T........C.............................T... [528]

Marchantia --..............C.....A..T........................ [525]

[ 910 920 930 940 950]

[ . . . . .]

Huperzia_rpl2 GCTTTGCGAATGAAAGGGGAAGAGAAACATGTTTCATTCTCTCCCGAATC [782]

Huperzia_rpl2_79548_79488 -------------------------------------------------- [61]

Vitis_rpl2 ...CC........G..A.-------------------------------- [636]

Carica ...CC...........A.-------------------------------- [636]

Brassica_napus ...CC........G.CA.-------------------------------- [630]

Oryza_sativa_Japonica ..GGA........G..A.-------------------------------- [465]

Cycas ...CC........G..AA-------------------------------- [525]

Physcomitrella ....CC..........A--------------------------------- [494]

Anomodon ....C..........AA--------------------------------- [494]

Treubia .....T...........C---------------------C..T..TCCCT [557]

Marchantia .....T............---------------------C..T..TCCCT [554]

[ 960 970 980 990 1000]

[ . . . . .]

Huperzia_rpl2 AACAAAAGCATGGCGTTTTTTAAAGCTCAGAGAAGAGAATACATTTTCTC [832]

Huperzia_rpl2_79548_79488 -------------------------------------------------- [61]

Vitis_rpl2 -----..A.-----------------......G.A.A..C..G..C.... [664]

Carica -----..AG-----------------......G.A.A..C..G..C.... [664]

Brassica_napus -----..AG-----------------.G....G.A.A.GC..G..C.... [658]

Oryza_sativa_Japonica -----...A-----------------AA.TC.G.A.A..G..G....... [493]

Cycas -----G.A.-----------------......G.A.A..G..G..C.... [553]

Physcomitrella ------------------------CT..G.G...C.T.....G......A [520]

Anomodon ------------------------CT.TG...G.C.TG....G......G [520]

Treubia ...GGG.AG.GA...CC.G.CGGCC...G..A....A.....G..C..G. [607]

Marchantia ...GGG.AG.GA...CC.A.CGCCC.......G...A.....G..C.... [604]

[ 1010 1020 1030 1040 1050]

[ . . . . .]

Huperzia_rpl2 AAAGCGAAGGCCGAAGGTGGAGGAGAACGCATAGCGTTCTCTGGGCGCAT [882]

Huperzia_rpl2_79548_79488 -------------------------------------------------- [61]

Vitis_rpl2 TTT....G.T.....A......---..........A..........A... [711]

Carica TTT....G.T.....A......---..........A..........A... [711]

Brassica_napus TTT....G.T.....A......---..........A..........A... [705]

Oryza_sativa_Japonica TTT....GAT.....A......---.......T.............A... [540]

Cycas TCT....G.T............---......................... [600]

Physcomitrella G...T......G...A.....A---...........------...T...A [561]

Anomodon ...AT..G...G....A....A---G..........------...T...A [561]

Treubia ........A...C.........---...........------......CG [648]

Marchantia ............A........A---......G....------......CG [645]

[ 1060 1070 1080 1090 1100]

[ . . . . .]

Huperzia_rpl2 AAAATCAAACGTAAAGC---GCTTTCCTGGCTTAATGAGAGTTTTTGGCA [929]

Huperzia_rpl2_79548_79488 -------------------------------------------------- [61]

Vitis_rpl2 .GG..............AGC......T....------.....------TT [749]

Carica .GG..T...........AGC......T....------.....------TT [749]

Brassica_napus .GG......G.......AGG......T....------.....------TT [743]

Oryza_sativa_Japonica .GG..............AGC......T....------.....------TT [578]

Cycas .GG..............AGC......T....------.....------T. [638]

Physcomitrella .G....G....C.....---......T...-------...AG------.. [595]

Anomodon .G....G.G..C.....---......T...-------...AC------.. [595]

Treubia .T..GA...A.CCT..T---A..C..T...TCCG.--..GAC------.G [687]

Marchantia .G..GA...A.CCT..T---A..C..T...TCCC.--..G.C------.G [684]

[ 1110 1120 1130 1140 1150]

[ . . . . .]

Huperzia_rpl2 AAAAAGAAAAATCCTTTT----CTTTTTCGAAC----CTGAG-------- [963]

Huperzia_rpl2_79548_79488 -------------------------------------------------- [61]

Vitis_rpl2 T.GGC.GC..GAGAC...AGGG...G..G..G.--TG.....CATAACGA [797]

Carica T.GGC.GC..GAGAC...AGGG...G..G..G.--TG.....CATAACGA [797]

Brassica_napus T.GGC.GC..GATAC...AGGG...G..G..G.--TG...G.CATAACAA [791]

Oryza_sativa_Japonica G.GGCAGC...AAAC...AGAA...G..G..G.--TG.....CATAACGA [626]

Cycas TTGGC.GC..GAGAC...AGGG...GC.GA.G.--TG.....CATAACGG [686]

Physcomitrella .T--------..AT....----T.C...TA...----..A..-------- [621]

Anomodon .T--------..AT....----T.C....A...----.....-------- [621]

Treubia ..GGCCGG....GGC...CCAAT..CCG.AC..GATAT..G.-------G [730]

Marchantia ..GGCC.G....GGC..GATGAT..CCG.AC..GATAT..G.AAGAA-GG [733]

[ 1160 1170 1180 1190 1200]

[ . . . . .]

Huperzia_rpl2 --CATAG--CGAAGAAAGG----------CCGAAGGCAGTT--------- [990]

Huperzia_rpl2_79548_79488 -------------------------------------------------- [61]

Vitis_rpl2 AT.GA..-C......CG.AT--------------CAAG..---------- [822]

Carica AT.GA..-C......CG.AT--------------CAAG..AGC------- [825]

Brassica_napus AT.GA..-C......CG.AT--------------CAAG..AGC------- [819]

Oryza_sativa_Japonica AT.GA..-CT....GCG.AT--------------CAAG..AGCTTGCTCC [661]

Cycas AT..AG.-C.....GCG.AT--------------CAAG------------ [709]

Physcomitrella --.....--.......G.---------------.C-.GA.G--------- [642]

Anomodon --.....--....A..G.---------------.C-..A.G--------- [642]

Treubia GC.GA..GC..G.A.TG.CTTTCCAATTT..AC.CA.GA.ATTGGGAAGA [780]

Marchantia AC.GA..GC..G.A.TG.CTGG------T...C.CA.GA.A--------- [768]

[ 1210 1220 1230 1240 1250]

[ . . . . .]

Huperzia_rpl2 -------------------------------------------------- [990]

Huperzia_rpl2_79548_79488 -------------------------------------------------- [61]

Vitis_rpl2 -------------------------------------------------- [822]

Carica -------------------------------------------------- [825]

Brassica_napus -------------------------------------------------- [819]

Oryza_sativa_Japonica CAAGGCAAGTGCTTGCTTACGCTTTATGTAGTGGTCGGCCTTCCTACCTT [711]

Cycas -------------------------------------------------- [709]

Physcomitrella -------------------------------------------------- [642]

Anomodon -------------------------------------------------- [642]

Treubia AGGACACGAGACTCGAGGGAGAGGCACGTAGTGCTCGACCCTTATTTCAT [830]

Marchantia -------------------------------------------------- [768]

[ 1260 1270 1280 1290 1300]

[ . . . . .]

Huperzia_rpl2 -------------------------------------------------- [990]

Huperzia_rpl2_79548_79488 -------------------------------------------------- [61]

Vitis_rpl2 -------------------------------------------------- [822]

Carica -------------------------------------------------- [825]

Brassica_napus -------------------------------------------------- [819]

Oryza_sativa_Japonica CATGCCTCTAGAAGCTTCTACAAAGCTTTGCTTCCGGTAGAAGCTAGTCG [761]

Cycas -------------------------------------------------- [709]

Physcomitrella -------------------------------------------------- [642]

Anomodon -------------------------------------------------- [642]

Treubia AAAG---------------------------------------------- [834]

Marchantia -------------------------------------------------- [768]

[ 1310 1320 1330 1340 1350]

[ . . . . .]

Huperzia_rpl2 -------------CAAAGTACGCAG------------------------- [1002]

Huperzia_rpl2_79548_79488 -------------------------------------------------- [61]

Vitis_rpl2 ----------TTTT.TTCC.A..C.---ATAGGCGAAGGGCCGAAG---- [855]

Carica ----------TTG.CTGCC.A..C.---ATAGGCGAAAGGCCGAAG---- [858]

Brassica_napus ----------TTG.CTGCC.A..C.---ATAGGCGAAAGGGCGAAGCAAC [856]

Oryza_sativa_Japonica CTTCGGTAGCTTG.CCGCC.A..C.CCTATAGGCGAAGGGCCGAAG---- [807]

Cycas -----------------------------------------CGAGG---- [714]

Physcomitrella -------------------------------------------------- [642]

Anomodon -------------------------------------------------- [642]

Treubia ------------C.CG.AC....TCCCCGCGCACTACGTGCC-------- [864]

Marchantia ------------C.CG.AC....TCCTCGCGCACTCCATGCC-------- [798]

[ 1360 1370 1380 1390 1400]

[ . . . . .]

Huperzia_rpl2 -----------------------------GATGAAGC---------GTGC [1014]

Huperzia_rpl2_79548_79488 -------------------------------------------------- [61]

Vitis_rpl2 -----------------------------....G...---------.... [867]

Carica -----------------------------...AG...---------.... [870]

Brassica_napus TCAAAGCTCTCCGGGGTTTGAGGGCGAAG....G...---------.... [897]

Oryza_sativa_Japonica -----------------------------....G...---------..A. [819]

Cycas -----------------------------....G...---------.... [726]

Physcomitrella -----------------------------.T..G...---------.G.. [654]

Anomodon -----------------------------.T...G..---------.G.. [654]

Treubia -----------------------------.TC.GGC.TTCGGGTTC.G.T [885]

Marchantia -----------------------------.TC.GGC.TTCGGGTTC.G.T [819]

[ 1410 1420 1430 1440 1450]

[ . . . . .]

Huperzia_rpl2 AAAGTATATCATCGTGT------ACCTTTC-------------------- [1038]

Huperzia_rpl2_79548_79488 -------------------------------------------------- [61]

Vitis_rpl2 .....C---G......C------....G..-------------------- [888]

Carica .....C---G......C------....G..-------------------- [891]

Brassica_napus .....C---G......C------....G..-------------------- [918]

Oryza_sativa_Japonica .....C---G......C------....G..GGTTCGGGAGCCGAGCCACT [860]

Cycas .....C---G......C------....G..-------------------- [747]

Physcomitrella ......---G......C------.......-------------------- [675]

Anomodon ......---G......C------.......-------------------- [675]

Treubia CG...G---.TA..CACCTCCGAG......-------------------- [912]

Marchantia CG...G---.TA..CACCTCCGAG......-------------------- [846]

[ 1460 1470 1480 1490 1500]

[ . . . . .]

Huperzia_rpl2 --------------------ACTTATATATTAGCTAGTGATCAATTGGAA [1068]

Huperzia_rpl2_79548_79488 -------------------------------------------------- [61]

Vitis_rpl2 --------------------.........A....C...C........... [918]

Carica --------------------.........A....C...C.......A... [921]

Brassica_napus --------------------.........A....C...C.......A... [948]

Oryza_sativa_Japonica GCACAGGGGCTTAGGTCAAC..............C...C........... [910]

Cycas --------------------..........C....G..C......C..G. [777]

Physcomitrella --------------------.......................G..A... [705]

Anomodon --------------------.......................G..A... [705]

Treubia --------------------..............C.....AA........ [942]

Marchantia --------------------..............C.....AA.T...... [876]

[ 1510 1520 1530 1540 1550]

[ . . . . .]

Huperzia_rpl2 GCAGGCAAGACGGTGCTGAATTGTGATTGGTCTAACCCTTTGACCTCATT [1118]

Huperzia_rpl2_79548_79488 -------------------------------------------------- [61]

Vitis_rpl2 ..G.......T....A.......C........C..A....C....----- [963]

Carica ........A.T....A.......C........C..A....C....----- [966]

Brassica_napus ........A.T....A.......C........C..A....C....----- [993]

Oryza_sativa_Japonica ..G..T..T.T....A.A.....C.....C..C..A....CT.------- [953]

Cycas ..........T....A.......C........C..A....C......G.. [827]

Physcomitrella ..T...........AA...................A....C...T..G.. [755]

Anomodon ..T...........AA...................A....C...T..G.. [755]

Treubia .T...T.........A.T....T.C..C.......A.T..C....C.G.. [992]

Marchantia .T......T.....TA......T.C..G.......A....C....CTG.. [926]

[ 1560 1570 1580 1590 1600]

[ . . . . .]

Huperzia_rpl2 ---------CAACTACCATCAACCTGACCATAATTTGAGAGCCCATACAG [1159]

Huperzia_rpl2_79548_79488 -------------------------------------------------- [61]

Vitis_rpl2 -------AG.G...TATTG.G.....C..GG...------.....----- [995]

Carica -------AG.G...TATTG.G...C.C...G...------.A...----- [998]

Brassica_napus -------AG.TC..T.TCG...T.C.C...G...------.A...----- [1025]

Oryza_sativa_Japonica -AA----AG.GG..T.TTG.G.....C...G...------..T......T [992]

Cycas CAACGGCGG.GG..............C..---..------....---..A [865]

Physcomitrella ---------.G..C.A......T..TC..........CT........AC. [796]

Anomodon ---------.G.TC.A...A..T..TC..........CT........AC. [796]

Treubia ---------A...A...........TC...G..------...---..AT. [1024]

Marchantia ---------A...............TC...G..------...---..AT. [958]

[ 1610 1620 1630 1640 1650]

[ . . . . .]

Huperzia_rpl2 ACCTTCGGTTCCAAGACCACTTCGTTCGCACAGCGAATGAGGGCCTAAGG [1209]

Huperzia_rpl2_79548_79488 -------------------------------------------------- [61]

Vitis_rpl2 ---------.A..-------------TA.TA.------------------ [1005]

Carica ---------....-------------TA.TA.------------------ [1008]

Brassica_napus ---------...T.AG---.CCT---TATT..CT.TG...---------- [1050]

Oryza_sativa_Japonica ................---GC.T.GA.......T....A.A..T.----- [1034]

Cycas ................---.C.T.................A....----- [907]

Physcomitrella ..............A.......T....A....A.A.....A....A.... [846]

Anomodon ............G.A.......T....A.CT.A.A.....A....A.... [846]

Treubia ...----------------...T.-----------------...------ [1035]

Marchantia ...----------------.....-----------------...------ [969]

[ 1660 1670 1680 1690 1700]

[ . . . . .]

Huperzia_rpl2 TCCCATCTGGTG---GAGCCCGCGCGGGGCAGTAAGACTGC---TTCCTC [1253]

Huperzia_rpl2_79548_79488 -------------------------------------------------- [61]

Vitis_rpl2 -------------------------------------------------- [1005]

Carica -------------------------------------------------- [1008]

Brassica_napus -------------------------------------------------- [1050]

Oryza_sativa_Japonica -------G...TGAA.G.------G.CA.TCAGCT.G.A..TTC..GGC. [1071]

Cycas -------G....GAA.G..GG.G.G.CA.TCAGC..G....TTC..GGC. [950]

Physcomitrella ....T.AG....GAA......A.....C.T...C..G....TTC.GGGCT [896]

Anomodon ....TCAG....GAA.......T..A.C.T...C..G....TTC..GG.T [896]

Treubia ---.T.GG...TAAA...A......A..AT..CC..G..------.GGC. [1076]

Marchantia ---.T.AG....GAA...A....AT...AT..CC..G..------.GGCT [1010]

[ 1710 1720 1730 1740 1750]

[ . . . . .]

Huperzia_rpl2 ACGCCCTGAGCAAAACTACGCTTATAGTGGTGAGAACAATTACATACTTG [1303]

Huperzia_rpl2_79548_79488 -------------------------------------------------- [61]

Vitis_rpl2 -------------------------------------------------- [1005]

Carica -------------------------------------------------- [1008]

Brassica_napus -------------------------------------------------- [1050]

Oryza_sativa_Japonica ......CC---------CT........AC---------.CG.G...T.G. [1103]

Cycas ......CC..T---..GC.........AT---------GCG.......C. [988]

Physcomitrella ......G.G.GGG..........CA....---....T..AA......... [943]

Anomodon ........G.GGGG.........CA....---....T..AA......... [943]

Treubia G.A...CCGTGGTG.......C.T.....---....T..A.......... [1123]

Marchantia ..A...CCGTGG.G.........C.....---....T..A.......... [1057]

[ 1760 1770 1780 1790 1800]

[ . . . . .]

Huperzia_rpl2 ATTTCTATAATCAAATGGTGGGAAATAGCGTACCATTAGCCAATATACCT [1353]

Huperzia_rpl2_79548_79488 -------------------------------------------------- [61]

Vitis_rpl2 -------------------------------------------------- [1005]

Carica -------------------------------------------------- [1008]

Brassica_napus -------------------------------------------------- [1050]

Oryza_sativa_Japonica ..C.AA..TCAA..---..A.........A..........TG......G. [1150]

Cycas ..CCG...TC....---..A...G..T..A......C...........G. [1035]

Physcomitrella ...CA...T..........A......T.....T....G..T........C [993]

Anomodon ...C....T..........A......T.....T....G..TC........ [993]

Treubia ...CA...T..........C......T..A.............A...... [1173]

Marchantia ...CA...T..........C......T..A.............A...... [1107]

[ 1810 1820 1830 1840 1850]

[ . . . . .]

Huperzia_rpl2 ATAGGCACGTGGGTACATAATATTGAATGGAATCCGGGTCAAGGCGCTAA [1403]

Huperzia_rpl2_79548_79488 -------------------------------------------------- [61]

Vitis_rpl2 -------------------------------------------------- [1005]

Carica -------------------------------------------------- [1008]

Brassica_napus -------------------------------------------------- [1050]

Oryza_sativa_Japonica ..G..A..A.........G..........TC....A...........A.. [1200]

Cycas .C...A..A....C........C......TG....A...........A.. [1085]

Physcomitrella ........A..........................A...........A.. [1043]

Anomodon ........A...A..................................A.. [1043]

Treubia .......................A...A....C..A..G.....T..A.. [1223]

Marchantia ...........................A....C..A...........A.. [1157]

[ 1860 1870 1880 1890 1900]

[ . . . . .]

Huperzia_rpl2 GTTTACTCGAGCTGCAGGAACTTTTGCTAAAATACTTAAGAAACTTGATA [1453]

Huperzia_rpl2_79548_79488 -------------------------------------------------- [61]

Vitis_rpl2 -------------------------------------------------- [1005]

Carica -------------------------------------------------- [1008]

Brassica_napus -------------------------------------------------- [1050]

Oryza_sativa_Japonica .C.GG....G..C..........A..........A.....G.G.CA.--- [1247]

Cycas .CCG.................C......G.....A.G.......C..GG. [1135]

Physcomitrella ...G.T............G..C......C.....A........T.C..A. [1093]

Anomodon ...GGT............G..C.....CC.....A.C......T.C..A. [1093]

Treubia ...G.................C......C.....A....A..GG....G. [1273]

Marchantia ...G.................C......C.....A..C.A...G....G. [1207]

[ 1910 1920 1930 1940 1950]

[ . . . . .]

Huperzia_rpl2 ATACACCACAATGTGTTGTGCAGTTACCATCAGGTGTTGACAAACTCATA [1503]

Huperzia_rpl2_79548_79488 -------------------------------------------------- [61]

Vitis_rpl2 -------------------------------------------------- [1005]

Carica -------------------------------------------------- [1008]

Brassica_napus -------------------------------------------------- [1050]

Oryza_sativa_Japonica ---.C.........C......G.C.......G........A...T..... [1294]

Cycas ..G...........C......G..C...TC.G........A......... [1185]

Physcomitrella ..............A.C....GA.....T..G.................. [1143]

Anomodon ..............A......GA..G..T..G.................. [1143]

Treubia .....T........A......G......C..G..............A... [1323]

Marchantia ..............A......G......T..G..............A... [1257]

[ 1960 1970 1980 1990 2000]

[ . . . . .]

Huperzia_rpl2 GATTCCAGATGCCGAGCTACTATTGGTATAGTGTCTAATCCCAATCATGG [1553]

Huperzia_rpl2_79548_79488 -------------------------------------------------- [61]

Vitis_rpl2 -------------------------------------------------- [1005]

Carica -------------------------------------------------- [1008]

Brassica_napus -------------------------------------------------- [1050]

Oryza_sativa_Japonica ......C.........................T..C........C..... [1344]

Cycas ...C..C..........C..............TC.C........C..... [1235]

Physcomitrella ......C..............G.C...........C....TTC....... [1193]

Anomodon ......C..............G.C...........C....TTC....... [1193]

Treubia ......C.........................T.......TT........ [1373]

Marchantia ......C.........................T.......TT........ [1307]

[ 2010 2020 2030 2040 2050]

[ . . . . .]

Huperzia_rpl2 TAAACGTGAGCTTAACAAAGCAGGACGAAACCGGTGGTTAGGCAGACGCC [1603]

Huperzia_rpl2_79548_79488 -------------------------------------------------- [61]

Vitis_rpl2 -------------------------------------------------- [1005]

Carica -------------------------------------------------- [1008]

Brassica_napus -------------------------------------------------- [1050]

Oryza_sativa_Japonica .GC....A......GA..........A..G........C........... [1394]

Cycas .GC....A.....GGA.G........A..G.................... [1285]

Physcomitrella .......A.....G.......G....A..G...A..............T. [1243]

Anomodon .......A.....G............A..G...A..............T. [1243]

Treubia .......A..................A..G.................... [1423]

Marchantia .......A..T...............A..G.................... [1357]

[ 2060 2070 2080 2090 2100]

[ . . . . .]

Huperzia_rpl2 CCATCGTTCGGGGGGTTGCTATGAATCCAGTTGATCATCCTCATGGAGGA [1653]

Huperzia_rpl2_79548_79488 -------------------------------------------------- [61]

Vitis_rpl2 -------------------------------------------------- [1005]

Carica -------------------------------------------------- [1008]

Brassica_napus -------------------------------------------------- [1050]

Oryza_sativa_Japonica ....T.....T..T.....A...........G.................. [1444]

Cycas ....T.C......T.................G.................. [1335]

Physcomitrella ....T........T..............G..G.................. [1293]

Anomodon ....T........T..............G..G.................. [1293]

Treubia ....T........T.................C..C..............C [1473]

Marchantia ....T........T.......................C...........C [1407]

[ 2110 2120 2130 2140 2150]

[ . . . . .]

Huperzia_rpl2 GGTGAAGGACGCACGAAAGGGGGTAGACCTTCAGTGTCACCTTGGGGAAA [1703]

Huperzia_rpl2_79548_79488 -------------------------------------------------- [61]

Vitis_rpl2 -------------------------------------------------- [1005]

Carica -------------------------------------------------- [1008]

Brassica_napus -------------------------------------------------- [1050]

Oryza_sativa_Japonica .....G..G...........A...........G..............G.. [1494]

Cycas ....................A...........G...............G. [1385]

Physcomitrella ..C.................A...........G..A.............. [1343]

Anomodon ..............A.....A...........G..A.............. [1343]

Treubia ..............T.....A...........G..A...........C.. [1523]

Marchantia ..............T.....A...........G..A...........C.. [1457]

[ 2160 2170 2180 2190 2200]

[ . . . . .]

Huperzia_rpl2 GCCCACCAAAGGTGGATTTAAAACAGTGGT------AAGAAAACGCAAAA [1747]

Huperzia_rpl2_79548_79488 -------------------------------------------------- [61]

Vitis_rpl2 -------------------------------------------------- [1005]

Carica -------------------------------------------------- [1008]

Brassica_napus -------------------------------------------------- [1050]

Oryza_sativa_Japonica ...........CA......CGGG...GA..GGGGGTGG.G.......G.. [1544]

Cycas ...........C.....C.CG......A..------GG.G.......G.. [1429]

Physcomitrella ....G......................A..------...........G.. [1387]

Anomodon ....G...............G......A..------...........G.. [1387]

Treubia ...T.......................A..------...........G.. [1567]

Marchantia ...T.......................A..------...........G.. [1501]

[ ]

[ ]

Huperzia_rpl2 TTTAG [1752]

Huperzia_rpl2_79548_79488 ----- [61]

Vitis_rpl2 ----- [1005]

Carica ----- [1008]

Brassica_napus ----- [1050]

Oryza_sativa_Japonica ..... [1549]

Cycas A.C.. [1434]

Physcomitrella A.... [1392]

Anomodon A.... [1392]

Treubia AC... [1572]

Marchantia A.... [1506]

;

END;

**rps2**

#NEXUS

[MacClade 4.05 registered to Yin-Long Qiu Lab, EEB, University of Michigan]

BEGIN DATA;

DIMENSIONS NTAX=9 NCHAR=883;

FORMAT DATATYPE=DNA MISSING=? GAP=- MATCHCHAR=. INTERLEAVE ;

MATRIX

[ 10 20 30 40 50]

[ . . . . .]

Huperzia_rps2 ATGTACAATTATCATTCCAGTTTGGTTCTTCAAAAATTACTG---AGTAC [47]

Huperzia_rps2_232189_232865 ............T.........C....A....GG........---..... [47]

Isoetes_HQ616414 ..........C......T.........A...CG.G....T..CCG..... [50]

Zea_perennis_rps2_1 ...-.....C---C..T..AC.--..CTG.ACT........T---T.... [41]

Cycas_rps2 ..........---....T........C.....G....C....---..... [44]

Anomodon ..........C.AG..TG---......A...........T.A---..... [44]

Physcomitrella ..........C.AG..TG---......A...........T.A---..... [44]

Treubia_rps2 ..........C.A...TG---......A..............---..... [44]

Marchantia ..........C.A...TG---......A..............---..... [44]

[ 60 70 80 90 100]

[ . . . . .]

Huperzia_rps2 AAACGCATATCTGGGCCATCGGATACCTACTTCCGATTTTCAAGGAT--- [94]

Huperzia_rps2_232189_232865 .C......G.....A......A...........T...C.........GAT [97]

Isoetes_HQ616414 .C..........................-------............--- [90]

Zea_perennis_rps2_1 G..T...C....C....G....G..G..G..CA.C....CA...TC.--- [88]

Cycas_rps2 G......C....A....G....G..G..G..CA.C...CCA...T..--- [91]

Anomodon ...T.................A.........................--- [91]

Physcomitrella ...T.................A.........................--- [91]

Treubia_rps2 ...........................A.....T.............--- [91]

Marchantia ...........................A.....T.............--- [91]

[ 110 120 130 140 150]

[ . . . . .]

Huperzia_rps2 ATTTATACGGATTTAGAAATGAAATGGCTATTATTGATTTAGAAAAAA-C [143]

Huperzia_rps2_232189_232865 .............CG.........GA.---.....A...C........A. [144]

Isoetes_HQ616414 --.C-------...G..G..----------.CT.CA.A..-.......-. [119]

Zea_perennis_rps2_1 ..A.CCGTT.T..C.......G...T......C.C....C...C..G.-. [137]

Cycas_rps2 ..A.......T..CG....C.....T......C.C...CC...C..G.-. [140]

Anomodon ...................................A............-. [140]

Physcomitrella ...................................A............-. [140]

Treubia_rps2 .......T....................................C...-. [140]

Marchantia .......T........................................-. [140]

[ 160 170 180 190 200]

[ . . . . .]

Huperzia_rps2 ACTTATTTGTTT-----ACAAAAGGCTTGTAAATTGGTGGCATCTATCAT [188]

Huperzia_rps2_232189_232865 ............CGTTT..G..----......T.C.A......T...... [190]

Isoetes_HQ616414 ............-----GAG..G.........T...A.A........... [164]

Zea_perennis_rps2_1 ...G........-----..G...C...CT.G.T..T..A.G..A.C.... [182]

Cycas_rps2 ...G.......C-----..G...C......C.T.CTA.A.G....CC... [185]

Anomodon ............-----..G..G.........T...A.T.A......T.. [185]

Physcomitrella ............-----..G..G.........T...A.T.A......T.. [185]

Treubia_rps2 ............-----..G..G.........T...A.T.G......... [185]

Marchantia ............-----..G..G.A.......T...A.T.G......... [185]

[ 210 220 230 240 250]

[ . . . . .]

Huperzia_rps2 TCGTTCAAAAGAAAGCCATTTATTATTGGTAAATATCAATAATC----CG [234]

Huperzia_rps2_232189_232865 ....G......---.G........T.......G..C......ATAATG.A [237]

Isoetes_HQ616414 ...AGGTG...---.......CG.T.........GC....G-C.AATAA. [210]

Zea_perennis_rps2_1 .T..CA....A---...G..CC..C..TT....G.CG..G...T----TA [225]

Cycas_rps2 ....AA...G.---...G..C....CCC.......C...---.T----.. [225]

Anomodon ....G......---.......T.....A..G....C.G..---.----.A [225]

Physcomitrella ....G......---.......T.....A.......C.G..---.----.A [225]

Treubia_rps2 .A..G......---.......G.............C....---.----.. [225]

Marchantia .A..G......---.....................C....---.----.. [225]

[ 260 270 280 290 300]

[ . . . . .]

Huperzia_rps2 GTATATAATAAGATTATTCAACAAACGGCGAAAAG---AACC-AATCAAA [280]

Huperzia_rps2_232189_232865 AA....C..G...................AGG..A---....C....... [284]

Isoetes_HQ616414 ..G....G.......G.....GG......A..G..---G..T-.....G. [256]

Zea_perennis_rps2_1 T.TATAT..TC...A..GGC.A...T.-------------------TGG. [256]

Cycas_rps2 T...TCG..G....A..AG....G.T...ACT.TCCTT..T.-G...G.. [274]

Anomodon .A.........A..AG...G.....T...A.....---....-.....G. [271]

Physcomitrella .A.........A..AG.........T........A---....-.....G. [271]

Treubia_rps2 .A.........A..A..........T...A..C..---....-....... [271]

Marchantia .A.........A..A..........T...A....A---....-....... [271]

[ 310 320 330 340 350]

[ . . . . .]

Huperzia_rps2 GCTATATTAATGATA---AATGGA---TTGGGGGGGTTTTGACCAATTGG [324]

Huperzia_rps2_232189_232865 ..GG...C...C..G---......---.G..A..AAGG..T......-.. [327]

Isoetes_HQ616414 ........G..C..G---......---....C........A..T...... [300]

Zea_perennis_rps2_1 ...G...C......TCTC......AGA.C....CTT............CT [306]

Cycas_rps2 ...G...C......TATC......GGA.C.....TT........G...AT [324]

Anomodon .......C...C...---......---....A..AT...........C.C [315]

Physcomitrella .......C...C...---......---....A..AT...........C.C [315]

Treubia_rps2 .......A...C...---......---.......TT.............. [315]

Marchantia .......C...C...---......---.......TT.............. [315]

[ 360 370 380 390 400]

[ . . . . .]

Huperzia_rps2 GAACATATGGAGGATGTACAGCAACACTTTCAAGATCTCTCTGAGGATCC [374]

Huperzia_rps2_232189_232865 A....G...A.AA.G----.AA........ATTC..T.G....C.A.... [373]

Isoetes_HQ616414 ..G......ATA...T....AA.....-----------------...--- [330]

Zea_perennis_rps2_1 T-----...---------------.---.----------------A.... [317]

Cycas_rps2 TC.AG....---------------..T..----------------AT.T. [343]

Anomodon A........A.AA.......AA..........GA..T.T....C.C..T. [365]

Physcomitrella A........A.AA.......AA..........GA..T......C.C..T. [365]

Treubia_rps2 A........A.A..A.....AA.........G....T......CAC..T. [365]

Marchantia A........A.AA.A...A.AA..............T......CAC.... [365]

[ 410 420 430 440 450]

[ . . . . .]

Huperzia_rps2 CGAATTTAAAGACGCCTTTACATCTTCGCCCTT---TTCTCTTCCACGTT [421]

Huperzia_rps2_232189_232865 ......G...T....A.................TGA....A...T..... [423]

Isoetes_HQ616414 -------G.C........G..T...........C--C..GA..----A.. [367]

Zea_perennis_rps2_1 TA.G---...---------------------..C------------.... [331]

Cycas_rps2 ....---.....------------.C..TTT..CA-----------...C [367]

Anomodon .A...............C..T...G........CGA..T.T....G.A.. [415]

Physcomitrella .A....G..........C......G........CGA..T.T......A.. [415]

Treubia_rps2 TA.T.CG........T..............T..CGA..A.T......... [415]

Marchantia TA.T..G........T..............T..CGA..A.T......... [415]

[ 460 470 480 490 500]

[ . . . . .]

Huperzia_rps2 TTAGAA--------AGATGCAAAATTGTTTT---GAAGGAATCATGACAC [460]

Huperzia_rps2_232189_232865 ..G...GAATGGAA......C...A......---.........G...... [470]

Isoetes_HQ616414 .G....--------.....A.G........G---.G...G.......... [406]

Zea_perennis_rps2_1 CA....--------.C.A.A...TC.A....------------GG.TTGA [361]

Cycas_rps2 CGG...--------.T.A.A.G.TCAA....TTC.G....TC.GG.T.GA [409]

Anomodon .C....--------..........A......---................ [454]

Physcomitrella ......--------..........A......---................ [454]

Treubia_rps2 .C.A..--------.A........A......---................ [454]

Marchantia ...A..--------..........A......---................ [454]

[ 510 520 530 540 550]

[ . . . . .]

Huperzia_rps2 ACTGTATTCCGAATTGTTTAGTAATAATGAATGCAAATTTAAATTCCATG [510]

Huperzia_rps2_232189_232865 ..CA......A...............--..........C.C..AA..... [518]

Isoetes_HQ616414 ..C.......A............G..............C.T.G....... [456]

Zea_perennis_rps2_1 ..C.ACAA..TG.....G.G..T..TC.TC..C..G..AG...G..ATC. [411]

Cycas_rps2 ..CAACGA..AG..C..G.................G..AG.G.G...TC. [459]

Anomodon ..GA......AG................AG........AA......T... [504]

Physcomitrella ..GA......AG................A.........AA......T... [504]

Treubia_rps2 ..AA......AG..........T.....A.........AA......A... [504]

Marchantia ..AA...C..AG................A.........CA.......... [504]

[ 560 570 580 590 600]

[ . . . . .]

Huperzia_rps2 GCTATACTTGAAGCTGATAAATTACAAATACCTATTATATCCTTGGTGGA [560]

Huperzia_rps2_232189_232865 .............T.A..C...G...............----.G...T.. [564]

Isoetes_HQ616414 ..................C......GG.........G.C...CC...... [506]

Zea_perennis_rps2_1 .TC.....G.........CG..C.........G...GC..A...A..... [461]

Cycas_rps2 .TC.......G.......CG................G....T........ [509]

Anomodon ..............CA..C................CG....T........ [554]

Physcomitrella ..............CA..C................CG....T........ [554]

Treubia_rps2 ...............A..C....................G.T.....T.. [554]

Marchantia ...............A..C.................G..G.T........ [554]

[ 610 620 630 640 650]

[ . . . . .]

Huperzia_rps2 TTCTAATATTCCGAACAGATTACATGAATTAATCACTTATCCTATTCCAG [610]

Huperzia_rps2_232189_232865 .......G----A...........C........T......T.C.....G. [610]

Isoetes_HQ616414 ..G.C.....T.....G.........................C....... [556]

Zea_perennis_rps2_1 ...C.CG..C...TC.------...A..AG.T......T.A.C..C.... [505]

Cycas_rps2 ...G.....C..ATTGG.....TG.A..GG...A........C....... [559]

Anomodon ..........T.............AA.......A........C....... [604]

Physcomitrella ..........T.A...........AA.......A........C....... [604]

Treubia_rps2 ...C........A............A.......A.........G...... [604]

Marchantia ............A............A..C....A........CG...... [604]

[ 660 670 680 690 700]

[ . . . . .]

Huperzia_rps2 TGAATGAT---GATTCTATACAGTT---TGTATATCTCTTTTGTAATTTG [654]

Huperzia_rps2_232189_232865 ........---....T..C----..---C....T..CA...C........ [650]

Isoetes_HQ616414 ........TAT..............---C........A..C......... [603]

Zea_perennis_rps2_1 C.......------C..T...G.C.GCTC........A...C......C. [549]

Cycas_rps2 C.......------...........---C.......CA.C.C......C. [600]

Anomodon .A......---..............---.........A............ [648]

Physcomitrella ........---..............---.........A............ [648]

Treubia_rps2 ........---.........A....---.......T.A..C......... [648]

Marchantia ........---.........A....---.......T.A............ [648]

[ 710 720 730 740 750]

[ . . . . .]

Huperzia_rps2 ATTACGGAAACTGT------------------------------------ [668]

Huperzia_rps2_232189_232865 ......AT...A..------------------------------------ [664]

Isoetes_HQ616414 G.G...A....A..------------------------------------ [617]

Zea_perennis_rps2_1 ..C...A.......GAGGCAAAAAAGAACAGCAATCAACGGGCCGAGAGC [599]

Cycas_rps2 ..C...A....A..------------------------------------ [614]

Anomodon ......A....A..------------------------------------ [662]

Physcomitrella ......A....A..------------------------------------ [662]

Treubia_rps2 ...G..A....AA.------------------------------------ [662]

Marchantia .....CA....A..------------------------------------ [662]

[ 760 770 780 790 800]

[ . . . . .]

Huperzia_rps2 -------------------------------------------------- [668]

Huperzia_rps2_232189_232865 -------------------------------------------------- [664]

Isoetes_HQ616414 -------------------------------------------------- [617]

Zea_perennis_rps2_1 GGGAGCACGAGGAGCGAGTACCCTTCTGGGTATTCGCACCTACTCTGCCG [649]

Cycas_rps2 -------------------------------------------------- [614]

Anomodon -------------------------------------------------- [662]

Physcomitrella -------------------------------------------------- [662]

Treubia_rps2 -------------------------------------------------- [662]

Marchantia -------------------------------------------------- [662]

[ 810 820 830 840 850]

[ . . . . .]

Huperzia_rps2 -CATTCTTT---CGCAA--------------------------------- [681]

Huperzia_rps2_232189_232865 -........---.A...--------------------------------- [677]

Isoetes_HQ616414 -.....ACGAGG..GG.TCAGGGCGAAGTAACCTTGAAAGAAGTGATCGC [666]

Zea_perennis_rps2_1 C.TC..C.AAG-T.G..GAAGAAGTTTTTTCGCTTCAAAAATGGGATTGT [698]

Cycas_rps2 -TCC....GAA-..GGGAAAAA-------TCG--------------TTGC [641]

Anomodon -........---.A...--AGTGCTTGGCCGCTCTCGCGAAAACCCTTAG [706]

Physcomitrella -........---.A...--AGTGCTTGGCCGCTCTCGCGAAAACCCTTAA [706]

Treubia_rps2 -....T...---.AA..--AGAGCGCAGAGGC-----CGAAAG---TGAA [698]

Marchantia -........---.AA..--AGATCGCAGAGGC-----CAAAGG---TTAA [698]

[ 860 870 880 ]

[ . . . ]

Huperzia_rps2 --------------------------------- [681]

Huperzia_rps2_232189_232865 --------------------------------- [677]

Isoetes_HQ616414 ATTGACTGGACTGGCTCTTCCATGCATACATAG [699]

Zea_perennis_rps2_1 CATGAATC-GTAAATTTAGTTTGATGCTTCTGC [730]

Cycas_rps2 GATGAA---GTAG-------------------- [651]

Anomodon ATCGGGGCCGCAGGCCCTAG------------- [726]

Physcomitrella GTCGAGGCCGCAGGCCCTAG------------- [726]

Treubia_rps2 AGTGAAA----AGGCTCTGA------------- [714]

Marchantia AGTAAAA----AGGCTTTGA------------- [714]

;

END;

**rps3**

#NEXUS

[MacClade 4.05 registered to Yin-Long Qiu Lab, EEB, University of Michigan]

BEGIN DATA;

DIMENSIONS NTAX=10 NCHAR=1993;

FORMAT DATATYPE=DNA MISSING=? GAP=- MATCHCHAR=. INTERLEAVE ;

MATRIX

[ 10 20 30 40 50]

[ . . . . .]

Huperzia_rps3 ATGGCACAAAAAGTAAATCCGATTTCAGTCAGACTCAATCTTAATCGTAG [50]

Huperzia_rps3_298955_299087 -------------------------------------------------- [0]

Isoetes_HQ616415 ..........................G........T...T.......... [50]

Zea_perennis .......G.....G......A.....G..A.....TG....G........ [50]

Brassica_napus .......G.....G............G..A.....TGGAAAA........ [50]

Cycas .......G.G....C....................T....CG........ [50]

Anomodon .....G......A......................G.....G........ [50]

Physcomitrella .....G......A....................T.G.....G........ [50]

Treubia ..............C..........................G........ [50]

Marchantia .........................................G........ [50]

[ 60 70 80 90 100]

[ . . . . .]

Huperzia_rps3 CTCAGATTCAAGTTGGTTTAGTGATTATTATTATGGAAAATTGTTGTA-- [98]

Huperzia_rps3_298955_299087 -------------------------........C.......C..G...AC [25]

Isoetes_HQ616415 T.......T.G.....................T...............-- [98]

Zea_perennis T......C.....C....C.................T....CA.....-- [98]

Brassica_napus T............C....C.....G...........T.....TG....-- [98]

Cycas T......C.....C...CC............C....G......G....-- [98]

Anomodon T...............................................-- [98]

Physcomitrella T...............................................-- [98]

Treubia T........C......................................-- [98]

Marchantia T........C......................................-- [98]

[ 110 120 130 140 150]

[ . . . . .]

Huperzia_rps3 ---TCAAGATGTAAATTTTAGAGATTATTCTGCTTC------AATACGTC [139]

Huperzia_rps3_298955_299087 CAC............A..G.T.A.......CAG...CGTAAT........ [75]

Isoetes_HQ616415 ---.......T.T...C.G..........T..G...------G....... [139]

Zea_perennis ---.........C...C.....TC.....TCAG...------G....... [139]

Brassica_napus ---.........C...C.G...TC.....TC.G...------G....... [139]

Cycas ---.........C...C............TC.G...------........ [139]

Anomodon ---........................C.T..A..T------........ [139]

Physcomitrella ---........................C.T..A..T------........ [139]

Treubia ---.......T..................TC.G...------........ [139]

Marchantia ---.......T..................T..G...------........ [139]

[ 160 170 180 190 200]

[ . . . . .]

Huperzia_rps3 CACCTAGGCGAAACAAGTTTGGCTTCCGTCTCGGTAGATGTATTATTCAT [189]

Huperzia_rps3_298955_299087 ....C.C.G..C.A.C.........T.....T.T................ [125]

Isoetes_HQ616415 ....A.....G............C.T.....T.....G............ [189]

Zea_perennis ......C.A..CT..CC........T...........G..........TA [189]

Brassica_napus ......C.A..CT..CC........T...........G......C...TA [189]

Cycas ......C.A....A.CC........T...........G............ [189]

Anomodon ......C.G....A.C.........T..........AG.T.......... [189]

Physcomitrella ......C.G....A.C.........T..........AG.T.......... [189]

Treubia ......C.G......C.........T...........G............ [189]

Marchantia ......C.G......C.........T...........G............ [189]

[ 210 220 230 240 250]

[ . . . . .]

Huperzia_rps3 CATTCTCCTAAAAGGACATTTATTCATGTATTTTATCTGGGTCGACA--- [236]

Huperzia_rps3_298955_299087 ....TC.T------------------------------------------ [133]

Isoetes_HQ616415 ....T.A.............C......T...A.CT.....A....A.--- [236]

Zea_perennis ....T...C...........C......T.C...CT..CCC......CACT [239]

Brassica_napus ....T.........A.....C......T.C...CT..CCC......CACG [239]

Cycas ....T.A.C...........C......T...C.CT..CCC......CACG [239]

Anomodon ....T...C...........C.....C..C....T.T...A.....---- [235]

Physcomitrella ....T...............C.....C.......T.....A.....---- [235]

Treubia ....T...............C.............T.....A.....---- [235]

Marchantia ....T...............C.............T.....A.....---- [235]

[ 260 270 280 290 300]

[ . . . . .]

Huperzia_rps3 ----CTACCGCCAATCAGGACCTACGGGCCTTA----------------- [265]

Huperzia_rps3_298955_299087 -------------------------------------------------- [133]

Isoetes_HQ616415 ----....T..G.....A..-ACCA....T..G----------------- [264]

Zea_perennis ACGA...AAA.G.CG.GAC.AA.CAA.A..CGGAAAGGATAAGGGCCGGT [289]

Brassica_napus ACGA..GAAA.G.CGTGAA.AAACTA.A..CGGAAAGGAGAAGGGCCGGT [289]

Cycas ACGA..GAAA.G.CG.G.C.AA.CAA....CGGAAAGG------GCCGGT [283]

Anomodon ----T..--...G...GA...AC..A......G----------------- [262]

Physcomitrella ----T..--...GG...A...AC..A......G----------------- [262]

Treubia ----T..--........A...A.CAA......G----------------- [262]

Marchantia ----T..--........A...A.CAA......G----------------- [262]

[ 310 320 330 340 350]

[ . . . . .]

Huperzia_rps3 ---GGGC---AAAAAAATAT---AAGTTGACCAGGCGTATTGA------C [300]

Huperzia_rps3_298955_299087 -------------------------------------------------- [133]

Isoetes_HQ616415 ---..C.---TCCTC.G..G--------.GT..A---.G.CCG------T [291]

Zea_perennis GGT....---.TTTGGGA.AGTCGG.CC..T.G..T..C..C.TTCAAG. [336]

Brassica_napus GGT..A.GAC.TTCGGGA.AGCCGG.CC..T.G..T..C..C------G. [333]
[truncated: 189,463 more chars]
