# Supplementary material for: The Mitochondrial Genome of the Lycophyte Huperzia squarrosa: The Most Archaic Form in Vascular Plants
Source: PLoS One. 2012 Apr 12;7(4):e35168. doi: 10.1371/journal.pone.0035168 (PMC3325193; doi:10.1371/journal.pone.0035168)
Supplement: Figure S3 — Alignment of introns that are attached to pseudogene piece(s) or located in the functional gene in the Huperzia mitochondrial genome and the ortholog intron from other plants. Most of these plants have their mitochondrial genomes sequenced, which are available at NCBI Organelle Genome Resources (http://www.ncbi.nlm.nih.gov/genomes/GenomesHome.cgi?taxid=2759&hopt=html). Coordinate numbers indicating location of a pseudogene intron piece within the Huperzia mitochondrial genome are listed in the sequence name. If desired, each matrix can be copied in “word” to make a “.txt” file and opened in PAUP to run a phylogenetic analysis to determine evolutionary relationships of the introns attached to the pseudogene pieces. (DOCX) [file pone.0035168.s003.docx]

**cox2i691**

#NEXUS

[MacClade 4.05 registered to Yin-Long Qiu Lab, EEB, University of Michigan]

BEGIN DATA;

DIMENSIONS NTAX=5 NCHAR=2735;

FORMAT DATATYPE=DNA MISSING=? GAP=- MATCHCHAR=. INTERLEAVE ;

MATRIX

[ 10 20 30 40 50]

[ . . . . .]

Huperzia_cox2_i691 GTGCGCCCGAATACATAGGCCGACTGCTGAGCCCACTCTGGCTCAGCCGC [50]

Huperzia_cox2_in_259730_259261 ................................................A. [50]

Phaeoceros_cox2i691 .......................T........T.............T... [50]

Anomodon_cox2i691 .............A...................T.T..........T... [50]

Vitis_cox2i691 .........G.A...................................... [50]

[ 60 70 80 90 100]

[ . . . . .]

Huperzia_cox2_i691 ---ACCTTCTCGTTCTTTGGCCAACCTGGATGCGAGCTACCTAAAGA--- [94]

Huperzia_cox2_in_259730_259261 CAC............----TT.GG..C..T........G.....G..GCA [96]

Phaeoceros_cox2i691 ---.........AA.AGG.CT........G.......CG..C..G..--- [94]

Anomodon_cox2i691 ---..T......AA.AAG.CA-......AG...........C...A.A-- [94]

Vitis_cox2i691 ---...----------------.C..G..G.......C...CG.GA.GCA [81]

[ 110 120 130 140 150]

[ . . . . .]

Huperzia_cox2_i691 -GCTATCATAGCAATATCATGGCTAGAGCAGTAGGTAAAAGCTGGGGATC [143]

Huperzia_cox2_in_259730_259261 -.......C...............G.....AG.......---C....... [142]

Phaeoceros_cox2i691 -......T.........TT......C...CA....G......C.A..... [143]

Anomodon_cox2i691 -................................A.G......C....... [143]

Vitis_cox2i691 A.....T.C...G.----GC....G....T.....G.---..C.A...G. [124]

[ 160 170 180 190 200]

[ . . . . .]

Huperzia_cox2_i691 GA-----TGGGC---CT-------GCTGCTGCCCCGGCTCCCCAGTAAAG [178]

Huperzia_cox2_in_259730_259261 .G-----GC...GGT..-------..CCT------A...-.......... [173]

Phaeoceros_cox2i691 ..-----.....---..-------..CC.CATTAG....T...G..G... [178]

Anomodon_cox2i691 ..-----.....---..-------..CC.CATTAG........G.GG... [178]

Vitis_cox2i691 A.GGCAG.A.ATAAGA.AGAAGAA.GG..CA----....-...G..GG.. [169]

[ 210 220 230 240 250]

[ . . . . .]

Huperzia_cox2_i691 CGGAGGATACGGTTAGGATAACGAGCTTGAAACGCGGAGCCCGTCTGAAG [228]

Huperzia_cox2_in_259730_259261 .A......G....G.......A...T---.....A--------------- [205]

Phaeoceros_cox2i691 .A............................G..............CA... [228]

Anomodon_cox2i691 .A.......T...................................CT... [228]

Vitis_cox2i691 .A.....G................A..................------- [212]

[ 260 270 280 290 300]

[ . . . . .]

Huperzia_cox2_i691 CTGGACCGAACCTCC-AAGCAAGATATAGCGGCGAGCAAGTGGTTAGTAG [277]

Huperzia_cox2_in_259730_259261 ----------------...G.G..G...AAA.-..A------...--.G. [230]

Phaeoceros_cox2i691 .A....T....G...C.....G.............A..C........... [278]

Anomodon_cox2i691 .........C.G...C.....G...............G........A..A [278]

Vitis_cox2i691 -----------------...GGC------........G..........-. [238]

[ 310 320 330 340 350]

[ . . . . .]

Huperzia_cox2_i691 GCCAATCGCGCCGGACCTGCAGTTGATGGCATTATCCCCTGCAGC----A [323]

Huperzia_cox2_in_259730_259261 ..A.T..ATT.TAC.-----.......C.G.A-.CT.TT..T.------- [267]

Phaeoceros_cox2i691 ......A....T.A....................G..T.C..G..----. [324]

Anomodon_cox2i691 A.....A.T.TTAA...C................G.TT....G..----. [324]

Vitis_cox2i691 ......A....----...--........A....CCT.T....G..TGGC. [282]

[ 360 370 380 390 400]

[ . . . . .]

Huperzia_cox2_i691 CTCGAGGAAC---CACAGGGCACTCCATACAGAGCAAG----TCTGAGAG [366]

Huperzia_cox2_in_259730_259261 --T.G.CG.TTTT.T.T...-.G.TA..C..A.A....----...----- [305]

Phaeoceros_cox2i691 ......T...---.........................----.......A [367]

Anomodon_cox2i691 ......A...---.....................T...----..C..... [367]

Vitis_cox2i691 ..........---...G............A........CAAG...T..G. [329]

[ 410 420 430 440 450]

[ . . . . .]

Huperzia_cox2_i691 ATCAGACGCCCGCGCAAGGACCTAAATTCTCAC---GAGTTAAACCCCAA [413]

Huperzia_cox2_in_259730_259261 -..----------------------------------------------- [307]

Phaeoceros_cox2i691 ..................A..............TAA...G....AA.A.. [417]

Anomodon_cox2i691 .................................TAG...G....A..T.. [417]

Vitis_cox2i691 ..G....................C........TTAG...G.CG.A..A.- [378]

[ 460 470 480 490 500]

[ . . . . .]

Huperzia_cox2_i691 TTCGGACCTAGGAAAGTCGGGGCTAACC-ATCCCCATG---------ACA [453]

Huperzia_cox2_in_259730_259261 -------------------------------------------------- [307]

Phaeoceros_cox2i691 G.........T............C...T-.........---------... [457]

Anomodon_cox2i691 ..........T.G.............A.-.........---------G.. [457]

Vitis_cox2i691 ---.......T.G............C..CG........GGCAACGCA... [425]

[ 510 520 530 540 550]

[ . . . . .]

Huperzia_cox2_i691 GTGTCCTGAGG---GAGTT--CAGAGGCCTTATAGTAACACAGACCTTTT [498]

Huperzia_cox2_in_259730_259261 -------------------------------------------------- [307]

Phaeoceros_cox2i691 T...G..TG..---.....--.........C......G...G....C... [502]

Anomodon_cox2i691 .....G.....---.....--.........C......TT....G...C.. [502]

Vitis_cox2i691 ...........GAG.....--T...............G...G...T.C.. [473]

[ 560 570 580 590 600]

[ . . . . .]

Huperzia_cox2_i691 C--------AGG----TCATGCGAAGGGGGCCAGTCCAAGATCGTACTGT [536]

Huperzia_cox2_in_259730_259261 -------------------------------------------------- [307]

Phaeoceros_cox2i691 T--------.A.GTCG......C....A..............T....... [544]

Anomodon_cox2i691 .--------...----.................A..............T. [540]

Vitis_cox2i691 TTTCTTTCT...----..........A....................... [519]

[ 610 620 630 640 650]

[ . . . . .]

Huperzia_cox2_i691 TCCTCTCTTTTTTGCAACAGACGTTCTCAACGGATCTGTACACCACTCTA [586]

Huperzia_cox2_in_259730_259261 -------------------------------------------------- [307]

Phaeoceros_cox2i691 ....T.ACCAAG-A....G.G..C..C..G..........GG...T.... [593]

Anomodon_cox2i691 C...T.AC.AAG-A.....AGA.C.......AAG...A...G.T.G.T.. [589]

Vitis_cox2i691 ......ACAAAG-A.........C.........--..AGG.G.......C [566]

[ 660 670 680 690 700]

[ . . . . .]

Huperzia_cox2_i691 CGCGCAAGCTGGACTGGACAGACCGTGTCC--GTCTCTCGA--------- [625]

Huperzia_cox2_in_259730_259261 -------------------------------------------------- [307]

Phaeoceros_cox2i691 .A........AA.T..........A..C.T--........G--------- [632]

Anomodon_cox2i691 ...T....T.AA..........T.T...TT--.......A.--------- [628]

Vitis_cox2i691 TTTCTG..T.ATT.CA.CTTCTT.A.TCTTTC..GC.G..GTGAACAAAC [616]

[ 710 720 730 740 750]

[ . . . . .]

Huperzia_cox2_i691 --------------------------CAGCGGAACAGGTAAAGACCCGCA [649]

Huperzia_cox2_in_259730_259261 -------------------------------------------------- [307]

Phaeoceros_cox2i691 --------------------------....A..GT....G..A...T... [656]

Anomodon_cox2i691 --------------------------....CAT.TG...G.G..T.TA.. [652]

Vitis_cox2i691 AAAACAAAAAAGAAAAAGGGCCGTCT........GGA.-..G....T... [665]

[ 760 770 780 790 800]

[ . . . . .]

Huperzia_cox2_i691 AAGGCAAAGACGGCAAATGCTACGGATTGATCAATAAAAAATGGAGTGAC [699]

Huperzia_cox2_in_259730_259261 -------------------------------------------------- [307]

Phaeoceros_cox2i691 ...C...........T---------------------------------- [672]

Anomodon_cox2i691 ..AA..G.C......----------------------------------- [667]

Vitis_cox2i691 .C....G....TA.TG.CC..----------------------------- [686]

[ 810 820 830 840 850]

[ . . . . .]

Huperzia_cox2_i691 CCTTTGTTTCTAGCTGTCTGTTACAAGTCTATTTTTTTTTCCGAGATAAG [749]

Huperzia_cox2_in_259730_259261 -------------------------------------------------- [307]

Phaeoceros_cox2i691 -----------------------------------------.A..T.T-- [679]

Anomodon_cox2i691 --------------------------------------------....-- [671]

Vitis_cox2i691 --------------------------------------C.TTTT.T.C-- [696]

[ 860 870 880 890 900]

[ . . . . .]

Huperzia_cox2_i691 CCTGGAAATATGACACGGGATCCGTAAATGAAATTCTGATGGTCTTTCCC [799]

Huperzia_cox2_in_259730_259261 -------------------------------------------------- [307]

Phaeoceros_cox2i691 -------------------------------------------------- [679]

Anomodon_cox2i691 -------------------------------------------------- [671]

Vitis_cox2i691 -------------------------------------------------- [696]

[ 910 920 930 940 950]

[ . . . . .]

Huperzia_cox2_i691 CAGCAGGGCCCTTTTTGGCGGGAAGCTAAGCAAAAGCCAGTTGTCTTCAC [849]

Huperzia_cox2_in_259730_259261 -------------------------------------------------- [307]

Phaeoceros_cox2i691 -------------------------T..G..G-------------C.... [691]

Anomodon_cox2i691 -------------------------...C.G.-------------..... [683]

Vitis_cox2i691 -------------------------...GC.G-------------.CTGT [708]

[ 960 970 980 990 1000]

[ . . . . .]

Huperzia_cox2_i691 GGCTCGTAGAAGGGAAATAACCAAGCCAGGCGAGATAGAAACGAGACCTC [899]

Huperzia_cox2_in_259730_259261 -------------------------------------------------- [307]

Phaeoceros_cox2i691 -------------------------------------------------- [691]

Anomodon_cox2i691 -------------------------------------------------- [683]

Vitis_cox2i691 -------------------------------------------------- [708]

[ 1010 1020 1030 1040 1050]

[ . . . . .]

Huperzia_cox2_i691 TGGGCAGAGAGCCGCCGCCCGAGGGAAAGGGTCGTAAAAAATACCCGTTT [949]

Huperzia_cox2_in_259730_259261 -------------------------------------------------- [307]

Phaeoceros_cox2i691 -------------------------------------------------- [691]

Anomodon_cox2i691 -------------------------------------------------- [683]

Vitis_cox2i691 -------------------------------------------------- [708]

[ 1060 1070 1080 1090 1100]

[ . . . . .]

Huperzia_cox2_i691 CTAATGCCAGCCCATTCTGAGCCTGTCTTTCTCGACTGCTCGCACGAATT [999]

Huperzia_cox2_in_259730_259261 -------------------------------------------------- [307]

Phaeoceros_cox2i691 ------------------------------------------TG....G- [698]

Anomodon_cox2i691 ------------------------------------------.-.....- [689]

Vitis_cox2i691 ------------------------------------------T....G.C [716]

[ 1110 1120 1130 1140 1150]

[ . . . . .]

Huperzia_cox2_i691 CCGGCCCCACTGAGTATAACGCATACGGCCCTAGATAATCTTGACTTGAA [1049]

Huperzia_cox2_in_259730_259261 -------------------------------------------------- [307]

Phaeoceros_cox2i691 -----------A....CG-------------------------------- [705]

Anomodon_cox2i691 -----------..A...T-------------------------------- [696]

Vitis_cox2i691 .----------.G.A.GC-------------------------------- [724]

[ 1160 1170 1180 1190 1200]

[ . . . . .]

Huperzia_cox2_i691 AGGCTCTTGGCCATGAGAAGGGGATTTCCTACAGGTTTTGACTCGATTCC [1099]

Huperzia_cox2_in_259730_259261 -------------------------------------------------- [307]

Phaeoceros_cox2i691 -------------------------------------------------- [705]

Anomodon_cox2i691 -------------------------------------------------- [696]

Vitis_cox2i691 -------------------------------------------------- [724]

[ 1210 1220 1230 1240 1250]

[ . . . . .]

Huperzia_cox2_i691 CCACCCAGTCATGCTAAATCTGGCGAAAAGAGTAGGATGTCAGCGCACCC [1149]

Huperzia_cox2_in_259730_259261 -------------------------------------------------- [307]

Phaeoceros_cox2i691 -----...C.G.A..C..GTA.---------------------------- [722]

Anomodon_cox2i691 -----G..CG..T..----T..---------------------------- [709]

Vitis_cox2i691 -----.T.GA..C..C...A..AG.G------------------------ [745]

[ 1260 1270 1280 1290 1300]

[ . . . . .]

Huperzia_cox2_i691 TAAATCTATTAACGAAAGCGCTTGACGCGGAATCTTCTCTTGACCTAAAG [1199]

Huperzia_cox2_in_259730_259261 -------------------------------------------------- [307]

Phaeoceros_cox2i691 ----....CA.TTAC.G.T..A----------------------.CG... [746]

Anomodon_cox2i691 ----...---------G.TA.-------------------------G.G. [721]

Vitis_cox2i691 ---...CC.C.----..ATAGAG-----------------------..G. [765]

[ 1310 1320 1330 1340 1350]

[ . . . . .]

Huperzia_cox2_i691 GCCCACGGCCCAAAAGTTGAAGCAGGAACCCCGCCCGCAGGGGCCGCCGT [1249]

Huperzia_cox2_in_259730_259261 -------------------------------------------------- [307]

Phaeoceros_cox2i691 ..G.C.A......------------------------------------- [759]

Anomodon_cox2i691 ----------.T.------------------------------------- [724]

Vitis_cox2i691 ..GGG.A.GGTGG------------------------------------- [778]

[ 1360 1370 1380 1390 1400]

[ . . . . .]

Huperzia_cox2_i691 CTAAGCCCGCTACTCTGATTACTCTCCACGTCCTCTCAAAATTTGTGCAT [1299]

Huperzia_cox2_in_259730_259261 -------------------------------------------------- [307]

Phaeoceros_cox2i691 -----------------------------------------....----- [763]

Anomodon_cox2i691 -----------------------------------------....----- [728]

Vitis_cox2i691 ------------------------------------------.C..---- [782]

[ 1410 1420 1430 1440 1450]

[ . . . . .]

Huperzia_cox2_i691 AAGCTCCAGTCAAAATTGGTGAGGGGAAAACGAGGAAATTAAAGCTAGGA [1349]

Huperzia_cox2_in_259730_259261 -------------------------------------------------- [307]

Phaeoceros_cox2i691 -----------..C.--------------------------......A.. [776]

Anomodon_cox2i691 -----------....--------------------------.G..A.AA. [741]

Vitis_cox2i691 -----------------------------------------.GATC...G [791]

[ 1460 1470 1480 1490 1500]

[ . . . . .]

Huperzia_cox2_i691 AACACCTAACCTACCAAATCTCTCTCTGCGAAGACCGGAAAAAAAACACC [1399]

Huperzia_cox2_in_259730_259261 -------------------------------------------------- [307]

Phaeoceros_cox2i691 .T...--------------------------------------------- [781]

Anomodon_cox2i691 ..A.T--------------------------------------------- [746]

Vitis_cox2i691 ..A----------------------------------------------- [794]

[ 1510 1520 1530 1540 1550]

[ . . . . .]

Huperzia_cox2_i691 TTTTATTTCATGGCTACGTCAACGTATTCCCGACCCCAAAGACGACCCAA [1449]

Huperzia_cox2_in_259730_259261 -------------------------------------------------- [307]

Phaeoceros_cox2i691 -------------------------------.G.--.GG----C.....- [793]

Anomodon_cox2i691 -------------------------------...A..G.----..T.TG- [760]

Vitis_cox2i691 -------------------------------.G.T.AGG.A.G..G.AG. [813]

[ 1560 1570 1580 1590 1600]

[ . . . . .]

Huperzia_cox2_i691 ACTTGGCGAAGACTGATGTATCTAGGATACGCCGACGACTCTGTACCCC- [1498]

Huperzia_cox2_in_259730_259261 -------------------------------------------------- [307]

Phaeoceros_cox2i691 -------------..C..CGC.------------------...CG....- [811]

Anomodon_cox2i691 -------------.A.G.A..A------------------..------.- [772]

Vitis_cox2i691 G------A.G.GG.TG..CGC.------------------..CCG....A [839]

[ 1610 1620 1630 1640 1650]

[ . . . . .]

Huperzia_cox2_i691 CATAACCAGGTCAGTTAGGAGGGAAGCAAGGATCATCAAGGATCACAAAA [1548]

Huperzia_cox2_in_259730_259261 -------------------------------------------------- [307]

Phaeoceros_cox2i691 T.-----..C.T.A--------------------------------.G.. [824]

Anomodon_cox2i691 TG-----.A--T..--------------------------------.... [783]

Vitis_cox2i691 TC.T..T.A--T.A.A------------------------------.TG. [857]

[ 1660 1670 1680 1690 1700]

[ . . . . .]

Huperzia_cox2_i691 AAAACTTCCTTTCCAAGAAGACGGGGCTGCAACTCAGTATTGACGAGGCG [1598]

Huperzia_cox2_in_259730_259261 -------------------------------------------------- [307]

Phaeoceros_cox2i691 ...G..GGA..CG.CG---------------------------------- [840]

Anomodon_cox2i691 .G...------C...G---------------------------------- [793]

Vitis_cox2i691 .GGG...TGA.G...GC...CAA.CA------------------------ [883]

[ 1710 1720 1730 1740 1750]

[ . . . . .]

Huperzia_cox2_i691 AAAGAAAAAAATATCCCACTTAGTTTGGCCAAAGACGGCTTCGATTTCTT [1648]

Huperzia_cox2_in_259730_259261 -------------------------------------------------- [307]

Phaeoceros_cox2i691 --------------.T.G..-G.C-------------------------- [849]

Anomodon_cox2i691 --------------....T.....-------------------------- [803]

Vitis_cox2i691 --------------...TTG.....------------------------- [894]

[ 1760 1770 1780 1790 1800]

[ . . . . .]

Huperzia_cox2_i691 AGGCGCCCACTGCAATACGCAGCCATCTCAAGTGACAAGTACAGGTGCTA [1698]

Huperzia_cox2_in_259730_259261 -------------------------------------------------- [307]

Phaeoceros_cox2i691 -------------------------------------------------- [849]

Anomodon_cox2i691 -------------------------------------------------- [803]

Vitis_cox2i691 -------------------------------------------------- [894]

[ 1810 1820 1830 1840 1850]

[ . . . . .]

Huperzia_cox2_i691 CTTTTGGCGCGCCCAGCCCAATCCAACGAAATAAGGCTAGGAATAATTAC [1748]

Huperzia_cox2_in_259730_259261 -------------------------------------------------- [307]

Phaeoceros_cox2i691 ----------------------------GGCGG.AC..T.T.GG.C..C. [871]

Anomodon_cox2i691 ----------------------------.......C.G..C..A...--- [822]

Vitis_cox2i691 -----------------------------GT.GG.--G..T.GGGCG.TA [913]

[ 1860 1870 1880 1890 1900]

[ . . . . .]

Huperzia_cox2_i691 GGCACCCATCAAAAAACTTCTCATAAAGCTTAAAGAAGCCGCGCCCGCCC [1798]

Huperzia_cox2_in_259730_259261 -------------------------------------------------- [307]

Phaeoceros_cox2i691 TC.G..-------------------------------------------- [877]

Anomodon_cox2i691 -----.-------------------------------------------- [823]

Vitis_cox2i691 A.....-------------------------------------------- [919]

[ 1910 1920 1930 1940 1950]

[ . . . . .]

Huperzia_cox2_i691 GCCATAACCTGGTTAGTTCCCCGCTCCCGCTTGCCAAAGAAAGAGATCTC [1848]

Huperzia_cox2_in_259730_259261 -------------------------------------------------- [307]

Phaeoceros_cox2i691 ----------..C.TA..A..----------------------------- [888]

Anomodon_cox2i691 ----------A...CT..TT.----------------------------- [834]

Vitis_cox2i691 ---------.T...TT..AGTATA-------------------------- [934]

[ 1960 1970 1980 1990 2000]

[ . . . . .]

Huperzia_cox2_i691 ATCACGACGTGGGACCACGCAGACATACCAAAGTTC--TACCA-TC---A [1892]

Huperzia_cox2_in_259730_259261 -------------------------------------------------- [307]

Phaeoceros_cox2i691 ------....A.................T..G....--..TG.-..-GA. [928]

Anomodon_cox2i691 ------..-----.....-..A.A.....C...CCAGGC.AG.-..AGA. [871]

Vitis_cox2i691 -----A.AA..A..AT.-AT.A.A.G.AG..GC.C.CC.TTG.A.AATA. [978]

[ 2010 2020 2030 2040 2050]

[ . . . . .]

Huperzia_cox2_i691 AAAAGCCTAT----------GGCATTGTGAACTT-----CTACTCCTTCG [1927]

Huperzia_cox2_in_259730_259261 -------------------------------------------------- [307]

Phaeoceros_cox2i691 GGGG...C..----------..TC......---.-----...T.....T. [960]

Anomodon_cox2i691 C..G.GTAGC----------A....C...-------------------T. [892]

Vitis_cox2i691 T..G.TAAGCTTTCAAGCCAA..C..CC.CTGG.TTTGG..TGC..C.T. [1028]

[ 2060 2070 2080 2090 2100]

[ . . . . .]

Huperzia_cox2_i691 CAGCGAACTGCAACTCCCTACTCAAAATTACCTATCTCTGAAAAA----- [1972]

Huperzia_cox2_in_259730_259261 -------------------------------------------------- [307]

Phaeoceros_cox2i691 ..TT...GC.......A.....G..C..C..........T.....AAAAA [1010]

Anomodon_cox2i691 A.AA..C..C.GG..AA-.G..GG.GG.C--....T.T.TGCCT.----- [934]

Vitis_cox2i691 T.TA.G-T..G.G..A--.G..T..CGCC-.T...TAT.AG.G..AG--G [1072]

[ 2110 2120 2130 2140 2150]

[ . . . . .]

Huperzia_cox2_i691 --TTTTTCTGATCTTGGC------TTTCAAATAAGTTGCAGACCACTATG [2014]

Huperzia_cox2_in_259730_259261 -------------------------------------------------- [307]

Phaeoceros_cox2i691 GTAACCCTC.GC.--...GGGCGGCC.GG..G.......G....GT..CC [1058]

Anomodon_cox2i691 -C...C.TC.G..--.--------C..G--------C..G..TTT.GGCC [965]

Vitis_cox2i691 GGAAAGGG..G..G.A.A------.CAG---------..T..T.TT.GAT [1107]

[ 2160 2170 2180 2190 2200]

[ . . . . .]

Huperzia_cox2_i691 TAAGTGTTCCGTCGATTTGGTGGGAGATTAGCCTGTACGAGTACAG-ACC [2063]

Huperzia_cox2_in_259730_259261 -------------------------------------------------- [307]

Phaeoceros_cox2i691 .TGT.T.GG.A-------..CA.............CC...AG...AAGTT [1101]

Anomodon_cox2i691 .--------.A-------CAAA...AG---------C.A..G.T.T-GTT [990]

Vitis_cox2i691 C.GAA..G.TC-----------.T....C.----.GCT..T.GT.A-.T. [1141]

[ 2210 2220 2230 2240 2250]

[ . . . . .]

Huperzia_cox2_i691 CGGGGGCTGCTATTAGACCCG--AAGAGCT---TTTTCGCCATAAACGTG [2108]

Huperzia_cox2_in_259730_259261 -------------------------------------------------- [307]

Phaeoceros_cox2i691 T...--..ATAT---......--......C---.GAAA...........A [1141]

Anomodon_cox2i691 .CAA--T.A.ACGCG.G....CT...G..C----GAAG........G..T [1034]

Vitis_cox2i691 A...T...CTAC.AC..T.TTAGGCAG...GAT.GAAGAT..G..GT.CT [1191]

[ 2260 2270 2280 2290 2300]

[ . . . . .]

Huperzia_cox2_i691 TTTAAGCCTGAGCAACTAGAGCTCCCAGAGAAGATCGTAGACCTGAGTGA [2158]

Huperzia_cox2_in_259730_259261 -------------------------------------------------- [307]

Phaeoceros_cox2i691 .............C.....C...AG..A.C.G............A..--- [1188]

Anomodon_cox2i691 ......T........T...C......GC.T.....T.....TGC...--- [1081]

Vitis_cox2i691 C.ACGAT.A.G.TGGTCGT..A.--...GCT-....T.T..T.A..AGTG [1238]

[ 2310 2320 2330 2340 2350]

[ . . . . .]

Huperzia_cox2_i691 GCTAGGACG-CC-----GCCAAGTTA--------ACCCTAAGGGT--GTG [2192]

Huperzia_cox2_in_259730_259261 -------------------------------------------------- [307]

Phaeoceros_cox2i691 -..G..G..-..AAG--..G.CC...AATTCTTGG...CG..T..GT... [1234]

Anomodon_cox2i691 -..G..G..-..AAA--.TTG.C.CG--CTCGGGGTTA.TTC...-T... [1124]

Vitis_cox2i691 .TCG.A...AT.AGGGT..TCCCC..CGATCTTAGG.AGGCT.A.-T..A [1287]

[ 2360 2370 2380 2390 2400]

[ . . . . .]

Huperzia_cox2_i691 CGCCA-----TCTGAGGATGTTCTCGAGTGGAGATGCA-----TCATCTG [2232]

Huperzia_cox2_in_259730_259261 -------------------------------------------------- [307]

Phaeoceros_cox2i691 .A...-----.T..T...CC...CTAG..A........-----....... [1274]

Anomodon_cox2i691 TA.T.-----.TC.TA...C.AA...G.GA..T..A..-----.------ [1158]

Vitis_cox2i691 AAT..GGTTGCTC.TA...C.--..AGAG..CTGCT.GCTTTC..GCT.C [1335]

[ 2410 2420 2430 2440 2450]

[ . . . . .]

Huperzia_cox2_i691 AGAAAGATGATATACGGTA---TTAGAGCT---AAGATTGGGTGGATTTA [2276]

Huperzia_cox2_in_259730_259261 -------------------------------------------------- [307]

Phaeoceros_cox2i691 .A....G...A.G...------.GGC....---......-AT.CAG---G [1311]

Anomodon_cox2i691 --.G..G.A.G.G...------.A......---..A...C.C.C.G---G [1194]

Vitis_cox2i691 C..G..GC..AGGGA.CCTGAC...CG.T.TCC...CC...CGC..AGCG [1385]

[ 2460 2470 2480 2490 2500]

[ . . . . .]

Huperzia_cox2_i691 ACC--------TCTGACCAGTGAGTGCGGCGGGCATTCAAGC--TTAAGC [2316]

Huperzia_cox2_in_259730_259261 -------------------------------------------------- [307]

Phaeoceros_cox2i691 GA---------.GCC...CA.CTA...T.GA...T....G.T--G..... [1350]

Anomodon_cox2i691 .AG--------.G.T...TA.--------AA------.C..T--G....T [1220]

Vitis_cox2i691 .AGGGATTGGA.T.C...TA.--------GA-----...GATCAG.GG.. [1422]

[ 2510 2520 2530 2540 2550]

[ . . . . .]

Huperzia_cox2_i691 A-AATACCATTGCGCAGACAACA----CCGCAACGCCTATTACCACGGT- [2360]

Huperzia_cox2_in_259730_259261 -------------------------------------------------- [307]

Phaeoceros_cox2i691 .-G.....C...T......T...CTTCTTCTGG.--..T.C.T...A..- [1396]

Anomodon_cox2i691 .TG.A..TGC..T.TG.......AATA..A.....-.CGCC.--..A..- [1266]

Vitis_cox2i691 .----.....A.T-----TT..TTTTTT..TGGT.-T.G..GA.GTT..T [1462]

[ 2560 2570 2580 2590 2600]

[ . . . . .]

Huperzia_cox2_i691 -------AACCTTACGGTCG-CCA---ACAT-GGAGAAACTTTCAGCTTG [2398]

Huperzia_cox2_in_259730_259261 -------------------------------------------------- [307]

Phaeoceros_cox2i691 -------..........C.A-..G---.T..-.A...T...........A [1434]

Anomodon_cox2i691 -------G..T.ATG..CT.-.GGCGC.G..-TA...T...GAG.A..CT [1307]

Vitis_cox2i691 GAAAGCT..TT...AA..A.G..TCGCTTT.C....CTG..CC.....CA [1512]

[ 2610 2620 2630 2640 2650]

[ . . . . .]

Huperzia_cox2_i691 CACTATTGCG-GAGGAGGGGACCGCAAGGCCCAAGGTTAAGATCTAGGAG [2447]

Huperzia_cox2_in_259730_259261 -------------------------------------------------- [307]

Phaeoceros_cox2i691 ........T.T.T....-------T......----..CG.T........A [1473]

Anomodon_cox2i691 A.......T.-...A...TTG..-T....T...................A [1355]

Vitis_cox2i691 ......G.T.-.......T---------...----..G............ [1548]

[ 2660 2670 2680 2690 2700]

[ . . . . .]

Huperzia_cox2_i691 TGTGAGCAGTACGAGCTGAAAGGCTCCCATACTGTTTGGAGGGCAGGGGG [2497]

Huperzia_cox2_in_259730_259261 -------------------------------------------------- [307]

Phaeoceros_cox2i691 G.C....C.......T.........T.....TG...CT.........CTC [1523]

Anomodon_cox2i691 G...................................C..........--- [1402]

Vitis_cox2i691 .................................................. [1598]

[ 2710 2720 2730 ]

[ . . . ]

Huperzia_cox2_i691 GGCGAGCAGAGATGCTTCT----CCTGACCCCTAT [2528]

Huperzia_cox2_in_259730_259261 ----------------------------------- [307]

Phaeoceros_cox2i691 .-----..AG...A.C.AC--AG............ [1551]

Anomodon_cox2i691 ---------G.C.TT.-------............ [1421]

Vitis_cox2i691 ------..T......CAAACAAA............ [1627]

;

END;

**cox3i171**

#NEXUS

[MacClade 4.05 registered to Yin-Long Qiu Lab, EEB, University of Michigan]

BEGIN DATA;

DIMENSIONS NTAX=7 NCHAR=946;

FORMAT DATATYPE=DNA MISSING=? GAP=- MATCHCHAR=. INTERLEAVE ;

MATRIX

[ 10 20 30 40 50]

[ . . . . .]

Huperzia_cox3_i171g2 GTGCGCCCGAAGACATTTACTCCGACGAGGAGGGCATTCTCCTCTCCTCC [50]

Huperzia_cox3_i171g2_82426_81719 ...............C................T.T.A------------- [37]

Huperzia_cox3_i171g2_321018_321725 ...............C................T.T.A------------- [37]

Huperzia_cox3_i171g2_379535_380262 ...............C................T.T.A------------- [37]

Pleurozia_cox3_i171 .............T.CA.CG.A....A..AG.A..TA..CA....TT..T [50]

Marchantia_cox3i171 .........G...T.CA.CG.A....A..A..A..TA..CA....TT.TT [50]

Treubia_cox3_i171 .........G...T.CA.CG.A....A..A..A..TA..CA....TT.TT [50]

[ 60 70 80 90 100]

[ . . . . .]

Huperzia_cox3_i171g2 GTGACAT---------CTCA--GGCTAGGTCACGCGCTCAACCTGTAGG- [88]

Huperzia_cox3_i171g2_82426_81719 .......---------....--.....................---...- [72]

Huperzia_cox3_i171g2_321018_321725 .......---------....--.....................---...- [72]

Huperzia_cox3_i171g2_379535_380262 .......---------....--.....................---...- [72]

Pleurozia_cox3_i171 ...C.G.TCAGGCTAG..T.TAA......G...AG.......T..CG.AT [100]

Marchantia_cox3i171 ...C...TCAGGCTAG....TAA......A...AG.......T..C..AG [100]

Treubia_cox3_i171 ...C...TCAGGCTAG....TAA......A...AG.......T..C..AG [100]

[ 110 120 130 140 150]

[ . . . . .]

Huperzia_cox3_i171g2 --------GT------ACACACCCGGGAGCAGCAGGTGTAAATCGAGG-- [122]

Huperzia_cox3_i171g2_82426_81719 --------..------G...C.....C.....................-- [106]

Huperzia_cox3_i171g2_321018_321725 --------..------G...C.....C.....................-- [106]

Huperzia_cox3_i171g2_379535_380262 --------..------G...C.....C.....................-- [106]

Pleurozia_cox3_i171 GAGCCATA..AACATGG.TT..T...........A..T.C....AGA.AA [150]

Marchantia_cox3i171 GAGCCATA..AACATGG.TT..T...........A..T.C....AGA.AA [150]

Treubia_cox3_i171 GAGCCATA..AACATGG.TT..T...........A..T.C....AGA.AA [150]

[ 160 170 180 190 200]

[ . . . . .]

Huperzia_cox3_i171g2 CTCGAAGAATGTGGGTCTGCCCCCACCGAGATGCCTCGAGGGAGCAGAAG [172]

Huperzia_cox3_i171g2_82426_81719 .----......--------.......TA----.................. [140]

Huperzia_cox3_i171g2_321018_321725 .----......--------.......TA----.................. [140]

Huperzia_cox3_i171g2_379535_380262 .----......--------.......TA----.................. [140]

Pleurozia_cox3_i171 ..GTGG.GC..--------..A..G.TA...C..TCT..AA......G.. [192]

Marchantia_cox3i171 ..GTGG.GC..--------..A..G.TA...C..TCT..AA......... [192]

Treubia_cox3_i171 ..GTGG.GC..--------..A..G.TA...C..TCT..AA......... [192]

[ 210 220 230 240 250]

[ . . . . .]

Huperzia_cox3_i171g2 GTACGGTTAGGATAACGCACGTGATATTACGCATTTCCCGGAGCAGATAG [222]

Huperzia_cox3_i171g2_82426_81719 ......................A...C...........T.AC........ [190]

Huperzia_cox3_i171g2_321018_321725 ......................A...C...........T.AC........ [190]

Huperzia_cox3_i171g2_379535_380262 ......................A..---..........T.AC........ [187]

Pleurozia_cox3_i171 ...G..C...T.....TA..T....---.....A.G.T..CGT..C.... [239]

Marchantia_cox3i171 ...G..C.........TA..T....---.....A.G.T..CGT..C.... [239]

Treubia_cox3_i171 ...G..C.........TA..T....---.....A.G.T..CGT..C.... [239]

[ 260 270 280 290 300]

[ . . . . .]

Huperzia_cox3_i171g2 GCCCTCCTGTCCCAAGCAGAATATAGTGGCAGGGGCACGACAAGC----- [267]

Huperzia_cox3_i171g2_82426_81719 .........................................G...---CC [237]

Huperzia_cox3_i171g2_321018_321725 .........................................G...---CC [237]

Huperzia_cox3_i171g2_379535_380262 ..................................A......G...AAGCC [237]

Pleurozia_cox3_i171 CT....T....T............TAC....A.A.....GT...TAGGCC [289]

Marchantia_cox3i171 CT....T....T............TAC....A.A.....GT...TAGGCC [289]

Treubia_cox3_i171 CT....T....T............TAC....A.A.....GT...TAGGCC [289]

[ 310 320 330 340 350]

[ . . . . .]

Huperzia_cox3_i171g2 ----CAG-GGCCAGAGTTGGGCACAATACATGGTGTGTGCACAGCACC-- [310]

Huperzia_cox3_i171g2_82426_81719 ---TT.-----.....................A...............-- [277]

Huperzia_cox3_i171g2_321018_321725 ---TT.-----.....................A...............-- [277]

Huperzia_cox3_i171g2_379535_380262 AAGC...-.....A..................................TG [286]

Pleurozia_cox3_i171 TCCT.G.A....GA.ACA.TC..................T....T...-- [337]

Marchantia_cox3i171 TCCTTG.A..T.GA.ACA.TC..................T....T...-- [337]

Treubia_cox3_i171 TCCTTG.A..T.GA.ACA.TC..................T....T...-- [337]

[ 360 370 380 390 400]

[ . . . . .]

Huperzia_cox3_i171g2 -TGAGAAATCGTGGGGAACTCTACGAGGAGACCGGCT--GGGCAACCAGC [357]

Huperzia_cox3_i171g2_82426_81719 -C..C........A.....------...........CTA........... [320]

Huperzia_cox3_i171g2_321018_321725 -C..C........A.....------...........CTA........... [320]

Huperzia_cox3_i171g2_379535_380262 AC..C.........TA...G-----...........CTA........... [331]

Pleurozia_cox3_i171 -...A.G.C.......C....CGAC.A.GA..TA...--.A..G.T.... [384]

Marchantia_cox3i171 -...A.G.C.......C....CGAC.A.GA..TA...--.A..G.T.... [384]

Treubia_cox3_i171 -...A.G.C.......C....CGAC.A.GA..TA...--.A..G.T.... [384]

[ 410 420 430 440 450]

[ . . . . .]

Huperzia_cox3_i171g2 CGAATGCGCTAGGACCTCA-CCCTTCGAGAATTCTGCCTCCTTCGAAGGG [406]

Huperzia_cox3_i171g2_82426_81719 ...................-.........-------............AT [362]

Huperzia_cox3_i171g2_321018_321725 ...................-.........-------............AT [362]

Huperzia_cox3_i171g2_379535_380262 ...................-.........-------............AT [373]

Pleurozia_cox3_i171 TA.T.....AG..G...A.A......TG..--..GTTG..T.C.C..A.A [432]

Marchantia_cox3i171 TA.T.....AG..G...A.A.T....TG..--..GTTG..T.C.T..A.A [432]

Treubia_cox3_i171 TA.T.....AG..G...A.A.T....TG..--..GTTG..T.C.T..A.A [432]

[ 460 470 480 490 500]

[ . . . . .]

Huperzia_cox3_i171g2 GGCGGAGAACGAGGAGTGTATGGAAGTCGGGGAAATAATGCATT-CCACG [455]

Huperzia_cox3_i171g2_82426_81719 ....A....--.....G.----.G....................A..... [406]

Huperzia_cox3_i171g2_321018_321725 ....A....--.....G.----.G....................A..... [406]

Huperzia_cox3_i171g2_379535_380262 ....A....--.....G.----.G....................A..... [417]

Pleurozia_cox3_i171 CA..A.-..TA..T.C.A.G.-TG..............CC...CG----. [476]

Marchantia_cox3i171 CA..A.-..TA..T.C.A.G.-TG..............CC...CG----. [476]

Treubia_cox3_i171 CA..A.-..TA..T.C.A.G.-TG..............CC...CG----. [476]

[ 510 520 530 540 550]

[ . . . . .]

Huperzia_cox3_i171g2 GGAATGGCATTCCGAGGGTCCGTAGTAGCGCTTCGGGCGGAAACACTCAT [505]

Huperzia_cox3_i171g2_82426_81719 .T................................................ [456]

Huperzia_cox3_i171g2_321018_321725 .T................................................ [456]

Huperzia_cox3_i171g2_379535_380262 .T................................................ [467]

Pleurozia_cox3_i171 .TG....GG...G.....CT....A....--.....ATTTGGCAGTC..G [524]

Marchantia_cox3i171 .TG....GG...G.....CT....A....--.....ATTTGGCAGTC..G [524]

Treubia_cox3_i171 .TG....GG...G.....CT....A....--.....ATTTGGCAGTC..G [524]

[ 560 570 580 590 600]

[ . . . . .]

Huperzia_cox3_i171g2 TCTACCTGGCCGCGAAGGGACCTAGCTTACGATCGTACTGCTCGAGACCC [555]

Huperzia_cox3_i171g2_82426_81719 ..........TTT.---..........C.............G........ [503]

Huperzia_cox3_i171g2_321018_321725 ..........TTT.---..........C.............G........ [503]

Huperzia_cox3_i171g2_379535_380262 ..........TTT.---..........C.............G........ [514]

Pleurozia_cox3_i171 C..TGGC..TTAA.---.AG.......GT......C....T..T.CCGTG [571]

Marchantia_cox3i171 C..-GGC..TTAA.---.AG.......CT...........T..T.CCGTA [570]

Treubia_cox3_i171 C..-GGC..TTAA.---.AG.......CT...........T..T.CCGTA [570]

[ 610 620 630 640 650]

[ . . . . .]

Huperzia_cox3_i171g2 GAGATCTCGGCGGAGGATGGCAATGCTTCAGCTCGTCCCAGCGGAGCAGG [605]

Huperzia_cox3_i171g2_82426_81719 A.........------.....G..........................A. [547]

Huperzia_cox3_i171g2_321018_321725 A.........------.....G..........................A. [547]

Huperzia_cox3_i171g2_379535_380262 A.........------.....G..........................A. [558]

Pleurozia_cox3_i171 ...G...T..------...T.G.GA.A.TGT......T.G....C..-.. [614]

Marchantia_cox3i171 ...G...T..------...T.G.GA.A.TGT......T......C..-.. [613]

Treubia_cox3_i171 ...G...T..------...T.G.GA.A.TGT......T......C..-.. [613]

[ 660 670 680 690 700]

[ . . . . .]

Huperzia_cox3_i171g2 CCGCGCAGCCAGCAAGGTTGACTGG-----TCCGGCCGCATCCGTATCTC [650]

Huperzia_cox3_i171g2_82426_81719 .........................-----..T.A....G.......... [592]

Huperzia_cox3_i171g2_321018_321725 .........................-----..T.A....G.......... [592]

Huperzia_cox3_i171g2_379535_380262 .........................-----..T.A........-.....T [602]

Pleurozia_cox3_i171 ..AAT.....CAT..A..C..A...-----....T....G.T........ [659]

Marchantia_cox3i171 ..AAT.....CAT..A..C..A...GTCCG....T....G.T........ [663]

Treubia_cox3_i171 ..AAT.....CAT..A..C..A...GTCCG....T....G.T........ [663]

[ 710 720 730 740 750]

[ . . . . .]

Huperzia_cox3_i171g2 ------------------------------------AGTCTCTGC----- [659]

Huperzia_cox3_i171g2_82426_81719 ------------------------------------.........----- [601]

Huperzia_cox3_i171g2_321018_321725 ------------------------------------.........----- [601]

Huperzia_cox3_i171g2_379535_380262 ------------------------------------.........TG--- [613]

Pleurozia_cox3_i171 CATTATTCGGAACGGAATCAAGCTTATTCTGGGAGT.A..C.G..ATTTG [709]

Marchantia_cox3i171 CATTATTCGGAGCAGAATCAAGCTTATTCTGGGAGT.A..C.G..CTTTA [713]

Treubia_cox3_i171 CATTATTCGGAGCAGAATCAAGCTTATTCTGGGAGT.A..C.G..CTTTA [713]

[ 760 770 780 790 800]

[ . . . . .]

Huperzia_cox3_i171g2 ---ACAAATTCAAACC---TA--CCAGGGAA------------------- [682]

Huperzia_cox3_i171g2_82426_81719 ---.............---..--........------------------- [624]

Huperzia_cox3_i171g2_321018_321725 ---.............---..--........------------------- [624]

Huperzia_cox3_i171g2_379535_380262 ---.AG..........---..--........------------------- [636]

Pleurozia_cox3_i171 GTT.TG...C..TC.TAGG..AG.....A..TTTATGCTACAACGCCCTC [759]

Marchantia_cox3i171 GTT.TG...C..TC..AGG..AG.....A..TTGATGCTACAACGCCCTT [763]

Treubia_cox3_i171 GTT.TG...C..TC..AGG..AG.....A..TTGATGCTACAACGCCCTT [763]

[ 810 820 830 840 850]

[ . . . . .]

Huperzia_cox3_i171g2 --------------------------------------------CATGGT [688]

Huperzia_cox3_i171g2_82426_81719 --------------------------------------------...... [630]

Huperzia_cox3_i171g2_321018_321725 --------------------------------------------...... [630]

Huperzia_cox3_i171g2_379535_380262 --------------------------------------------...AA. [642]

Pleurozia_cox3_i171 GGATGGTCCCTCCCATAGAGATTTGAATCATAAGATTGAAAGTT...A.. [809]

Marchantia_cox3i171 GGGTGGTCTCTCTCATAGAGATTTGAATCATAAGATTGAAAGTT...A.. [813]

Treubia_cox3_i171 GGGTGGTCTCTCTCATAGAGATTTGAATCATAAGATTGAAAGTT...A.. [813]

[ 860 870 880 890 900]

[ . . . . .]

Huperzia_cox3_i171g2 -------GGAGATCGGAGGTGCAAGCCATATGAGGCGAAAATCCCACGTA [731]

Huperzia_cox3_i171g2_82426_81719 -------.......................GA..........AT.....C [673]

Huperzia_cox3_i171g2_321018_321725 -------.......................GA..........AT.....C [673]

Huperzia_cox3_i171g2_379535_380262 -------.A.........A...........GA...-A.....ATT....C [684]

Pleurozia_cox3_i171 TATAAGC..............AG....G.T.....T....GC.T.T.... [859]

Marchantia_cox3i171 TATAAGT..............AG....G.T.....T....GC.T.T.... [863]

Treubia_cox3_i171 TATAAGT..............AG....G.T.....T....GC.T.T.... [863]

[ 910 920 930 940 ]

[ . . . . ]

Huperzia_cox3_i171g2 TAGTTTTGAGGGCAGCCACTCAGA-CATGGATGGACTGACCCCTAG [776]

Huperzia_cox3_i171g2_82426_81719 ..................T.....A.T..A.....----------- [708]

Huperzia_cox3_i171g2_321018_321725 ..................T.....A.T..A.....----------- [708]

Huperzia_cox3_i171g2_379535_380262 ........G..A......T.....-.G..................- [728]

Pleurozia_cox3_i171 CG...CG.........TGTGTT..--.A.ACCC............C [903]

Marchantia_cox3i171 CG...CG.........TGTGTT..--.A.ACCC............C [907]

Treubia_cox3_i171 CG...CG.........TGTGTT..--.A.ACCC............C [907]

;

END;

**rps10i235**

**(several related introns located in other genes are also included.)**

#NEXUS

[MacClade 4.05 registered to Yin-Long Qiu Lab, EEB, University of Michigan]

BEGIN DATA;

DIMENSIONS NTAX=9 NCHAR=2942;

FORMAT DATATYPE=DNA MISSING=? GAP=- MATCHCHAR=. INTERLEAVE ;

MATRIX

[ 10 20 30 40 50]

[ . . . . .]

Huperzia_rps10i235 GTGCGACTCGAAGGACATAA---GACAATGCGTATAGCCCTT-------- [39]

Huperzia_rps10psi235 .........T.G........---...................-------- [39]

Cycas_rps10i235 ....................---...TT.TT.GTC..G..CA-------- [39]

Vitis_rps10i235 ..........G.........---...TTCTT.GTC.AG..AA-------- [39]

Megaceros_nad9i246 ........T..G........---...C..............A-------- [39]

Huperzia_squarrosa_nad5i392 ....................---A...............----------- [36]

Huperzia_selago_nad5_intron ........T..G........TGAA...............----------- [39]

Huperzia_squarrosa_nad5i1242 ........T..G........TGAA...............----------- [39]

Pleurozia_rrn26i827 ........T.........T.---........AA..G.....GTGGGTGGG [47]

[ 60 70 80 90 100]

[ . . . . .]

Huperzia_rps10i235 --------------------------------------G---AG---TTA [45]

Huperzia_rps10psi235 --------------------------------------.TTGGT---... [48]

Cycas_rps10i235 -------------------------------------A.ATAGT----C. [48]

Vitis_rps10i235 -------------------------------------------------. [40]

Megaceros_nad9i246 --------------------------------------.--GGC----.. [45]

Huperzia_squarrosa_nad5i392 --------------------------------------A--GGC----.. [42]

Huperzia_selago_nad5_intron --------------------------------------A--GGCGG-C.. [48]

Huperzia_squarrosa_nad5i1242 --------------------------------------A--GGC----.. [45]

Pleurozia_rrn26i827 GCGACGCCCGGACCTCTTCCCGATCCGGAGGACCGGGG.GGGGCAACCC. [97]

[ 110 120 130 140 150]

[ . . . . .]

Huperzia_rps10i235 ----TA--TCG-CCAT--CCCAGCCGACGGGATACTCCTACTCC----AC [82]

Huperzia_rps10psi235 ----.C--...-....--.........G..T..G........T.CTTC.. [89]

Cycas_rps10i235 A---..AT.A.AT...TG...............G.......C..----.. [91]

Vitis_rps10i235 -----.---A.---..T-----......A....G.......C..----.. [70]

Megaceros_nad9i246 ----..GG.T.---..--T..............GT......CT.----.. [82]

Huperzia_squarrosa_nad5i392 ----..--.T.-....--...............G.......CT.----.. [79]

Huperzia_selago_nad5_intron ----..--.T.-....--.........G.....G.......CT.----.T [85]

Huperzia_squarrosa_nad5i1242 ----..--.T.-....--.........G.....G.......CT.----.T [82]

Pleurozia_rrn26i827 GGGC..--CT.-....--...............G........T.----.. [138]

[ 160 170 180 190 200]

[ . . . . .]

Huperzia_rps10i235 CATGC---CTTCCTGG--------------CAACGGAAGG---------- [105]

Huperzia_rps10psi235 ..GT.---G.......--------------T...T.G.A.---------- [112]

Cycas_rps10i235 .....---GC......CCCTTCACCTTATAT..GA.GG..GGGAAGGGGG [138]

Vitis_rps10i235 .....CTGGCC.T.TACCTTACCTTAAGAAG..A.AGG..G--------- [111]

Megaceros_nad9i246 .....---GC....------------------....GGA.A--------- [102]

Huperzia_squarrosa_nad5i392 .....---G.......--------------......G.A.---------- [102]

Huperzia_selago_nad5_intron .....---G.......--------------T.....G...---------- [108]

Huperzia_squarrosa_nad5i1242 .....---G.......--------------T.....G...---------- [105]

Pleurozia_rrn26i827 .....---.C...C..--------------T.....GT..---------- [161]

[ 210 220 230 240 250]

[ . . . . .]

Huperzia_rps10i235 GTATG-AAGCGTGGGAAACAATTACATAAATGTATGATA-CAGCATATAC [153]

Huperzia_rps10psi235 ..C..G...T....A.....T.....G..G.T.......-G......C.. [161]

Cycas_rps10i235 .....-................GCG.G..G.........A.....-.... [186]

Vitis_rps10i235 .....-..............T.GCA.G..G.........-..A..GG..- [158]

Megaceros_nad9i246 .....-...T...A.GG.....GTA.G..G.........-.........- [149]

Huperzia_squarrosa_nad5i392 ..T..-.......A.G..........G..G....GA...-.......C.. [150]

Huperzia_selago_nad5_intron .....-.........G..........G..G...T.....-...T...C.. [156]

Huperzia_squarrosa_nad5i1242 ..C..-.........G..........G..G...T.....-...T...C.. [153]

Pleurozia_rrn26i827 .....-.........G......GTA.G..G......T..-.........- [208]

[ 260 270 280 290 300]

[ . . . . .]

Huperzia_rps10i235 ACTTGCTAGTAGGAGTGGC---AAGACTAT----TGATCAACGTAAGTGA [196]

Huperzia_rps10psi235 .TC......---.......---........----.........CT..... [201]

Cycas_rps10i235 -.C...CG.---.......---GGC.A..CTA-T.........CG..... [228]

Vitis_rps10i235 ..C.-TA..---.......GGC..A...C.TGAT.........CG..... [204]

Megaceros_nad9i246 ..C......---....A..---........---T................ [190]

Huperzia_squarrosa_nad5i392 ..C......---.G.....---....T...---T-......T.CG..... [190]

Huperzia_selago_nad5_intron .TC......---.......---G..GA..CAA-T.........CT..... [199]

Huperzia_squarrosa_nad5i1242 .TC......---.......---G..GA..CAA-T.........CT..... [196]

Pleurozia_rrn26i827 ..C......---.......---.......CG----........C...... [248]

[ 310 320 330 340 350]

[ . . . . .]

Huperzia_rps10i235 ACTGTGCGACGACGCTTCGTAAA--AC---CGCGTCAGA----GAGCC-T [236]

Huperzia_rps10psi235 .......................--.G---...AC....CAGA....TG. [246]

Cycas_rps10i235 .......TTA......C......--..---...AC.GC------.T.T-- [265]

Vitis_rps10i235 .......TTA.............--..---...AC.-------..T.T-- [240]

Megaceros_nad9i246 ....................C..--.T---...CG.--------.C..-. [226]

Huperzia_squarrosa_nad5i392 .......................--..---...AC.G..----..T..-C [230]

Huperzia_selago_nad5_intron .......................--.GAAG...AC.G.-----.....-. [241]

Huperzia_squarrosa_nad5i1242 .......................--.GAAG...AC.G.-----.....-. [238]

Pleurozia_rrn26i827 .............A.........TA..-----.AC.TAC----....TT. [289]

[ 360 370 380 390 400]

[ . . . . .]

Huperzia_rps10i235 ATTGAGTA----------GGGCCG--------ATAGG-GACGAAAAATGA [267]

Huperzia_rps10psi235 .A.T-...----------......CCGGCTCC..T..C.G....G..... [285]

Cycas_rps10i235 .CGAGA-----------G.A..T.T-------..T.AT..GT.GGC---- [293]

Vitis_rps10i235 .CGAGA-----------G.A..T.--------..G.AT..GT.GGCT.CC [271]

Megaceros_nad9i246 .C----------------.A..T.T-------..T.ACT.---------- [243]

Huperzia_squarrosa_nad5i392 ...T.A..AAGAAATAAG....T.T-------..T.AA..T--------- [264]

Huperzia_selago_nad5_intron .A.T...GGGGGCGAAAG.CA.TAT-------G.T..C.G.......AT. [284]

Huperzia_squarrosa_nad5i1242 .A.T...GGGGGCGAAAG.CA.TAT-------G.T..C.G.T.....AT. [281]

Pleurozia_rrn26i827 ..G.TA..----------.....C--------....TCATT--------- [312]

[ 410 420 430 440 450]

[ . . . . .]

Huperzia_rps10i235 GCC----AACGAAGCAATGTGATTTGGGACACGATGGGAGTTTGCGTGCC [313]

Huperzia_rps10psi235 ...----...A.............G.....G.....TTTAG......... [331]

Cycas_rps10i235 ---------.T.C.G........C.......................... [334]

Vitis_rps10i235 C.TTTCG.TTC.C.G........C.......................... [321]

Megaceros_nad9i246 -------------.GGCC.................CA..AGG........ [280]

Huperzia_squarrosa_nad5i392 -----------.G.GGG......C..T......T..TTTC.GC....... [303]

Huperzia_selago_nad5_intron ...----...A.........................TTGAG..A...... [330]

Huperzia_squarrosa_nad5i1242 ...----...A.........................TTTAG..A...... [327]

Pleurozia_rrn26i827 ---------TCGT.GG........C.............CC.......... [353]

[ 460 470 480 490 500]

[ . . . . .]

Huperzia_rps10i235 TCGATCGGCAAAT-ATCACCGGAGTATAGCACAAGATCGCCAAATCTT-- [360]

Huperzia_rps10psi235 .............-.......A...GA..................T..CT [380]

Cycas_rps10i235 .....A......AG...........................TTT....GC [384]

Vitis_rps10i235 ...G.A..A..GAG........................A..TTT....GC [371]

Megaceros_nad9i246 C..T........GC............G......G.......CG.....-- [328]

Huperzia_squarrosa_nad5i392 .............G............A.A..A.G..............-- [351]

Huperzia_selago_nad5_intron .............-..................................-- [377]

Huperzia_squarrosa_nad5i1242 .............-..................................-- [374]

Pleurozia_rrn26i827 .........G..AG.....T.............G.....TG.......-- [401]

[ 510 520 530 540 550]

[ . . . . .]

Huperzia_rps10i235 ---GCCAGAGAGTCAAACCTGTCAACAAGGTAAACCCAAAGGGCTCCCCG [407]

Huperzia_rps10psi235 TTG.............-......CG.T......-...C.C.......... [428]

Cycas_rps10i235 T--....T........-.....A...G............T...AA..GT. [431]

Vitis_rps10i235 T--....TG.G...G.-.....G................T...AA..--- [415]

Megaceros_nad9i246 ---...CT.AG.....-AA.......G............T........TC [374]

Huperzia_squarrosa_nad5i392 ---..A.CG.......-................T.....T.......... [397]

Huperzia_selago_nad5_intron ---.............-................C.....T.......... [423]

Huperzia_squarrosa_nad5i1242 ---.............-................C.....T.......... [420]

Pleurozia_rrn26i827 ---.TA.T........-.G........G...........C........T- [446]

[ 560 570 580 590 600]

[ . . . . .]

Huperzia_rps10i235 GCCTC---GGGAGGTTTGGTCGTGAGATCAAATAGTGCCCAGTGGGCAGA [454]

Huperzia_rps10psi235 .G.AA---..........................CC.............. [475]

Cycas_rps10i235 AAAAAAAC......................G...CC..--.T........ [479]

Vitis_rps10i235 --------AAA.AA------..G...----GG..CC..--.T........ [445]

Megaceros_nad9i246 .G--------...A..G......A...C..G...CC............A. [416]

Huperzia_squarrosa_nad5i392 .G.AA---............T....T..T.C...CC...T.......... [444]

Huperzia_selago_nad5_intron .G.AA---............T.............CC...T.......... [470]

Huperzia_squarrosa_nad5i1242 .G.AA---............T.............CC...T.......... [467]

Pleurozia_rrn26i827 --T..---....................T.G...CC.............. [491]

[ 610 620 630 640 650]

[ . . . . .]

Huperzia_rps10i235 AGA---CGATCCAAAAAG-CGAAGGCTCAACCTGGTTCAG---------- [490]

Huperzia_rps10psi235 ...AGA........G...G....A..C.GG--AAAAA..A---------- [513]

Cycas_rps10i235 ...---............-...G......C..G.TGAG.----------- [514]

Vitis_rps10i235 ...---............-..........C..A.C.GA..GAAG------ [485]

Megaceros_nad9i246 ...---...CT..G....-.....A....C..GTAA-------------- [448]

Huperzia_squarrosa_nad5i392 ...---....T..G....-......T...C..G...-------------- [476]

Huperzia_selago_nad5_intron ...---.......GG...-......T...C.TG.AAGA.TATAAAGGTGA [516]

Huperzia_squarrosa_nad5i1242 ...---.......GG...-......T...C.TG.AAGA.TATAAAGGTGA [513]

Pleurozia_rrn26i827 ...---....A.....G.-...G....T.C.TGTCA-------------- [523]

[ 660 670 680 690 700]

[ . . . . .]

Huperzia_rps10i235 --------------------------------------CAGGTGA----- [497]

Huperzia_rps10psi235 --------------------------------------A...CT------ [519]

Cycas_rps10i235 --------------------------------------T...C..C---- [522]

Vitis_rps10i235 --------------------------------GAGCTTTG.C.T.ACGCC [503]

Megaceros_nad9i246 --------------------------------------TGA....----- [455]

Huperzia_squarrosa_nad5i392 --------------------------------------TG...A.----- [483]

Huperzia_selago_nad5_intron GAAGGCAAAAAGTAGCCGACCAGTGCTGACTTGAATCTTG.....CGAAA [566]

Huperzia_squarrosa_nad5i1242 GAAGGCAAAAAGTAGCCGACCAGTGCTGACTTGAATCTTG.....CGAAA [563]

Pleurozia_rrn26i827 --------------------------------------.....T.----- [530]

[ 710 720 730 740 750]

[ . . . . .]

Huperzia_rps10i235 -------------------------------------------------- [497]

Huperzia_rps10psi235 -------------------------------------------------- [519]

Cycas_rps10i235 -------------------------------------------------- [522]

Vitis_rps10i235 CTT----------------------------------------------- [506]

Megaceros_nad9i246 -------------------------------------------------- [455]

Huperzia_squarrosa_nad5i392 -------------------------------------------------- [483]

Huperzia_selago_nad5_intron CACTATTAGGGGCCACTCTTTCTTATTGACCACGTATATAAAAAGAGGAG [616]

Huperzia_squarrosa_nad5i1242 CACTATTAGGGGCCACTCTTTCTTATTGACCACGTATATAAAAAGAGGAG [613]

Pleurozia_rrn26i827 -------------------------------------------------- [530]

[ 760 770 780 790 800]

[ . . . . .]

Huperzia_rps10i235 -------------------------------------------------- [497]

Huperzia_rps10psi235 -------------------------------------------------- [519]

Cycas_rps10i235 -------------------------------------------------- [522]

Vitis_rps10i235 -------------------------------------------------- [506]

Megaceros_nad9i246 -------------------------------------------------- [455]

Huperzia_squarrosa_nad5i392 -------------------------------------------------- [483]

Huperzia_selago_nad5_intron GAGCGGTCAAGACTGCAAGCAGTGCGCGCGTAAAAATTGGTCAATCGCAA [666]

Huperzia_squarrosa_nad5i1242 GAGCGGTCAAGACTGCAAGCAGTGCGCGCGTAAAAATTGGTCAATCGCAA [663]

Pleurozia_rrn26i827 -------------------------------------------------- [530]

[ 810 820 830 840 850]

[ . . . . .]

Huperzia_rps10i235 ------------------------GAT--------------------AGG [503]

Huperzia_rps10psi235 ------------------------...--------------------.A. [525]

Cycas_rps10i235 ------------------------..A-------------------A... [529]

Vitis_rps10i235 -------------------------..TATTATTCA----------A... [521]

Megaceros_nad9i246 ------------------------...--------------------... [461]

Huperzia_squarrosa_nad5i392 ------------------------...--------------------... [489]

Huperzia_selago_nad5_intron AGTCGCGACAAGAAAAGGTTCGAA...CTCTACTAAGAGATCTAAAA..A [716]

Huperzia_squarrosa_nad5i1242 AGTCGCGACAAGAAAAGGTTCGAA...CTCTACTAAGAGATTTTAAA..A [713]

Pleurozia_rrn26i827 ------------------------A..--------------------... [536]

[ 860 870 880 890 900]

[ . . . . .]

Huperzia_rps10i235 C-G----------------------------------------------- [505]

Huperzia_rps10psi235 TA.----------------------------------------------- [528]

Cycas_rps10i235 AA.AA--------------------------------------------- [534]

Vitis_rps10i235 GGAAAGGGTGGT-------------------------------------- [533]

Megaceros_nad9i246 .GA----------------------------------------------- [464]

Huperzia_squarrosa_nad5i392 .AACATATCAAGAATATGATACGTAT------------------------ [515]

Huperzia_selago_nad5_intron .TAATAAAAAAAAAGATATTATGAATTGGAAAGAAATAAATTGGAAACAA [766]

Huperzia_squarrosa_nad5i1242 .GAATAAAAAAAAAGATATTATGAATTGGAAAGAAATAAATTGGAAACAA [763]

Pleurozia_rrn26i827 .AA----------------------------------------------- [539]

[ 910 920 930 940 950]

[ . . . . .]

Huperzia_rps10i235 -------------------------------------------------- [505]

Huperzia_rps10psi235 -------------------------------------------------- [528]

Cycas_rps10i235 -------------------------------------------------- [534]

Vitis_rps10i235 -------------------------------------------------- [533]

Megaceros_nad9i246 -------------------------------------------------- [464]

Huperzia_squarrosa_nad5i392 -----------------------------------------------ATG [518]

Huperzia_selago_nad5_intron GCAGCGAAGCTACTGCTACTCTTGAGTGGCAGAACGCTTTAGTGAGAAAG [816]

Huperzia_squarrosa_nad5i1242 GCAGCGAAGCTACTGCTACTCTTGAGTGGCAGAACGCTTTAGTGAGAAAG [813]

Pleurozia_rrn26i827 -------------------------------------------------- [539]

[ 960 970 980 990 1000]

[ . . . . .]

Huperzia_rps10i235 -ACA-AT------------------------------------------- [510]

Huperzia_rps10psi235 -.A.-..------------------------------------------- [533]

Cycas_rps10i235 -.T.G..------------------------------------------- [540]

Vitis_rps10i235 CGT.G..CAG---------------------------------------- [543]

Megaceros_nad9i246 --T.-..------------------------------------------- [468]

Huperzia_squarrosa_nad5i392 T.T.-..GTATATTATA--------------------------------- [534]

Huperzia_selago_nad5_intron T.T.-..CAGTAGATTAGCTTCTAATCCACCGCGGACCCAAAACAAAATC [865]

Huperzia_squarrosa_nad5i1242 T.T.-..CAGTAGATTAGCTTCTAATCCACCGCGGATCCAAAA-AAAATC [861]

Pleurozia_rrn26i827 --..T..G------------------------------------------ [545]

[ 1010 1020 1030 1040 1050]

[ . . . . .]

Huperzia_rps10i235 -------------------------------------------------- [510]

Huperzia_rps10psi235 -------------------------------------------------- [533]

Cycas_rps10i235 -------------------------------------------------- [540]

Vitis_rps10i235 -------------------------------------------------- [543]

Megaceros_nad9i246 -------------------------------------------------- [468]

Huperzia_squarrosa_nad5i392 -------------------------------------------------- [534]

Huperzia_selago_nad5_intron ATTCATTCACTGAGCGCACGAGCCCTAGCGGTTCGAAGGATGACAACTCA [915]

Huperzia_squarrosa_nad5i1242 ATTCATTCACTGAGCGCACGAGCCCTAGCGGTTCGAAGGATGACAACTCA [911]

Pleurozia_rrn26i827 -------------------------------------------------- [545]

[ 1060 1070 1080 1090 1100]

[ . . . . .]

Huperzia_rps10i235 -------------------------------------------------- [510]

Huperzia_rps10psi235 -------------------------------------------------- [533]

Cycas_rps10i235 -------------------------------------------------- [540]

Vitis_rps10i235 -------------------------------------------------- [543]

Megaceros_nad9i246 -------------------------------------------------- [468]

Huperzia_squarrosa_nad5i392 -------TAACGTATATGTAAAGTGGGC---------------------- [555]

Huperzia_selago_nad5_intron CTATGGTCGAAACACACCTGGAGTAGACAGAGTTGTGTGGAAGACTTATC [965]

Huperzia_squarrosa_nad5i1242 CTATGGTCGAAACACACCTGGAGTAGACAGAGTTGTGTGGAAGACTTATC [961]

Pleurozia_rrn26i827 -------------------------------------------------- [545]

[ 1110 1120 1130 1140 1150]

[ . . . . .]

Huperzia_rps10i235 -------------------------------------------------- [510]

Huperzia_rps10psi235 -------------------------------------------------- [533]

Cycas_rps10i235 -------------------------------------------------- [540]

Vitis_rps10i235 -------------------------------------------------- [543]

Megaceros_nad9i246 -------------------------------------------------- [468]

Huperzia_squarrosa_nad5i392 --------------------------------------CATGAAAATGCC [567]

Huperzia_selago_nad5_intron GAAAGAAAAAGAGAAGCCTTACAATCCTTAACCCACCACATGAAAAATCC [1015]

Huperzia_squarrosa_nad5i1242 GAAAGAAAAAGAGAAGCCTTACAATCCTTAACCCACCACATGAAAAATCC [1011]

Pleurozia_rrn26i827 -------------------------------------------------- [545]

[ 1160 1170 1180 1190 1200]

[ . . . . .]

Huperzia_rps10i235 -------------------------------------------------- [510]

Huperzia_rps10psi235 -------------------------------------------------- [533]

Cycas_rps10i235 -------------------------------------------------- [540]

Vitis_rps10i235 -------------------------------------------------- [543]

Megaceros_nad9i246 -------------------------------------------------- [468]

Huperzia_squarrosa_nad5i392 -------------------------------------------------- [567]

Huperzia_selago_nad5_intron TCTWCCTTACATGCCCCACCACTTCGAGTTCAGATTACGAAACCGGAGAG [1065]

Huperzia_squarrosa_nad5i1242 TCTTCCTTACATGCCCCACCACTTCGAGTTCAGATTACGAAACCGGAGAG [1061]

Pleurozia_rrn26i827 -------------------------------------------------- [545]

[ 1210 1220 1230 1240 1250]

[ . . . . .]

Huperzia_rps10i235 -------------------------------------------------- [510]

Huperzia_rps10psi235 -------------------------------------------------- [533]

Cycas_rps10i235 -------------------------------------------------- [540]

Vitis_rps10i235 -------------------------------------------------- [543]

Megaceros_nad9i246 -------------------------------------------------- [468]

Huperzia_squarrosa_nad5i392 -------------------------------------------------- [567]

Huperzia_selago_nad5_intron TAAATAAATCAGGCCCCGTAGACCAACCATGATAGAGCAAGAAGGCAGGC [1115]

Huperzia_squarrosa_nad5i1242 TAAATAAATCAGGCCCCGTAGACCAACCATGATAGAGCAAGAAGGCAGGC [1111]

Pleurozia_rrn26i827 -------------------------------------------------- [545]

[ 1260 1270 1280 1290 1300]

[ . . . . .]

Huperzia_rps10i235 -------------------------------------------------- [510]

Huperzia_rps10psi235 -------------------------------------------------- [533]

Cycas_rps10i235 -------------------------------------------------- [540]

Vitis_rps10i235 -------------------------------------------------- [543]

Megaceros_nad9i246 -----------------TTG------------------------------ [471]

Huperzia_squarrosa_nad5i392 ----------AAGCGTTTTGGCCACGCTTTTTTTG----------GGGGC [597]

Huperzia_selago_nad5_intron TTTCTACTAGAAGGCCTTCGATCCTACTACAGTGGAATGTCAGTCGGGCC [1165]

Huperzia_squarrosa_nad5i1242 TTTCTACTAGAAGGCCTTCGATCCTACTACAGTGGAATGTCAGTCGGGCC [1161]

Pleurozia_rrn26i827 -------------------------------------------------- [545]

[ 1310 1320 1330 1340 1350]

[ . . . . .]

Huperzia_rps10i235 -------------------------------------------------- [510]

Huperzia_rps10psi235 -------------------------------------------------- [533]

Cycas_rps10i235 -------------------------------------------------- [540]

Vitis_rps10i235 -------------------------------------------------- [543]

Megaceros_nad9i246 -------------------------------------------------- [471]

Huperzia_squarrosa_nad5i392 G-------------------------------TTTTTCTTCACAAAAAGA [616]

Huperzia_selago_nad5_intron G------GCTAGTAGGGGCAACGCCTGCTTTTTATTGCTTTACGAAAAAA [1209]

Huperzia_squarrosa_nad5i1242 GCCGCCGGCTAGTTGGGGCAACGCCTGCTTTTTATTGCTTTACGAAAAAA [1211]

Pleurozia_rrn26i827 -------------------------------------------------- [545]

[ 1360 1370 1380 1390 1400]

[ . . . . .]

Huperzia_rps10i235 -------------------------------------------------- [510]

Huperzia_rps10psi235 -------------------------------------------------- [533]

Cycas_rps10i235 -------------------------------------------------- [540]

Vitis_rps10i235 -------------------------------------------------- [543]

Megaceros_nad9i246 -------------------------------------------------- [471]

Huperzia_squarrosa_nad5i392 -------------------------------------------------- [616]

Huperzia_selago_nad5_intron AAAGAAGGGTTCCGGAAAGGGCGCTCCCACAATA----CGCTATCTCGTA [1255]

Huperzia_squarrosa_nad5i1242 AAATAAGGGTTCCGGAAAGGGCGCTCCCACAATAAATACGCTATCTCGTA [1261]

Pleurozia_rrn26i827 -------------------------------------------------- [545]

[ 1410 1420 1430 1440 1450]

[ . . . . .]

Huperzia_rps10i235 -------------------------TCG---CCA---------------- [516]

Huperzia_rps10psi235 -------------------------...---.------------------ [537]

Cycas_rps10i235 -------------------------CTTTTT..GC--------------- [550]

Vitis_rps10i235 ------------------------GCT.ATCTTTGATC------------ [557]

Megaceros_nad9i246 -------------------------...AC-..G---------------- [479]

Huperzia_squarrosa_nad5i392 -------------------------...AC-...CTACTTTT-------- [632]

Huperzia_selago_nad5_intron CCTGCTGAGTAAATGCCTACCTCCA..AAC-..GGATGTCTTGAATGGAT [1304]

Huperzia_squarrosa_nad5i1242 CCTGCTGAGTAAATGCCTCCCTCCA..AAC-..GGATGTCTTGAATGGAT [1310]

Pleurozia_rrn26i827 -------------------------C..ACA..----------------- [553]

[ 1460 1470 1480 1490 1500]

[ . . . . .]

Huperzia_rps10i235 -------------------------------------------------- [516]

Huperzia_rps10psi235 -------------------------------------------------- [537]

Cycas_rps10i235 -------------------------------------------------- [550]

Vitis_rps10i235 -------------------------------------------------- [557]

Megaceros_nad9i246 -------------------------------------------------- [479]

Huperzia_squarrosa_nad5i392 -------------------------------------------------- [632]

Huperzia_selago_nad5_intron CTTCGATGCCGATGTAAAGGTGGCTT---TTTTTACCCCACTCTGTTTGC [1351]

Huperzia_squarrosa_nad5i1242 CTTCGATGCCGATGTAAAGGTGGCTTCTTTTTTTACCCCACTCTGTTTGC [1360]

Pleurozia_rrn26i827 -------------------------------------------------- [553]

[ 1510 1520 1530 1540 1550]

[ . . . . .]

Huperzia_rps10i235 ---------AGTG----------------------------CTGA----- [524]

Huperzia_rps10psi235 -----------..----------------------------....----- [543]

Cycas_rps10i235 --------CG...----------------------------....----- [559]

Vitis_rps10i235 ------AGA....---------------CTCGTAGATCAGG....TT--- [583]

Megaceros_nad9i246 -----AAAC....----------------------------....----- [491]

Huperzia_squarrosa_nad5i392 -AACCGAAC....----------------------------....----- [648]

Huperzia_selago_nad5_intron GAAGCGAAC.A..GGATGCTTCGGCAAACATAAGCCACCCC.C..A---- [1397]

Huperzia_squarrosa_nad5i1242 GAAGCGAAC.A..GGATGCTTCGGCAAACATAAGCCACCCC.C..ACGAA [1410]

Pleurozia_rrn26i827 --------C....----------------------------....----- [562]

[ 1560 1570 1580 1590 1600]

[ . . . . .]

Huperzia_rps10i235 -------------------CTCGA-----------------ATCTTGA-- [536]

Huperzia_rps10psi235 -------------------...A.-----------------.......-- [555]

Cycas_rps10i235 -------------------..T.G-----------------....C.G-- [571]

Vitis_rps10i235 --GTAAATCAGGGTGCTCC.CTAC----------------A.....AGGC [615]

Megaceros_nad9i246 -------------------.....-----------------......G-- [503]

Huperzia_squarrosa_nad5i392 -------------------..T..-----------------......G-- [660]

Huperzia_selago_nad5_intron AAAGCCGGATAAGATCACA..TA.GGAGGCACAAAGATGAA......GAA [1447]

Huperzia_squarrosa_nad5i1242 AAAGCCGGATAAGATCACA..TA.GGAGGCACAAAGATGAA......GAA [1460]

Pleurozia_rrn26i827 -------------------..T.T-----------------...G..G-- [574]

[ 1610 1620 1630 1640 1650]

[ . . . . .]

Huperzia_rps10i235 ----------GTGACGA----AA---CACTATTA---CCAGG-------- [558]

Huperzia_rps10psi235 ----------.....A.----..---........TTA.....-------- [580]

Cycas_rps10i235 ----------.......GAGA..---........----TG..-------- [596]

Vitis_rps10i235 TTAAGCAAGCAG.CT..TTGT..ATC.G..T..TCTC.G..A-------- [657]

Megaceros_nad9i246 ----------.......----..---.....AGG----A...-------- [524]

Huperzia_squarrosa_nad5i392 ----------.......----..---........----.C..-------- [681]

Huperzia_selago_nad5_intron ATGGCTCCGA.C..G..----GG---T.TC..GG----A...ATACAACC [1486]

Huperzia_squarrosa_nad5i1242 ATGGCTCCGA.C..G..----GG---T.TC..GG----A...ATACAACC [1499]

Pleurozia_rrn26i827 ----------.....A.----..---.G------------..-------- [587]

[ 1660 1670 1680 1690 1700]

[ . . . . .]

Huperzia_rps10i235 --------------------------CTACTCAATTT------------- [569]

Huperzia_rps10psi235 --------------------------...........------------- [591]

Cycas_rps10i235 --------------------------.CGGG.GGCCC------------- [607]

Vitis_rps10i235 -----------------------GGG...TCTT.C.AAA----------- [673]

Megaceros_nad9i246 --------------------------TC...---CA.------------- [532]

Huperzia_squarrosa_nad5i392 --------------------------TC...TTTC..------------- [692]

Huperzia_selago_nad5_intron TCCCGCAGCCTATAGGGGCCAGGGCATC.T.TTCC..CGCCTATGCTGCT [1536]

Huperzia_squarrosa_nad5i1242 TCCCGCAGCCTAGAGGGGCCAGGGCATC.T.TTCC..CGCCTATGCTGCT [1549]

Pleurozia_rrn26i827 -------------------------------------------------- [587]

[ 1710 1720 1730 1740 1750]

[ . . . . .]

Huperzia_rps10i235 ------------------AATTG---ACCACGCG----------AAAGC- [587]

Huperzia_rps10psi235 ------------------...CA---........----------.....- [609]

Cycas_rps10i235 ------------------.C...---G.......CGAATC-----...TA [631]

Vitis_rps10i235 -----------------A.G...-AA.GGG..T.AGATAGGCGAC.G.TA [705]

Megaceros_nad9i246 -------------------....---.......A----------.....A [550]

Huperzia_squarrosa_nad5i392 -------------------....---.A...A..----------....TT [710]

Huperzia_selago_nad5_intron CTGCTGGCCAACTAGTCGG....GAA.G.....AGACCCTACCG...A.T [1586]

Huperzia_squarrosa_nad5i1242 CTGCTGGCCAACTAGTCGG....GAA.G.....AGACCCTACCG...A.T [1599]

Pleurozia_rrn26i827 -------------------------------------------------- [587]

[ 1760 1770 1780 1790 1800]

[ . . . . .]

Huperzia_rps10i235 ---AG--------------------------------------------- [589]

Huperzia_rps10psi235 ---..--------------------------------------------- [611]

Cycas_rps10i235 CGG..--------------------------------------------- [636]

Vitis_rps10i235 GCTT.--------------------------------------------- [710]

Megaceros_nad9i246 CAG..--------------------------------------------- [555]

Huperzia_squarrosa_nad5i392 TGG..--------------------------------------------- [715]

Huperzia_selago_nad5_intron CGA..GTTTAGTCGATCTAAGTGGAGGAGGCCACCTTACGGGATCCTTGT [1636]

Huperzia_squarrosa_nad5i1242 CGA..GTTCAGTCGATCTAAGTGGAGGAGGCCACCTTACGGGATCCTTGT [1649]

Pleurozia_rrn26i827 -------------------------------------------------- [587]

[ 1810 1820 1830 1840 1850]

[ . . . . .]

Huperzia_rps10i235 --------------------------CGGTCAATACCG------------ [601]

Huperzia_rps10psi235 --------------------------............------------ [623]

Cycas_rps10i235 --------------------------.....G.GC...------------ [648]

Vitis_rps10i235 --------------------------.TTC...GC.GATA---------- [724]

Megaceros_nad9i246 --------------------------...C.G.GC.T.------------ [567]

Huperzia_squarrosa_nad5i392 --------------------------.......G....------------ [727]

Huperzia_selago_nad5_intron GCCCAGATTGACGATTTATTGATTCT.T.C...G...AGAAACGGAAAAC [1686]

Huperzia_squarrosa_nad5i1242 GCCCAGATTGACGATTTATTGAGTCT.T.C...G...AGAAACGGAAAAC [1699]

Pleurozia_rrn26i827 -------------------------------..CG.T.------------ [594]

[ 1860 1870 1880 1890 1900]

[ . . . . .]

Huperzia_rps10i235 -------------------------------------------------- [601]

Huperzia_rps10psi235 -------------------------------------------------- [623]

Cycas_rps10i235 -------------------------------------------------- [648]

Vitis_rps10i235 -------------------------------------------------- [724]

Megaceros_nad9i246 -------------------------------------------------- [567]

Huperzia_squarrosa_nad5i392 -------------------------------------------------- [727]

Huperzia_selago_nad5_intron AACCTAGTCCCTGTGGTATCCGTCCAAGAGTTAAAAAAGGTTCGAAGAAT [1736]

Huperzia_squarrosa_nad5i1242 ACCCTAGTCCCTGTGGTATCCGTCCAAGAGTTAAAAAAGGTTCGAAGAAT [1749]

Pleurozia_rrn26i827 -------------------------------------------------- [594]

[ 1910 1920 1930 1940 1950]

[ . . . . .]

Huperzia_rps10i235 -------------------------------------------------- [601]

Huperzia_rps10psi235 -------------------------------------------------- [623]

Cycas_rps10i235 -------------------------------------------------- [648]

Vitis_rps10i235 -------------------------------------------------- [724]

Megaceros_nad9i246 -------------------------------------------------- [567]

Huperzia_squarrosa_nad5i392 -------------------------------------------------- [727]

Huperzia_selago_nad5_intron TATTCTGCGGCTAATTCTAGATAAAGGTGCTGTCCGGAACGTAAAATAAG [1786]

Huperzia_squarrosa_nad5i1242 TATTCTGCGGCTAATTATAGATAAAGGTGCTGTCCGGAACGTAAAATAAG [1799]

Pleurozia_rrn26i827 -------------------------------------------------- [594]

[ 1960 1970 1980 1990 2000]

[ . . . . .]

Huperzia_rps10i235 -------------------------------------------------- [601]

Huperzia_rps10psi235 -------------------------------------------------- [623]

Cycas_rps10i235 -------------------------------------------------- [648]

Vitis_rps10i235 -------------------------------------------------- [724]

Megaceros_nad9i246 -------------------------------------------------- [567]

Huperzia_squarrosa_nad5i392 -------------------------------------------------- [727]

Huperzia_selago_nad5_intron GATTCTTCTTTTATGCGCTTGGATCCCACATCAAGAAAGTGGAGCACAAA [1836]

Huperzia_squarrosa_nad5i1242 GATTCTTCTTTTATGCGCTTGGATCCCACATCAAGAAAGTGGAGCACAAA [1849]

Pleurozia_rrn26i827 -------------------------------------------------- [594]

[ 2010 2020 2030 2040 2050]

[ . . . . .]

Huperzia_rps10i235 -------------------------------------------------- [601]

Huperzia_rps10psi235 -------------------------------------------------- [623]

Cycas_rps10i235 -------------------------------------------------- [648]

Vitis_rps10i235 -------------------------------------------------- [724]

Megaceros_nad9i246 -------------------------------------------------- [567]

Huperzia_squarrosa_nad5i392 -------------------------------------------------- [727]

Huperzia_selago_nad5_intron CCTCACCTATTATCGTAAGGAGAGGCTAGAGATAAATCAGAAATACTAAT [1886]

Huperzia_squarrosa_nad5i1242 CCTCACCTATTATCGTAAGGAGAGGCTAGAGATAAATCATAAATACTCAT [1899]

Pleurozia_rrn26i827 -------------------------------------------------- [594]

[ 2060 2070 2080 2090 2100]

[ . . . . .]

Huperzia_rps10i235 -------------------------------------------------- [601]

Huperzia_rps10psi235 -------------------------------------------------- [623]

Cycas_rps10i235 -------------------------------------------------- [648]

Vitis_rps10i235 -------------------------------------------------G [725]

Megaceros_nad9i246 -------------------------------------------------- [567]

Huperzia_squarrosa_nad5i392 -------------------------------------------------- [727]

Huperzia_selago_nad5_intron GAAGGTAGTACCTAGGACGGCAAAAATTAAATCCTTAAATAAAGACAAGG [1936]

Huperzia_squarrosa_nad5i1242 GAAGGTAGTACCTAGGACGGCAAAAATGAAATCCTTAAAGAAAGACAAGG [1949]

Pleurozia_rrn26i827 -------------------------------------------------- [594]

[ 2110 2120 2130 2140 2150]

[ . . . . .]

Huperzia_rps10i235 --CAAGCA--------------GTGTGCGG-------------------- [615]

Huperzia_rps10psi235 --......--------------........-------------------- [637]

Cycas_rps10i235 --......--------------...CTTA--------------------- [661]

Vitis_rps10i235 GAT.G..G--------------.C.GC.C.G------------------- [742]

Megaceros_nad9i246 --......--------------...C..---------------------- [579]

Huperzia_squarrosa_nad5i392 --.....T--------------...C..---------------------- [739]

Huperzia_selago_nad5_intron AT......CATTGTGCGTTAGA...C..AAAATGGCCCAAGCCCCCAAAA [1986]

Huperzia_squarrosa_nad5i1242 AT......CATTGTGCGTTAGA...C..AAAATGGCCCAAGCCCAAAAAA [1999]

Pleurozia_rrn26i827 -------------------------------------------------- [594]

[ 2160 2170 2180 2190 2200]

[ . . . . .]

Huperzia_rps10i235 -------------------------------------------------- [615]

Huperzia_rps10psi235 -------------------------------------------------- [637]

Cycas_rps10i235 -------------------------------------------------- [661]

Vitis_rps10i235 -------------------------------------------------- [742]

Megaceros_nad9i246 -------------------------------------------------- [579]

Huperzia_squarrosa_nad5i392 -------------------------------------------------- [739]

Huperzia_selago_nad5_intron AAAGGGACTTGACCCACTTCCCCGAGGATGTACTGTATATTTTTCCGGAA [2036]

Huperzia_squarrosa_nad5i1242 AAAGGGACTTGACCCACTTCCACGAGGATGTACTGTATATTTTTCCGGAA [2049]

Pleurozia_rrn26i827 -------------------------------------------------- [594]

[ 2210 2220 2230 2240 2250]

[ . . . . .]

Huperzia_rps10i235 --------------GTGG-------------------------------- [619]

Huperzia_rps10psi235 --------------....-------------------------------- [641]

Cycas_rps10i235 --------------..A.-------------------------------- [665]

Vitis_rps10i235 ------------GC..A.-------------------------------- [748]

Megaceros_nad9i246 --------------.C---------------------------------- [581]

Huperzia_squarrosa_nad5i392 --------------.CC.CTT----------------------------- [746]

Huperzia_selago_nad5_intron CCACGGCCTACTGT.CC.ATTGTTAGCGTATTACATCTGGACGAAATATT [2086]

Huperzia_squarrosa_nad5i1242 CCACGGCCTACTGT.CC.ATTGTTAGCGTATTACATCTGGACGAAATATT [2099]

Pleurozia_rrn26i827 -------------------------------------------------- [594]

[ 2260 2270 2280 2290 2300]

[ . . . . .]

Huperzia_rps10i235 -------------------------------------------ATATTGG [626]

Huperzia_rps10psi235 -------------------------------------------....... [648]

Cycas_rps10i235 -------------------------------------------..C.... [672]

Vitis_rps10i235 -------------------------------------------.AGCGA. [755]

Megaceros_nad9i246 -----------------------------------------------G.. [584]

Huperzia_squarrosa_nad5i392 ------------------------------------AAATTAA....G.. [760]

Huperzia_selago_nad5_intron TATTACTGGACGCAAGTGGGGAAGGCACCCCATAAGAACCGGCGA..G.. [2136]

Huperzia_squarrosa_nad5i1242 TATTACTGGACGCAAGTGGGGAAGGCACCCCATAAGAACCGGCGA..G.. [2149]

Pleurozia_rrn26i827 -----------------------------------------------GAA [597]

[ 2310 2320 2330 2340 2350]

[ . . . . .]

Huperzia_rps10i235 TCA--A-----TCGCA-------------------AAGTCG--------- [641]

Huperzia_rps10psi235 ...--.-----.....-------------------......--------- [663]

Cycas_rps10i235 ...--.-----.....-------------------.CA.T.--------- [687]

Vitis_rps10i235 ..GCC.GAAGC.TT.GGT-----------------.GC.T.--------- [779]

Megaceros_nad9i246 ...--.-----....G-------------------..A.T.--------- [599]

Huperzia_squarrosa_nad5i392 ...--.-----.T..--------------------..C.T.--------- [774]

Huperzia_selago_nad5_intron .T.--.AGTAT.TC.GTTCCTAAGTAAATTCGAAA..A.T.GAGAGCGAG [2184]

Huperzia_squarrosa_nad5i1242 .T.--.AGTAT.TC.GTTCCTAAGTAAATTCGAAA..A.T.GAGAGCAAG [2197]

Pleurozia_rrn26i827 C..--.-----------------------------....TT--------- [607]

[ 2360 2370 2380 2390 2400]

[ . . . . .]

Huperzia_rps10i235 -------------------------------------------------- [641]

Huperzia_rps10psi235 -------------------------------------------------- [663]

Cycas_rps10i235 -------------------------------------------------- [687]

Vitis_rps10i235 -------------------------------------------------- [779]

Megaceros_nad9i246 -------------------------------------------------- [599]

Huperzia_squarrosa_nad5i392 -------------------------------------------------- [774]

Huperzia_selago_nad5_intron AGCCAAAGGATGGAACGTAAGCTAAATCAAATTTCGATTTTTCACTCCAA [2234]

Huperzia_squarrosa_nad5i1242 AGCCAAAGGATGGAACGTAAGCTAAATCAAATTTCGATTTT-CACTCCAA [2246]

Pleurozia_rrn26i827 -------------------------------------------------- [607]

[ 2410 2420 2430 2440 2450]

[ . . . . .]

Huperzia_rps10i235 ----------------------------------------CGACATACGC [651]

Huperzia_rps10psi235 ----------------------------------------.A...----- [668]

Cycas_rps10i235 ------------------------------------------.TT.---- [691]

Vitis_rps10i235 ----------------------------------------.TT.C----- [784]

Megaceros_nad9i246 ----------------------------------------......---- [605]

Huperzia_squarrosa_nad5i392 ----------------------------------------....C----- [779]

Huperzia_selago_nad5_intron CTTAAAATCGGAGAGGACTTAAAGATGTTGAACTCCTTCTT.....CTTT [2284]

Huperzia_squarrosa_nad5i1242 CTTAAAATCGGAGAGGACTTAAAGATGTTGAACTCCTTCTT.....CTTT [2296]

Pleurozia_rrn26i827 -------------------------------------------------- [607]

[ 2460 2470 2480 2490 2500]

[ . . . . .]

Huperzia_rps10i235 ------------------------------------AACGC--------A [657]

Huperzia_rps10psi235 ------------------------------------..A..--------. [674]

Cycas_rps10i235 -------------------------------------------------- [691]

Vitis_rps10i235 -------------------------------------.A..C-------G [790]

Megaceros_nad9i246 -------------------------------------....--------. [610]

Huperzia_squarrosa_nad5i392 --------------------------------------...--------. [783]

Huperzia_selago_nad5_intron CAAGCCAGAACCTAGGCTCTGCTAAGATCGAATTCG.....CGCTTACA. [2334]

Huperzia_squarrosa_nad5i1242 CAAGCCAGAACCTAGGCTCTGCTAAGATTGAATTCG.....CGCTTACA. [2346]

Pleurozia_rrn26i827 -------------------------------------------------- [607]

[ 2510 2520 2530 2540 2550]

[ . . . . .]

Huperzia_rps10i235 ACTCAC------AGTGT--------------------------------- [668]

Huperzia_rps10psi235 ......------.....--------------------------------- [685]

Cycas_rps10i235 .TC..T------..G..--------------------------------- [702]

Vitis_rps10i235 G.C.GGCCGCGGG.AA.--------------------------------- [807]

Megaceros_nad9i246 ....G.------..A..--------------------------------- [621]

Huperzia_squarrosa_nad5i392 ....-T------T.A..--------------------------------- [793]

Huperzia_selago_nad5_intron ....T.CAACAA..A..ATTTCGAAGAACGCTCAATCACTTACCAAAGGA [2384]

Huperzia_squarrosa_nad5i1242 ....T.CAACAA..A..ATTTCGAAGAACGCTCAATCACTTACCAAAGGA [2396]

Pleurozia_rrn26i827 ------------T.G.C--------------------------------- [612]

[ 2560 2570 2580 2590 2600]

[ . . . . .]

Huperzia_rps10i235 ----------------GGCAG--------AACACTC---TAG-------- [683]

Huperzia_rps10psi235 ----------------.....--------.......---...-------- [700]

Cycas_rps10i235 ----------------.....--------.G....TGGG...-------- [720]

Vitis_rps10i235 --------------CAA.AGATCTTTGCCGGTG..GACT.G.A------- [836]

Megaceros_nad9i246 ----------------....A--------...G..T---...-------- [636]

Huperzia_squarrosa_nad5i392 ----------------..T.AT-------......A---...-------- [809]

Huperzia_selago_nad5_intron TGAAACTTTATGAATC..A.AC-------......G---...AATAAATA [2424]

Huperzia_squarrosa_nad5i1242 TGAAACTTTATGAATC..A.AC-------......G---...AATAAATA [2436]

Pleurozia_rrn26i827 -----------------------------.....------..-------- [619]

[ 2610 2620 2630 2640 2650]

[ . . . . .]

Huperzia_rps10i235 -------------------------------------------------- [683]

Huperzia_rps10psi235 -------------------------------------------------- [700]

Cycas_rps10i235 -------------------------------------------------- [720]

Vitis_rps10i235 ----------------------------------------------TCTC [840]

Megaceros_nad9i246 -------------------------------------------------- [636]

Huperzia_squarrosa_nad5i392 -------------------------------------------------- [809]

Huperzia_selago_nad5_intron TCTATGCCCACATTATTTGTCAAGATTCATTCCTGTCCTTTGGGCCTGTC [2474]

Huperzia_squarrosa_nad5i1242 TCTATGCCCACATTCTTTGTCAAGATTCATTCCTGTCCTTTGGGCCTGTC [2486]

Pleurozia_rrn26i827 -------------------------------------------------- [619]

[ 2660 2670 2680 2690 2700]

[ . . . . .]

Huperzia_rps10i235 ---TAATAGCATAACC--GCT-----------GGAGCC------------ [705]

Huperzia_rps10psi235 ---.............--...-----------......------------ [722]

Cycas_rps10i235 ---.G.....G....A--.A.-----------.A....AAA-ATAAGGCC [753]

Vitis_rps10i235 GGG.G.CG.A....GGCG..CA----------.A...GACG---AGCAGT [877]

Megaceros_nad9i246 ---.G.........G.--...-----------A.....A-----AAAGTC [665]

Huperzia_squarrosa_nad5i392 ---.G........CA---...-----------...A..A-----AAGGT- [836]

Huperzia_selago_nad5_intron CTT.C.C.T...G.A.CT.T.CTGATTACTGTAA.A.TAGGTGAAAGGTG [2524]

Huperzia_squarrosa_nad5i1242 CTT.C.C.T...G.A.CT.T.CTGATTACTGTAA.A.TAGGTGAAAGGTG [2536]

Pleurozia_rrn26i827 ------------------.T.--------------...------------ [625]

[ 2710 2720 2730 2740 2750]

[ . . . . .]

Huperzia_rps10i235 --TGGGCAA------------CACTGG-----TTGCC------------- [723]

Huperzia_rps10psi235 --.......------------......-----.....------------- [740]

Cycas_rps10i235 CC..AATG.-------------.GAATAGGTAAA.T.------------- [777]

Vitis_rps10i235 CGC..T.G.-------------.GCTTGGCTC.A..TTTAGATTAGTA-- [912]

Megaceros_nad9i246 -A.....G.------------...C.C----T.....------------- [685]

Huperzia_squarrosa_nad5i392 -G...-.C.------------TGGC.--------A..------------- [851]

Huperzia_selago_nad5_intron GAA..A.G.AATTATCCCACTT.AC.CAAGAT.....ATGATCAAATACA [2574]

Huperzia_squarrosa_nad5i1242 GAA..A.G.AATTATCCCACTTCAC.CAAGAT.....ATGATCAAA---A [2583]

Pleurozia_rrn26i827 --.A.-----------------------------...------------- [631]

[ 2760 2770 2780 2790 2800]

[ . . . . .]

Huperzia_rps10i235 ----------------------TGGGCC---------------------- [729]

Huperzia_rps10psi235 ----------------------......---------------------- [746]

Cycas_rps10i235 ----------------------......---------------------- [783]

Vitis_rps10i235 ------------AGGCTAGCAG.AA..TT--------------------- [929]

Megaceros_nad9i246 ----------------------......---------------------- [691]

Huperzia_squarrosa_nad5i392 ----------------------...-..---------------------- [856]

Huperzia_selago_nad5_intron CCTTGGGAATCCAAAGAATGGA..A...CCTTTCGCAAAAGCTGAAGGTA [2624]

Huperzia_squarrosa_nad5i1242 CCTTGGGAATCCAAAGAATGGA..A...CCTTTCGCAAAAGCTGAAGGTA [2633]

Pleurozia_rrn26i827 ----------------------..TA.T---------------------- [637]

[ 2810 2820 2830 2840 2850]

[ . . . . .]

Huperzia_rps10i235 -----------------------------TTCTT---------------- [734]

Huperzia_rps10psi235 -----------------------------.....---------------- [751]

Cycas_rps10i235 ------------------TGAGAAAGAAAC....---------------- [799]

Vitis_rps10i235 ----------------GGCTACAGCGAAGCG...ACCAAGCGCGAAGGAA [963]

Megaceros_nad9i246 -------------------------TAAAC.A.----------------- [699]

Huperzia_squarrosa_nad5i392 -------------------------TAAAC.T..---------------- [865]

Huperzia_selago_nad5_intron AAGCCGTGGGTCCATCGGTGAATAATAAAC.G.CACTTC----------- [2663]

Huperzia_squarrosa_nad5i1242 AAGCCGTGGGTCCATCGGTGAATAATAAAC.G.CACTTC----------- [2672]

Pleurozia_rrn26i827 ------------------------------..C.---------------- [641]

[ 2860 2870 2880 2890 2900]

[ . . . . .]

Huperzia_rps10i235 -----CCTCGCCACTTGAGCCGTATGCGGGGAAA-CTTGCACGTGTGGTT [778]

Huperzia_rps10psi235 -----.T....................------.-............... [789]

Cycas_rps10i235 -----..........................G..-..C.......C.... [843]

Vitis_rps10i235 AGGGC.T........................G..-..C.......C.... [1012]

Megaceros_nad9i246 --------..A...CG..................A..C.......C.... [741]

Huperzia_squarrosa_nad5i392 -----.T.......A...........TT......-.........AC.C.. [909]

Huperzia_selago_nad5_intron ----C..........C...........A......-..AT.....AC.... [2708]

Huperzia_squarrosa_nad5i1242 ----C..........C...........A......-..AT.....AC.... [2717]

Pleurozia_rrn26i827 -----.T...T..T....................-..........C.... [685]

[ 2910 2920 2930 2940]

[ . . . . ]

Huperzia_rps10i235 CTTAGGGAGGTTAAAGCTGAAAATAGCCTACCCATCCCAATA [820]

Huperzia_rps10psi235 .......G..-------------------------------- [799]

Cycas_rps10i235 .......G..GA..-...TGT..G.................- [883]

Vitis_rps10i235 .......G..GAG.-...AGT.GG....AT..T........- [1052]

Megaceros_nad9i246 ..C.A..GA.GG..-...TG----...T....T..T.....- [777]

Huperzia_squarrosa_nad5i392 .CG....G..GA.C-....GG..A........T.....GC.- [949]

Huperzia_selago_nad5_intron .C.....G..GGG--...TGG..G........T.....G..- [2747]

Huperzia_squarrosa_nad5i1242 .C.....G..GGG---..TGG..G........T.....G..- [2755]

Pleurozia_rrn26i827 .......G..GA..GA.ATG...-GT......T....A.C.- [725]

;

END;
