# Supplementary material for: The Mitochondrial Genome of the Lycophyte Huperzia squarrosa: The Most Archaic Form in Vascular Plants
Source: PLoS One. 2012 Apr 12;7(4):e35168. doi: 10.1371/journal.pone.0035168 (PMC3325193; doi:10.1371/journal.pone.0035168)
Supplement: Figure S4 — Alignment of functional genes and their pseudogene pieces in the Huperzia mitochondrial genome. All queries are functional genes whereas subjects are pseudogene pieces. (DOCX) [file pone.0035168.s004.docx]

**atp6 (with intronless pseudogene pieces)**

CDS blast

Exon1: 1-80

Exon2: 81-439

Exon3: 440-759

Exon1+ Exon2

Score = 423 bits (468), Expect = 1e-119

Identities = 357/432 (83%), Gaps = 19/432 (4%)

Strand=Plus/Minus

Query 1 ATGGCTTGGAGTCCACTAGAACAATTTGCCATTATTTCATTGATTCCTATTCATATAGGA 60

|||||||||||||||||||||||||||||||||||||| | |||||||||||||||| |

Sbjct 183913 ATGGCTTGGAGTCCACTAGAACAATTTGCCATTATTTCCTCGATTCCTATTCATATATAA 183854 cox1==>atp4

Query 61 AACTTGTATCTTTCATTTACCAATTCATCTTTGTTTATGCCACTAACTATCAATTTAGTA 120

|||| || ||||||||| ||||||| |||||||||||||| || ||| | |||||||||

Sbjct 183853 AACTCGTCTCTTTCATTCACCAATTTATCTTTGTTTATGCTACCGACTCTTAATTTAGTA 183794

Query 121 TTGCTTTTAGTCAATTTTGTCACCC---GAAATGGAGGACAC--TTAGTACCAAATGCAT 175

|||||| ||| ||||||||||||| |||| | || || || ||||||||||||

Sbjct 183793 TTGCTTCGAGTTAATTTTGTCACCCCAAGAAAAGTGTGATACATTTTATACCAAATGCAT 183734

Query 176 GGCAATCCTTGGTGGAAATGATTTATGATTTTGTGCTTAACTTGGTGAACGAACAAATAA 235

| || | || |||||| |||||||||||| |||||||||||| ||||||||||

Sbjct 183733 GACATT--TTC------ATGATTCATGATTTTGTGCCTAACTTGGTGAATGAACAAATAA 183682

Query 236 GTGGTGCTTCTTCGGTGAAACAACG-Gttttttcctctgatctttgtcacttttactttt 294

||||| ||| | |||| ||||||| ||||||| | ||||||||||||||||||||||||

Sbjct 183681 GTGGTACTTATCCGGTAGAACAACGAGTTTTTTTC-CTGATCTTTGTCACTTTTACTTTT 183623

Query 295 ttattattttGTAATCTTATCGGTATGATACCATATAGTTTTACAGTAACAAGTCATTTT 354

| |||| |||||| ||||| ||||||| || ||||||| ||||||||||||||||||

Sbjct 183622 TCATTACAAAGTAATCCTATCGATATGATATCACATAGTTTGACAGTAACAAGTCATTTT 183563

Query 355 ATAATTACTTTGGGTCTTTCATTATCTCTCTTTATTGGAATAACTATAGTTGGATTCCAA 414

| |||||| |||||| || ||||||| | | |||||||| | |||||||||||||

Sbjct 183562 GTCATTACTCTGGGTCCTTTATTATCT---TCTCCTGGAATAA-TTGAGTTGGATTCCAA 183507

Query 415 ACACATGGGCTT 426

||||||| ||||

Sbjct 183506 ACACATGCGCTT 183495

**atp9 (with intronless pseudogene pieces)**

CDS blast

Exon1: 1-21

Exon2: 22-87

Exon3: 88-94

Exon4: 95-225

Exon2+Exon3+Exon4

Score = 86.0 bits (94), Expect = 1e-18

Identities = 130/183 (71%), Gaps = 13/183 (7%)

Strand=Plus/Plus

Query 45 TGCTTCAGCGGGGGCTGCTGT---AGGTATTGGAAACGTTTTTA-GTTCTTCGACTCATT 100

|| || |||||| |||||| | |||| ||||||||| | |||||| || | |||

Sbjct 299089 TGATTTAGCGGGAGCTGCTATTATAGGTCCTGGAAACGTATGAGCGTTCTTTGATTTATT 299148 trnCGCA==>trnICAU

Query 101 CTGTTGCGCGAAATCCATCATTAGCTAAGCAATTATTTGGTTATGCT------ATTCTAG 154

| || ||||||||||||||| ||||||||||| ||||||||||| | |

Sbjct 299149 CCGTGGCGCGAAATCCATCACGGGCTAAGCAATTTATTGGTTATGCTGCCACCCGGCACG 299208

Query 155 GTTTTGCTT---CAACTGAAGCTACTGCTTTGTCTGCCTTAATGATGGCGTTTTCAATAT 211

||| ||||| ||| ||||| || |||| || ||||| | ||||| || |||||

Sbjct 299209 GTTCTGCTTTAACAAAAGAAGCGCCTCGTTTGCCTAAATTAATTAAGGCGTCTTTAATAT 299268

Query 212 TAT 214

|||

Sbjct 299269 TAT 299271

**cox2 (with intronless pseudogene pieces)**

CDS blast

Exon1: 1-103

Exon2: 104-382

Exon3: 383-700

Exon4: 701-765

Exon1+ Exon2+ Exon3

Score = 554 bits (614), Expect = 3e-159

Identities = 455/552 (82%), Gaps = 21/552 (4%)

Strand=Plus/Minus

Query 1 ATGATTTTGAGAAACATATGGCTATTTGTTCCAATTGCTTATCGTGATGCTGCGGAACCT 60

||||||||||||||||||||||||||||||||||||||||||||||||||||||||| ||

Sbjct 268943 ATGATTTTGAGAAACATATGGCTATTTGTTCCAATTGCTTATCGTGATGCTGCGGAATCT 268884 cox2==>tatC

Query 61 TGGCAATTAGGATTTCAAGACGCAGCAACACCTATGATGCAAGGAATAATTGACTTGCAT 120

|||| || || ||||||| ||||| ||||||||||| ||||||||||||||||||||||

Sbjct 268883 TGGCGATGAGAATTTCAAAACGCAAAAACACCTATGACGCAAGGAATAATTGACTTGCAT 268824

Query 121 CATGATAtttttttCTCTTTAATGATTATATTGATCTTCGTTTTATGGATGTTGGTTCGC 180

||||||||||||||| | ||| ||| ||||||| || || | ||||||||||| ||

Sbjct 268823 CATGATATTTTTTTCCTTCCAATCATTGTATTGATTTTTGTCT---GGATGTTGGTTTGC 268767

Query 181 GCTTTATGGCATTTTCACTATGAAAGAAATCCTATTCCAGAAAGGATTGTTCATGGAACT 240

|||||||||||||||||||| |||||||| | |||||||||||||||||||||||||||

Sbjct 268766 GCTTTATGGCATTTTCACTAGAAAAGAAATGCGATTCCAGAAAGGATTGTTCATGGAACT 268707

Query 241 ACTATAGAGATTATTTGGACCATTTTTCCTAGTATTATTCTGATGTTTATTGCTATACCA 300

|||||||| |||||||||| |||| ||| ||| |||||| ||| ||||| ||||||| |

Sbjct 268706 ACTATAGACATTATTTGGATTATTTCTCCCAGTGTTATTCCGATATTTATGGCTATACTA 268647

Query 301 TCTTTTGCCTTATTATATTCAATGGACGAGGTAGTAGATCCAGCCATTACTATCAAAGCT 360

|||||| | ||||| | |||||| ||| ||||||||||||| ||| || ||||||

Sbjct 268646 CTTTTTGCTTCATTATCTCCAATGGTCGATAAAGTAGATCCAGCCTTTAGTAGCAAAGCC 268587

Query 361 ATTGGACATCAATGGTATTGGAC------TTATGAATATTCAGACT---ATAACCGTTCT 411

|||||||| |||||||||| ||| |||||||| || || || |||||

Sbjct 268586 ATTGGACACCAATGGTATTTGACTACTTATTATGAATCTTTTTGCTTACGCAATAGTTCT 268527

Query 412 GATGAACAGTCATTAACCTTTGA--------CAGTTATATGATTCCAGAAGATGACTCAG 463

|| |||||| ||||||| ||||| |||||| |||||| |||||| | |||

Sbjct 268526 GACGAACAGCCATTAACTTTTGATAGTTATTTAGTTATTTGATTCTGGAAGATAATTCAT 268467

Query 464 AATTAGGTCAATTACGCTTATTAGAAGTGGACAATCGAATGGTTGTACCAGCAAAAACTC 523

|||||||| ||||||| |||||| |||||||| ||||| ||||||||||||| || ||||

Sbjct 268466 AATTAGGTTAATTACGTTTATTAAAAGTGGACGATCGAGTGGTTGTACCAGCCAACACTC 268407

Query 524 ATCTACGTATGA 535

|| || |||||

Sbjct 268406 ATACAC-TATGA 268396

Exon2+ Exon3

Score = 370 bits (410), Expect = 6e-104

Identities = 290/341 (85%), Gaps = 13/341 (4%)

Strand=Plus/Plus

Query 116 TGCATCATGAT-AtttttttCTCTT-TAATGATTATATTGATCTTCGTTTTATGGATGTT 173

||||| ||||| || |||||| || |||| ||| ||||||||||||| ||| |||||||

Sbjct 112293 TGCATTATGATGATATTTTTCCTTTCTAATTATTGTATTGATCTTCGTCTTACGGATGTT 112352 ccmFC1-pseudo==>rps1-pseudo

Query 174 GGTTCGCGCTTTATGGCATTTTCACTATGAAAGAAATCCTATTCCAGAAAGGATTGTTCA 233

|||||||||||||||||||||||||||| | |||||| ||||||||||||||||||||

Sbjct 112353 GGTTCGCGCTTTATGGCATTTTCACTAT---ATAAATCCGATTCCAGAAAGGATTGTTCA 112409

Query 234 TGGAACTACTATAGAGATTATTTGGACCATTTTTCCTAGTATTATTCTGATGTTTATTGC 293

|||||||||||||||||||||| ||| ||| || | || ||||||||||||||||| ||

Sbjct 112410 TGGAACTACTATAGAGATTATTCGGATTATTCTTGCCAG-ATTATTCTGATGTTTATGGC 112468

Query 294 TATACCATCTTTTGCCTTATTATATTCAATGGACGAGGTAGTAGATCCAGCCATTACTAT 353

||||| | ||| ||| | ||||||| ||||||| || | |||||||||||||||||||

Sbjct 112469 TATACTACCTTCTGCTTCATTATATCTAATGGACAAGATGGTAGATCCAGCCATTACTAG 112528

Query 354 CAAAGCTATTGGACATCAATGGTATTGGACTTATGAATATTCAGACTATAACCGTTCTGA 413

||||||||||||||||||||||||| |||||||||| | || | || || |||||||

Sbjct 112529 CAAAGCTATTGGACATCAATGGTATCGGACTTATGAGTCTTTACAC---CACAGTTCTGA 112585

Query 414 TGAACAGTCATTAACCTTTGACAGT----TATATGATTCCA 450

|||||||||| |||| ||| ||||| ||||||||||||

Sbjct 112586 TGAACAGTCACTAACTTTTAACAGTTATATATATGATTCCA 112626

**nad3 (with intronless pseudogene pieces)**

CDS blast:

Exon1: 1-52

Exon2: 53-140

Exon3: 141-357

Exon1+ Exon2+ Exon3

Score = 223 bits (246), Expect = 1e-59

Identities = 201/247 (81%), Gaps = 13/247 (5%)

Strand=Plus/Minus

Query 2 TGGAATTTGCACCTATTTGTGTCTATTTAGTAATCAGTTTGCTACTTTCTTTGATCTCAA 61

||||||||||||||||| ||||||||| |||||||||||||||||| ||||||| |

Sbjct 235967 TGGAATTTGCACCTATTCGTGTCTATTCAGTAATCAGTTTGCTACT---TTTGATCCTCA 235911 trnFGAA==>cox3

Query 62 TTGGTGTTTCTCTTTTATTTGCTTCTTCTTCTGGTTCGGCGTATCCAGAGAAATTGTCAG 121

||||||||| |||||||||||||| || | || ||| |||||| | ||||||

Sbjct 235910 TTGGTGTTTTTCTTTTATTTGCTTTTTACTGTGT---GGCTTATCCAAATGGGATGTCAG 235854

Query 122 CTCAC---GAATGTGGTTTTGATCCTTTTGAT--GATGCCAGAAGTCGTTCCGATATAAG 176

|| || |||||||||||||||| ||||||| |||||||||||||||| || | ||

Sbjct 235853 CTTACTACGAATGTGGTTTTGATCTTTTTGATATGATGCCAGAAGTCGTTTTGAGACAAA 235794

Query 177 ATTTTATCCTGTTTCTATTTCATCCATTACATTCGATTTGGAAGTCA-CCTTTTCATTTC 235

||||||| | ||||| |||||||| ||||||| |||||||||| || ||||||| ||||

Sbjct 235793 ATTTTATTCCGTTTCCATTTCATCTATTACATCTGATTTGGAAGCCATCCTTTTC-TTTC 235735

Query 236 CTTGGGC 242

|| ||||

Sbjct 235734 CTCGGGC 235728

Exon1+ Exon2+ Exon3

Score = 210 bits (232), Expect = 6e-56

Identities = 250/335 (75%), Gaps = 33/335 (10%)

Strand=Plus/Plus

Query 2 TGGAATTTGCACCTATTTGTGTCTATTTAGTAATCAGTTTGCTACTTTCTTTGATCTCAA 61

||||||||||||||||| ||||||||| ||| |||||||||||||| ||||||| |

Sbjct 128097 TGGAATTTGCACCTATTCGTGTCTATTCAGTGATCAGTTTGCTACT---TTTGATCCTCA 128153 nad9==>nad1

Query 62 TTGGTGTTTCTCTTTTATTTGCTTCTTCTTCTGGTTCGGCGTATCCA---GAG----AAA 114

||||||||||| ||| |||||||| | |||| || ||| |||| ||| ||

Sbjct 128154 TTGGTGTTTCTTTTTCATTTGCTTTTCCTTC---TTTGGC--TTCCAAATGAGGAATTAA 128208

Query 115 TTGTCAGCTCACGAATGTGGTTTTGATCCTTTTGATGATGCCAG---AAGTCGTTCCGAT 171

||||| ||| | ||||||||||||||||| |||| ||||||||| |||| ||

Sbjct 128209 TTGTCCGCTTAGGAATGTGGTTTTGATCCCTTTGGTGATGCCAGCAAAAGTAGT------ 128262

Query 172 ATAAGATTTTATCCTGTTTCTATTTCATCCATTACATTCGATTTGGAAGTCACCTTT--- 228

| | | |||| |||||||| | |||||||| ||||||||| |||||||

Sbjct 128263 -GGGGGCTGGCTGTTGTTCCTATTTCACCTATTACATTTTATTTGGAAGCCACCTTTCTC 128321

Query 229 ----TCATTTCCTTGGGCAATCTCT-CTTAACAAGATTGGTTTGTTTGGATTTTGGTCTA 283

| |||| |||||||||||| | || |||||| ||| || |||||||||||| ||||

Sbjct 128322 TTAATTATTTACTTGGGCAATCTTTCCTAAACAAGGTTGATTGGTTTGGATTTTGCTCTA 128381

Query 284 TGATGGTATTTCTATTGATTTCAACGATTGGATTT 318

|| |||||||| ||| ||| || |||||| ||

Sbjct 128382 TGTTGGTATTTTCATTAATTCTCACCATTGGAGTT 128416

Exon1+ Exon2

Score = 93.3 bits (102), Expect = 1e-20

Identities = 72/86 (84%), Gaps = 0/86 (0%)

Strand=Plus/Plus

Query 6 ATTTGCACCTATTTGTGTCTATTTAGTAATCAGTTTGCTACTTTCTTTGATCTCAATTGG 65

|||||||| |||||||||||||| ||||||||||||| | ||| ||| ||| ||||||

Sbjct 63988 ATTTGCACTTATTTGTGTCTATTCAGTAATCAGTTTGTGATTTTTTTTTATCCTAATTGG 64047 cob==>atp1

Query 66 TGTTTCTCTTTTATTTGCTTCTTCTT 91

||||||||||| | | ||||||||

Sbjct 64048 TGTTTCTCTTTCTTGAGGTTCTTCTT 64073

**nad5 (with intronless pseudogene pieces)**

CDS blast

Exon1: 1-392

Exon2: 393-1242

Exon3: 1243-1455

Exon4: 1456-1477

Exon5: 1478-2034

Exon1+Exon2

Score = 542 bits (600), Expect = 5e-155

Identities = 448/545 (82%), Gaps = 25/545 (5%)

Strand=Plus/Minus

Query 3 GTATTTACTCATAGTATTGTTGCCCCTGCTAGGTAGCTTCGTTGCAGGAGCTTTTGGTCG 62

|||||||||| |||| |||||||| ||| |||||||| ||||||||||||||||||||

Sbjct 108187 GTATTTACTCCCAGTACTGTTGCCCTTGCCTGGTAGCTTTGTTGCAGGAGCTTTTGGTCG 108128 nad5==>trnLCAA

Query 63 TTTTCTGGGTTC----------AAAAGGAACCGCTATAGTAACCACCACGTGCGTTTCAT 112

||||| ||||| ||| ||||||||||||||||||||||||||||||||||

Sbjct 108127 TTTTCCGGGTTGTTAAAAAAAAAAAGGGAACCGCTATAGTAACCACCACGTGCGTTTCAT 108068

Query 113 TATCTTCCATTTTATCTTTGATTGCTTTCTATGAAGTTGCACTGGGAGCCAGTGCTTGCT 172

|||||| |||||||||||||||||||| | ||| |||||||||||||||||||||||||

Sbjct 108067 TATCTTATATTTTATCTTTGATTGCTTTTTCTGACGTTGCACTGGGAGCCAGTGCTTGCT 108008

Query 173 ATATCAAGATTGCTCCCTGGATTTTATCGGAGATGTTTGATGCTTCTTGGGGCTTCTTGT 232

||| |||||||||||| |||||| ||| ||||||||| | |||| ||||||| |||

Sbjct 108007 ATACCAAGATTGCTCCTTGGATTGTATTTTCGATGTTTGACGTTTCTCGGGGCTTTTTGC 107948

Query 233 TTGATAGTCTGACTGTAGTTATGTTAATTGTGGTTACATTTGCAAGTAGCTTAGTTCATC 292

||||||||| ||||||||| || ||||| |||||||||||||||||||||||||||||

Sbjct 107947 TTGATAGTCCAACTGTAGTTGCGTCAATTGCGGTTACATTTGCAAGTAGCTTAGTTCATC 107888

Query 293 TTTATTCTATTTCATATATGTCCGAGGATCCACATAGCCCTCGATTTATGTGTTATTTAT 352

||| ||||||| | ||||||||||||||||||||| ||||||||||||||||||||

Sbjct 107887 CCTATCTTATTTCAGAGGTGTCCGAGGATCCACATAGCCTTCGATTTATGTGTTATTTAT 107828

Query 353 CCATTCCTACtttttttATGCTAATGCTGGTTACCGGAGATAACTTTATTCAATTATTCC 412

||||||||||||||||| ||||| ||||||||||||| | || ||||||| | |

Sbjct 107827 CCATTCCTACTTTTTTTCTGCTATTGCTGGTTACCGGGG----CTCCATTCAATCCATTC 107772

Query 413 TAGGA---TGGGAGGGCGTGGGT--CTCGCTTCATATTTGTCAATTAATTTTTGGTTTAC 467

|||| |||| | |||| ||| | |||||| ||||| | ||||||||

Sbjct 107771 AAGGAAGGTGGGTTACCAGGGGTAACTCCATATTTATTTGCCAATTTAGGGATGGTTTAC 107712

Query 468 AC---GACTTCCGGCAAATAAAGCAGCTATCAAAGCTATGCTTGTC---AATCGAGTAGG 521

|| | ||||||||||||||||||||||| |||||||||||||| ||| |||||||

Sbjct 107711 ACGGTGGTTTCCGGCAAATAAAGCAGCTATCGAAGCTATGCTTGTCAAGAATTGAGTAGG 107652

Query 522 TGATT 526

|||||

Sbjct 107651 TGATT 107647

**cox2 (with intron-containing pseudogene pieces)**

Gene blast

Exon1: 1-103

Intron1: 104-1111

Exon2: 1112-1390

Intron1: 1391-3708

Exon3: 3709-4026

Intron4: 4027-6554

Exon4: 6555-6619

Exon3+ Intron3

Score = 295 bits (326), Expect = 4e-80

Identities = 237/280 (85%), Gaps = 17/280 (6%)

Strand=Plus/Minus

Query 3956 CTTTATTAAACGAGAAGGAGTTTACTATGGTCAGTGCAGTGAAATTTGTGGAACCAATCA 4015

||| || ||||||||||||||||||||||||||||||||| || |||||||||| |||||

Sbjct 259801 CTTCATCAAACGAGAAGGAGTTTACTATGGTCAGTGCAGTAAATTTTGTGGAACTAATCA 259742 trnRACG==>cox2

Query 4016 TGCGTTTATGCGTGCGCCCGAATACATAGGCCGACTGCTGAGCCCACTCTGGCTCAG--- 4072

|||||||||||||||||||||||||||||||||||||||||||||||||||||||||

Sbjct 259741 TGCGTTTATGCGTGCGCCCGAATACATAGGCCGACTGCTGAGCCCACTCTGGCTCAGCCA 259682

Query 4073 CCGCACCTTCTCGTTCTTTGGCCAACCTGGATGCGAGCTACCTAAAG---AGCTATCATA 4129

|| ||||||||||||||| || || || |||||||| ||||| | |||||||| |

Sbjct 259681 CCACACCTTCTCGTTCTTCGG----CCCGGTTGCGAGCTGCCTAAGGAGCAGCTATCACA 259626

Query 4130 GCAATATCATGGCTAGAGCAGTAGGTAAAAGCTGGGGATCGATGGGCCTGCTGCTGCCCC 4189

|||||||||||||| ||||| ||||||| |||||||| ||| | | ||||||

Sbjct 259625 GCAATATCATGGCTGGAGCAAGAGGTAAA---CGGGGATCGGGCGGC--GGT-CTGCCCT 259572

Query 4190 GGCTCCCCAGTAAAGCGGAGGATACGGTTAGGATAACGAG 4229

||| ||||||||||| |||||| |||| ||||||| |||

Sbjct 259571 AGCT-CCCAGTAAAGCAGAGGATGCGGTGAGGATAAAGAG 259533

**cox3 (with intron-containing pseudogene pieces)**

Gene blast

Exon1+ Intron1

Score = 1005 bits (1114), Expect = 0.0

Identities = 754/881 (86%), Gaps = 68/881 (8%)

Strand=Plus/Minus

Query 63 TTCACTGGGAGCTTTGGCAAGCACTATGGGTGGCGTTATGTACATGCACTCTTTTGCGGG 122

|||||||||||||||||||||||| ||||||||||||||||||| |||||| || ||||

Sbjct 82538 TTCACTGGGAGCTTTGGCAAGCACCATGGGTGGCGTTATGTACACGCACTCCTTGCCGGG 82479 atp1==>rrn26

Query 123 AGGTGGAACACTTCTTAGTTCAGGCTTGGGAATAATCTTAT---ACACCATGGTGCGCCC 179

|||||||||||||| |||||||||||||| ||| || |||| | ||||||||||||||

Sbjct 82478 AGGTGGAACACTTCCTAGTTCAGGCTTGGAAATGATTTTATTATATACCATGGTGCGCCC 82419

Query 180 GAAGACATTTACTCCGACGAGGAGGGCATTCTCCTCTCCTCCGTGACATCTCAGGCTAGG 239

||||||| |||||||||||||||| | | ||||||||||||||||||

Sbjct 82418 GAAGACACTTACTCCGACGAGGAGTGTAA-------------GTGACATCTCAGGCTAGG 82372

Query 240 TCACGCGCTCAACCTGTAGGGTACACACCCGGGAGCAGCAGGTGTAAATCGAGGCTCGAA 299

|||||||||||||| ||||| ||| ||||| |||||||||||||||||||||| |

Sbjct 82371 TCACGCGCTCAACC---AGGGTGCACCCCCGGCAGCAGCAGGTGTAAATCGAGGC----A 82319

Query 300 GAATGTGGGTCTGCCCCCACCGAGATGCCTCGAGGGAGCAGAAGGTACGGTTAGGATAAC 359

||||| ||||||| ||||||||||||||||||||||||||||||||||

Sbjct 82318 GAATG--------CCCCCACTA----GCCTCGAGGGAGCAGAAGGTACGGTTAGGATAAC 82271

Query 360 GCACGTGATATTACGCATTTCCCGGAGCAGATAGGCCCTCCTGTCCCAAGCAGAATATAG 419

|||||| ||| ||||||||||| | ||||||||||||||||||||||||||||||||||

Sbjct 82270 GCACGTAATACTACGCATTTCCTGACGCAGATAGGCCCTCCTGTCCCAAGCAGAATATAG 82211

Query 420 TGGCAGGGGCACGACAAGCCAGGGCCAGAGTTGGGCACAATACATGGTGTGTGCACAGCA 479

||||||||||||||| |||| | |||||||||||||||||||| |||||||||||||

Sbjct 82210 TGGCAGGGGCACGACGAGCCCTTAC-AGAGTTGGGCACAATACATGATGTGTGCACAGCA 82152

Query 480 CCTGAGAAATCGTGGGGAACTCTACGAGGAGACCGGCT--GGGCAACCAGCCGAATGCGC 537

|| || |||||||| ||||| ||||||||||| ||||||||||||||||||||

Sbjct 82151 CCCGACAAATCGTGAGGAAC------AGGAGACCGGCCTAGGGCAACCAGCCGAATGCGC 82098

Query 538 TAGGACCTCACCCTTCGAGAATTCTGCCTCCTTCGAAGGGGGCGGAGAACGAGGAGTGTA 597

||||||||||||||||||| |||||||||||| |||| |||| ||||| |

Sbjct 82097 TAGGACCTCACCCTTCGAG-------CCTCCTTCGAAGATGGCGAAGAA--AGGAGGG-- 82049

Query 598 TGGAAGTCGGGGAAATAATGCATT-CCACGGGAATGGCATTCCGAGGGTCCGTAGTAGCG 656

| |||||||||||||||||||| |||||| ||||||||||||||||||||||||||||

Sbjct 82048 --GGAGTCGGGGAAATAATGCATTACCACGGTAATGGCATTCCGAGGGTCCGTAGTAGCG 81991

Query 657 CTTCGGGCGGAAACACTCATTCTACCTGGCCGCGAAGGGACCTAGCTTACGATCGTACTG 716

|||||||||||||||||||||||||||||| | |||||||||| ||||||||||||

Sbjct 81990 CTTCGGGCGGAAACACTCATTCTACCTGGCTTTG---GGACCTAGCTCACGATCGTACTG 81934

Query 717 CTCGAGACCCGAGATCTCGGCGGAGGATGGCAATGCTTCAGCTCGTCCCAGCGGAGCAGG 776

| |||||||| ||||||||| ||||| |||||||||||||||||||||||||| |

Sbjct 81933 CGCGAGACCCAAGATCTCGG------ATGGCGATGCTTCAGCTCGTCCCAGCGGAGCAAG 81880

Query 777 CCGCGCAGCCAGCAAGGTTGACTGGTCCGGCCGCATCCGTATCTCAGTCTCTGCACAAAT 836

||||||||||||||||||||||||||| | |||| |||||||||||||||||||||||||

Sbjct 81879 CCGCGCAGCCAGCAAGGTTGACTGGTCTGACCGCGTCCGTATCTCAGTCTCTGCACAAAT 81820

Query 837 TCAAACCTACCAGGGAACATGGTGGAGATCGGAGGTGCAAGCCATATGAGGCGAAAATCC 896

|||||||||||||||||||||||||||||||||||||||||||||| ||||||||||

Sbjct 81819 TCAAACCTACCAGGGAACATGGTGGAGATCGGAGGTGCAAGCCATAGAAGGCGAAAATAT 81760

Query 897 CACGTATAGTTTTGAGGGCAGCCACTCAG-ACATGGATGGA 936

||||| |||||||||||||||||| |||| || || |||||

Sbjct 81759 CACGTCTAGTTTTGAGGGCAGCCATTCAGAACTTGAATGGA 81719

Exon1: 1-171

Intron1: 172-947

Exon2: 948-1574

Exon1+ Intron1+ Exon2

Score = 1094 bits (1212), Expect = 0.0

Identities = 918/1116 (82%), Gaps = 101/1116 (9%)

Strand=Plus/Plus

Query 3 GAATGTCTCTCAAAAG---CATCCTTATCATTTAGTAGATCCAAGTCCATGGCCTATTT- 58

|||||| ||||||||| ||||||||||||| |||||||||||||||||| |||||||

Sbjct 379357 GAATGTGTCTCAAAAGAAGCATCCTTATCATTCAGTAGATCCAAGTCCATGACCTATTTC 379416 rps10==>rpl2

Query 59 TGGGTTCACTGGGAGCTTTGGCAAGCACTATGGGTGGCGTTATGTACATGCACTCTTTTG 118

| |||| ||||||| |||||||||||| || ||||||||||||||||||||||| ||

Sbjct 379417 TAGGTT-ACTGGGATTTTTGGCAAGCACCATAGGTGGCGTTATGTACATGCACTCCTTGC 379475

Query 119 CGGGAGGTGGAACA-----CTTCTTAGTTCAGGCTTGGGAATAAT---CTTATACACCAT 170

||||| |||| | | || ||||||||||| ||||||||| |||||| ||||

Sbjct 379476 CGGGA--TGGACCTTCGGCCCTCCTAGTTCAGGCTCGGGAATAATGATCTTATATACCAC 379533

Query 171 GGTGCGCCCGAAGACATTTACTCCGACGAGGAGGGCATTCTCCTCTCCTCCGTGACATCT 230

|||||||||||||||| |||||||||||||||| | | |||||||||

Sbjct 379534 GGTGCGCCCGAAGACACTTACTCCGACGAGGAGTGTAA-------------GTGACATCT 379580

Query 231 CAGGCTAGGTCACGCGCTCAACCTGTAGGGTACACACCCGGGAGCAGCAGGTGTAAATCG 290

||||||||||||||||||||||| ||||| ||| ||||| ||||||||||||||||||

Sbjct 379581 CAGGCTAGGTCACGCGCTCAACC---AGGGTGCACCCCCGGCAGCAGCAGGTGTAAATCG 379637

Query 291 AGGCTCGAAGAATGTGGGTCTGCCCCCACCGAGATGCCTCGAGGGAGCAGAAGGTACGGT 350

|||| |||||| ||||||| |||||||||||||||||||||||||

Sbjct 379638 AGGC----AGAATG--------CCCCCACTA----GCCTCGAGGGAGCAGAAGGTACGGT 379681

Query 351 TAGGATAACGCACGTGATATTACGCATTTCCCGGAGCAGATAGGCCCTCCTGTCCCAAGC 410

||||||||||||||| ||| ||||||||| | |||||||||||||||||||||||||

Sbjct 379682 TAGGATAACGCACGTAATA---CGCATTTCCTGACGCAGATAGGCCCTCCTGTCCCAAGC 379738

Query 411 AGAATATAGTGGCAGGGGCACGAC---------AAGCCAGGGCCAGAGTTGGGCACAATA 461

||||||||||||||||| |||||| |||||||||||| ||||||||||||||

Sbjct 379739 AGAATATAGTGGCAGGGACACGACGAGCAAGCCAAGCCAGGGCCAAAGTTGGGCACAATA 379798

Query 462 CATGGTGTGTGCACAGCACCTGAG---AAATCGTGGGGAACTCTACGAGGAGACCGGCT- 517

||||||||||||||||||||||| ||||||||| ||| ||||||||||||

Sbjct 379799 CATGGTGTGTGCACAGCACCTGACGACAAATCGTGGTAAAC-----GAGGAGACCGGCCT 379853

Query 518 -GGGCAACCAGCCGAATGCGCTAGGACCTCACCCTTCGAGAATTCTGCCTCCTTCGAAGG 576

||||||||||||||||||||||||||||||||||||||| ||||||||||||

Sbjct 379854 AGGGCAACCAGCCGAATGCGCTAGGACCTCACCCTTCGAG-------CCTCCTTCGAAGA 379906

Query 577 GGGCGGAGAACGAGGAGTGTATGGAAGTCGGGGAAATAATGCATT-CCACGGGAATGGCA 635

|||| |||| ||||| | | |||||||||||||||||||| |||||| |||||||

Sbjct 379907 TGGCGAAGAA--AGGAGGG----GGAGTCGGGGAAATAATGCATTACCACGGTAATGGCA 379960

Query 636 TTCCGAGGGTCCGTAGTAGCGCTTCGGGCGGAAACACTCATTCTACCTGGCCGCGAAGGG 695

||||||||||||||||||||||||||||||||||||||||||||||||||| | ||

Sbjct 379961 TTCCGAGGGTCCGTAGTAGCGCTTCGGGCGGAAACACTCATTCTACCTGGCTTTG---GG 380017

Query 696 ACCTAGCTTACGATCGTACTGCTCGAGACCCGAGATCTCGGCGGAGGATGGCAATGCTTC 755

|||||||| ||||||||||||| |||||||| ||||||||| ||||| |||||||

Sbjct 380018 ACCTAGCTCACGATCGTACTGCGCGAGACCCAAGATCTCGG------ATGGCGATGCTTC 380071

Query 756 AGCTCGTCCCAGCGGAGCAGGCCGCGCAGCCAGCAAGGTTGACTGGTCCGGCCGCATCCG 815

||||||||||||||||||| |||||||||||||||||||||||||||| | ||||||||

Sbjct 380072 AGCTCGTCCCAGCGGAGCAAGCCGCGCAGCCAGCAAGGTTGACTGGTCTGACCGCATCC- 380130

Query 816 TATCTCAGTCTCTGCACAA--ATTCAAACCTACCAGGGAACATGGTGGAGATCGGAGGTG 873

||||| ||||||||| || |||||||||||||||||||||| || ||||||||| ||

Sbjct 380131 TATCTTAGTCTCTGCTGAAGAATTCAAACCTACCAGGGAACATAATGAAGATCGGAGATG 380190

Query 874 CAAGCCATATGAGGCGAAAATCCCACGTATAGTTTTGAGGGCAGCCACTCAGACATGGAT 933

||||||||| ||| ||||| |||| |||||||| || |||||| |||||| |||||

Sbjct 380191 CAAGCCATAGAAGGA-AAAATATTACGTCTAGTTTTGGGGACAGCCATTCAGACGTGGAT 380249

Query 934 GGACTGACCCCTAGTTTTTATGGTGGCGCGATGTTATACGTGAATCCACTTACGAAGGAC 993

||||||||||||| || ||||||| |||||||||| | ||| ||||||||||||||

Sbjct 380250 GGACTGACCCCTA-----TAGGGTGGCGTGATGTTATACATAAATTCACTTACGAAGGAC 380304

Query 994 ATCATACATTTGTGGTACAATTAGGACTTCGCTACGGTTTTATTTTGTTCATTGTCTCAG 1053

||||||||||||||||||||||||| | | ||||| |||||||||||| || | |

Sbjct 380305 ATCATACATTTGTGGTACAATTAGGGCCAAGGGCCGGTTGTATTTTGTTCATCATCCCGG 380364

Query 1054 AGGTTATGTCTTTTCTAGCTTTCTTTTGGGCTTTTT 1089

||| | || | || |||||| || | |||||||||

Sbjct 380365 GGGTCACGTATCTTTTAGCTTCCTCTCGGGCTTTTT 380400

Exon1+ Intron1

Score = 1005 bits (1114), Expect = 0.0

Identities = 754/881 (86%), Gaps = 68/881 (8%)

Strand=Plus/Plus

Query 63 TTCACTGGGAGCTTTGGCAAGCACTATGGGTGGCGTTATGTACATGCACTCTTTTGCGGG 122

|||||||||||||||||||||||| ||||||||||||||||||| |||||| || ||||

Sbjct 320906 TTCACTGGGAGCTTTGGCAAGCACCATGGGTGGCGTTATGTACACGCACTCCTTGCCGGG 320965 rrn26==>nad6

Query 123 AGGTGGAACACTTCTTAGTTCAGGCTTGGGAATAATCTTAT---ACACCATGGTGCGCCC 179

|||||||||||||| |||||||||||||| ||| || |||| | ||||||||||||||

Sbjct 320966 AGGTGGAACACTTCCTAGTTCAGGCTTGGAAATGATTTTATTATATACCATGGTGCGCCC 321025

Query 180 GAAGACATTTACTCCGACGAGGAGGGCATTCTCCTCTCCTCCGTGACATCTCAGGCTAGG 239

||||||| |||||||||||||||| | | ||||||||||||||||||

Sbjct 321026 GAAGACACTTACTCCGACGAGGAGTGTAA-------------GTGACATCTCAGGCTAGG 321072

Query 240 TCACGCGCTCAACCTGTAGGGTACACACCCGGGAGCAGCAGGTGTAAATCGAGGCTCGAA 299

|||||||||||||| ||||| ||| ||||| |||||||||||||||||||||| |

Sbjct 321073 TCACGCGCTCAACC---AGGGTGCACCCCCGGCAGCAGCAGGTGTAAATCGAGGC----A 321125

Query 300 GAATGTGGGTCTGCCCCCACCGAGATGCCTCGAGGGAGCAGAAGGTACGGTTAGGATAAC 359

||||| ||||||| ||||||||||||||||||||||||||||||||||

Sbjct 321126 GAATG--------CCCCCACTA----GCCTCGAGGGAGCAGAAGGTACGGTTAGGATAAC 321173

Query 360 GCACGTGATATTACGCATTTCCCGGAGCAGATAGGCCCTCCTGTCCCAAGCAGAATATAG 419

|||||| ||| ||||||||||| | ||||||||||||||||||||||||||||||||||

Sbjct 321174 GCACGTAATACTACGCATTTCCTGACGCAGATAGGCCCTCCTGTCCCAAGCAGAATATAG 321233

Query 420 TGGCAGGGGCACGACAAGCCAGGGCCAGAGTTGGGCACAATACATGGTGTGTGCACAGCA 479

||||||||||||||| |||| | |||||||||||||||||||| |||||||||||||

Sbjct 321234 TGGCAGGGGCACGACGAGCCCTTAC-AGAGTTGGGCACAATACATGATGTGTGCACAGCA 321292

Query 480 CCTGAGAAATCGTGGGGAACTCTACGAGGAGACCGGCT--GGGCAACCAGCCGAATGCGC 537

|| || |||||||| ||||| ||||||||||| ||||||||||||||||||||

Sbjct 321293 CCCGACAAATCGTGAGGAAC------AGGAGACCGGCCTAGGGCAACCAGCCGAATGCGC 321346

Query 538 TAGGACCTCACCCTTCGAGAATTCTGCCTCCTTCGAAGGGGGCGGAGAACGAGGAGTGTA 597

||||||||||||||||||| |||||||||||| |||| |||| ||||| |

Sbjct 321347 TAGGACCTCACCCTTCGAG-------CCTCCTTCGAAGATGGCGAAGAA--AGGAGGG-- 321395

Query 598 TGGAAGTCGGGGAAATAATGCATT-CCACGGGAATGGCATTCCGAGGGTCCGTAGTAGCG 656

| |||||||||||||||||||| |||||| ||||||||||||||||||||||||||||

Sbjct 321396 --GGAGTCGGGGAAATAATGCATTACCACGGTAATGGCATTCCGAGGGTCCGTAGTAGCG 321453

Query 657 CTTCGGGCGGAAACACTCATTCTACCTGGCCGCGAAGGGACCTAGCTTACGATCGTACTG 716

|||||||||||||||||||||||||||||| | |||||||||| ||||||||||||

Sbjct 321454 CTTCGGGCGGAAACACTCATTCTACCTGGCTTTG---GGACCTAGCTCACGATCGTACTG 321510

Query 717 CTCGAGACCCGAGATCTCGGCGGAGGATGGCAATGCTTCAGCTCGTCCCAGCGGAGCAGG 776

| |||||||| ||||||||| ||||| |||||||||||||||||||||||||| |

Sbjct 321511 CGCGAGACCCAAGATCTCGG------ATGGCGATGCTTCAGCTCGTCCCAGCGGAGCAAG 321564

Query 777 CCGCGCAGCCAGCAAGGTTGACTGGTCCGGCCGCATCCGTATCTCAGTCTCTGCACAAAT 836

||||||||||||||||||||||||||| | |||| |||||||||||||||||||||||||

Sbjct 321565 CCGCGCAGCCAGCAAGGTTGACTGGTCTGACCGCGTCCGTATCTCAGTCTCTGCACAAAT 321624

Query 837 TCAAACCTACCAGGGAACATGGTGGAGATCGGAGGTGCAAGCCATATGAGGCGAAAATCC 896

|||||||||||||||||||||||||||||||||||||||||||||| ||||||||||

Sbjct 321625 TCAAACCTACCAGGGAACATGGTGGAGATCGGAGGTGCAAGCCATAGAAGGCGAAAATAT 321684

Query 897 CACGTATAGTTTTGAGGGCAGCCACTCAG-ACATGGATGGA 936

||||| |||||||||||||||||| |||| || || |||||

Sbjct 321685 CACGTCTAGTTTTGAGGGCAGCCATTCAGAACTTGAATGGA 321725

**rps10 (with intron-containing pseudogene pieces)**

Gene blast

Exon1: 1-235

Intron1: 236-1054

Exon2: 1055-1131

Exon1+Intron1

Score = 1086 bits (1204), Expect = 0.0

Identities = 888/1061 (84%), Gaps = 72/1061 (7%)

Strand=Plus/Plus

Query 1 ATGACTGCCAAAATCTGCATTGTGATTAAATCTTTTGAAAATAAAAGGCTGGGGCATCTG 60

|||||||||||||| || |||||||||||||||||||| ||| |||||| ||||||||||

Sbjct 406015 ATGACTGCCAAAATATGTATTGTGATTAAATCTTTTGAGAATCAAAGGCCGGGGCATCTG 406074 trnYGUA==>nad4

Query 61 CATAACACACGCAAGATTGGATTGCCTCAAAAACAAATCTTATATACAGTG-TTACGATC 119

|||||||||||| |||||| ||||||||||||||||||||||||| ||||| |||| ||

Sbjct 406075 CATAACACACGCGAGATTGAATTGCCTCAAAAACAAATCTTATATGCAGTGATTACTATA 406134

Query 120 ACCTCATATTGATAAAAAGTCTAGAGAACAATTTGAAATGAGGATACATAAAGAATTGCT 179

||||||||||| ||||||| | || ||| ||| |||||||||||| ||| |||||||

Sbjct 406135 ACCTCATATTGGGAAAAAGTATGGAAAACGATTCGAAATGAGGATA---AAACAATTGCT 406191

Query 180 GGTCATAGAAACGGAAACGCCTAAATTGCGCGAAAAGTTACATTGGTTAAAACTCCGTGC 239

|||||| |||| ||||||||| |||||||| |||| ||||||||||||||||||||

Sbjct 406192 GGTCATTGAAAGGGAAACGCCAAAATTGCG-----AGTTGCATTGGTTAAAACTCCGTGC 406246

Query 240 GACTCGAAGGACATAAGACAATGCGTATAGCCCTTGAG---TTATATCGCCATCCCAGCC 296

||||| | |||||||||||||||||||||||||||| |||| ||||||||||||||

Sbjct 406247 GACTCTAGGGACATAAGACAATGCGTATAGCCCTTGTTGGTTTATCTCGCCATCCCAGCC 406306

Query 297 GACGGGATACTCCTACTCC----ACCATGCCTTCCTGGCAACGGAAGGGTATG-AAGCGT 351

|| || || |||||||| | |||| | ||||||| ||| | | ||| || ||| ||

Sbjct 406307 GAGGGTATGCTCCTACTTCCTTCACCAGTCGTTCCTGGTAACTGGAAGGTCTGGAAGTGT 406366

Query 352 GGGAAACAATTACATAAATGTATGATACAGCATATACACTTGCTAGTAGGAGTGGCAAGA 411

|| ||||| ||||| || | ||||||| |||||| ||| |||||| |||||||||||

Sbjct 406367 GGAAAACATTTACAGAAGTTTATGATAGAGCATACACATCTGCTAG---GAGTGGCAAGA 406423

Query 412 CTATTGATCAACGTAAGTGAACTGTGCGACGACGCTTCGTAAAACCGC----GTCAGAGA 467

||||||||||||| ||||||||||||||||||||||||||||| ||| | ||||||

Sbjct 406424 CTATTGATCAACGCTAGTGAACTGTGCGACGACGCTTCGTAAAAGCGCACCAGACAGAGA 406483

Query 468 GCC-TATTGAGTAGGGCCG--------ATAGG-GACGAAAAATGAGCCAACGAAGCAATG 517

|| || | ||||||||| || || | |||| ||||||||||| ||||||||

Sbjct 406484 GCTGTAATT-GTAGGGCCGCCGGCTCCATTGGCGGCGAAGAATGAGCCAACAAAGCAATG 406542

Query 518 TGATTTGGGACACGATGGGAGTTTGCGTGCCTCGATCGGCAAATATCACCGGAGTATAGC 577

||||| ||||| ||||| ||||||||||||||||||||||||||||| ||| |||

Sbjct 406543 TGATTGGGGACGCGATGTTTAGTTGCGTGCCTCGATCGGCAAATATCACCGAAGTGAAGC 406602

Query 578 ACAAGATCGCCAAATCTT-----GCCAGAGAGTCAAACCTGTCAACAAGGTAAACCCAAA 632

||||||||||||||| || ||||||||||||| |||||| | |||||| ||||

Sbjct 406603 ACAAGATCGCCAAATTTTCTTTGGCCAGAGAGTCAA-CCTGTCCGCTAGGTAACCCCAC- 406660

Query 633 GGGCTCCCCGGCCTCGGGAGGTTTGGTCGTGAGATCAAATAGTGCCCAGTGGGCAGAAGA 692

||||||||||| | |||||||||||||||||||||||||| |||||||||||||||||

Sbjct 406661 GGGCTCCCCGGGCAAGGGAGGTTTGGTCGTGAGATCAAATACCGCCCAGTGGGCAGAAGA 406720

Query 693 ---CGATCCAAAAAG-CGAAGGCTC--AACCTGGTTCAGCAGG-TGAGATAGGCGACAAT 745

|||||||| ||| |||| || | || || ||| ||| | || || |||

Sbjct 406721 AGACGATCCAAGAAGGCGAAAGCCCGGAAAAA----CAAAAGGCTGATA-AGTAGAAAAT 406775

Query 746 TCGCCAAGTGCTGACTCGAATCTTGAGTGACGAAACACTATTA---CCAGGCTACTCAAT 802

|||| ||||||||| ||||||||||||| ||||||||||| ||||||||||||||

Sbjct 406776 TCGC----TGCTGACTCAAATCTTGAGTGACAAAACACTATTATTACCAGGCTACTCAAT 406831

Query 803 TTAATTGACCACGCGAAAGCAGCGGTCAATACCGCAAGCAGTGTGCGGGTGGATATTGGT 862

||||| |||||||||||||||||||||||||||||||||||||||||||||||||||||

Sbjct 406832 TTAATCAACCACGCGAAAGCAGCGGTCAATACCGCAAGCAGTGTGCGGGTGGATATTGGT 406891

Query 863 CAATCGCAAAGTCGCGACATACGCAACGCAACTCACAGTGTGGCAGAACACTCTAGTAAT 922

||||||||||||||| ||| || |||||||||||||||||||||||||||||||||

Sbjct 406892 CAATCGCAAAGTCGCAACA-----AAAGCAACTCACAGTGTGGCAGAACACTCTAGTAAT 406946

Query 923 AGCATAACCGCTGGAGCCTGGGCAACACTGGTTGCCTGGGCCTTCTTCCTCGCCACTTGA 982

|||||||||||||||||||||||||||||||||||||||||||||||| |||||||||||

Sbjct 406947 AGCATAACCGCTGGAGCCTGGGCAACACTGGTTGCCTGGGCCTTCTTCTTCGCCACTTGA 407006

Query 983 GCCGTATGCGGGGAAACTTGCACGTGTGGTTCTTAGGGAGG 1023

||||||||| ||||||||||||||||||||||| ||

Sbjct 407007 GCCGTATGC------ACTTGCACGTGTGGTTCTTAGGGGGG 407041
