# Supplementary material for: The Mitochondrial Genome of the Lycophyte Huperzia squarrosa: The Most Archaic Form in Vascular Plants
Source: PLoS One. 2012 Apr 12;7(4):e35168. doi: 10.1371/journal.pone.0035168 (PMC3325193; doi:10.1371/journal.pone.0035168)
Supplement: Table S1 — Pseudogene pieces in intergenic spacers of Huperzia squarrosa mitochondrial genome. (DOC) [file pone.0035168.s005.doc]

**Table S1.** Pseudogene pieces in intergenic spacers of *Huperzia squarrosa* mitochondrial genome1.

| ***Spacer*** | **Gene piece^2^** | **Length (bp)** | **Identity^3^** | **Direction^4^** |
| --- | --- | --- | --- | --- |
| *atp1-rrn26* | *atp4* (3-362) | 355 | 93% | - |
|  | *cox3* (63-163)^a^ | 101 | 91% | - |
|  | *cox1* (1495-1596) | 102 | 98% | - |
| *atp4-trnKuuu* | *nad6* (6-249) | 246 | 80% | - |
|  | *rrn5* (26-109) | 91 | 82% | - |
| *atp6-nad3* | *nad4L* (188-278) | 87 | 80% | - |
| *atp8-rrn5* | *nad5* (1478-2034) | 557 | 100% | + |
| *atp9-trnRacg* | *nad9* (3-356) | 358 | 95% | + |
|  | *cox3* (511-786) | 287 | 80% | + |
|  | *cob* (328-545) | 226 | 78% | + |
|  | *cob* (1051-1142) | 97 | 73% | + |
| *ψccmFC-ψrps1* | *cox2* (116-450)^b^ | 334 | 85% | + |
| *cob-atp1* | *sdh3* (223-344) | 122 | 88% | + |
|  | *rpl2* (1-59) | 61 | 79% | - |
|  | *nad3* (6-91)^b^ | 86 | 84% | + |
|  | *rrn26* (2192-2396) | 205 | 92% | - |
|  | *rrn26* (2580-2879) | 304 | 77% | - |
|  | *atp8* (11-468) | 436 | 76% | - |
|  | *atp8* (214-410) | 187 | 76% | - |
| *cox1-atp4* | *atp4* (397-533) | 137 | 93% | - |
|  | *atp6* (1-426)^b^ | 419 | 83% | - |
|  | *nad5* (1859-1919) | 61 | 92% | - |
| *cox2-tatC* | *cox2* (1-535)^b^ | 548 | 82% | - |
|  | *atp4* (467-522) | 56 | 89% | - |
| *cox3-rps12* | *atp1* (1054-1537) | 481 | 88% | - |
|  | *atp1* (1-181) | 191 | 90% | - |
| *nad1-trnMcau* | *cob* (935-1109) | 187 | 85% | + |
| *nad2-trnMfcau* | *atp1* (9-453) | 485 | 81% | + |
|  | *cox1* (1308-1575) | 285 | 86% | + |
| *nad4L-rps10* | *rps4* (8-82) | 76 | 93% | + |
| *nad5-trnLcaa* | *nad5* (3-526)^b^ | 541 | 82% | - |
| *nad6-rps11* | *nad6* (2-549) | 549 | 80% | + |
|  | *cox1* (1309-1599) | 340 | 76% | + |
|  | *rrn26* (2589-2871) | 295 | 78% | + |
| *nad9-nad1* | *atp1* (995-1053) | 59 | 88% | + |
|  | *nad3* (2-318)^b^ | 320 | 75% | + |
|  | *nad1* (143-394) | 248 | 78% | + |
|  |  |  |  |  |
| *rps10-rpl2* | *cox2* (142-265) | 125 | 83% | + |
|  | *cox3* (3-169)^a^ | 176 | 82% | + |
|  | *cox3* (176-313)^a^ | 138 | 81% | + |
| *rps11- ψtrnMfcau* | *atp1* (993-1345) | 365 | 83% | + |
|  | *atp4* (1-534) | 511 | 86% | + |
|  | *cox1* (28-174) | 146 | 84% | + |
| *rps12-trnEuuc* | *nad5* (393-1163) | 800 | 84% | - |
| *rps13-trnRucu* | *atp8* (224-299) | 79 | 82% | + |
| *rrn26-nad6* | *cox3* (63-163)^a^ | 101 | 91% | + |
|  | *cox1* (1495-1596) | 102 | 98% | + |
|  | *atp4* (3-362) | 355 | 93% | + |
| *trnCgca-trnIcau* | *atp1* (216-992) | 781 | 81% | + |
|  | *rps12* (1-170) | 167 | 86% | - |
|  | *atp9* (45-214)^b^ | 183 | 71% | + |
|  | *rps3* (76-198) | 133 | 77% | + |
| *trnFgaa-cox3* | *atp1* (94-176) | 81 | 87% | - |
|  | *rps2* (1-681) | 677 | 78% | + |
|  | *nad3* (2-242)^b^ | 240 | 81% | - |
| *trnGgcc-cob* | *cob* (579-655) | 73 | 86% | - |
| *ψtrnKuuu-trnFgaa* | *cob* (838-1142) | 307 | 79% | - |
| *trnLuaa-rps4* | *rrn26* (414-496) | 85 | 84% | + |
| *trnMcau-trnAugc* | *nad4* (2-441) | 450 | 86% | - |
|  | *rrn5* (27-106) | 80 | 91% | - |
| *ψtrnMfcau-trnNguu* | *atp8* (9-454) | 447 | 70% | + |
| *trnPugg-atp9* | *sdh3* (1-366) | 368 | 80% | + |
| *trnQuug-atp8* | *rrn26* (2061-2396) | 367 | 75% | + |
|  | *rrn26* (2580-2862) | 295 | 78% | + |
| *trnRacg-cox2* | *cox2* (630-700)^a^ | 71 | 93% | - |
| *trnRucu-trnCgca* | *nad4L* (168-265) | 112 | 74% | - |
|  | *nad6* (2-211) | 197 | 74% | - |
|  | *rrn26* (2585-2844) | 263 | 86% | - |
| *trnYgua-nad4* | *rps10* (1-238)^a^ | 231 | 87% | + |

^1^Only gene pieces longer than 50 bp in intergenic spacers are reported here.

^2^The numbers following the gene name in the parenthesis indicate the range of the functional coding sequence to which the pseudogene piece matched.

^3^The identity is calculated by dividing the number of identical nucleotides by the aligned length of the pseudo- and functional genes.

^4^“+” indicates that direction of the pseudogene piece is the same as the genes on the outside of the circle in figure 1; “-” indicates that the pseudogene piece shows the opposite direction.

^a^Intron sequence(s) were found near the pseudogene piece. When calculating the pseudogene length, the intron sequences were not included because it is difficult to determine the boundaries.

^b^A joint piece of two or more exons was found, presumably resulted from a retroposition event.
