# Supplementary material for: The Mitochondrial Genome of the Lycophyte Huperzia squarrosa: The Most Archaic Form in Vascular Plants
Source: PLoS One. 2012 Apr 12;7(4):e35168. doi: 10.1371/journal.pone.0035168 (PMC3325193; doi:10.1371/journal.pone.0035168)
Supplement: Table S2 — Gene contents in mitochondrial genomes of selected charophyte and land plants. (DOC) [file pone.0035168.s006.doc]

**Table S2.** Gene contents in mitochondrial genomes of selected charophyte and land plants^1^.

| **gene/species** |  | ***Ch.vu.*** | ***Ma.po.*** | ***Ph.pa.*** | ***Me.ae.*** | ***Hu. sq.*** | ***Is. en.*** | ***Se*.*mo*.** | ***Cy.ta.*** | ***Or.sa.*** | ***Br.na.*** |
| --- | --- | --- | --- | --- | --- | --- | --- | --- | --- | --- | --- |
| *atp1* | a1 | + | + | + | + | + | + | + | + | + | + |
| *atp4* | a4 | + | + | + | + | + | + |  | + |  |  |
| *atp6* | a6 | + | + | + | + | + | + | + | + | + | + |
| *atp8* | a8 | + | + | + | ψ | + | + | + | + |  |  |
| *atp9* | a9 | + | + | + | + | + | + | + | + | + | + |
| *ccmB* | mb | + | + | + |  |  |  |  | + | + | + |
| *ccmC* | mc | + | + | + |  |  |  |  | + | + | + |
| *ccmF*^2^ | mf | + |  |  |  |  |  |  |  |  |  |
| *ccmFC*^2^ | my |  | + | + | ψ | ψ |  |  | + | + | + |
| *ccmFN*^2^ | mz |  | + | + |  |  |  |  | + | + | + |
| *cob* | cb | + | + | + | + | + | + | + | + | + | + |
| *cox1* | c1 | + | + | + | + | + | + | + | + | + | + |
| *cox2* | c2 | + | + | + | + | + | + | + | + | + | + |
| *cox3* | c3 | + | + | + | + | + | + | + | + | + | + |
| *nad1* | n1 | + | + | + | + | + | + | + | + | + | + |
| *nad2* | n2 | + | + | + | + | + | + | + | + | + | + |
| *nad3* | n3 | + | + | + | + | + | + | + | + | + | + |
| *nad4* | n4 | + | + | + | + | + | + | + | + | + | + |
| *nad4L* | na | + | + | + | + | + | + | + | + | + | + |
| *nad5* | n5 | + | + | + | + | + | + | + | + | + | + |
| *nad6* | n6 | + | + | + | + ψ | + | + | + | + | + | + |
| *nad7* | n7 | + | ψ | + |  |  | + | + | + | + | + |
| *nad9* | n9 | + | + | + | + | + | + | + | + | + | + |
| *rpl2* | l2 | + | + | + |  | + |  |  | + | + | + |
| *rpl5* | l5 | + | + | + | ψ | + | + |  | + | + | + |
| *rpl6* | l6 | + | + | + | ψ | + |  |  |  |  |  |
| *rpl10* | l10 | + | + | + | + | + |  |  | + |  | ψ |
| *rpl14* | l14 | + |  |  |  |  |  |  |  |  |  |
| *rpl16* | l16 | + | + | + |  | + |  |  | + | + | + |
| *rps1* | s1 | + | + | + | ψ | ψ | ψ |  | + | + |  |
| *rps2* | s2 | + | + | + |  | + | + |  | + | + |  |
| *rps3* | s3 | + | + | + |  | + | + |  | + | + | + |
| *rps4* | s4 | + | + | + | ψ | + | + |  | + | + | + |
| *rps7* | s7 | + | + | + | ψ |  |  |  | + | + | + |
| *rps8* | s8 |  | + | ψ | ψ | ψ |  |  |  |  |  |
| *rps10* | s10 | + | + | ψ |  | + |  |  | + |  |  |
| *rps11* | s11 | + | + | + | ψ | + |  |  | + | + |  |
| *rps12* | s12 | + | + | + | ψ | + |  |  | + | + | + |
| *rps13* | s13 |  | + | + | + | + |  |  | + | + |  |
| *rps14* | s14 | + | + | + | + | + |  |  | + | + | + |
| *rps19* | s19 | + | + | + |  | + |  |  | + | + |  |
| *rrn5* | r5 | + | + | + | + | ++ | + |  | + | + | + |
| **gene/species** |  | ***Ch.vu.*** | ***Ma.po.*** | ***Ph.pa.*** | ***Me.ae.*** | ***Hu. sq.*** | ***Is. en.*** | ***Se*.*mo*.** | ***Cy.ta.*** | ***Or.sa.*** | ***Br.na.*** |
| *rrn18* | r18 | + | + | + | + | ++ | + | + | + | + | + |
| *rrn26* | r26 | + | + | + | + | ++ | + | + | + | + | + |
| *rtl*^3^ | x1 | + | + | ψ |  |  |  |  |  |  |  |
| *sdh3* | d3 | + | + | + | ψ | + | + |  | + |  |  |
| *sdh4* | d4 | + | + | + | + | + |  |  |  |  |  |
| *tatC* | w2 | + | + | + | + | + | + | + | + | + | + |
| *trnAugc* | ta | + | + | + | + | + |  |  |  |  |  |
| *trnCgca* | tc | + | + | + | + | + | + |  | + | ψ | + |
| ***trnCgca^cp^*** | tcc |  |  |  |  |  |  |  |  | + |  |
| *trnDguc* | td | + | + | + | + | + |  |  | + | + |  |
| ***trnDguc^cp^*** | tdc |  |  |  |  |  |  |  |  |  | + |
| *trnEuuc* | te | + | + | + | + | + | + |  | + | + | + |
| *trnFgaa* | tf | + | + | + | + | +ψ | + |  | + |  |  |
| ***trnFgaa^cp^*** | tfc |  |  |  |  |  |  |  |  | + |  |
| *trnGgcc* | tg | + | + | + | + | + | + |  | + |  | + |
| *trnGucc* | t2 | + | + | + |  |  |  |  |  |  |  |
| *trnHgug* | th | + | + | + | + | ++ |  |  |  |  |  |
| ***trnHgug^cp^*** | thc |  |  |  |  |  |  |  | + | + | + |
| *trnIcau* | ti | + | + | + | + | + | + |  | + | + | + |
| *trnIgau* | t3 | + |  |  |  |  |  |  |  |  |  |
| *trnKuuu* | tk | + | + | + | + | +ψ | + |  | + | + | + |
| *trnLcaa* | t5 | + | + | + | + | ++ |  |  | + |  |  |
| *trnLgag* | t6 |  |  |  |  | + |  |  |  |  |  |
| *trnLuaa* | t7 | + | + | + | + | + |  |  |  |  |  |
| *trnLuag* | t8 | + | + | + |  | + | ψ |  | + |  |  |
| *trnMcau* | tm | + | + | + | + | ++ | + |  |  |  |  |
| ***trnMcau^cp^*** | tmc |  |  |  |  |  |  |  | + | + | + |
| *trnMfcau* | t9 | + | ++ | + | + | +++ψ | + |  | ++++ | + | + |
| *trnNguu* | tn | + | + |  |  | + |  |  | + |  |  |
| ***trnNguu^cp^*** | tnc |  |  |  |  |  |  |  |  | + | + |
| *trnPugg* | tp | + | + | + | + | + | + |  | + | + | + |
| ***trnPugg^cp^*** | tpc |  |  |  |  |  |  |  |  | + |  |
| *trnQuug* | t10 | + | + | + | + | ++ | + |  |  | + | + |
| *trnRacg* | tr | + | + | + |  | ++ |  |  |  |  |  |
| *trnRccu* | t11 |  |  |  |  |  |  |  | + |  |  |
| *trnRucg* | t12 |  | + |  |  |  |  |  |  |  |  |
| *trnRucu* | t13 | + | + | + |  | ++ |  |  |  |  |  |
| ***trnRucu^cp^*** | t13c |  |  |  |  |  |  |  |  | + |  |
| *trnSgcu* | t14 | + | + |  |  | + |  |  | + | + | + |
| *trnSgga* | ts |  |  |  |  | + |  |  |  |  |  |
| ***trnSgga^cp^*** | tsc |  |  |  |  |  |  |  | + | + | + |
| *trnSuga* | t15 | + | + | + |  | + | + |  | + | + | + |
| **gene/species** |  | ***Ch.vu.*** | ***Ma.po.*** | ***Ph.pa.*** | ***Me.ae.*** | ***Hu. sq.*** | ***Is. en.*** | ***Se*.*mo*.** | ***Cy.ta.*** | ***Or.sa.*** | ***Br.na.*** |
| *trnTggu* | tt | + | + | + | + |  |  |  |  |  |  |
| *trnVuac* | tv | + | + | + |  | + |  |  |  |  |  |
| ***trnVuac^cp^*** | tvc |  |  |  |  |  |  |  | + |  |  |
| *trnWcca* | tw | + | + | + | + | + | + |  | + |  |  |
| ***trnWcca^cp^*** | twc |  |  |  |  |  |  |  |  | + | + |
| *trnYgua* | ty | + | ++ | + | + | + | + |  | + | + | + |

#### ^1^The second column represents abbreviated gene names used in Figure 2. The full species names are as follows (in the order as they appear): *Chara vulgaris*, *Marchantia polymorpha, Physcomitrella patens, Megaceros aenigmaticus, Huperzia squarrosa, Isoetes engelmannii,* *Selaginella moellendorffii*, *Cycas taitungensis, Oryza sativa,* and *Brassica napus*. “+” or “ψ” indicate presence of a functional gene or a pseudogene respectively. More than one plus sign indicates presence of duplicated copies.

^2^The genes *ccmFC* and *ccmFN* of land plants appear as a single gene *ccmF* in *Chara*. The gene *ccmFN* is split into *ccmFN1* and *ccmFN2* in *Brassica.*

^3^The gene *rtl* is located inside a group II intron, *nad3i211* in *Chara* and *nad9i283* in *Physcomitrella.*
