# Supplementary material for: The Mitochondrial Genome of the Lycophyte Huperzia squarrosa: The Most Archaic Form in Vascular Plants
Source: PLoS One. 2012 Apr 12;7(4):e35168. doi: 10.1371/journal.pone.0035168 (PMC3325193; doi:10.1371/journal.pone.0035168)
Supplement: Table S3 — Intron contents in mitochondrial genomes of selected charophyte and land plants. (DOC) [file pone.0035168.s007.doc]

**Table S3.** Intron contents in mitochondrial genomes of selected charophyte and land plants1.

| **intron/species** | ***Ch. vu.*** | ***Ma. po.*** | ***Ph. pa.*** | ***Me. ae.*** | ***Hu. sq.*** | ***Is. en.*** | ***Se. mo.*** | ***Cy. ta.*** | ***Or. sa.*** | ***Br. na.*** |
| --- | --- | --- | --- | --- | --- | --- | --- | --- | --- | --- |
| *atp1i805g2* |  |  |  | + |  |  |  |  |  |  |
| *atp1i989g2* |  | + |  |  |  |  |  |  |  |  |
| *atp1i1019g2* |  |  |  | + |  |  |  |  |  |  |
| ***atp1i1050g2***** |  | + |  | + |  |  |  |  |  |  |
| *atp1i1129g2* |  |  | + |  |  |  |  |  |  |  |
| ***atp6i80g2****** |  |  | + | + | + |  |  |  |  |  |
| ***atp6i439g2***** |  |  |  | + | + | + | + |  |  |  |
| ***atp9i21g2***** |  |  | + |  | + | + | *trans* |  |  |  |
| ***atp9i87g2****** |  | + | + |  | + | + | + |  |  |  |
| ***atp9i95g2****** |  |  | + | + | + |  | + |  |  |  |
| *atp9i145g2* | + |  |  |  |  |  |  |  |  |  |
| *atp9i214g2* | + |  |  |  |  |  |  |  |  |  |
| ***ccmFCi829g2****** |  |  | + | + |  |  |  | + | + | + |
| *cobi274g2* | + |  |  |  |  |  |  |  |  |  |
| *cobi372g2* |  | + |  |  |  |  |  |  |  |  |
| *cobi420g1* |  |  | + |  |  |  |  |  |  |  |
| *cobi537g2* | + |  |  |  |  |  |  |  |  |  |
| *cobi688g2* | + |  |  |  |  |  |  |  |  |  |
| *cobi693g2* |  |  |  |  | + | + | + |  |  |  |
| *cobi783g2* |  | + |  |  |  |  |  |  |  |  |
| ***cobi787g2***** |  |  |  | + | + | + | *trans* |  |  |  |
| *cobi824g2* |  | + |  |  |  |  |  |  |  |  |
| *cobi838g2* |  |  |  | + |  |  |  |  |  |  |
| ***cox1i44g2***** |  | + |  | + |  |  |  |  |  |  |
| *cox1i150g2* |  |  |  | + |  |  |  |  |  |  |
| *cox1i178g2* |  | + |  |  |  |  |  |  |  |  |
| *cox1i211g2* | + |  |  |  |  |  |  |  |  |  |
| *cox1i227g2* |  |  |  |  |  | + | + |  |  |  |
| *cox1i266g2* |  |  |  |  |  | + | + |  |  |  |
| *cox1i323g2* |  |  |  |  | + | + |  |  |  |  |
| *cox1i375g1* |  | + |  |  |  |  |  |  |  |  |
| ***cox1i395g1***** |  | + |  |  |  | + |  |  |  |  |
| ***cox1i511g2****** |  | + | + |  |  |  | + |  |  |  |
| ***cox1i624g1***** |  | + | + |  |  |  |  |  |  |  |
| ***cox1i729g1***** | + | + |  |  |  |  |  |  |  |  |
| *cox1i732g2* |  |  | + |  |  |  |  |  |  |  |
| *cox1i740g1* | + |  |  |  |  |  |  |  |  |  |
| *cox1i835g2* | + |  |  |  |  |  |  |  |  |  |
| ***cox1i876g1***** | + |  |  |  |  |  | + |  |  |  |
| *cox1i909g1* | + |  |  |  |  |  |  |  |  |  |
| *cox1i995g2* |  |  |  |  | + | + | + |  |  |  |
| *cox1i1064g2* |  |  | + |  |  |  |  |  |  |  |
| **intron/species** | ***Ch. vu.*** | ***Ma. po.*** | ***Ph. pa.*** | ***Me. ae.*** | ***Hu. sq.*** | ***Is. en.*** | ***Se. mo.*** | ***Cy. ta.*** | ***Or. sa.*** | ***Br. na.*** |
| *cox1i1116g1* |  | + |  |  |  |  |  |  |  |  |
| *cox1i1149g2* |  |  |  |  | + |  | + |  |  |  |
| *cox1i1298g2* |  |  |  | + |  |  |  |  |  |  |
| ***cox1i1305g1***** |  | + |  |  |  | *trans* | *trans* |  |  |  |
| *cox2i94g2* |  |  |  |  | + | + | + |  |  |  |
| *cox2i97g2* |  | + |  |  |  |  |  |  |  |  |
| ***cox2i104g2***** | + |  | + |  |  |  |  |  |  |  |
| *cox2i250g2* |  | + |  |  |  |  |  |  |  |  |
| *cox2i281g2* |  |  |  | + |  |  |  |  |  |  |
| ***cox2i373g2******* |  |  | + | + | + |  | *trans* | + | + |  |
| *cox2i564g2* |  |  |  |  |  |  |  |  |  |  |
| ***cox2i691g2****** |  |  | + |  | + |  | + | + |  | + |
| ***cox3i171g2***** |  | + |  |  | + |  |  |  |  |  |
| *cox3i506g2* |  |  | + |  |  |  |  |  |  |  |
| *cox3i625g2* |  | + |  |  |  |  |  |  |  |  |
| ***nad1i287g2***** |  |  | + | + |  |  |  |  |  |  |
| *nad1i348g2* |  |  |  | + |  |  |  |  |  |  |
| ***nad1i394g2***** |  |  |  |  | + | + | + | *trans* | *trans* | *trans* |
| ***nad1i477g2***** |  |  |  |  | + |  | + | + | + | + |
| ***nad1i669g2***** |  |  |  |  | + |  | + | *trans* | *trans* | *trans* |
| ***nad1i728g2******* |  |  | + | + | + |  | + | + | *trans* | + |
| ***nad2i156g2****** |  |  | + |  | + | + | + | + | + | + |
| ***nad2i542g2***** |  |  |  |  |  | + | + | *trans* | *trans* | *trans* |
| ***nad2i709g2******* |  | + |  | + |  | + | + | + | + | + |
| *nad2i830g2* |  |  |  |  | + | + | + |  |  |  |
| ***nad2i1282g2***** |  |  |  | + |  |  |  | + | + | + |
| ***nad3i52g2***** |  |  |  | + | + | + | + |  |  |  |
| ***nad3i140g2******* | + | + |  | + | + | + | + |  |  |  |
| *nad3i211g2* | + |  |  |  |  |  |  |  |  |  |
| ***nad4i461g2******* |  |  | + | + | + | + | + | + | + | + |
| *nad4i548g2* |  | + |  |  |  |  |  |  |  |  |
| ***nad4i976g2******* | + |  |  | + |  |  | + | + | + | + |
| ***nad4i1399g2***** |  |  |  |  |  | + | + | + |  | + |
| *nad4Li100g2* |  | + |  |  |  |  |  |  |  |  |
| ***nad4Li283g2***** |  | + | + |  |  |  |  |  |  |  |
| ***nad5i230g2****** |  |  | + | + |  |  |  | + | + | + |
| *nad5i392g2*^2^ |  |  |  |  | + |  |  |  |  |  |
| ***nad5i753g1***** |  | + | + |  |  |  |  |  |  |  |
| *nad5i1242g2* |  |  |  |  | + | + | + |  |  |  |
| ***nad5i1455g2******* |  |  | + | + | + | + | + | *trans* | *trans* | *trans* |
| ***nad5i1477g2****** |  |  |  | + | + | + | + | *trans* | *trans* | *trans* |
| *nad5i1872g2* |  |  |  |  |  |  |  | + | + | + |
| *nad6i444g2* |  |  |  | + |  |  |  |  |  |  |
| **intron/species** | ***Ch. vu.*** | ***Ma. po.*** | ***Ph. pa.*** | ***Me. ae.*** | ***Hu. sq.*** | ***Is. en.*** | ***Se. mo.*** | ***Cy. ta.*** | ***Or. sa.*** | ***Br. na.*** |
| ***nad7i140g2****** |  |  | + |  |  |  | + | + | + | + |
| ***nad7i209g2****** |  |  | + |  |  | + | + | + | + | + |
| *nad7i336g2* |  | + |  |  |  |  |  |  |  |  |
| ***nad7i676g2***** |  |  |  |  |  | + | + | + | + | + |
| ***nad7i917g2***** |  |  |  |  |  | + | + | + | + | + |
| ***nad7i1113g2***** |  | + |  |  |  | + |  |  |  |  |
| *nad9i246g2* |  |  |  | + |  |  |  |  |  |  |
| *nad9i283g2* |  |  | + |  |  |  |  |  |  |  |
| *nad9i502g2* |  |  |  | + |  |  |  |  |  |  |
| *rpl2i28g2* |  | + |  |  |  |  |  |  |  |  |
| ***rpl2i917g2***** |  |  |  |  | + |  |  | + | + | + |
| ***rps3i74g2****** | + |  |  |  | + | + |  | + | + | + |
| ***rps3i257g2***** |  |  |  |  | + |  |  | + |  |  |
| ***rps10i235g2***** |  |  |  |  | + |  |  | + |  |  |
| ***rps14i114g2***** |  | + |  |  | + |  |  |  |  |  |
| *rrn18i839g1* |  |  |  |  |  | + | + |  |  |  |
| *rrn18i1065g2* |  | + |  |  |  |  |  |  |  |  |
| *rrn26i819g1* | + |  |  |  |  |  |  |  |  |  |
| *rrn26i827g2* |  | + |  |  |  |  |  |  |  |  |
| *rrn26i1871g1* | + |  |  |  |  |  |  |  |  |  |
| *rrn26i1879g1* | + |  |  |  |  |  |  |  |  |  |
| *rrn26i1891g1* | + |  |  |  |  |  |  |  |  |  |
| *rrn26i2191g1* | + |  |  |  |  |  |  |  |  |  |
| *rrn26i2429g1* | + |  |  |  |  |  |  |  |  |  |
| *rrn26i2462g1* | + |  |  |  |  |  |  |  |  |  |
| *rrn26i2500g1* | + |  |  |  |  |  |  |  |  |  |
| *rrn26i2513g1* | + |  |  |  |  |  |  |  |  |  |
| ***sdh3i100g2***** |  |  | + | + |  |  |  |  |  |  |
| *trnNguui38g2* | + |  |  |  |  |  |  |  |  |  |
| *trnSgcui43g2* |  | + |  |  |  |  |  |  |  |  |
| *trnVuac^cp^i39g2* |  |  |  |  |  |  |  | + |  |  |

#### ^1^The full species names are as follows (in the order as they appear): *Chara vulgaris, Marchantia polymorpha, Physcomitrella patens, Megaceros aenigmaticus, Huperzia squarrosa, Isoetes engelmannii,* *Selaginella moellendorffii*, *Cycas taitungensis, Oryza sativa,* and *Brassica napus*. “+” indicates presence of an intron, and “*trans*” denotes a *trans*-splicing intron. Introns present in more than one clade are bold-faced, and the number of stars indicates the number of plants clades in which the intron is present (clades recognized here are *Chara*, liverworts, mosses, hornworts, lycophytes, and seed plants). Intron nomenclature follows .

^2^The intron *nad5i392g2* was first discovered in *Huperzia selago* and was erroneously called *nad5i391g2* in a previous study .

**Reference**

1. Dombrovska O, Qiu Y-L (2004) Distribution of introns in the mitochondrial gene *nad1* in land plants: phylogenetic and molecular evolutionary implications. Mol Phylogen Evol 32: 246-263.

2. Knoop V (2004) The mitochondrial DNA of land plants: peculiarities in phylogenetic perspective. Curr Genet 46: 123-139.

3. Vangerow S, Teerkorn T, Knoop V (1999) Phylogenetic information in the mitochondrial nad5 gene of pteridophytes: RNA editing and intron sequences. Plant Biol 1: 235-243.
